# Standardised data on initiatives—STARDIT: Beta version

**DOI:** 10.1186/s40900-022-00363-9

**Published:** 2022-07-19

**Authors:** Jack S. Nunn, Thomas Shafee, Steven Chang, Richard Stephens, Jim Elliott, Sandy Oliver, Denny John, Maureen Smith, Neil Orr, Jennifer Preston, Josephine Borthwick, Thijs van Vlijmen, James Ansell, Francois Houyez, Maria Sharmila Alina de Sousa, Roan D. Plotz, Jessica L. Oliver, Yaela Golumbic, Rona Macniven, Samuel Wines, Ann Borda, Håkon da Silva Hyldmo, Pen-Yuan Hsing, Lena Denis, Carolyn Thompson

**Affiliations:** 1 Director of Science for All (Education Charity Registered in Australia), Melbourne, Australia; 2 School of Public Health, La Trobe University, Melbourne, VIC Australia; 3 School of Life Sciences, La Trobe University, Melbourne, VIC Australia; 4 La Trobe University, Melbourne, VIC Australia; 5 Patient Advocate, Co-Editor-in-Chief, ‘Research Involvement and Engagement’, London, UK; 6 Public Involvement Lead at Health Research Authority (England), London, UK; 7 Professor of Public Policy at UCL Social Research Institute, London, UK; 8 University of Johannesburg, Johannesburg, South Africa; 9 Adjunct Professor, Ramaiah University of Applied Sciences, Bengaluru, India; 10 Chair, Campbell and Cochrane Economic Methods Group, London, UK; 11 Cochrane Consumer Executive Chair, Ottawa, Canada; 12 Department of Linguistics, Faculty of Medicine, Health and Human Sciences, Macquarie University, Sydney, Australia; 13 Poche Centre Indigenous Health, The University of Sydney, Sydney, Australia; 14 National Institute for Health and Care Research, Alder Hey Clinical Research Facility, Liverpool, UK; 15 University of Melbourne, Melbourne, Australia; 16 Taylor and Francis, Abingdon, UK; 17 Consumers Health Forum of Australia, Deakin, Australia; 18 European Organisation for Rare Diseases, Paris, France; 19 Independent Impact Intelligence Design & Strategy Consultant, Research Impact Academy Brazil Ambassador, Sao Paulo, Brazil; 20 Applied Ecology and Environmental Change Research Group, Institute for Sustainable Industries and Liveable Cities, Victoria University, Melbourne, Australia; 21 Australian Citizen Science Association, Sydney, Australia; 22 University of Sydney, Sydney, Australia; 23 The Poche Centre for Indigenous Health, Faculty of Medicine and Health, The University of Sydney, Sydney, NSW 2006 Australia; 24 School of Population Health, Faculty of Medicine and Health, University of New South Wales, Sydney, Sydney, 2052 Australia; 25 Faculty of Medicine, Health and Human Sciences, Macquarie University, Sydney, 2109 Australia; 26 Co-Labs Melbourne, Melbourne, Australia; 27 University College London, London, UK; 28 Department of Geography, Norwegian University of Science and Technology, Trondheim, Norway; 29 University of Bath, Bath, UK; 30 MammalWeb Project, London, UK; 31 Johns Hopkins University, Baltimore, USA; 32 Institute of Zoology, Zoological Society of London, London, UK

**Keywords:** Data, Open, Standardised, Participatory, Democracy, Evidence, Systematic, Genomics, Health, Indigenous

## Abstract

**Background and objective:**

There is currently no standardised way to share information across disciplines about initiatives, including fields such as health, environment, basic science, manufacturing, media and international development. All problems, including complex global problems such as air pollution and pandemics require reliable data sharing between disciplines in order to respond effectively. Current reporting methods also lack information about the ways in which different people and organisations are involved in initiatives, making it difficult to collate and appraise data about the most effective ways to involve different people. The objective of STARDIT (**Sta**nda**r**dised **D**ata on **I**ni**t**iatives) is to address current limitations and inconsistencies in sharing data about initiatives. The STARDIT system features standardised data reporting about initiatives, including who has been involved, what tasks they did, and any impacts observed. STARDIT was created to help everyone in the world find and understand information about collective human actions, which are referred to as ‘initiatives’. STARDIT enables multiple categories of data to be reported in a standardised way across disciplines, facilitating appraisal of initiatives and aiding synthesis of evidence for the most effective ways for people to be involved in initiatives. This article outlines progress to date on STARDIT; current usage; information about submitting reports; planned next steps and how anyone can become involved.

**Method:**

STARDIT development is guided by participatory action research paradigms, and has been co-created with people from multiple disciplines and countries. Co-authors include cancer patients, people affected by rare diseases, health researchers, environmental researchers, economists, librarians and academic publishers. The co-authors also worked with Indigenous peoples from multiple countries and in partnership with an organisation working with Indigenous Australians.

**Results and discussion:**

Over 100 people from multiple disciplines and countries have been involved in co-designing STARDIT since 2019. STARDIT is the first open access web-based data-sharing system which standardises the way that information about initiatives is reported across diverse fields and disciplines, including information about which tasks were done by which stakeholders. STARDIT is designed to work with existing data standards. STARDIT data will be released into the public domain (CC0) and integrated into Wikidata; it works across multiple languages and is both human and machine readable. Reports can be updated throughout the lifetime of an initiative, from planning to evaluation, allowing anyone to be involved in reporting impacts and outcomes. STARDIT is the first system that enables sharing of standardised data about initiatives across disciplines. A working Beta version was publicly released in February 2021 (ScienceforAll.World/STARDIT). Subsequently, STARDIT reports have been created for peer-reviewed research in multiple journals and multiple research projects, demonstrating the usability. In addition, organisations including Cochrane and Australian Genomics have created prospective reports outlining planned initiatives.

**Conclusions:**

STARDIT can help create high-quality standardised information on initiatives trying to solve complex multidisciplinary global problems.

**Supplementary Information:**

The online version contains supplementary material available at 10.1186/s40900-022-00363-9.

## Plain English Summary

All major problems, including complex global problems such as air pollution and pandemics, require reliable data sharing between disciplines in order to respond effectively. Such problems require evidence-informed collaborative methods, multidisciplinary research and interventions in which the people who are affected are involved in every stage. However, there is currently no standardised way to share information about initiatives and problem-solving across and between fields such as health, environment, basic science, manufacturing, education, media and international development. A multi-disciplinary international team of over 100 citizens, experts and data-users has been involved in co-creating STARDIT to help everyone in the world share, find and understand information about collective human actions, which are referred to as ‘initiatives’. STARDIT is an open access data-sharing system to standardise the way that information about initiatives is reported, including information about which tasks were done by different people. Reports can be updated at all stages, from planning to evaluation, and can report impacts in many languages, using Wikidata. STARDIT is free to use, and data can be submitted by anyone. Report authors can be verified to improve trust and transparency, and data checked for quality. STARDIT can help create high-quality standardised information on initiatives trying to solve complex multidisciplinary global problems. Among its main benefits, STARDIT offers those carrying out research and interventions access to standardised information which enables well-founded comparisons of the effectiveness of different methods. This article outlines progress to date; current usage; information about submitting reports; planned next steps and how anyone can become involved.

**Supplementary Information:**

The online version contains supplementary material available at 10.1186/s40900-022-00363-9.

## Introduction

### Background

Many problems facing life on earth transcend the capacity of any single discipline to address. For example, problems such as pandemics, air pollution and biodiversity destruction cannot be characterised solely as ‘public health’, ‘environment’ or ‘education’ problems [1, 2]. Solving such problems calls for holistic approaches [3] and will require governments, industry, research organisations and people around the world to work in partnership.

People need access to valid and reliable information to make informed decisions [4], which typically requires evidence. Depending on the context, this evidence-informed approach is called ‘research’, ‘evaluation’ [5], ‘international development’, ‘education’ or an ‘initiative’. Hereafter all of the above will be referred to as ‘initiatives’. For example, when deciding a response to a pandemic, standardised data can improve retrieval of relevant information which can be used to inform which affected individuals or organisations could be involved in the design of the response and which outcomes are most important [6]. This can include deciding which stakeholders should be involved in which tasks, such as prioritising outcomes.

In this article we explain how S**ta**nda**r**dised **D**ata on **I**ni**t**iatives (STARDIT) builds on work to date by standardising a wide variety of data in a format applicable across multiple sectors, disciplines and languages. It is hoped that the creation of this evidence base will add to understanding and evaluating what works, for whom, why, and in what circumstances [7–10]. Hereafter, data generated by an initiative (including raw data), information about the data (meta-data) and information about the initiative will all be referred to as ‘data’ unless otherwise specified.

In 2020, the United Nations Secretary-General stated that ‘purposes that involve data and analytics permeate virtually all aspects of our work in development, peace and security, humanitarian, and human rights’, encouraging ‘everyone, everywhere’ to ‘nurture data as a strategic asset for insight, impact and integrity—to better deliver on our mandates for people and planet’ [11]. Similarly, the United Nation’s Paris Agreement highlighted the critical role of ‘sharing information, good practices, experiences and lessons’ in response to preventing irreversible climate change [12]. While organisations such as Cochrane (health) and The Campbell Collaboration (social sciences) are working to create high-quality systematic reviews of medical, social and economic initiatives, there remain limitations to the data available for such reviews. After a recommendation from the Organisation for Economic Co-operation and Development (OECD), successful data sharing initiatives in biodiversity exist, such as the Global Biodiversity Information Facility (GBIF) [13], however there also remain limitations and accessibility issues in sharing and standardising biodiversity data [14, 15].

It is often essential to include those affected by initiatives in the design and delivery of those initiatives [16]. For example, with an initiative to respond to a pandemic, those creating and delivering an initiative, and those affected by the outcome may be the same people. Forms of participatory action research where anyone can be involved in any aspect of research [17] (including amorphous terms such as ‘citizen science’ [18]) are increasingly recognised as crucial paradigms for solving such global problems, as they can help ensure that initiatives are aligned with the priorities of those affected [19–21]. However, while the importance of involving people is clear [7], evidence-informed methods of doing so are limited [9, 22–26].

A recent statement defined a role for the public in ‘data intensive’ health research [27]. While in the health research disciplines there are over 60 different tools or frameworks for reporting or supporting public involvement, most published tools or frameworks are not used beyond the groups that developed them, and none work across multiple disciplines or languages [28]. Current reporting methods also lack information about the ways in which different people are involved in initiatives, making it difficult to collate and appraise data about the most effective ways to involve different people. In addition, ‘citizen science’ and ‘participatory action research’ are blurring the lines between concepts such as ‘researcher’, ‘public’, ‘patient’ and ‘citizen’ [9, 29–33].

The STARDIT tool features standardised data reporting about initiatives, including who has been involved, what tasks they did, and any impacts observed. STARDIT was created to help everyone in the world find and understand information about collective human actions, which are referred to as ‘initiatives’. In addition to providing new standardised data categories for describing who was involved in which tasks of an initiative, STARDIT can also incorporate the many existing data standards (see Additional file 1 ‘Using Standardised Data on Initiatives (STARDIT): Beta Version Manual’), thus creating a unifying system for data hosting, linking and analysis. STARDIT can also report any different ‘interests’ of stakeholders and the ways power is shared between different stakeholders. The word ‘stakeholders’ here includes the public, those who have important knowledge, expertise or views that should be taken into account and others with a ‘stake’ in an initiative [34, 35].

Stakeholders can also include people who have financial, professional, social or personal ‘interests’. An ‘interest’ can include a kind of commitment, goal, obligation, duty or sense of connection which relates to a particular social role, practice, profession, experience, medical diagnosis or genomic variation [36]. These can include financial or other interests which may compete or conflict with ‘public interest’ [37]. For example, a systematic review found that industry funded research is more likely to have outcomes favouring those with financial interests who are sponsoring the research [37, 38]. Other examples include people from certain sub-populations (including those from populations more likely to be exploited [39]), Indigenous peoples, or people affected by rare diseases may have a personal interest in initiatives relevant to those specific populations, separate to the ‘general public’ [9, 40–42]. For example a person with a rare disease may have a personal ‘interest’ in research into a treatment for that disease [42]. STARDIT allows standardised reporting of stakeholders and any interests.

Sharing data in a consistent way may help ensure that benefits of initiatives are shared more equitably (for example, by improving accountability) [9]. In addition sharing information about who ‘owns’ or controls access to data and how such data access decisions are made can help people make informed decisions about participating in research [42]. By reporting involvement in initiatives, STARDIT also allows acknowledgement of people otherwise excluded from the public record—such as patients, people donating personal data, medical writers, laboratory assistants, citizen scientists collecting or analysing data, custodians of traditional or Indigenous knowledge, translators, interviewers, coders and code reviewers.

### Objective

The objective of STARDIT is to address current limitations and inconsistencies in sharing data about initiatives. The STARDIT tool features standardised data reporting about initiatives, including who has been involved, what tasks they did, and any impacts observed. STARDIT is designed to support a culture of partnership across disciplines and beyond, and is, wherever possible, aligned and interoperable with existing reporting models and frameworks such as those used in health, environment, manufacturing, publishing, government policy, education, arts and international development (see Table 1). In addition, the STARDIT Preference Mapping (STARDIT-PM) tool provides a standardised way to report information about different stakeholders’ preferences, including preferences for power-sharing and methods of involving people during an initiative (see section ‘Mapping preferences for involvement’).

**Table 1:** Example applications of STARDIT

Area	Sub-area	Relevant data categories
Research	Health research	Reporting: Funding, conflicting or competing interests, co-design, experts involved, people affected involved, methods, process for deciding and measuring outcomes, protocols, who is accountable for ensuring protocol is followed, information about data storage, sharing, ownership and custodianship, information about data security practices and standards, information about consent and withdrawal processes evaluation of entire research process, ethical review, information about data analysis and data validation
	Social research	
	Genomics research	
	Environmental research	
Policy	Health and social policy	Reporting: Values of people involved, sources of data and evidence, data on past and current initiatives and spending [156], process for policy (or proposed policy) creation, process for deciding and measuring outcomes, experts involved, people affected involved, policy or manifesto writers, conflicting or competing interests of people involved, purpose of policy (what needs have been identified, how and by who), outcomes from policy (including outcomes measured by those affected by policy), policy evaluation (reporting if it achieved what was intended)
	Other government policy (transport, arts, education, environment etc.)	
	Foreign policy	
	Proposed policy (including draft policy and manifestoes)	
	International development	
Education and learning	Educational initiatives	Reporting: Sources of data and evidence for intervention, purpose of intervention, process for educational intervention creation, funding, conflicting or competing interests, experts involved, people affected involved, conflicting or competing interests, process for deciding and measuring outcomes, outcomes from intervention, evaluation of intervention, ethical review
Arts	Community arts projects	Reporting: Purpose of project, process for project design and implementation, experts involved, people from communities intended to benefit involved, funding, conflicting or competing interests, process for evaluating project, project evaluation, project outcomes
	Arts funding	Reporting: People involved in deciding funding process, purpose of funding, people allocating funding (funding sources), funding amount, conflicting or competing interests, process for deciding outcomes of funding, evaluating the funding allocation process
Information, media and cultural heritage	Health and medical information	Reporting: People involved in researching, writing (including medical writers), creating, reviewing (including peer reviewers), disseminating and funding, information about any potential risks (to human health or lifeforms, natural or cultural heritage), information about who assessed those risks and how (for example, medical information standards [147]), information about consent to appear in images and verified appearances of public figures, information about ownership of data or knowledge (including concepts of intellectual property, copyright and licence information, relevant blockchains and non-fungible tokens), evaluating knowledge translation, reporting impacts and outcomes [89]
	Disaster and emergency communication	
	Public interest, factual information commentary, documentaries and other informative media	
	Intangible cultural heritage (including folklore, traditions, language), traditional, local and Indigenous knowledge and wisdom	Reporting: Who created any content containing the Indigenous or traditional knowledge, what tasks they had, how this knowledge was shared and any relevant concepts of ‘owning’ or ‘property’; reporting who knows certain things (for example, people who are recognised as ‘Preservers of Important Intangible Cultural Properties’ [157]); reporting who is recognised as an Elder, community leader, Indigenous elders or leaders (and by who); reporting who does or does not have permission to verify, share, redact or edit content (including stories, beliefs, cultural practices and medicine) [158]; information about data custodianship [50], information about any potential risks (to human health or lifeforms, natural or cultural heritage); information about who assessed those risks and how, information about informed consent process, information about any cultural sensitivities or restrictions (including relevant information about gender, clan, tribe or other culturally constructed groupings) [159–161], information about relevant laws and lore [50], ethics processes and their impacts (including who was involved and how) [162], reporting impacts and outcomes from dissemination [89]
	Tangible cultural heritage (including cultural property [163])	Reporting: Who was involved in creating the property, any concepts of ownership or guardianship in relation to the property, data about ongoing management (including monitoring, exhibiting, restoring or moving), data about cultural significance and stakeholders involved in defining this
	Hardware designs (including hardware architecture, device designs or other abstract representations)	Reporting: Who was involved in creating the designs and how, who reviewed them and how (including relevant safety, regulation or standards information), what formats are the designs shared as and in what medium, information on licence(s), outcomes and impact of the hardware
	Code and algorithms	Reporting: Who created code (including algorithms), who is involved in reviewing and scrutinising code (including who is involved in which ethical review processes), what code is part of which distinct projects or forks, what language the code is in, what medium (for example, machine or DNA), information about ownership of data or knowledge (including concepts of intellectual property and copyright), information on licence(s), purpose of code, outcomes and impact of the code
Management and monitoring	Environmental and natural heritage, natural resource management	Reporting: Data about who was involved in service design, monitoring and management processes, data about funding for monitoring or management (for example, funding for pollution monitoring), data about how information will be stored and shared (including what will be redacted and data security), data about who decides what data will be redacted and how this decision is made, information about how data will be analysed (including relevant code and algorithms) and how learning from data will be shared, information about relevant data privacy legislation and regulation
	Public and private essential services management (health, infrastructure, waste and recycling, water and sewage, electricity)	
	Data management and monitoring	
Evaluation	Process evaluation	Reporting: Data about processes (industrial, public health, organisational) [5], people involved, outcomes
	Evaluation of participatory methods	Reporting: Data about participatory research methods (including ‘citizen science’ [18], ‘co-design’, ‘co-production’ and ‘co-evaluation’, ‘participatory action research’ [17] and ‘public involvement’),evaluate methods and compare outcomes [164]
	Transparent rating	Reporting: Processes of transparency rating (or ‘scoring’) data quality about initiatives based on how much information about the initiatives is shared in a publicly accessible way (or reasons for redaction, including Indigenous knowledge)
Production, consumerism and business	Industry standards	Reporting: Internal processes and data sharing practices of self-regulating industry standards (for example, the Forest Stewardship Council, Marine Stewardship Council [54]. and Certified B Corporations [55].), data sharing principles, process evaluation (including by those affected)
	‘Green’ industries and eco-tourism	Reporting: Transparent process for defining ‘green’ and ‘eco’, experts involved, people affected involved, process for deciding and measuring outcomes, outcome measures, evaluation of process
	Infrastructure, construction and interiors	Reporting: Transparent reporting of sources of building and furniture materials, such as wood (including relevant DNA information to verify sources of timber), metals and other materials (including information verifying the supply chain is slavery free), data from building and structural assessments
	Finance and financial services	Reporting: Who is involved in decision making (including investment and divestment), who scrutinises decision making, who is involved in holding individuals to account and who scrutinises this process, competing or conflicting interests of people involved in decision making, data about how concepts such as ‘ethical investments’ are defined, impacts or outcomes from investments or donations, data sharing practices and security practices, data about who scrutinises security practices
	Donation and philanthropy	Reporting: Any stated purposes or caveats for donation, organisations or individuals donating, how money was spent, who was involved in deciding how it was spent, what was the method for deciding this, who is accountable for overseeing this, any outcomes or impacts
	Other products (medical devices, electronics)	Reporting: Experts involved in production, other people involved in production process, resources involved in production process (including relevant DNA information to verify products from plants, animals and fungi), ingredients, funding for resources (for example demonstrating it is ‘slavery free’), process reporting (including Good Manufacturing Practice), regulation and authorisation processes (for example medicines and medical devices), code and algorithm checking (for example, autonomous vehicles) process for designing impact assessment, impact assessment (including human and environmental), experts involved in dismantling process (including recycling), other people involved in dismantling process and disposal, evaluation of product according to transparently-decided outcome measures
Products for human use or ingestion	Food	
	Medicines	
Products for non-human lifeforms	Food	
	Medicines	
	Other products	
Health Technology assessment	Assessment process for pharmaceuticals, devices, procedures and organisational systems used in health care [165]	Reporting: Process for deciding health technology assessment (oversight and scrutiny), sources of data and evidence, process for deciding and measuring outcomes, experts involved, people affected involved, conflicting or competing interests, outcomes from assessment decisions (including outcomes measured by those affected by assessment decisions), collation of adverse event reports from Governments and reputable sources, assessment evaluation (did it achieve what was intended?), results of economic evaluations
Health and social care and services	Health care and services	Reporting: Process for assessing needs (including who was involved, the method and budget), process for prioritisation of services (including budgets and ‘rationing’ decisions), process for designing and implementing service or care (including who was involved, the method and the budget), process for evaluating service or care (including impacts), patterns for evaluating service improvement initiatives, process for reporting adverse events and malpractice (including the overview and scrutiny of this process), process for identifying patterns of sub-optimal service, process for responding to malpractice or other identified issues, process for identifying impact indicators (including geolocation data)
	Social care and services	
Other services		

In alignment with the UNESCO Recommendation on Open Science [43], the co-created values of the STARDIT project state that designs and code should always be open access and relevant licences should always be those which allow others to build on and improve the project, while maintaining central control over quality (such as the Creative Commons Attribution-ShareAlike 4.0 International license (CC BY-SA 4.0) and the GNU General Public License (GPL) 3.0 for code. STARDIT data will released into the public domain (CC0) and integrated into Wikidata, which is a free and open knowledge base for collaboratively editing structured data [44]. The working Beta Version of STARDIT uses Wikidata to enable definitions to be co-created by contributors anywhere in the world, and therefore works across human languages, with interoperability with other platforms planned for future versions.

### Potential applications

STARDIT’s potential applications are summarised in Table 1. Among the principal applications, STARDIT offers public access to standardised information which enables the comparison of methods with the most impacts, such as ways of involving stakeholders in initiatives. The United Nations defines assessing impact as ‘establishing cause and effect chains to show if an intervention has worked and, if so, how’ [45]. With more data being shared, STARDIT could support decision making when planning stakeholder involvement in initiatives, and enable more people to assess the rigour of impact assessments [45]. This will be achieved by structuring the data in a way to allow such comparisons between different outcomes and methods of involving people, including using machine learning algorithms (including artificial intelligence).

In addition, STARDIT could be used to share information which makes research more reproducible [46, 47], improving accessibility to the information required to critically appraise research and evidence and thus improving trust in processes such as the scientific method [48, 49], and facilitate an appraisal of different knowledge systems, including Indigenous knowledge systems [50]. Such data sharing could also improve the translation of trusted, quality research and data, by empowering people to both access and appraise relevant data. For example, improved access to more standardised information (in multiple languages) about data and outcomes, could help to facilitate more informed collaborations between researchers and those monitoring and protecting critically-endangered species, particularly where there is no common language [51–53].

In addition, many industries use self-regulatory processes to govern industry practices, with examples including the Forest Stewardship Council (FSC), Marine Stewardship Council (MSC) [54], Certified B Corporations [55], and multiple Good Manufacturing Practice (GMP) guidelines. STARDIT could be used to improve public awareness of, and access to, the data already reported by such self-regulatory standards. Increased transparency could, for example, support people to make informed decisions when investing or buying products; automate analysis of data to facilitate such decisions, and improve accountability overall.

### Defining ‘initiative’ and ‘involvement’

As STARDIT is designed to report data across disciplines, distinctions between concepts such as ‘intervention’, ‘research’, ‘project’, ‘policy’, ‘initiative’ (and similar terms) are of secondary importance compared with communicating ‘the aims or purposes of specified actions’; ‘who did which tasks or actions’; ‘are there competing or conflicting interests’, and the ‘outcomes from a specific action’. In this way, STARDIT can be used to report on any kind of collective action, which can include interventions, projects or initiatives—including a clinical study, education interventions or any kind of evaluation [5, 56, 57]. In this article, we use the word ‘initiative’ to describe any intervention, research or planned project which is a kind of collective human action. We define ‘involving’ people as the process of carrying out research, initiatives or interventions with people, rather than on them [58]. Involvement occurs when power is shared by researchers, research participants, and other relevant stakeholders (such as the public, industry representatives and experts). While meanings of these terms are often imprecise and can be used interchangeably, ‘involvement’ here is distinct from ‘engagement’. We consciously use 'involvement' rather than 'engagement' to emphasise active participation that goes beyond simply receiving information about initiatives. We use ‘engagement’ here to mean where information and knowledge about initiatives is shared, for example, with study participants who remain passive recipients of interventions [59–61].

#### Using and developing data standards

The current Beta Version of STARDIT maps terms and concepts using the Wikidata initiative (part of the Wikimedia Foundation) [36], which includes definitions (taxonomy), a way of describing relationships between concepts (ontology) [37], and a system to translate definitions and ontology between many languages. Examples of existing taxonomies include the National Library of Medicine’s Medical Subject Headings (MeSH), which are used extensively in multiple kinds of literature reviews [38].

How to involve people in combining or merging overlapping taxonomies for different subsets of data has been identified as an important question in the process of taxonomy [62, 63]. By using Wikidata, STARDIT can be used by anyone to store both publicly accessible data and meta data (data about data), and link to hosted structured linked data. While STARDIT is a novel element set, where possible it will also incorporate element sets from established data standards and map them where possible (see Table 6 in the Additional file 1 for examples of data standards which could be incorporated). This includes standard elements and value sets and controlled vocabularies [64]. The terms used in this paper are working terms, which will be progressively standardised over the lifetime of the project.

Structured Wikidata can help define terms and concepts clearly and unambiguously, in a transparent and open way. For example, colours in the spectrum are described by a standard numerical code in Wikidata, whereas the names of colours change according to different languages. Also, people with different DNA variations will also experience some colours differently. Similarly, the Wikidata entry for ‘patient’ has the human-readable definition of ‘person who takes a medical treatment or is subject of a case study’ (translated into 54 other languages) and a machine-readable definition consisting of dozens of semantic links to and from other Wikidata entries [39]. The terms ‘participant’ and ‘research participant’ are similarly coded, defined and translated. For terms that do not currently exist in Wikidata (for example, ‘biobank participant’), a definition can be contributed by anyone in any language, refined by other users, then coded and translated into multiple languages by Wikidata. Developing taxonomies and ontologies will be an ongoing process facilitated by the current Wikidata infrastructure, and may require creating additional tools to create more inclusive ways of involving people in developing taxonomies [40].

## Methods and paradigms

### Participatory action research

STARDIT development is guided by participatory action research (PAR) paradigms, which guide initiatives by aiming to involve all stakeholders in every aspect of the development and evaluation of an initiative [65, 66]. Participatory research is a form of collective, self-reflective enquiry undertaken by people in order to understand their situation from different perspectives [67]. Development has also been influenced by existing work in health research, including the multidisciplinary area of public health, which incorporates social, environmental and economic research. In a health context, participatory research attempts to reduce health inequalities by supporting people to be involved in addressing health issues that are important to them, data collection, reflection and ultimately in action to improve their own health [68]. At the core of participatory research is ‘critical reflexivity’. The process asks people involved to reflect on the causes of problems, possible solutions, take any actions required which might improve the current situation, and evaluate the actions [66].

### Rights-based paradigm

The United Nations (UN) Universal Declaration Human Rights states everyone should be able to ‘receive and impart information and ideas’ [69]. The UN also states that democracy, development and respect for all human rights and fundamental freedoms are interdependent and mutually reinforcing’ [70]. To uphold human rights and ‘environmental rights’ [71], and for ‘the maintenance of peace’, people require ‘media freedom’ in order to ‘seek, receive and impart information’ [70], free of unaccountable censorship. STARDIT has been created in order to help anyone uphold these universal rights, by providing a way to share open access information in a structured way with a transparent process for quality checking.

### Cultural neutrality

Values, assumptions, ways of thinking and knowing are not shared universally. The participatory process used for developing STARDIT required and will continue to require that it attempts to map cultural variations, in order to avoid unconsciously reinforcing particular (often ‘dominant’) [72] values. Transparent acknowledgement of differing values and perspectives is critically important, in particular when mapping if different stakeholders’ values are complementary or opposing. A participatory process requires mapping all of these perspectives and, where possible, involving people in labelling different perspectives and values. For example, STARDIT has already been used to map the varying perspectives of multiple stakeholders when planning a multi-generational cohort study [73].

Many problems facing humans are shared by non-human life forms and ecosystems, including rapid climate change, air pollution and sea-level rise. If initiatives are to operate in inclusive, culturally-neutral ways, reconsideration of the language used to describe relationships between humans, non-human life and the environment is essential [74]. Environmental and social sciences are challenging and redefining colonial-era concepts of what can be ‘owned’ as property or who ‘owns’ [74, 75]. As a result, ecosystems such as rivers and non-human animals, are being assigned ‘personhood’ [76–78]. For example, a public consultation by a ‘dominant’ group might ask, ‘who owns the rights to the water in a river system?’ [72]. This question imposes the dominant group’s values on people who may not share the same concept of ‘ownership’. In this way, Western European legal and economic traditions are frequently incompatible with those of some Indigenous peoples’ [74, 79, 80].

The participatory process used for developing STARDIT has attempted to be transparent about how different stakeholders have been involved in shaping it in order to improve how the system can be used to map values and provide more culturally neutral guidance for planning and evaluating involvement in initiatives. However, it is acknowledged that it will be a challenging process to ‘de-colonialise’ and ‘de-anthropocise’ language and action [81, 82], as this may be perceived as a challenge to some people’s cultural attitudes which may not align with the United Nation’s universally enshrined principles of democracy, human rights and environmental rights. In addition, ongoing co-design will be required to ensure STARDIT is as accessible and inclusive as possible.

### Development phases and methods

**Fig. 1:**
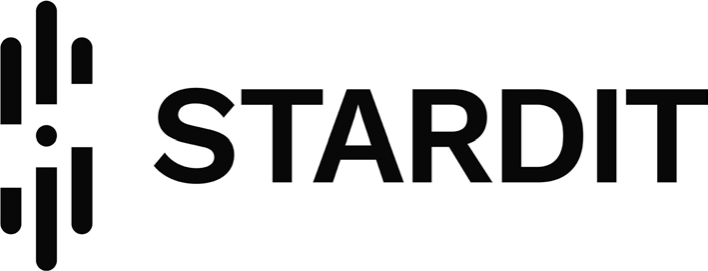
STARDIT Logo

Both the STARDIT Alpha version (0.1) and the Beta version (0.2) have already involved people from diverse disciplines and backgrounds in the development, as this is integral to its effectiveness (Fig. 1). It has been co-created using methodologies informed by PAR and other health research reporting guidelines [83]. PAR describes related approaches which involve experts (such as researchers), the public and other stakeholders “working together, sharing power and responsibility from the start to the end of the project” [84, 85].

The Alpha version of STARDIT (version 0.1) followed the recommendations of a 2019 scoping review led by Nunn et al., which mapped public involvement in global genomics research [9]. This review stated that ‘without a standardized framework to report and transparently evaluate ways people are involved, it will be difficult to create an evidence base to inform best-practice’ [9]. This review was followed by an additional review (conducted in 2020 led Nunn et al., and to be submitted for publication in 2022), which mapped international guidance for planning, reporting and evaluating initiatives across multiple disciplines, and found 158 different reporting standards and reporting guidelines across disciplines (see the preliminary results in Table 7 of Additional file 1) [86]. This included 7 different biodiversity reporting standards, and 15 different reporting standards for health research. STARDIT was also informed by a number of PAR projects [41, 87, 88], and a report for the Wikimedia Foundation by the charity Science for All [89].

The charity Science for All has hosted the co-creation process since 2019. Science for All is a charity based in Australia which supports everyone to get involved in shaping the future of human knowledge, with co-created values guiding their work [90]. Development was informed by a number of literature reviews and guidelines, with methods of involving people in the development of STARDIT guided by the Enhancing the Quality and Transparency of Health Research (EQUATOR) network’s approach to developing reporting guidelines [83, 91]. Methods of involving people included public events, online discussions and a consultation process. Owing to there being no formal budget for this project, the ability to actively involve people who can’t afford to volunteer their time for free was restricted. Details about how inclusive ways of involving people were used are included in the publication consultation report [92]. This includes information about working with people from lower, middle and high-income countries, Indigenous peoples from Australia and Indonesia, people affected by cancer and rare diseases from Europe and the Americas, and people with expert knowledge of protecting endangered animals and eco-systems. The STARDIT project is actively seeking funding from organisations which align with our values, in order to ensure the project is as inclusive as possible.

The co-creation process is currently being supported pro-bono by Science for All, and has also received in-kind support from individuals and organisations worldwide. A modified Delphi technique was used at some stages, with this method to be reviewed when co-creating future versions [93, 94]. Many people were invited to provide feedback on all aspects of STARDIT, including its feasibility, design and implementation. They could comment anonymously using online forms and shared documents, in online discussion forums, via email or during face-to-face or video meetings.

After the feedback from the Alpha version was collated, work began on the Beta version. Between January 2020 and August 2021 multiple meetings and presentations took place to inform the Beta version, with some planned face-to-face involvement cancelled owing to the COVID-19 pandemic. Online activities where feedback on STARDIT was invited and given included interactive presentations by Jack Nunn to the WikiCite 2020 Virtual conference [95], Poche Centre for Indigenous Health [96], Ludwig Boltzmann Gesellschaft [97], La Trobe University [98], Australian Citizen Science Association [99] and Rare Voices Australia. In addition, between February 2021 and May 2021, a total of 27 people provided feedback on the Beta version via the online form and collaborative document. Over 7000 words of feedback and comments were provided via the online form with 144 separate points, comments or corrections [92]. More detailed information about the consultation process for the Alpha and Beta versions up to May 2021 can be found in the 2020 and 2021 public consultation reports [92, 100] and in the Additional files 2, 3 and 4. Further information about who was involved in the Beta Version development and proposed future development phases can be found in the Additional file 1.

**Fig. 2:**
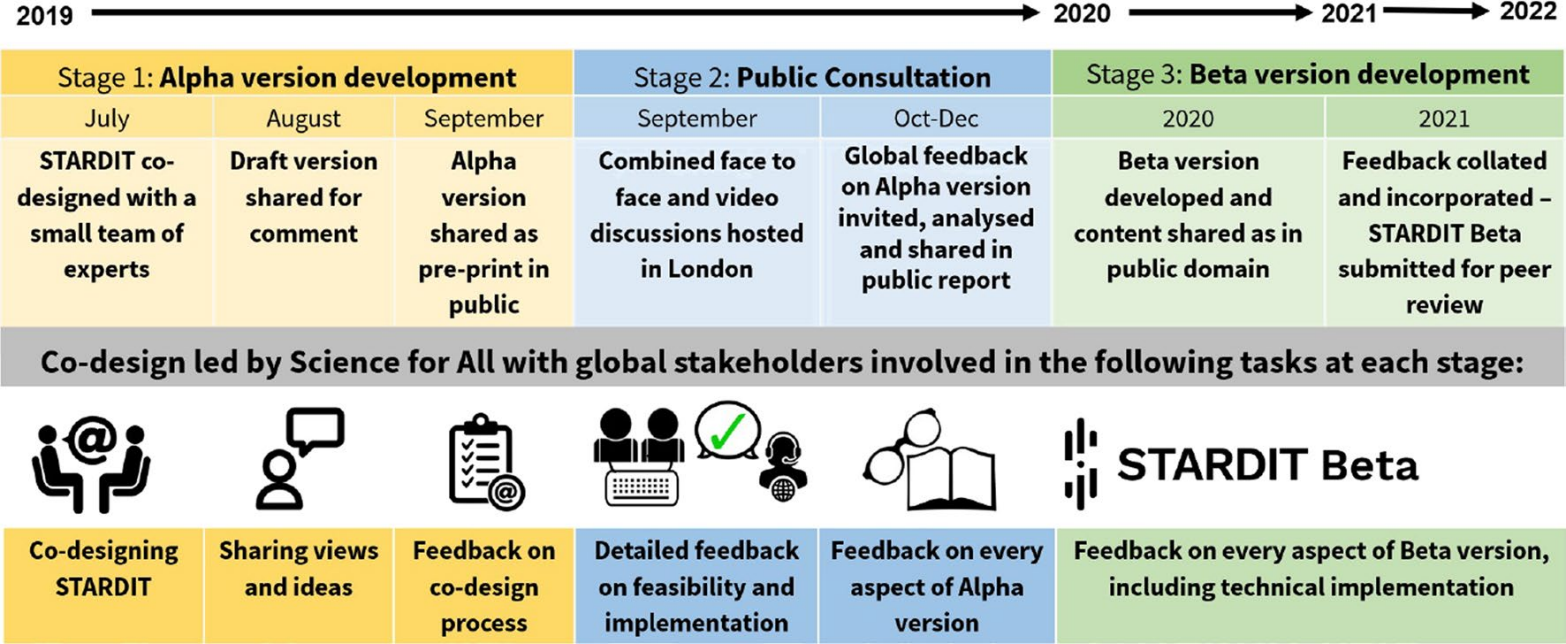
STARDIT development

Science for All also hosts an online working group which continues to guide the development of STARDIT according to the terms of reference [101]. Anyone is welcome to join the working group, contribute to discussions and vote on decisions and ensure alignment with other initiatives. STARDIT and all associated work and co-designed logos (see Fig. 1) are currently published under the Creative Commons Attribution-ShareAlike 4.0 International license (CC BY-SA 4.0) [102], with the quality of any future iterations being the responsibility of not-for-profit host organisations and future licensing decisions to be made transparent, with anyone invited to be involved. The co-design process so far is summarised in Fig. 2, with further information about the process available in Additional file 1.

#### Version one implementation

Now STARDIT Beta (version 0.2) has been published, a Beta version implementation article will be initiated, demonstrating the use of machine learning to generate STARDIT reports using mapped data from a number of international partner organisations. Work will then begin on the next version (version 1.0). Those involved with STARDIT development will disseminate information, gather feedback and recruit more people and organisations to participate as project partners and potentially funders. This stage is estimated to take between 2 and 3 years, at which point a working group will formally invite other appropriate partner organisations (such as the UN and WHO) to adopt the STARDIT framework. A Steering Group will be established to oversee and continually improve the STARDIT system. STARDIT will require continued working with publishers, research funders and governments to encourage adoption of the reporting tool. More detail on the proposed next stages can be found in the Additional file 1 in the section ‘Development phases’.

## Results

This section summarises the results from the process of co-designing STARDIT. Since the start of the project in 2019, over 100 people from multiple disciplines and countries have been involved in co-designing STARDIT. A working Beta version was publicly released in February 2021 (ScienceforAll.World/STARDIT). Subsequently, STARDIT reports have been created for peer-reviewed research in multiple journals and multiple research projects [41, 42, 87, 88, 103–105]. In addition, organisations including Cochrane [106, 107] and Australian Genomics [108] have created prospective STARDIT reports outlining planned initiatives that will use STARDIT to report them. The Cochrane Council voted to use STARDIT to report planned work on creating a values statement [106, 107], while the Australian Genomics working group ‘Involve Australia’ voted to use STARDIT to report their planned work [108].

### Beta version interface

A link to the working Beta version can be found at: ScienceforAll.World/STARDIT/Beta [109]. The data fields in the STARDIT system co-created during the process described in this article are summarised in Table 4. Table 5 presents the full version of the data fields. The ‘**Mi**nimum **C**ontribution **R**ep**o**rting Form’ (MICRO) specifies the minimum information required to make a STARDIT report and these fields are highlighted in the table and marked with an asterisk (*).

#### Authorship

Acknowledging those involved in reporting ensures accountability for accuracy and increases trust in report content. STARDIT reports must be completed by named people who are accountable for the data being reported. Ideally, a public persistent digital identifier (for example, an ORCID number) [110] or an institutional email address will be linked to authors’ names using Wikidata.

Reports cannot be completed anonymously, but STARDIT editors can redact author details from publicly accessible reports for ethical reasons (such as privacy or risks to safety).

Report authorship can be led by any stakeholder, including people associated with, or affected by, the initiative such as employees, researchers, participants, or members of the public. The affiliations of people formally associated with the initiative can be shared in a report.

#### Submission and editorial process

Reports can currently be submitted to STARDIT via a simple online form or emailed as a document file. At present, only data which is already publicly accessible can be included in a STARDIT report. It is a way of collaboratively structuring data, not a primary repository for data. Once a report is submitted, editors can review content for quality control (for example, checking that publicly accessible URLs and URIs align with the data in the report), but will not critically appraise the initiatives or methods. The Editorial process is currently parallel to the WikiJournal process, involving selected Editors from these journals. While Editors will not approve the ethics of the initiative, a transparent process for considering ethical issues will be considered before publishing a report. The Editors may consider questions such as, ‘Does data need to be redacted in order to prevent harm and protect or preserve life?’ or, ‘Is personal information being shared without consent?’ For more information about the Editorial process for reviewing data quality and ethical considerations, see the section ‘Editorial and peer review of STARDIT reports’ of the Additional file 1 ‘STARDIT Manual Beta Version’.

Once approved by the Editors, the STARDIT data will be entered into the database in a machine-readable format using structured data, based on the widely used Resource Description Framework (RDF) developed by the World Wide Web Consortium (W3C), which is used by Wikidata [111]. Each STARDIT report is assigned a unique Wikidata item number and all previous versions are navigable in a transparent history.

In future versions, it is proposed that stakeholders will be able to submit reports directly via an application programming interface (API), which will facilitate machine automation of STARDIT report creation. In addition, machine learning algorithms could be programmed to generate STARDIT reports from existing databases. As humans and machines submit reports, categories or meta-tags will be suggested (such as ‘patient’, ‘member of the public’), with the option of adding, or co-defining, new categories using the Wikidata system for structured data [112].

The database will generate a unique version number for the report with a Digital Object Identifier (DOI). To create an immutable version, the report will also be using the Internet Archive (a charity which allows archives of the World Wide Web to be created, searched and accessed for free) [113]. Finally, the report will be assigned a status, with the data quality checking being described as:

- manually added, no human review (low quality checking—no DOI assigned)
- machine added, no human review (low quality checking—no DOI assigned)
- human review (medium quality checking—DOI assigned pending Editorial decision)
- peer or expert reviewed, with publicly accessible sources for indicators and references checked (higher quality checking—DOI assigned pending peer or expert review).

Processes for data checking and assigning report status need to be further developed and agreed by the STARDIT working group. For example, developing a transparent process if a report has been created about an initiative with no involvement from anyone associated with the project, or only one subset of stakeholders. In such cases, the Editorial team might give a short period of time for any other stakeholders to be involved in checking and editing any information.

#### Updating reports

STARDIT will enable reports to be updated as initiatives progress over time. Updates will be reviewed by the STARDIT Editors. Once an update is approved, the system generates a new version number, while also preserving the original report. Updates might include, for example, information about involvement in the initiative, or about dissemination, translation, co-creation of new metrics to assess impacts, or longer-term outcomes [114].

A minimum dataset is required for a STARDIT report. This is called the Minimum Contribution Report (MICRO) and the required categories are highlighted in green and marked with an asterisk (*). Relevant Wikidata items and qualifiers for these fields are provided in the Additional file 1 in the section ‘Developing taxonomies and ontologies’ and on the Science for All STARDIT Beta webpage [109].

### Scope and applications

STARDIT is the first and only data-sharing system that enables standardised sharing of data about how people are involved in any type of initiative, across any discipline, including involvement in the planning, evaluation and reporting of initiatives. In addition it allows comparison of both evaluation methods and any impacts or outcomes in relation to standardised terminology. The next section summarises the current usage of STARDIT, while Table 1 summarises the proposed scope and potential further applications.

### Current usage

STARDIT provides a way to report data about who did which tasks in an initiative. STARDIT reports have already been used to describe a number of research projects, including data about who did which tasks, ethics approval, funding, methods and outcomes [41, 87, 88].

In health and medicine, STARDIT is already being used by an Australian Genomics working group to have describe planned work to improve guidance on involving the public in genomic research [108]. The Cochrane Council voted to use STARDIT to outline a proposed process for co-creating a Cochrane values statement [107, 115]. Other projects which have used STARDIT reports include participatory action research projects involving a large cohort study of > 15,000 healthy, elderly research participants [103], a protocol for precision medicine for Aboriginal Australians [104], and a group of patients and families affected by a rare immunological disorder [42], and a project involving extended family of donor-siblings who share the same sperm-donor father [41, 105].

The Wikipedia-integrated open access peer reviewed WikiJournals are also using STARDIT, which has articles which are integrated into Wikidpedia [116]. For example, a STARDIT report has been created to share information about a Wiki Journal of Medicine article about systematic reviews (with an associated integrated Wikipedia page) [116], including information about authors, editors and peer-reviewers [117]. This allows readers to critically appraise the source before deciding whether to use or share it.

**Fig. 3:**
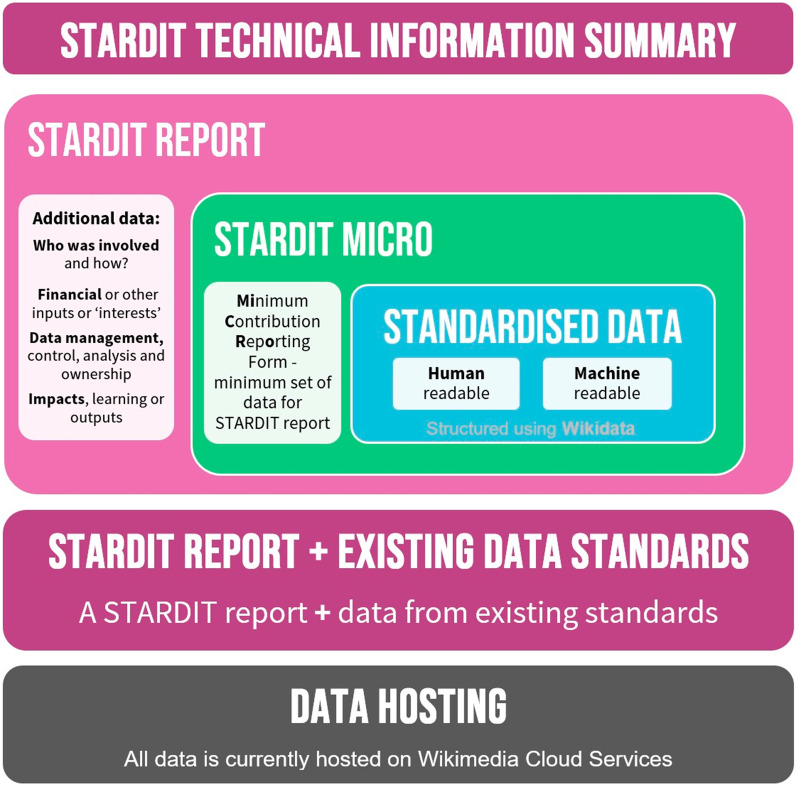
STARDIT technical information summary

An environmental research project has also used STARDIT to report the initiative, which works with citizen scientists to locate critically endangered species using eDNA [118, 119]. Currently, the Standardised Data, which makes up the STARDIT reports, is structured in WikiData, and hosted in the STARDIT report format using WikiSpore, which is hosted on Wikimedia Cloud Services, and is used as an experimental and supplementary space to develop potential Wikimedia projects [120]. Figure 3 summarises how Standardised Data is organised.

Further examples of how STARDIT can be used are provided in the Additional file 1, including; using STARDIT in genomic research for mapping phenotypes and reporting who was involved in helping define and describe them; providing data to critically appraise information sources (including public videos); report data about case studies consistently; create ‘living systematic reviews’ and train machine learning from STARDIT data.

#### Using STARDIT

**Fig. 4:**
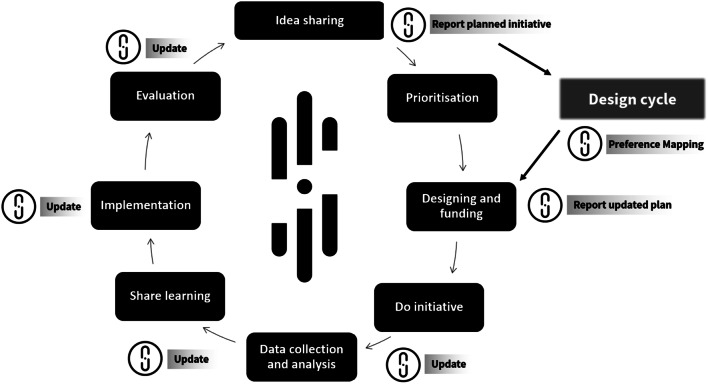
Planning and evaluating initiatives using STARDIT

Across all disciplines, ‘plan’, ‘do’ and ‘evaluate’ are recognised as distinct stages of initiatives [121]. While there are many ways to involve different people in these stages, standardised reporting and thus evidence-informed methods of doing so are lacking [7, 9, 122]. Figure 4 describes how STARDIT can be used to map how people might be involved in designing, doing, reporting and evaluating initiatives, starting with ‘idea sharing’ (Fig. 4).

##### Reporting initiative design in STARDIT

**Fig. 5:**
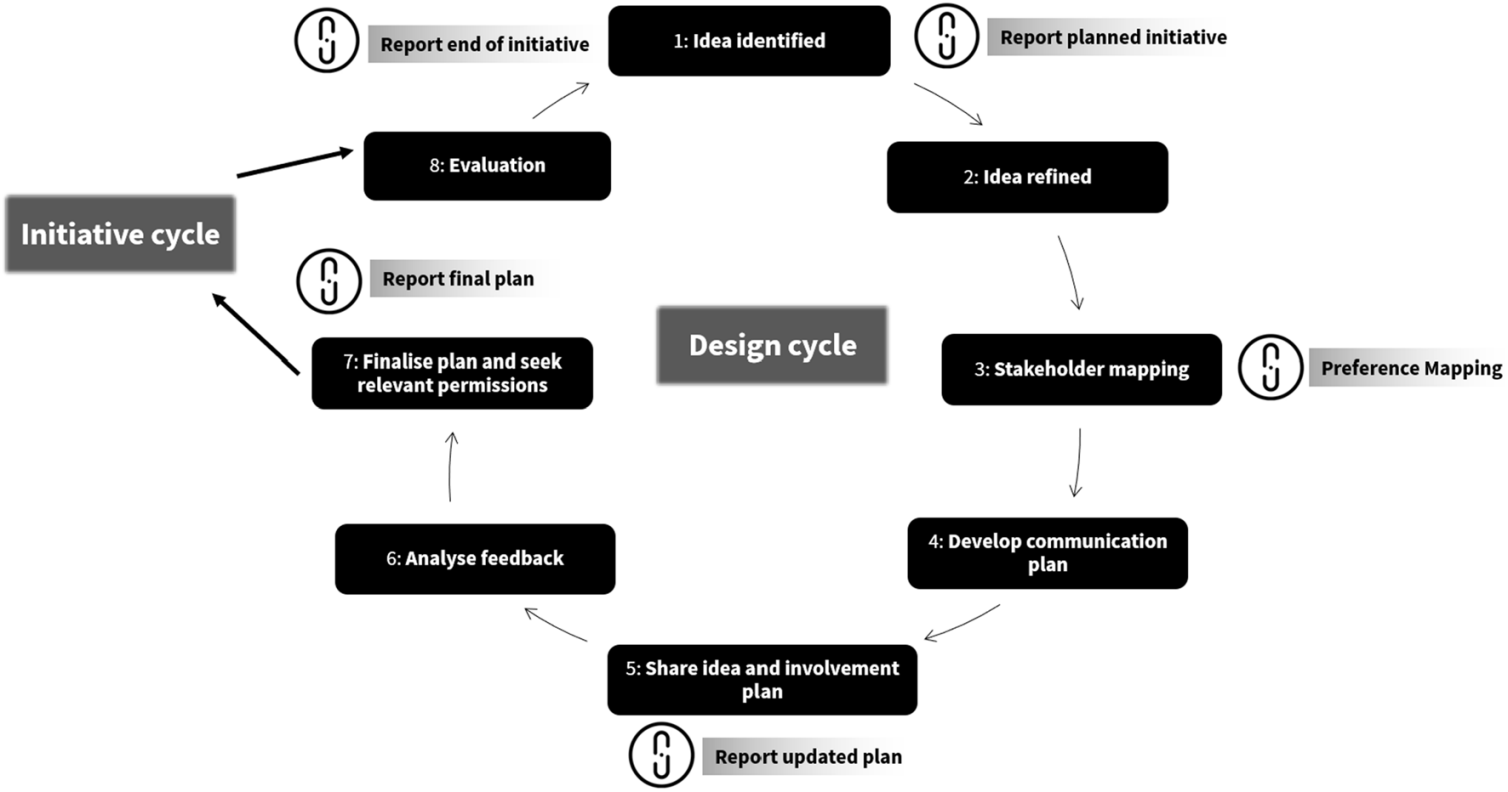
Reporting initiative design in STARDIT

Questions such as, ‘Who decides how people are involved?’ and, ‘Who is involving whom?’ and ‘what are people’s preferences for ways of working?’ can be difficult to answer and is an active area of research [42, 123]. For example, planning a healthcare initiative requires input from experts as well as from the people the initiative is intended to help [122]. Figure 5 summarises a way of using STARDIT to report the design process of initiatives, with Table 2 providing details about how involvement from different stakeholders can be reported at different stages. Table 2 also makes reference to the STARDIT Preference Mapping tool (STARDIT-PM). The section ‘Detailed reporting of design using STARDIT’ in the Additional file 1 ‘STARDIT Manual Beta Version’ provides more comprehensive information.

**Table 2:** Summary of reporting initiative design in STARDIT

Initiative stage	Data reported
**Stage 1: Idea identified:** An idea for an intervention, project or research is identified and articulated	Report planned initiative
**Stage 2: Idea refined** The idea is refined with a small group of stakeholders [7, 29, 124–129]	
**Stage 3: Stakeholder mapping:** Using the STARDIT-PM tool, existing stakeholders attempt to map who is included and who might currently be excluded from the process [29, 130]	Preference Mapping
**Stage 4: Co-create communication plan** Develop a communication plan to invite people to co-create involvement [29, 124, 131]	
**Stage 5: Share plan:** Share the idea (according to the communication plan) and ask for feedback on it (including the involvement plan) [129, 132, 133]	Report updated plan
**Stage 6: Analyse feedback:** Collect and analyse feedback, share results [131(p1)]	
**Stage 7: Finalise idea and involvement plan:** Co-create the plan (including the plan for involving people), seek relevant permissions and ethics [134, 135]	Report final plan
**Do initiative** (see ‘Planning and reporting initiatives using STARDIT’)	
**Stage 8: Evaluate involvement and outcomes:** Evaluate the process and the impact of both the initiative and involving people in the initiative	Report end of initiative

Bold text indicates the stage summarised in Fig. 5

##### Mapping preferences for involvement

Involving multiple stakeholders in designing how people should be involved in initiatives is considered best practice, as it may facilitate power sharing and improve the process overall [9, 136]. Current explanations of participatory research methods, and the language used to describe them, vary considerably. There is no agreed, consistent way to describe how people have been involved in an initiative, or to report the impacts of their involvement.

The STARDIT Preference Mapping (STARDIT-PM) tool provides a standardised way to report the preference of multiple stakeholders. Anyone can be involved in creating a STARDIT report, which means that data on the impacts and outcomes of participation can be contributed by diverse stakeholders. Such reports will help researchers make informed decisions when planning participation in research.

For example, a recent study showed how a charity for people affected by a rare disease involved a small number of people affected by the rare disease. They were involved in discussing preferences for how best to involve the wider community of people affected in future research prioritisation and planning [42]. Those involved had a good understanding of any specific needs or preferences for involvement, and shared preferences for the tasks (such as overseeing data access), method (facilitated discussions) and mode of involvement (online text-based discussion). The STARDIT-PM data about this processes showed a preference for being involved using online discussions, and the STARDIT report stated that involving people influenced the way the charity planned to involve people prioritising research in the future [87].

Examples of completed STARDIT-PM can be found in the additional files of a number of research projects [41, 87, 103]. Table 3 summarises questions which can be asked to map stakeholder preferences with respect to involvement in initiatives.

**Table 3:** Questions for mapping preferences for involvement

Question	Rationale for question
Which stakeholder group does this person align with?	To establish which grouping(s) the person identifies as being part of—for example ‘researcher’ or ‘participant’ (noting any groupings should be co-defined)
Describe any financial relationship or other interest this person has to this project	To provide a public record of any potential conflicting or competing financial interest
Views on the purpose and values of the research	To establish the purpose of the research, and the motivations and values of the initiative from multiple perspectives
Describe how you think the learning from this initiative could be used	To establish views about knowledge translation and application of learning
Views on which data from this project should be shared with which people and how	To establish that person’s view about data sharing and ownership
Views on who should be involved (which ‘groups’ of people)—including who should not be involved*—following answers may be categorised depending on the stakeholder group*	To establish that person’s views on which ‘groups’ of people they think should be ‘involved’ in research—that is, having a role in shaping the research design, direction and outcomes *Note: Answers may require sub-categories if there are multiple categories for who should be involved (see Fig. *4*)*
Views on specific tasks of this person or group	To establish that person’s views on the tasks of the specific stakeholders who they think should be involved
Preferred modes of communication	To establish that person’s preferences on communication modes with stakeholder groups
Views on what methods should be used	To establish that person’s views on which methods should be used to involve people—for example ‘online survey’
Views on facilitators of involvement	To explore that person’s perceptions of what might facilitate involving specified groups of people and help inform the design of involvement
Views on barriers of involvement	To explore that person’s perceptions of what might be a barrier to involving specified groups of people and help inform the design of involvement
Views on what the outcome or output of the involvement could be	To ascertain the expectations of that person about what involving the specified groups of people might achieve
Views on which stage of the research this group should be involved?	To establish that person’s views on which stage of the research the specified groups of people should be involved in

The first stage of preference mapping requires individuals to self-identify as belonging to a specific grouping of people. People from that grouping then share views on how people from other groupings could be involved (or which groupings should not be involved). For example, labels for such groupings could include:

- only people with a professional role in the initiative
- everyone (any member of the public who is interested)
- anyone who might be indirectly affected by the initiative
- only people who are directly affected by the initiative
- only people who are participating in the initiative
- only people with a financial interest in the initiative.

As a consistent mapping tool for use across all initiatives, STARDIT would allow both comparison of diverse stakeholder views and exploration of similarities and variations in relation to preferences for involvement. Used alongside other planning tools, this information could help align initiatives with stakeholders’ preferences. In this way, how stakeholders are involved throughout an initiative could be co-designed from the outset. Analysis of the data about preferences should involve stakeholders from multiple groupings to ensure that a diversity of perspectives are involved in assigning meaning to any data.

### Values

The STARDIT co-design process included co-defining shared values. It was agreed that the STARDIT project must be implemented in a way which encourages those involved to acknowledge cultural values and assumptions in a transparent way. For example, some people can be labelled as having human-centred (anthropocentric) values, which values natural resources in relation to benefits they can provide for humans. In contrast, some people who think the value of nature should be measured using non-human outcomes can be labelled ecocentric [137]. A participatory process requires mapping all of these perspectives and, where possible, labelling them.

The values for STARDIT were adapted from an existing values statement co-created by the charity Science for All [138], with values specific to the STARDIT project summarised in Table 4. Further information about the values are provided in the Additional file 1 ‘STARDIT Manual Beta Version’.

**Table 4:** Values of the STARDIT project

Value	Summary
System and language agnostic	STARDIT is system and language agnostic, it should always be designed to work across and with as many systems as possible, in as many countries and languages as possible
Designs and code should always be open access	In alignment with the UNESCO Recommendation on Open Science [43], STARDIT designs and code should always be open access and relevant licenses should always be those which allow others to build on and improve the project, while maintaining central control over quality (such as the Creative Commons Attribution-ShareAlike 4.0 International license (CC BY-SA 4.0) and the GNU General Public License (GPL) 3.0 for code)
Participatory paradigm	STARDIT development will be guided by the participatory action research (PAR) paradigm [66]. PAR is an umbrella term which describes a number of related approaches, including [85(p1)], community-based participatory research, participatory action research (including critical participatory action research), participatory health research, community-partnered participatory research, cooperative inquiry. It may also include other forms of action research embracing a participatory philosophy which may include ‘co-design’ of research and other kinds of research which might include forms of ‘public involvement’ (or sometimes ‘engagement’). The plain English definition of the paradigm is that power to control the project with be shared in a transparent, inclusive and equitable way.
United Nations rights-based paradigm	STARDIT will be guided by the United Nations rights-based paradigm, including human rights, environmental rights and other emerging rights

## Discussion and future versions

Since the inception of this project in 2019, subsequent world events have included; the worst bushfires in Australian history [139] in parallel with misinformation campaigns funded by industries whose actions increase the severity and frequency of such fires [140, 141]; the COVID-19 pandemic and associated "infodemic" of misinformation [142]; continued violence inspired by misinformation [143–145]; and "infowars" of information control which continue to take place alongside wars fought with physical weapons [146]. The need for tools which can provide a way for all global citizens (and their machines) to share, asses, verify, edit, and link data has never been greater or more urgent. STARDIT is one such tool, which, by using Wikidata, will make use of existing and trusted infrastructure, and allows people to co-define types of data in multiple languages [147–149].

STARDIT is the first tool that enables sharing of standardised data about initiatives across disciplines. It enables reporting of who was involved, any impacts of stakeholders’ involvement, and outcomes of initiatives over time. This functionality addresses a serious limitation of the current peer-reviewed publication process in which articles are not easily updated. However, there is no single process for making decisions that would improve and refine the processes, language and taxonomies associated with reporting initiatives, including who was involved in which tasks [150]. Similarly, based on feedback from Indigenous community leaders, patient representatives and others, it is essential to ensure access to learning and development opportunities is available to support people to both access and create STARDIT reports. The STARDIT project therefore needs to continually appraise the inclusiveness and effectiveness of its multidisciplinary, multilingual system, including accessibility of interfaces. To achieve this, the project will continue to work with its partner organisations, including the Wikimedia Foundation, a global leader in this field (Table 5).

**Table 5:** Summary of STARDIT Beta Version data fields

Section	Data category		Data field
Core: Initiative context—This information locates the initiative within a clear context	Identifying information		Initiative name*
			Geographic location(s)*
			Purpose of the initiative (aims, objectives, goals)*
			Organisations or other initiatives involved (list all if multi-centre)*
			Relevant publicly accessible URLs/URIs
			Other identifiers (e.g. RAiD [166], clinical trial ID [167, 168])
			Keywords or metatags—including relevant search headings (e.g. MeSH [169])
			Other relevant information (free text)
	Status of initiative		What is the current state of the initiative?* Select from: 1. Prospective—this report is prospective or describes planned activity 2. Ongoing—the initiative is still taking place 3. Completed—the initiative has finished (*evaluation and impact assessment may be ongoing*)
			Date range (start and end dates of initiative)
	Methods and paradigms		Methods of the initiative (what is planned to be done, or is being reported as done). Include information about any populations or eco-systems being studied, any ‘interventions’, comparators and outcome measures (qualitative or quantitative)* *If appropriate, include a link to a publicly accessible document (such as a research protocol or project plan)*
			Include any information about theoretical or conceptual models or relevant ‘values’ of people involved with this initiative, including any rationale for why certain methods were chosen
Report authorship—Information about who completed the report and how *Please note this section can be completed multiple times if there are multiple authors*	Identifying information for each author *(authors can be anonymised in the public report but at least one verified identity will need to be sent to STARDIT Editors to attempt to prevent falsified reports)*		Name*
			Publicly accessible profiles, institutional pages*
			Open Researcher and Contributor ID (orcid.org)*
			Tasks in report completion
			Other information
	Accountability		Key contact at initiative for confirming report content (include institutional email address)*
	Date		Date of report submission *(automatically generated)*
Input: Ethics assessment	Ethics approval information (if applicable)		Assessing organisation or group*
			Approval date and approval ID—*include any relevant URL*
Input: Human involvement in initiative Who is involved in this initiative and how? Editors assessing involvement may need to use the STARDIT ‘Indicators of involvement’ tool	Details about how each group or individual was involved in the initiative		Who was involved or how would you label those involved (select from group labels or submit new group label name in free-text)* *You can name individuals or use ‘labels’ to describe groups of people such as ‘professional researchers’, ‘service users’ or ‘research participants’*. *Additional ‘labels’ or ‘meta-tags’ to describe people may be added if appropriate*
			How many people were in each grouping label?
			Tasks of this person or group (list as many as possible)*—*including any information about why certain people were included or excluded in certain tasks (such as data analysis)*
			Method of doing task? How did these people complete these tasks? (what methods were used)—*for example ‘group discussion’ or ‘reviewing documents’*
			Communication modes? What modes of communication were used—*for example, ‘group video calls’, ‘telephone interviews’ or ‘postal survey’*
			How were people recruited, contacted or informed about these tasks?
	Involvement appraisal		Methods of appraising and analysing involvement (assessing rigour, deciding outcome measures, data collection and analysis)
			Enablers of involvement (what do you expect will help these people get involved—or what helped them get involved) *Examples of enablers*
			Barriers of involvement (what do you expect will inhibit these people from getting involved—or what inhibited them from getting involved). Are there any known equity issues which may contribute? *Examples of barriers, and any attempts to overcome them*
			How did the initiative change as a result of involving people? *For example, did the initiative design or evaluation plan change?* *Note: this can be answered separately for different individuals or groupings of people*
	Involvement outcomes, impacts or outputs		Were there any outcomes, impacts or outputs from people being involved?* *When describing these, attempt to label which groupings were affected and how. These can include impacts on people, organisations, processes or other kinds of impacts*
	Learning points from involving people		What worked well, what could have been improved? Was anything learned from the process of involving these people?
	Stage		Which stage of the initiative were these people involved? *(please provide information about any distinct stages of this initiative, noting some may overlap)*
	Financial or other interests (including personal or professional interests)		Describe any interests (financial or otherwise), conflicting or competing interests, or how anyone involved may be personally, financially or professionally affected by the outcome of the initiative* *Including any relevant information about authors of this report*
Input: Material involvement in initiative Mapping financial or other ‘interests’	Financial		What was the estimated financial cost for the initiative
			Funding information (link to publicly accessible URL if possible)—*this may include the project funder, funding agreements, grants, donations, public ledgers, transaction data or relevant block(s) in a blockchain*
	Time		How much time was spent on this project *Note: this can be answered separately for different individuals or groupings of people*
	Other		Describe any costs or resources that cannot be measured financially or quantitatively—*this may include expertise, traditional or Indigenous knowledge, volunteer time or donated resources*
Outputs: Data including code, hardware designs or other relevant information	Sensitive data	Secure criteria	Data adheres to relevant industry/discipline data security requirements
		Repository	How is data entered, changed or removed within a repository?
		Usage	Who is the data from this initiative shared with?
			Who has access to sensitive data and how is this decided?
		Safety	Is data encrypted? Is it anonymised or de-identified? What methods are used for re-identification? What is the risk of unauthorised re-identification?
	Open data	FAIR criteria	Data adheres to FAIR criteria [170]
		Findable	Describe relevant metadata, how the data is machine readable and other relevant information
		Accessible	How can data be accessed—*include any information about authentication and authorisation*
		Interoperable	How is data interoperable or integrated with other data? *Include information about applications or workflows for analysis, storage, and processing, and resulting file formats or other outputs*
		Reusable	How can data be replicated and/or combined?
	Indigenous data	CARE principles	Data adheres to CARE principles [171, 172]
		Collective Benefit	How will Indigenous Peoples derive benefit from the data
		Authority to Control	How will Indigenous Peoples and their governing bodies determine how relevant data are represented and identified
		Responsibility	How will those using the data provide evidence of these efforts and the benefits accruing to Indigenous Peoples
		Ethics	How have Indigenous Peoples’ rights and wellbeing been centred during the data life cycle
	All data	Hosting	Where is it data stored and hosted -s*hare any location data if appropriate*
		Owner	Who ‘owns’ the data or claims any kind of copyright, patent(s), or other specific types of intellectual property—*include relevant open licensing information*
		Analysis methods	Describe methods used to analyse the data (including a link to any relevant code and information about validity)
		Usage	How can data be used? *Include information about license and attribution*
		Dissemination	How is information about this data disseminated? *For example, how are results from analysis shared?*
		Impact	impact/effect of the output
		Data control	Who controls access to the data? How are decisions about data access made? Is data anonymised or de-identified? What methods are used for re-identification? What is the risk of unauthorised re-identification? How is this risk managed?
		Management and quality	Which person (or organisation) is responsible for managing (or ‘curating’) the data?
			Who is accountable for ensuring the quality and integrity of the data? (this may be an individual or organisation)
Impacts and outputs: Publications, events, changes, learning items etc	What was learned		What new knowledge has been generated? (if appropriate, include effect size, relevant statistics and level or evidence)*
	Knowledge translation		Describe how the learning or knowledge generated from this initiative has or will be used
	Impacts		Have there been any outcomes, or has anything changed or happened as a result of this initiative that isn’t captured in previous answers?*
	Measurement and evaluation		How has or how will this be measured or evaluated?
			Who is involved in measuring or evaluating this?
			Who was or is involved in deciding on the outcomes used to evaluate any impacts or outcomes? How were they involved?
Information completed by Editors			
STARDIT report version number (assigned)			Report number assigned to distinguish it from any future updated reports
Indicators completed by Editors and/or peer reviewers Editors and peer reviewers assessing the report will need to look for indicators in the following categories on publicly accessible URLs*	Indicators of involvement		Use the STARDIT ‘Indicators of involvement’ tool
	Indicators of data practice compliance		Use the relevant criteria
	Indicators of translation and impact		
	Other indicators		

The co-design process for STARDIT (hosted by the charity Science for All) ensured people from multiple organisations and countries were involved in both creating and refining STARDIT, ensuring it is usable and relevant in multiple disciplines. Consultation with experts, and source materials from around the world, have informed the design of STARDIT. Co-authors come from disciplines including health research and services, environmental research and management, economics, publishing with over 20 different institutions represented. Future versions should be informed by a regular, systematic search, review and appraisal processes, using the Preferred Reporting Items for Systematic Reviews and Meta-Analyses (PRISMA) data set [151], used for reporting in systematic reviews and meta-analyses.

While there are multiple methods for mapping values [152, 153], there is currently no agreed, standardised way to map the values (beliefs and personal ethics) of those involved in initiatives and those creating reports in STARDIT. Further research is needed to facilitate mapping of values and detect whether certain perspectives are being consciously or unconsciously excluded.

STARDIT seeks to be an easy-to-use way for people from multiple disciplines to share data about initiatives. However, amassing sufficient reports to create a useful database is estimated to take at least 5 years, and will likely require machine learning. For example, machine learning may be used in parallel with humans (for verifying data) to generate STARDIT reports from existing publicly accessible data at a scale and speed otherwise impossible for humans alone to achieve. In addition, both the potential and limits of machine learning should be transparently reviewed in relation to the field of adversarial machine learning [154]. Similarly, the process of creating ‘living systematic reviews’ from STARDIT reports is currently theoretical and would require significant development and rigorous testing to realise.

It is important to note that access to Wikidata is actively blocked by governments or internet service providers in some countries. While such censorship limits people’s ability to contribute or critically appraise data, STARDIT has been designed to be both interoperable with existing standards, and ‘future proofed’ by being system and language agnostic, to allow interoperability with existing and emerging data systems beyond Wikidata.

Science for All will continue to host the co-creation process and to monitor and evaluate the project. However, an open, transparent governance process that enables anyone to be involved in decision making and ongoing co-design of STARDIT will need to be established, and is proposed in more detail in the Additional file 1.

Ensuring that the STARDIT development process is inclusive and ethical, and that the database is quality assured, is paramount to ensuring that STARDIT is credible, useful and trustworthy. STARDIT currently relies on volunteers and pro-bono services from not-for-profit organisations. In the future, people should be paid for certain tasks, especially if the project is to avoid excluding the involvement of those from lower socio-economic backgrounds who may not be able to afford to volunteer their time. For the success and longevity of this project, a sustainable, transparently-decided funding model needs to be established, which ensures both the independence of the data, the hosting process and the governance.

## Conclusion

This article summarises work to date on developing **Sta**nda**r**dised **D**ata on **I**ni**t**iatives (STARDIT), an open access web-based data-sharing system for standardising the way that information about initiatives is reported across diverse fields and disciplines. It provides a way to collate and appraise data about how different people have been involved in different tasks of multiple types of initiatives. The current usage by multiple initiatives demonstrates to usability of STARDIT, and will inform the next stages of development. In accordance with the principles of transparent participatory action research, the authors invite the involvement of any interested persons in developing and improving the next version of STARDIT, Version 1.0. Detailed and up-to-date information about STARDIT is available on the Science for All website (ScienceforAll.World/STARDIT) [155].

## Supplementary Information

**Additional file 1.** This document contains additional information relevant to the article ‘Standardised Data on Initiatives(STARDIT) Beta Version’.

**Additional file 2.** This document contains a STARDIT Beta version report about the co-creation process of the STARDIT Beta version.

**Additional file 3.** This document contains a GRIPP report about the co-creation process of the STARDIT Beta version.

**Additional file 4.** This document describes how the public were invited to be involved in giving feedback on ‘Standardised Data on Initiatives (STARDIT)' between 2019 and 2021.

## Data Availability

Supplementary data can be found in the Additional files, with further data available in the associated STARDIT report for this article: [insert link].
