# Supplementary material for: Standardised data on initiatives—STARDIT: Beta version
Source: Res Involv Engagem. 2022 Jul 19;8:31. doi: 10.1186/s40900-022-00363-9 (PMC9294764; doi:10.1186/s40900-022-00363-9)
Supplement: Supplementary file 1 — Additional file 1. This document contains additional information relevant to the article ‘Standardised Data on Initiatives(STARDIT) Beta Version’. [file 40900_2022_363_MOESM1_ESM.pdf]

Supplementary File: STARDIT Beta

STARDIT

About this document

This document contains additional information relevant to the article ‘Standardised Data on Initiatives (STARDIT) Beta Version’ [https://doi.org/10.1186/s40900-022-00363-9].

Document Summary

STARDIT Development phases - contains a detailed description of the STARDIT co-creation process from Alpha onwards, including planned development towards Version 1.0.

Using Standardised Data on Initiatives (STARDIT): Beta Version Manual - contains information about how to use STARDIT, proposed governance of STARDIT and other technical detail. This is the first version of the STARDIT Manual and contains important information about values, governance, legal and technical information.

Additional research - summarises research conducted which informed STARDIT development. This includes an extensive table of models, frameworks and other resources which informed STARDIT. The search strategy for these resources is described in more detail in the scoping review conducted in 2020 as part of the development process (to be submitted for publication in 2022)[1].

Detailed reporting of design using STARDIT - provides detailed information about reporting initiatives using STARDIT.

Contents

About this document 1
Contents 2
STARDIT Development phases 3
Alpha version development 3
Beta version development 3
Version 1.0 development 4
Using Standardised Data on Initiatives (STARDIT): Beta Version Manual 5
About this manual 5
What is the purpose of STARDIT? 5
What is STARDIT? 5
Who is involved in STARDIT? 5
How is STARDIT being developed? 6
What are the values of STARDIT? 6
What is Standardised Data? 9
What is an 'initiative' 9
What is a STARDIT report? 10
Developing taxonomies and ontologies 11
Visual language and logo development 18
Using STARDIT 20
Detailed example applications 21
Editorial, peer and open review of STARDIT reports 23
How does STARDIT work within 'law' and 'lore'? 26
Recognising the limits of language, data and measures 27
Additional research 28
Models and papers which inform the standardised reporting framework 28
Figure 5: Table 6 Inclusion criteria 29
Table 6: Resources which informed the standardised reporting framework 30
Table 7: Reporting standards, guidelines or taxonomies discovered during scoping review 95
Table 8: Detailed summary of STARDIT solutions to current limitations in standardised reporting 116
Detailed reporting of design using STARDIT 119
Table 9: Detailed reporting of design using STARDIT 119
References 122

STARDIT Development phases

Alpha version development

The Alpha version was co-created with a small team of experts from a number of different disciplines, informed by their work with diverse communities around the world[2]. The initial Alpha version was informed by a number of frameworks and models (see supplementary materials 'Models which inform the standardised reporting framework'). A modified Delphi technique was used for some stages of the development, with this method to be reviewed to future versions[3]. Draft versions were shared with co-authors and final decisions were made using an online decision making system, hosted pro-bono by the not-for-profit organisation Science for All, working in partnership with the Wikimedia Journals. The team co-created a method to invite feedback and improvement from as many people as possible in the development of the beta version. The Alpha version was published as a pre-print, allowing anyone in the world to view it.

Beta version development

Once the Alpha version was published, people could provide feedback anonymously using online forms, in online discussion forums and at face to face and online discussions. A face to face event was held in London with participants from other countries joining online, including Australia and Belgium.

The Alpha version author team invited public feedback on the pre-print. A link to the paper and a feedback form was shared on a publicly accessible URL, on the Science for All website and via the authors' social media and email networks.

Decisions about the reporting framework were made using voting process on an online decision making system, hosted pro-bono by the not-for-profit organisation Science for All,[4] working in partnership with the Wikimedia Journals.

A pre-print version of the Beta was shared online and the public were again invited to give feedback through an online form, shared document, online discussion forum or via email. People who gave feedback were invited to be a co-author, contributor or named in the acknowledgements, in accordance with the guidelines of the Committee on Publication Ethics (COPE)[5], with transparent reporting of who did which tasks and the author order determined by the order in which people joined the STARDIT project. Once feedback has been collated, an updated version of the 'STARDIT Public Consultation Report' will be created and cited in the version of the STARDIT Beta version submitted for peer-review.

The co-creation process and public consultation is described in more detail in the public consultation reports from 2019 and 2021[6,7]. The 2021 report includes responses and actions taken as a result of the responses to the consultation[7].

A working Beta version submission form for STARDIT reports was created in February 2021. It should be noted that in the Beta version free text entry is converted to Wikidata manually by the Editorial team and then checked by report authors. In future versions it is hoped this process will be automated and also allow 'drop down a menus' and auto-suggest of Wikidata items.

Summary of changes from Alpha version to Beta version

A number of aspects of STARDIT have been altered from the Alpha version as a result of the co-design process. Below is a summary.

- Plain English summary and introduction rewritten in order to provide a clearer introduction

- Added categories to STARDIT reporting framework about data sharing and purpose of research in preference mapping to capture more nuanced views about preferences in power sharing
- Added categories to the STARDIT report in order to capture data about the purpose of research
- Built a working version of STARDIT reports and form for submitting them
- Multiple other changes, summarised in the public consultation report[7]

There are a number of acknowledged technical limitations to the Beta version owing to budget constraints which the authors hope to resolve in future versions. These include:

- Better articulation of initiative stages using standardised terminology (for example, planning stage, evaluation stage).
- Better articulation of the values of different stakeholders, including those involved in ethical decisions
- A better interface for report completion, informed by co-design and human centred design principles
- A 'tick box' compatibility option where report authors can choose to make STARDIT reports align with other standards. This is STARDIT +, for example, STARDIT + GBIF[8]
- Learning and development resources in multiple languages to empower more people to create reports

Version 1.0 development

Once STARDIT Beta (version 0.2) has been submitted for publication, work will begin on the next version, (version 1.0). Those involved with STARDIT development will disseminate information, gather feedback and recruit more people and organisations to participate as project partners and potentially funders. This stage is estimated to take between 2 to 3 years, at which point a working group will formally invite other appropriate partner organisations (such as the UN and WHO) to adopt the STARDIT framework. A Steering Group will be established to oversee and continually improve the STARDIT system. The working group and Steering Group will continue to be hosted by Science for All, although the Steering Group will be able to vote on a decision to change or involve other host organisations. STARDIT will require continued working with publishers, research funders and governments to encourage adoption of the reporting tool. Future co-creation processes should be decided collaboratively, informed by international best-practices[9].

Using Standardised Data on Initiatives (STARDIT): Beta Version Manual

About this manual

The purpose of this manual is to provide all the information required to understand and use Standardised Data on Initiatives (STARDIT). As STARDIT evolves and develops, so will this manual. This is the first version of this manual, which has been written as a supplementary resource for the STARDIT Beta version.

This manual was written by Jack Nunn (January 2021) with the assistance of Thomas Shafee and in-kind support from the charity Science for All.

What is the purpose of STARDIT?

Standardised Data on Initiatives was created to help everyone in the world find and understand information about collective human actions, which are referred to as 'initiatives'.

What is STARDIT?

STARDIT is an initiative that anyone in the world can get involved in, an idea owned and controlled by no one person, government or other kind of organisation. STARDIT is an idea about how data could be shared about initiatives, and an aspiration to make as much data as possible readable and understandable by both humans and machines.

The Alpha and Beta versions of STARDIT are suggestions for what kind of data could initially be standardised, how this data can be checked for quality, how this data can be shared and other suggestions about how the initiative should be governed transparently. The Beta version is a starting point, answering the questions 'why', 'what if' and 'how?' to the question 'how can humanity understand what it is doing?'.

Who is involved in STARDIT?

Anyone in the world can get involved in the STARDIT initiative. STARDIT is designed and run using a 'participatory action research' paradigm, which aims to involve all stakeholders (the public, experts and others with a 'stake' in the work) in every aspect of the initiative. In plain English, anyone can get involved and have a say in how it should be designed and run, with all decisions made transparently and stored in a publicly accessible way.

The participatory action research process is currently being hosted pro-bono by the charity Science for All. This means that Science for All hosts and supports the process for designing STARDIT, making collective decisions, and reporting this process. It is proposed the development of STARDIT will continue to hosted by the not-for-profit organisation 'Science for All', although co-designing a tender process may be necessary for future versions as the workload inevitably increases.

The STARDIT initiative is currently and using the Wikimedia Foundation's Wikidata project to host STARDIT data and reports, using structured, machine readable data.

STARDIT is also working with people from multiple partner organisations to help align the initiative with the ways of working of other organisations to make it as useful as possible. Other partners include experts from La Trobe University, Campbell South Asia, Health Research Authority (England), members of the Cochrane Advocacy Advisory Group, the Evidence for Policy and Practice Information and Co-ordinating Centre (EPPI-Centre), the Poche Centre for Indigenous Health (Sydney University), the UCL Centre for Co-production in Health Research and John Hopkins University.

The names of the authors and project supporters as of August 2021 can be found in the STARDIT Beta version.

Every person involved in STARDIT (and therefore any organisations they may represent) must agree to uphold the defined values and act within the code of conduct defined in this document.

How is STARDIT being developed?

STARDIT will continue to be transparent in how it is governed and how decisions are made, with the entire process open for anyone to be involved. Open online discussion forums and public events will inform decision making and future co-design processes. The data-sharing system will be designed to be both interoperable with existing standards, and future proofed to allow continuous improvement and data inter-operability.

What are the values of STARDIT?

As STARDIT is a new initiative, we have adopted the values of Science for All[10], the organisation which is hosting the participatory action research process.

Summary of values

1. We are not-for-profit – our motivation is not profit but working for all life on earth
2. We value the freedom to ask any question, using the scientific method wherever possible
3. We support the principles of democracy, the 'rule of law' and evidence-informed policy wherever we can
4. We are transparent and accountable
5. We include as many people as we can, as best we can in every aspect of our work
6. We know that science means knowledge, and knowledge takes many forms

STARDIT values

1. **We are not-for-profit – working for all life on earth**
   - We value outcomes that everyone is involved in creating – including ones which could be measured in biodiversity, happiness indexes and often – are currently immeasurable.
   - We recognise the need to be inclusive, and therefore, to pay people for their time, skills and expertise. When we work in economic structures, we do so only to support our values, not to make profit at the expense of others.
   - We do not seek to impose our values (or other values such as economic outcome measures) on any life on earth.
   - We value human rights, but also note that 'rights' don't start and end with humans – we seek to explore what 'working for all life on earth' means in any way possible.
2. **We value the freedom to ask any question, using the scientific method wherever possible**
   - Asking a question can be the most simple, most profound or the most complex thing we can do as a species. Questions like 'why?' and 'why not?' are powerful.

- The freedom to ask the questions is essential, and we hope to support people everywhere to ask any question they feel is important.
- Some questions can be answered using the 'scientific method' – which essentially seeks to build our knowledge by asking questions, making observations about the available reality and using this to make predictions. If the predictions can be tested – or are repeatable – others can verify results. We value this method of asking questions, 'peer review', sharing open data and results – new knowledge, for free, for everyone to share, reuse, and build upon..
- We recognise the limits of the scientific method and that some things can never be 'known' or 'peer reviewed'.

3. **We support the principles of democracy, the 'rule of law' and evidence-informed policy wherever we can**
    - We support the United Nation's statement on democracy, including that 'democracy is a universal value' and that the principles of 'democracy, development and respect for all human rights and fundamental freedoms are interdependent and mutually reinforcing'[1].
    - We also accept that there is no single model of democracy and that democracy does not belong to any country or region. To uphold human rights and for 'the maintenance of peace', we agree that people require 'media freedom' in order to 'seek, receive and impart information'[11]
    - We support decisions and policies which are informed by evidence, preferably created from open data, shared freely and peer-reviewed by those who are collectively defined as experts. We do not support decisions or policy which appears to be informed by real or perceived conflicting or competing interests. We do not align with any political 'parties' or ideologies, and will only work with elected representatives to help uphold the values articulated in this document.
    - We recognise 'law' and 'lore' as forms of knowledge which must evolve and interact with reality and language. We support universal access to this kind of knowledge. We believe the purpose and enforcement of these laws must be collectively decided in the interests of all life on earth. We support the principle of the rule of law, which implies that every person is subject to the law, including people who are lawmakers, law enforcement officials, elected representatives and judges. However, we recognise that that are often multiple, sometimes competing or conflicting systems of 'law' and 'lore', and concepts of 'property'. We will always be guided transparently and collectively when navigating any conflicts or competing interests.
4. **We are transparent and accountable**
    - We support transparent decision making, reporting and evaluation.
    - We aspire to be as transparent as possible. We will always publicly disclose any funding or 'in kind' donations. Partnership with organisations will always align with these values.
    - We recognise that real or perceived conflicting or competing interests can damage trust, and we will do everything we can to avoid any doubt in regards to the motives of our actions being anything other than aligned with our values.
    - We accept that some things may have to be confidential (for example, storing confidential personal information to align with privacy laws) and we will always work transparently, inviting everyone to be involved in helping us get the balance right between privacy and transparency as the world evolves with new technologies.

---

[1] http://www.un.org/en/ga/search/view_doc.asp?symbol=A/RES/64/155

- We expect and encourage others to hold us to account, to make sure we do what we say we do, and act as we say we do. Transparency is the best tool to enable this.
- We will always try to understand the effect or impact of our actions – and work openly with anyone who can help us improve how we are measuring this. This will help ensure we are doing things the best way we can.

5. **We include everyone we can as best we can in every aspect of our work**
   - 'Organisations' are people working together towards a shared goal or purpose – and everyone is welcome to get involved.
   - We will be transparent about who is involved, how we are supporting people to stay involved, how we are working to involve new people and will always welcome ideas about how we can improve this.
   - Ensuring we are inclusive and do not knowingly 'exclude' anyone is central to our way of working
6. **We know that science means knowledge, and knowledge takes many forms**
   - We recognise that knowledge takes many forms – this includes people who are subject area experts, people with personal experience, people with traditional, indigenous or local knowledge, artists – and those with big dreams and big ideas
   - Linguistic labels like 'knowledge', 'skills', 'expert', 'artist', 'patient' and 'citizen' can be both helpful and unhelpful. Wherever possible we will include people in helping us define what we mean when we use these words.
   - Some things are knowable, some things are not – we work to explore the limits of what knowledge means – with everyone, for all life on earth.

Additional values and paradigms

In addition to Science for All's values, the following values are specific to the STARDIT initiative:

- STARDIT is **system and language agnostic**, it should always be designed to work across and with as many systems as possible, in as many countries and languages as possible

- STARDIT **designs and code should always be open access** and relevant licenses should always be those which allow others to build on and improve the project, while maintaining central control over quality (such as the Creative Commons Attribution-ShareAlike 4.0 International license (CC BY-SA 4.0) and the GNU General Public License (GPL) 3.0 for code)

- STARDIT **development will be guided by the participatory action research (PAR) paradigm**[12]. PAR is an umbrella term which describes a number of related approaches, including [13], community-based participatory research, participatory action research (including critical participatory action research), participatory health research, community-partnered participatory research, cooperative inquiry. It may also include other forms of action research embracing a participatory philosophy which may include 'co-design' of research and other kinds of research which might include forms of 'public involvement' (or sometimes 'engagement').

- STARDIT will be guided by the **United Nations rights-based paradigm,** including human rights, environmental rights and other emerging rights.

Immutable values

While these values will evolve, we will keep an immutable record of our values. They will always be shared via a publicly accessible URL and regularly archived on the 'Internet Archive' for future reference[14].

Our values will be reviewed annually – with a process for involving the public, with final approval going to a vote in the Steering Committee. This review process may change over time but will always require and invite specialist, expert and public scrutiny, according to the transparent governance principles described in the manual.

STARDIT must be implemented in a way which encourages those involved to acknowledge cultural values and assumptions in a transparent way. For example, some people can be labelled as having human-centred (anthropocentric) values, which values natural resources in relation to benefits they can provide for humans. In contrast, some people who think the value of nature should be measured using non-human outcomes can be labelled ecocentric[15]. A participatory process requires mapping all of these perspectives and, where possible, labelling them.

What is Standardised Data?

People and organisations share data in different ways around the world, in multiple languages. While ways of standardising data have been around for millennia, many ways are still specific to certain areas of knowledge or geographical regions and are often not inter-operable, or at best, hard to access or interpret.

For humanity to survive, and to live in balance within the eco-systems which sustain all life on earth, we need to understand what has already happened, what humanity is currently doing, who is doing it, why, and what the effects are. Answering these questions is very complex, and sometimes impossible.

By standardising ways of sharing data bout human action, we can allow both humans and machines to start linking data, start asking new kinds of questions, and empower anyone in the world to be able to answer them. Machine readable data will allow data to be interpreted in new ways.

What is an 'initiative'

We use the word 'initiative' to describe any intervention, research or planned project. Examples include:

- **Research project** – any organised activity undertaken to answer a question. This includes data collection and analysis to help answer future questions which may arise. Projects may be nested within one another – the report context should clarify this.
- **Project or initiative** - any organised activity with planned outputs or outcomes.
- **Educational intervention** – any organised activity designed to improve people's knowledge, understanding or skills[16,17]
- **Evaluation** – an attempt to evaluate an action or phenomena, including related impacts and outcomes. This includes program or policy evaluation

What is a STARDIT report?

A STARDIT report contains standardised data, collected into a 'report' which contains a minimum set of data required to describe a collective human action (an 'initiative') for a STARDIT report at a certain point in time. The minimum amount of data required for a STARDIT report is called a '**Mi**nimum **C**ontribution **R**ep**o**rt' (STARDIT MICRO), with the ability to add additional information to this minimum dataset.

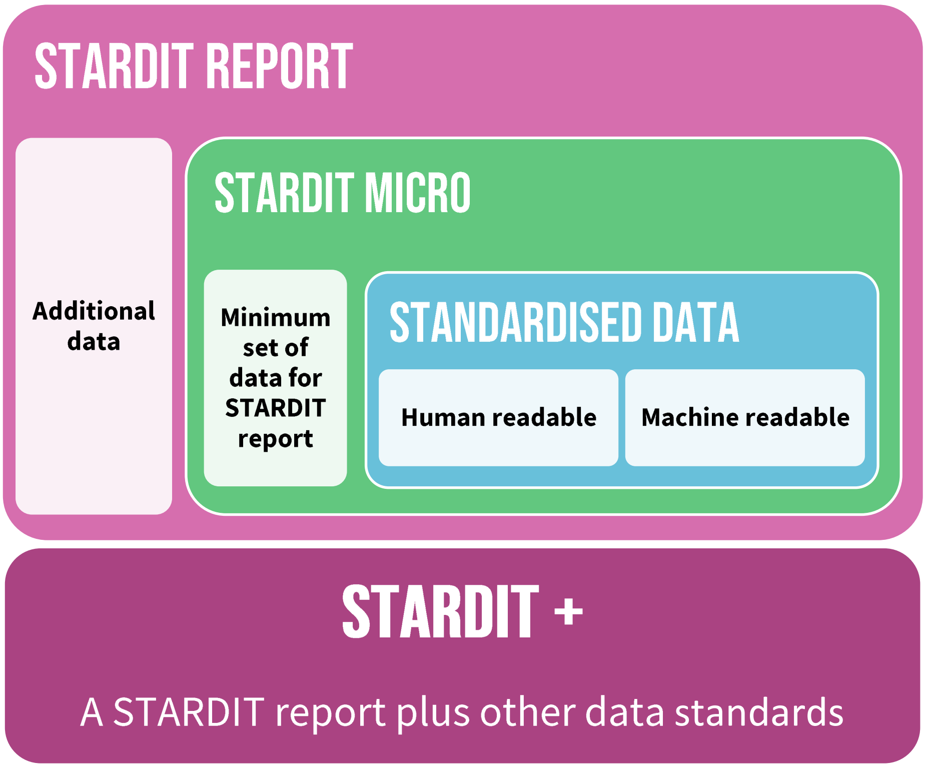

STARDIT reports can be created at any point of an initiative, including before, during and after. While they must describe an initiative at a fixed point in time, the reports can be updated to created updated versions of the same report, by updating or adding data.

Exemplar STARDIT report 1: A Pathway to Precision Medicine for Aboriginal Australians: A Study Protocol

A STARDIT report was created to prospectively describe how different people would be involved in the co-creation of a genomics precision medicine, working with a remote Aboriginal community.

The peer-reviewed article can be found here: https://doi.org/10.3390/mps4020042

The associated STARDIT Beta version report (human readable) is here: https://wikispore.wmflabs.org/wiki/A_Pathway_to_Precision_Medicine_for_Aboriginal_Australians:_A_Study_Protocol

Exemplar STARDIT report 2: Co-designing genomics research with donor-conceived siblings

A STARDIT report was created to describe how participatory action research methods were used to involve members of a sibling group in online discussions about how they would like to be involved in future research. The research process was co-designed using a participatory action research method to involve members of a sibling group in the co-design of online discussions to explore future genomic research with members of the group.

The STARDIT report describes who was involved in co-creating and checking the report, as well as reporting impacts and outcomes from the research.

The peer-reviewed article can be found here: https://doi.org/10.1186/s40900-021-00325-7

The associated STARDIT Beta version report (human and machine readable) is here: https://wikispore.wmflabs.org/wiki/Co-designing_genomics_research_with_donor-conceived_siblings

The Wikidata item for the STARDIT Beta version report is here: https://www.wikidata.org/wiki/Q108618394

Developing taxonomies and ontologies

Developing taxonomies and ontologies will be an ongoing process facilitated by the current Wikidata infrastructure, and may require creating additional tools to create more inclusive ways of involving people in developing taxonomies.[18] By using the Wikidata initiative, those involved can work to group concepts and code them, aligned with existing taxonomies.[19] Examples of existing taxonomies include the National Library of Medicine's Medical Subject Headings[20]. Terms with clear and easily defined descriptions will be assigned a code. This infrastructure may also aid machine learning, analysis by neural networks and other forms of artificial intelligence (AI), similar to the 'Cochrane Crowd' project[21].

The current STARDIT data fields are mapped below to existing Wikidata items, with a link to the most up to date version found here: https://wikispore.wmflabs.org/wiki/STARDIT/form_mapping

Section	Data category	STARDIT field	Wikidata encoding	MICRO Compulsory
**Core: Initiative context**	Identifying information	**Initiative name**	**Len**	**Compulsory**
		**Geographic location or scope**	**P937**	**Compulsory**
		**Purpose of the initiative**<br><br>'stated as' free text	**P3712**<br>P3712 Q P6001	**Compulsory**
		**Start date of initiative**	**P580**	**Compulsory**
		End date of initiative	P582	
		**Organisations or other initiatives involved**	**P664**	**Compulsory**
		Ethics approval (org)	P793 Q98550700 P1027	
		Ethics approval (date)	P793 Q98550700 P585	
		Ethics approval (ID)	P793 Q98550700 P1932	
		**Funding sources (org)**	**P8324**	**Compulsory**
		Funding sources (dept or scheme or grant ID)	P793 Q P1932	
		Relevant URLs	P856	
		keywords, metatags,	P921	

Section	Data category	STARDIT field	Wikidata encoding	MICRO Compulsory
		mesh terms, raid terms		
	Stage or state of initiative	**Date of report**	**P793 Q37260 P518 Q10870555**<br>**P793 Q37260 P585 or**<br>**P793 Q37260 P580 and**<br>**P793 Q37260 P582**	**Compulsory**
		**Methods of the initiative (what is planned to be done, or is being reported as done)**	**P4510**	**Compulsory**
		Link to a publicly accessible methodology document	P4510 Q P973	
		theoretical or conceptual models or relevant 'values' of people	P4510	
**Input: Report authorship & status**	How was this report generated?	**Name of report author (person or algoritm)**	**P50**	**Compulsory**
		ORCID	author item P496	
		Publicly accessible profile / institutional page	author item P856	
	Accountability	**Key contact email at initiative for**	**P793 Q37260 P968**	**Compulsory**

Section	Data category	STARDIT field	Wikidata encoding	MICRO Compulsory
		**confirming report content**		
	Report data review status	Who has checked the quality of the data in this report?	P4032	
**Input: Involvement in initiative**	Involvement details for each contributor	**Who was involved (named individual, organisation)**<br>**Who was involved (group of anonymous individuals acting in role)**	**P767**<br>**P767 Q P1114**	**Compulsory**
		**Specific tasks of this person or group**	**P767 Q P2868**	**Compulsory**
		Methods of involvement of participants	P767 Q P2283	
	Outcomes and outputs	**What was the outcome or output of the involvement?**	**P1542**	**Compulsory**
		**Were any publication produced as part of this?**	**P921**	**Compulsory**
	Involvement appraisal	Methods of appraising and analysing involvement (assessing rigour, deciding outcome measures, data		

Section	Data category	STARDIT field	Wikidata encoding	MICRO Compulsory
		collection and analysis)		
		Facilitators of involvement (what helps the contributors in achieving the project's outcomes?)<br><br>'stated as' free text	P1552 Q101097118 P5102<br><br>P1552 Q101097118 P6001	
		Barriers of involvement (what inhibits the contributors from achieving the project's outcomes?)<br><br>'stated as' free text	P1552 Q16515105 P5102<br><br>P1552 Q16515105 P6001	
		What was the outcome or output of the involvement of these people? What changed as a result of involving people? Were there any impacts?	P767 Q P1542	
	Stage	Which stage of the initiative were these people involved?	P767 Q P585 or<br><br>P767 Q P580 and<br><br>P767 Q P582	
	Cost and time	What was the estimated financial cost for involving each person or group	P767 Q P2130<br><br>P767 Q P2047<br><br>P767 Q P1542	

Section	Data category	STARDIT field	Wikidata encoding	MICRO Compulsory
		How much time did it take to involve each person or group<br><br>Were there any other non-financial costs in involving each person or group		
**Input: Financial or other interests**	Financial or other interests	**Financial relationship or other interest this person has to this project**	P767 **Q** P1932<br><br>**P1542 Q99429881 P6001**	**Compulsory**
		**Conflicting or competing interests**<br><br>'stated as' free text	**P1552 Q99429881 P1932**<br><br>P1552 Q99429881 P6001	**Compulsory**
		What was the estimated financial cost for the overall initiative.<br><br>How much time did it take.	P2130<br><br>P2047	
**Output: Research data**	FAIR data produced	Findable: How is information about this data disseminated	**P1056 Q42848 P1552 Q100451967**<br><br>**P1056 Q42848 P7228**	**Compulsory**
		Accessible: How is it stored and hosted	**P1056 Q42848 P4945**	**Compulsory**
		Interoperable: What analyses were	P4510	

Section	Data category	STARDIT field	Wikidata encoding	MICRO Compulsory
		Interoperable: What format is it in	P1056 Q42848 P2701	
		Reusable: Access restriction status	**P1056 Q42848 P7228 Q66739888**	**Compulsory**
		Reusable: License	**P1056 Q42848 P275**	
	Sensitive data produced	Who owns it	**P1056 Q42848 P1552 Q2587068**<br>**P1056 Q42848 P127**	**Compulsory**
		Where is it stored	**P1056 Q42848 P276**	**Compulsory**
		Access restriction status	**P1056 Q42848 P7228**	**Compulsory**
		How to access (email)<br>How to access (url)	P1056 Q42848 P968<br>P1056 Q42848 P2699	
		Data steward/curator	P1056 Q42848 P1640	
**Output: Impacts and outcomes**	Outcomes	Has anything changed or happened as a result of this initiative that isn't captured in previous answers?	**P1542**	**Compulsory**
	What was learned	What new knowledge has been generated? (if appropriate, include effect size, relevant statistics and	**P1542 Q133500**	**Compulsory**

Section	Data category	STARDIT field	Wikidata encoding	MICRO Compulsory
		level or evidence)		
		What part of the initiative was the learning about<br>What topic was learned	P1542 Q133500 P518<br>P1542 Q133500 P921	
	Knowledge translation	Describe how the learning or knowledge generated from this initiative has or will be used	P1542 Q133500 P1542	
	Measurement	How has or how will this be measured?	P1542 Q P459	
		Who is involved in measuring this?	P1542 Q P767	

Visual language and logo development

A culturally neutral logo was required for STARDIT in order for it to be recognised, including one which does not contain Latin alphabet letters so it could work across other scripts. Working with a professional graphic designer, the logos were co-designed, with anyone invited to give feedback and rank their favourite options. Inspiration for the logo was drawn from multiple traditions, with both tally sticks and DNA codons being integrated into the design of the logo, both systems of knowledge transfer which require a complementary half, representing the checking or tallying of the STARDIT data.

The novel and pronounceable word 'STARDIT' was purposely created, and it is proposed that the name for STARDIT in other languages be a phonetic way to spell the purposely invented word, with standardized spelling or signs to follow future co-design processes.

Future versions of the logo could contain Quick Response (QR) codes around the logo which link to report DOIs, to allow people to scan them easily on portable internet connected devices with cameras. Figure 4 is a mock up of such a QR code, linking to the first ever STARDIT report. Additional information about the data quality of each report could also be embedded in the logo by with

colours which can be discriminated by those who have a decreased ability to see colour or differences in colour.

For legal reasons, and to prevent uncontrolled use of the STARDIT logos and artworks, the ‘STARDIT’ name and other associated ‘intellectual property’ (such as copyright and trademarks), all of the aforementioned is owned by the charity Science for All. Discussions about ownership, and any transfer of such ownership to another organisation or persons will be made transparently, according the governance processes outlined in this document. The project welcomes advice and feedback on this.

Figure 2: Full logo with Latin Alphabet name

Figure 3: Icons

Small icon (0.5 CM – 2 CM)

Micro Icon (>0.5 CM)

Figure 4: Icon with Quick Response codes

Using STARDIT

Creating a report – Beta version

A stable link to the Beta version can be found at: https://ScienceForAll.World/STARDIT/Beta/

A direct link to the working Beta version of the reporting framework can be used at this address:

https://wikispore.wmflabs.org/wiki/STARDIT

This page also includes examples of human- and machine-readable filled in forms, and a link to the data mapping used to convert between these.

A short video with instructions for completing a STARDIT report to describe data from the Global Biodiversity Information Facility (GBIF) can be found here (please note that the Beta version interface has been updated since the creation of this video): https://commons.wikimedia.org/wiki/File:STARDIT_GBIF_video_V2.webm

Detailed example applications

Example 1: Mapping phenotypes

STARDIT has an additional potential application in reporting who was involved in defining phenotypes, which is relevant to genomics, other 'omics' and other areas of research including mental health[22,23]. Transparent reporting of who was involved and how in defining and describing 'qualia' (subjective lived-experience) will be crucial in ensuring that phenotype data is high-quality. For example, while there may be Wikidata codes for different parts of the spectrum, colour deficiency in an example of subjective lived-experience based on genetic variation that can affect many aspects of life. While this is a relatively easy to understand example, there are countless variations that mean we all will experience the world differently, including variations which may affect mental health. STARDIT can describe who was involved in describing lived experiences of phenotypes and any specific variations they may have. If such data is standardised across human languages and cultures (as much as is possible), this will facilitate such research, including machine learning.

Example 2: Providing data to critically appraise information

Organisations like Wikipedia provide an invaluable, impartial and free service for sharing information and works in partnership with multiple organisations including Google, Cochrane and many others. Trust in information underpins human civilisation. Ensuring that people can easily verify both who has created content, how, who funded it and who has reported information about this will allow both manual and automated checking of information sources. Google already uses Wikipedia to provide information about the authorship of YouTube videos and other information[24], and STARDIT could provide a much more detailed dimension to such reporting, which can be both viewed or edited by anyone.

Example 3: Reporting case studies

The STARDIT data-sharing system has been designed to align with case study methodology. It allows for multiple authors with different ways of analysing and interpreting data.[25,26] Multiple perspectives may aid understanding of any causal relationships by comparing (triangulating) data from different people, enhancing the reliability of the analysis[26.] STARDIT provides a way of consistently sharing and comparing data while using the case study method, and aligns with existing case study reporting tools.[27]

Example 4: Living systematic reviews

To improve evidence-informed ways of involving people in research, and to understand what works, for whom, why, and in what circumstances, several reviews have called for standardised ways of reporting involvement over extended periods of time.[23,28,29] 'Living systematic reviews',[30] which can be continually updated, could potentially be generated from STARDIT reports, and would contribute to an evidence base informing best practice and future policy. Future versions of STARDIT could:

1. form a team to scope the feasibility of creating a 'living systematic review' based on data from STARDIT reports
2. work in partnership with relevant stakeholders to ensure the process for creating living systematic reviews is transparent and conforms to best-practice agreeing on data for inclusion.

3. aid machine learning, analysis by neural networks and other forms of artificial intelligence (AI), similar to the 'Cochrane Crowd' project[21].

Many other applications have been proposed, including aligning STARDIT with the Global Biodiversity Information Facility (GBIF). More detail on STARDIT + GBIF is summarised on the Science for All website[8].

Editorial, peer and open review of STARDIT reports

Presently, the process for publishing reports relies on the Editorial team at the WikiJournals. In the future, the process will be designed to include an open public and peer-review process where appropriate (noting that public review does not involve being reviewed by experts or peers, but may involve checking the quality of data sources and critically appraising data). The process described in this section will be constantly reviewed and transparently decided. Experts from multiple relevant disciplines including ethics, law and data security will be involved, with decision making processes hosted at publicly accessible URLs. In future versions, machine learning algorithms could also be used to assess data completeness, data sources and other relevant metadata.

Validity and quality assurance of data

Processes for data quality assessment and assigning report status need to be further developed and agreed by the STARDIT working group, in order to decide when reports will be sent to humans for approval (authors or Editorial team), when it is necessary to confirm comprehension of an ethics statement, and what the public access will be to unchecked data. Existing systems have already implemented similar systems to quality assure 'research grade' data, and a high-quality process for deciding this will be central to the success of STARDIT Version 1.0.[31]

In future versions it will be necessary to further develop a transparent process if a report has been created about an initiative with no involvement from anyone associated with the project, or only one subset of stakeholders. In such cases, the Editorial team might give a standard period of time for any other stakeholders to be involved in checking and editing any information (similar to the concept of 'right of reply') before the report is published, or given the status 'human reviewed'. However, the process for deciding which stakeholders to contact and how to ensure equity (alongside capacity considerations of the Editorial team) is an area for active discussion and development in future versions. For example, ensuring Indigenous peoples who are stakeholders in a mining initiative have been involved in checking a STARDIT report about a mining initiative created by a company with a declared financial interest in the mining initiative might present challenges that it is not possible for the Editorial team to overcome. Such challenges could be labelled and incorporated as future data categories in the STARDIT reports, for example labels such as 'report not checked by all stakeholders' could be updated if more stakeholders have subsequently been involved in checking any report.

Consideration also needs to be given to creating data labels which describe if reports contain essential, emergency or 'life and death' information. For example, information about medicine safety, real-time natural disaster responses, or information about edible plants or fungi[7]. Such data labels might facilitate more efficient information sorting, and help ensure that high-quality, valid and relevant information can be shared with the right people, in the right place, at the right time.

A transparent process would need to be decided for agreeing 'trusted' sources, 'trusted' partner organisations. This might also involve developing an open-source algorithm for automating some critical appraisal decisions. A transparent and continually evolving process for agreeing when it is appropriate to restrict editing access from 'anyone' to 'trusted individual', 'trusted organisation' or 'trusted algorithm' (or 'bot') will also be essential for some data, in particular data which is 'life or death', or may be targeted by people making a deliberate attempt to create misinformation or to falsify data.

What is being proposed here is not simple, and will require extensive (and thus expensive) co-design, development and testing. However, solutions to these problems are urgent and essential, and the STARDIT project is well-positioned to offer some solutions, for the benefit of all life on earth.

Data ownership and hosting

o reduce data sharing barriers and encourage reuse, a STARDIT report cannot currently contain any proprietary information or information that is not open access or publicly accessible, except for information volunteered by the report authors (such as institutional email addresses), much like a 'corresponding author' on a peer reviewed paper. The report is therefore citing information already hosted in a publicly accessible way. STARDIT allows authors to structure publicly accessible data in a standard format, and the STARDIT data system will allow this to be read in a machine-readable format. The role of Editors (and in later versions of STARDIT, public and expert or peer-reviewers) is to ensure that the publicly accessible data sources cited in the report align with the report content. Where possible, content of URLs will be automatically archived using independent not-for-profit archiving services such as the Internet Archive[14] in order to prevent links expiring and to preserve the URL data at the time it was reviewed by the editors.

'Indicators of involvement' report review tool

For editors or peer-reviewers reviewing the STARDIT report, it may be necessary to look for objective publicly accessible indicators, in order to verify reports in categories such as involvement and research data. This section provides a proposed method to transparently review and label involvement.

Background to involvement indicators

Concepts such as 'involvement', 'ethics' and 'democracy' cannot always be expressed fully in linguistic constructs, as they carry resonances of concepts such as equality, justice and human rights [32]. A number of systematic and scoping reviews have attempted to map such concepts[23,33–35].

These indicators are an attempt to reduce subjectivity in describing 'involvement' by articulating in a standardised way what objective measures might exist which would suggest that 'involvement' (power sharing) is occurring, and for what purpose[36,37]. The indicators can be grouped and categorised in the table below and yet all of them rely on a foundational assumption or axiom – which is that those affected by interventions or research are the people most appropriate to design and deliver it – recognising all skills and knowledge have equal value [37]. The 'UK Standards for Public Involvement in Research' define indicators of involvement as 'a statement of good practice that describes what is needed to demonstrate meeting the standard'[38]. STARDIT aligns with this terminology, extending 'indicators' beyond public statements in order to incorporate other indicators, for example, governance documents or reported actions. Indicators may be either qualitative or quantitative data[39].

Defining the purpose of involvement

There may be intrinsic or extrinsic reasons for involving people [40]. For example, the purpose or 'driver' of involving people might be to include personal experiences in order to improve the research [41], to abide by regulation or law, or to do it because there is evidence which indicates involving people will improve the research [42]. A narrative review identified three broad value systems associated with involving people [43], which included values based on:

- ethical, human rights, equality, openness and accountability
- evidence based, objective assessments of effectiveness and consequences of involvement

For the purposes of this reporting framework, these have been categorised below as four broad ‘purposes’ of involvement.

Diagram of the purposes of involvement and measurable indicators

Purpose of involvement	Measurable indicators
**Obliged:** There are legal or other codified power constructs in place which oblige involvement in initiatives and research	• Public statements about methods or paradigms of involvement<br>• Public policy<br>• Support for people to get involved<br>• Reported actions<br>• Evidence of funding for involvement
**Post-positivist:** lived (subjective) experience and knowledge is valued equally alongside other kinds of knowledge and data	**Data sources can include:** Peer-reviewed papers, governance documents, legal or government documents, legislation or policy documents, publicly accessible websites
**Evidence-informed:** There is evidence which suggests that involving people improves the quality and direction of initiatives and research	
**Self-evident**: The concepts of ethics, human rights and democracy demand involvement	

Foundational principle

Those affected by initiatives are the people most appropriate to help design, deliver and evaluate it

Ethical review

In exceptional cases, the report may cite publicly accessible information that could cause harm to life (such as a report on poaching, corruption or other illegal activities) which may threaten the safety of the report authors if the information shared is undesirable to those in positions of power, or to those who may represent a real threat to the personal safety of authors or other lifeforms implicated in or impacted by a report. Authors are asked to self-asses their personal safety and that of others if disclosing personal information and are given the option of requesting to redact any identifying information from the report if they prefer. This request will be flagged on the report to indicate identity has been verified. The editorial team is not responsible for assessing this themselves (as all information is already publicly accessible), however they are required to flag with the report authors if they feel any personal or identifying information is being shared without consent.

Questions which editors should consider when reviewing a report include:

- Has data been sufficiently redacted in order to prevent harm and protect or preserve life?
- Is personal information being shared without consent?

It is not the responsibility of editors to ensure that publicly accessible content aligns with the values of other organisations and governments, as a central purpose of STARDIT is to create quality assured, peer-reviewed data immune from censorship (which is distinct from ethical redaction) and immune from future editing.

While STARDIT reports are immutable, if it is subsequently discovered that some information should be redacted for ethical reasons which were not apparent at the time of the report being published, this can be done so with an updated report, which will reflect which data field was redacted, by which author and for what reason. In extreme cases reports can be deleted, if approved by the Editorial team using a transparent decision-making process. Technical details on how this will be achieved will need to be constantly reviewed to balance ethical data hosting with database quality. If STARDIT reports have been archived on other databases (such as archiving sites) it is the responsibility of respective data-hosts to comply with any requests for redaction or removal.

How does STARDIT work within 'law' and 'lore'?

As stated by our values, "*we recognise 'law' and 'lore' as forms of knowledge which must evolve and interact with reality and language*". In addition, "*we recognise that there are often multiple, sometimes competing or conflicting systems of 'law' and 'lore', and concepts of 'property'. We will always be guided transparently and collectively when navigating any conflicts or competing interests*".

At present, there is no universally accepted authority to enforce 'international law' upon sovereign states or individuals. Accordingly, STARDIT conduct will be guided by the United Nations on all matters of law, not any one sovereign state. This has special relevance for Indigenous peoples globally who may not either be represented by or recognise the sovereignty of some nation states[44].

Relevant statements from the United Nations which underpin STARDIT include Article 19 of the Universal Declaration of Human Rights, which states[11]:

'*Everyone has the right to freedom of opinion and expression; this right includes freedom to hold opinions without interference and to seek, receive and impart information and ideas through any media and regardless of frontiers'*

In 2020, the United Nations Secretary-General stated '*purposes that involve data and analytics permeate virtually all aspects of our work in development, peace and security, humanitarian, and human rights'*, encouraging '*everyone, everywhere'* to '*nurture data as a strategic asset for insight, impact and integrity – to better deliver on our mandates for people and planet'*[45].

For the purposes of concepts of "intellectual property" (such as copyright and trademarks), and to protect STARDIT from being used by people in ways which are outside of the values defined in this document, any intellectual property (including logos or code associated with STARDIT) are currently held by the charity Science for All, which is currently hosting the participatory action research process to create and manage STARDIT. Any decisions relating to the above (including transfer of ownership of any intellectual property) are to be made by the STARDIT Steering Committee, which hosted by Science for All, but independent of Science for All. In the future ownership may be transferred to an appropriate organisation established specifically for the purpose of owning such

intellectual property, for example, establishing a charity called 'Standardised Data International – STARDIT).

STARDIT will always defer to United Nations declarations in favour of any sovereign laws, and does not recognise itself as a legal entity which is acting in any one sovereign state. Citizens of non-UN-recognised states will be recognised as individual legal persons and included equally alongside any other person, regardless of status (including citizens, residents, asylum seekers and refugees). STARDIT is an initiative to support individuals to self-organise ways of sharing information, and it is the responsibility of each individual (including individuals working on behalf of organisations) to act both within the values of STARDIT, and any laws to which they may be subject.

Recognising the limits of language, data and measures

STARDIT is not intended to be a way of explaining or expressing every aspect of reality or subjective existence in words and data, but a way of collectively mapping and then standardising language and data we are currently using as a species.

> "I realized that the difference that I saw between things was the same thing as their unity, because differences (borders, lines, surfaces, boundaries) don't really divide things from each other at all, they join them together, because all boundaries are held in common"[46]
>
> Alan Watts

The principle behind Goodhart's Law is important in the future development of STARDIT to ensure that the data collected, and how it is collectively structured does not have unintended consequences [47]. Goodhart's Law can be summarised as "when a measure becomes a target, it ceases to be a good measure".[48]

While STARDIT may provide the basis for reporting data and facilitating future machine learning, the data itself should be organised and structured collectively and transparently. Where possible is should facilitate critical appraisal, rather than the data collection itself become a target or measure. STARDIT should however encourage data reporting to improve the accessibility of data which can inform such critical appraisal. Any 'scoring' or 'rating' of data beyond the quality of available data would be in addition to anything already described and would need careful consideration and co-design.

Standardised data categories to describe the limits of data itself will be essential for future versions. In future versions of STARDIT, the limits of meta-data should be further articulated, and the concept of 'anti-meta data' should also be explored. The concept of 'anti-meta data' proposed here for the first time, is data which describes what something is not, or describes what is not described by the data. Distinct from this is ineffable-meta data, or data about something which inherently indescribable. Such developments in types of meta data will allow a more accurate mapping (and critical appraisal) of what is known, what is knowable but not unknown, what is knowable but indescribable, and what is both unknowable and indescribable.

Additional research

Models and papers which inform the standardised reporting framework

STARDIT has been informed by multiple models and reporting frameworks across many disciplines, and will be compatible with many of them and future-proofed to allow further development in the articulation of reporting.

Further information about the search strategy will be available with the forthcoming scoping review article to be submitted in 2022 [1]. Defining the inclusion and exclusion criteria was an iterative process informed by published scoping review methodologies. Any information source which included information about how an initiative was planned, reported or evaluated was initially included as 'guidance', before being screened for relevance. Initiatives were included if they described an action, intervention, project or other kind of participatory process.

Database search

As the search question incorporates multiple disciplines, the search strategy incorporated results from a number of databases representing multiple disciplines. We used the EBSCOHost Research Database to search CINAHL Complete, ERIC and Business Source Complete. We used the OVID database to search MEDLINE and APA PsycINFO.

Consulting experts and citation searching

As part of a related public consultation process[28], experts were invited to provide relevant citations from their discipline. The author team then appraised the information sources (according to the inclusion and exclusion criteria), and searched the citations of included information sources. Citation searching went to one level of depth, that is searching the citations of primary information sources for citations, but not searching the citations of the secondary information source, owing to the limited capacity of the author team. The results from this process can be found in Table X in the results section.

We also conducted a systematic site search, which is described in more detail in the forthcoming article.

Figure 5 summarises the inclusion criteria for the preliminary results in Table 6.

Table 6 summarises the frameworks and models which informed this work and how.

The search strategy is summarised in the figure 'STARDIT models and framework search strategy'.

In order to inform future iterations, the authors suggest that future versions of STARDIT are informed by regular systematic search, review and appraisal, with the protocol published in PRISMA.

Figure 5: Table 6 Inclusion criteria

Inclusion criteria	Description
**Planning initiatives**	We included sources which described ways of planning initiatives, including frameworks for reporting planning.
**Reporting initiatives or methodology**	We included sources which described ways of reporting methodology, including research methods, frameworks for reporting international development, assessing educational interventions and reporting environmental interventions.
**Methods of evaluating initiatives**	We included sources which described ways of evaluating initiatives, including reporting impact and outcomes.
**Reporting involvement**	Sources which described involvement or any ways of reporting it involvement were included. We developed a criteria to define ‘involvement’ based on the International Association for Public Participation’s participation spectrum and other studies. This included reports of ‘consultation’, ‘involvement’, ‘collaboration’ and ‘empowerment’. Involving people in initiatives was defined as the ‘active involvement’ in shaping and guiding research, rather than only providing data.

Sources which had unspecific or unclear prose descriptions were excluded. The search only included English language documents, with non-English studies excluded

Table 6: Resources which informed the standardised reporting framework

This table contains a summary of sources which have informed STARDIT. Each source has a reference with more information provided in the references. This is a preliminary version of this table (and has not been peer-reviewed) and is shared to provide context to the research conducted in parallel with the development of STARDIT. A more comprehensive version of this table will be available in the forthcoming scoping review 'Guidance for planning, reporting and evaluating initiatives: A multidisciplinary scoping review'[11]. The table is organised alphabetically, first by 'field' and then 'name'.

Name	Status	Access	Year	Field	Reporting standard, guideline or taxonomy	Aspects which informed STARDIT
A Standard Taxonomy for Peer Review[383]	Peer-reviewed	Publicly accessible website	2021	Academic integrity		Discovered after Alpha version development, informed Beta version
The Hong Kong Principles for assessing researchers: Fostering research integrity[371]	Peer-reviewed	Open access	2020	Academic integrity	Hong Kong Principles (HKG)	Discovered after Alpha version development, informed Beta version
Shortell[49]	Peer-reviewed	Paywall access	2015	Accountable care organisations		Informed terminology to describe involving people in assessing priorities and outcomes of care, including in accountable care organisations
Aircraft Accident and Incident Investigation , International Civil Aviation Organization[50]	Not peer-reviewed	Paywall access	2021	Aircraft incident investigation	International Standards and Recommended Practices for aircraft accident and incident	

This document contains additional information relevant to the article 'Standardised Data on Initiatives (STARDIT) Beta Version' https://doi.org/10.1186/s40900-022-00363-9

Name	Status	Access	Year	Field	Reporting standard, guideline or taxonomy	Aspects which informed STARDIT
					Investigation	
EBCD: Experience-based co-design toolkit[374]	Not peer-reviewed	Publicly accessible website	2022	Allied Health	EBCD	Discovered after Alpha version development, informed Beta version
Victorian Agency for Health Information[51]	Not peer-reviewed	Publicly accessible website	2020	Analysis and sharing of data (quality and safety of healthcare)		Informed ways of visualising data about the quality and safety of health systems
Planetary Health Alliance[349]	Not peer-reviewed	Publicly accessible website	2022	Anthropogenic disruption to natural ecosystems		Discovered after Alpha version development, informed Beta version
Paratiro.gr[363]	Not peer-reviewed	Publicly accessible website	2022	Anthropogenic impacts on wildlife	PARATIRO	Discovered after Alpha version development, informed Beta version
Global Anti Corruption Consortium [302]	Not peer-reviewed	Publicly Accessible Website	2022	Anti-corruption		Discovered after Alpha version development, informed Beta version
Dillon[52]	Peer-reviewed	Open access	2017	Assessing patient involvement in health research		Informed general terminology, including outcome measures for involvement
European Geosciences Union[320]	Peer-reviewed	Open access	2021	Authorship conflict prevention		Discovered after Alpha version development, informed Beta version

Name	Status	Access	Year	Field	Reporting standard, guideline or taxonomy	Aspects which informed STARDIT
Bank Track[298]	Not peer reviewed	Publicly Accessible Website	2003	Bank Finance Activities	Evaluates the social, environmental and economic consequences of major banks financial activities	Discovered after Alpha version development, informed Beta version
Maccarthy[53]	Peer-reviewed	Open access	2019	Basic and preclinical health research	PPI Ready: Researcher Planning Canvas	Informed general terminology, including impact and values
Global Biodiversity Information Facility (GBIF)	Not peer-reviewed	Publicly accessible website	2020	Biodiversity (data sharing)		Informed terminology to describe international data sharing methodologies
Access to Biological Collection Data (ABCD), Taxonomic Databases Working group (TDWG)[54]	Not peer-reviewed	Publicly accessible website	2020	Biodiversity (Data standardisation)	Access to Biological Collection Data (Taxonomic Databases Working group)	Informed terminology to describe methods of standardised reporting of biodiversity
Biodiversity Information Standards, Taxonomic Databases Working group (TDWG)[55]	Not peer-reviewed	Publicly accessible website	2020	Biodiversity (Data standardisation)	Biodiversity Information Standards, Taxonomic Databases Working group (TDWG)	Informed terminology to describe methods of standardised reporting of biodiversity
Darwin Core, Darwin Core Task	Not peer-reviewed	Publicly	2020	Biodiversity (Data	Darwin Core, Darwin	Informed terminology to

Name	Status	Access	Year	Field	Reporting standard, guideline or taxonomy	Aspects which informed STARDIT
Group, Taxonomic Databases Working group (TDWG)[56]		accessible website		standardisation)	Core Task Group, Taxonomic Databases Working group (TDWG)	describe methods of standardised reporting of biodiversity
Ecological Metadata Language, National Center for Ecological Analysis and Synthesis[57]	Not peer-reviewed	Publicly accessible website	2020	Biodiversity (Data standardisation)	Ecological Metadata Language (National Center for Ecological Analysis and Synthesis)	Informed terminology to describe methods of standardised reporting of biodiversity, including metadata languages
World Geographical Scheme for Recording Plant Distributions, Taxonomic Databases Working group (TDWG)[58]	Not peer-reviewed	Publicly accessible website	2020	Biodiversity (Data standardisation)	World Geographical Scheme for Recording Plant Distributions, (Taxonomic Databases Working group)	Informed terminology to describe methods of standardised reporting of biodiversity in plants
Encyclopedia of Life[299]	Peer-reviewed	Publicly Accessible Website	2014	Biodiversity (multidisciplinary)	Taxonomic classification of various Eukaryotes, Prokaryotes and acellular organisms	Discovered after Alpha version development, informed Beta version
Selinske[59]	Peer-reviewed	Paywall access	2019	Biological Conservation		Informed terminology to describe the social

Name	Status	Access	Year	Field	Reporting standard, guideline or taxonomy	Aspects which informed STARDIT
						dimensions and outcomes of land management, including describing how people can have different 'functions' in their environment
Whitburn[60]	Peer-reviewed	Open access	2019	Biological Conservation		Informed terminology for describing and comparing behaviours as an outcome measure (for example 'pro-environmental behaviour') and synthesising data. Informed terminology for self-reports and 'construct validity'
CRediT taxonomy[61]	Not peer-reviewed	Publicly accessible website	2017	Biology	Contributor Roles (CRediT) taxonomy	Informed taxonomy to define author contributions to primary research papers
Medical Subject Headings (MeSH), United States National Library of Medicine[62]	Controlled and hierarchically-organized vocabulary	Open access	2020	Biomedical and health information	Medical Subject Headings (MeSH)	Informed ways of structuring vocabulary and other terminology (including qualifiers) in a hierarchy, in a standardised way (including interoperability and API usage).
Shippee[63]	Peer-reviewed	Open access	2015	Biomedical and health research		Informed general terminology, in particular

Name	Status	Access	Year	Field	Reporting standard, guideline or taxonomy	Aspects which informed STARDIT
						terminology to articulate the stages of research
Shippee [63]	Peer-reviewed	Open access	2013	Biomedical and health services research		General terminology, description of research stages, informed search strategies
Mark2Cure[64]	Not peer-reviewed	Publicly accessible website	2020	Biomedical research		Informed terminology to describe a method of mapping key terms and concepts to identify connections
Virtual Communities for Impact[65]	Not peer-reviewed	Publicly accessible website	2020	Building virtual communities		Informed terminology for co-creating communities, informed terminology and methodology for evaluating impact
B Impact Assessment[66]	Not peer-reviewed	Publicly accessible website	2020	Business		Informed ways of reporting impact for organisations and initiatives
Deverka[67]	Peer-reviewed	Paywall access	2012	Cancer Genomics	Stakeholder participation in comparative effectiveness research: defining a framework for effective	Informed general terminology and terminology specific to genomics research, as well as methods of assessing impact of involving stakeholders.

Name	Status	Access	Year	Field	Reporting standard, guideline or taxonomy	Aspects which informed STARDIT
					engagement	
Embodied Carbon in Construction Calculator (EC3) tool	Not peer-reviewed	Publicly accessible website	2019	Carbon emissions in manufacturing	Embodied Carbon in Construction Calculator (EC3) tool	Informed terminology to describe reporting of carbon usage
Climate Active, Australian Government[68]	Not peer-reviewed	Public disclosure summary	2019	Carbon Neutral certification	Climate Active Standard Carbon Neutral Program	Informed terminology to describe standardised reporting of carbon usage
NISO[316]	Peer-reviewed	Publicly Accessible Website	2022	Categorization of scholarly content	JATS4R	Discovered after Alpha version development, informed Beta version
Australian Charities and Not-For Profit Commision[333]	Not peer-reviewed	Publicly accessible website	2022	Charity		Discovered after Alpha version development, informed Beta version
Charity Evaluation Working Group[69]	Not peer-reviewed	Publicly accessible website	2020	Charity evaluation (multidisciplinary)		Informed terminology for describing evaluating the work of charities and using evidence informed decision making
Support Guidelines (Evaluation Support Scotland)[70]	Not peer-reviewed	Publicly accessible website	2020	Charity evaluation (multidisciplinary)	Support Guidelines (Evaluation Support Scotland)	Informed terminology to describe reporting initiatives, outcomes and other areas of initiatives.
Code of Fundraising Practice, Fundraising Regulator (UK)[71]	Not peer-reviewed	Publicly accessible	2021	Charity financial reporting		Informed terminology to describe fundraising

Name	Status	Access	Year	Field	Reporting standard, guideline or taxonomy	Aspects which informed STARDIT
		website				methodology
Australian Charities and Non-for-profit Commission, Australian Government[72]	Not peer-reviewed	Publicly accessible website	2021	Charity reporting		
Charity Navigator Methodology [73]	Not peer-reviewed	Publicly accessible website	2001	Charity research		Informed terminology for recording and comparing data about charities in a consistent way, in particular methods for rating accountability and transparency
CitizenScienc.cz[364]	Not peer-reviewed	Publicly accessible website	2021	Citizen		Discovered after Alpha version development, informed Beta version
Participedia[317]	Not peer-reviewed	Publicly Accessible Website	2022	Citizen engagement design		Discovered after Alpha version development, informed Beta version
10 Principles of Citizen Science, Australian Citizen Science Association[74]	Not peer-reviewed	Publicly accessible website	2018	Citizen Science		
Australian Citizen Science Association[351]	Not peer-reviewed	Publicly accessible website	2022	Citizen science	ACSA'S PROJECT FINDER	Discovered after Alpha version development, informed Beta version
Australian Citizen Science Project Finder, Australian Citizen	Not peer-reviewed	Publicly accessible	2021	Citizen Science		Informed terminology to describe database of citizen

Name	Status	Access	Year	Field	Reporting standard, guideline or taxonomy	Aspects which informed STARDIT
Science Association[75]		website				science projects
Citizen Science COST Action[76]	Not peer-reviewed	Publicly accessible website	2019	Citizen science	Citizen-Science Ontology, Standards and Data	Informed terminology to describe metadata-concepts, vocabulary and an ontology to support data sharing about initiatives. Also informed terminology to describe data ownership.
Citizenlab[358]	Not peer-reviewed	Publicly accessible website	2022	Citizen Science		Discovered after Alpha version development, informed Beta version
CoActD7.1: Impact Assessment Plan.[324]	Peer-reviewed	Open access	2020	Citizen science		Discovered after Alpha version development, informed Beta version
Co-Creating and Implementing Quality Criteria for Citizen Science[384]	Peer-reviewed	Open access	2020	Citizen science		Discovered after Alpha version development, informed Beta version
Core Values, Ethics, Spectrum – The 3 Pillars of Public Participation[369]	Not peer-reviewed	Publicly accessible website	2022	Citizen science		Discovered after Alpha version development, informed Beta version
CS Track[331]	Not peer-reviewed	Publicly accessible website	2022	Citizen Science		Discovered after Alpha version development, informed Beta version

Name	Status	Access	Year	Field	Reporting standard, guideline or taxonomy	Aspects which informed STARDIT
EU-Citizen.Science[77]	Not peer-reviewed	Publicly accessible website	2020	Citizen science		Informed terminology to describe citizen science
Everyone counts? Design considerations in online citizen science [373]	Peer-reviewed	Open access	2019	Citizen science		Discovered after Alpha version development, informed Beta version
Göbel[78]	Not peer-reviewed (pre-print)	Open access	2019	Citizen science		Informed terminology to describe citizen science and open science
Göbel[79]	Peer-reviewed	Open access	2019	Citizen science		Informed terminology of governance and purpose of citizen science
Medborgarforskning.se[362]	Not peer-reviewed	Publicly accessible website	2020	Citizen science	ARCS	Discovered after Alpha version development, informed Beta version
Österreich forscht[361]	Not peer-reviewed	Publicly accessible website	2022	Citizen science		Discovered after Alpha version development, informed Beta version
scistarter[350]	Not peer-reviewed	Publicly accessible website	2022	Citizen science		Discovered after Alpha version development, informed Beta version
SciStarter[80]	Not peer-reviewed	Publicly accessible	2020	Citizen Science		Informed categorisation of citizen science projects

Name	Status	Access	Year	Field	Reporting standard, guideline or taxonomy	Aspects which informed STARDIT
		website				
Soleri[81]	Peer-reviewed	Open access	2016	Citizen science		Informed terminology to describe citizen science and ‘power’ dynamics
Strasser[82]	Peer-reviewed	Open access	2019	Citizen science		Informed terminology, typology and political considerations of ‘citizen science’ and participatory research
Ten Principles of Citizen Science, European Citizen Science Association (ECSA)[83]	Not peer-reviewed	Publicly accessible website	2015	Citizen Science	Ten Principles of Citizen Science (European Citizen Science Association)	Informed terminology to describe citizen science
The Observatory of Citizen Science in Spain[359]	Not peer-reviewed	Publicly accessible website	2022	Citizen Science		Discovered after Alpha version development, informed Beta version
Understanding Participation: The ‘citizen science’ of genetics[376]	Peer-reviewed	Open access	2014	Citizen science		Discovered after Alpha version development, informed Beta version
Weobserve[352]	Not peer-reviewed	Publicly accessible website	2022	Citizen science		Discovered after Alpha version development, informed Beta version
Citscibio[84]	Not peer-reviewed	Publicly accessible	2020	Citizen science		Informed terminology to describe citizen science in

Name	Status	Access	Year	Field	Reporting standard, guideline or taxonomy	Aspects which informed STARDIT
		website		(biomedical)		biomedical research
Borda[85]	Peer reviewed	Open access	2019	Citizen science (health and biomedical)		Informed terminology to describe citizen science in biomedical and health research
QuestaGame Core Values[86]	Not peer-reviewed	Open access	2020	Citizen Science (values)		Informed terminology to describe concepts such as 'values' and the 'purpose' of data collection
Opportunities and Risks for Citizen Science in the Age of Artificial Intelligence[334]	Peer reviewed	Open access	2019	Citizen science and AI		Discovered after Alpha version development, informed Beta version
Data and metadata working CSA[353]	Peer-reviewed	Publicly accessible website	2022	Citizen science metadata		Discovered after Alpha version development, informed Beta version
PPSR Core[354]	Not peer-reviewed	Publicly accessible website	2022	Citizen Science research Standards		Discovered after Alpha version development, informed Beta version
Pacific iCLIM[381]	Peer-reviewed	Publicly accessible website	2019	Climate information	iCLIM	Discovered after Alpha version development, informed Beta version
Sacristán[87]	Peer-reviewed	Open access	2016	Clinical research		Informed general terminology
Bloom[88]	Peer-reviewed	Open access	2018	Clinical Trials		Informed language to

Name	Status	Access	Year	Field	Reporting standard, guideline or taxonomy	Aspects which informed STARDIT
						describe partnerships between different stakeholder groups (industry, academia, patients and public)
Loudon[89]	Peer-reviewed	Open access	2015	Clinical trials	PRECIS-2 tool	Informed terminology to describe clinical trials
Toolkit for Researchers and Research Organisations, Australian Clinical Trials Alliance[90]	Not peer-reviewed	Publicly accessible website	2019	Clinical trials		Informed terminology for evaluating and reporting involvement
Searchable FCC ID Database, Federal Communications Commission[91]	Not peer-reviewed	Publicly accessible website	2020	Communications standards		
McIlduff[92]	Peer-reviewed	Open access	2019	Community Based Participatory Research	Model of Engaging Communities Collaboratively	Informed language to describe reporting of community based participatory research methods, including concepts such as 'community engagement' and 'implementation' (specifically in reference to Indigenous populations)

Name	Status	Access	Year	Field	Reporting standard, guideline or taxonomy	Aspects which informed STARDIT
The NGER Scheme[313]	Not peer-reviewed	Publicly Accessible Website	2022	Company GHG emissions respective of energy production and consumption		Discovered after Alpha version development, informed Beta version
Jane Goodall Institute Australia	Not peer-reviewed	Publicly accessible website	2018	Conservation		Informed terminology to describe reporting of conservation projects
Impact Evaluation - Case Study: Bwindi Impenetrable National Park and Mgahinga Gorilla National Park Conservation Project, Global Environment Facility[93]	Not peer-reviewed	Publicly accessible website	2007	Conservation biology, eco-tourism		Informed terminology for reporting theories of change and other outcomes and impacts
Australian Frozen Zoo[387]	Peer-reviewed	Publicly accessible website	2022	Conservation genetics		Discovered after Alpha version development, informed Beta version
The Frozen Ark[386]	Peer-reviewed	Publicly accessible website	2022	Conservation genetics		Discovered after Alpha version development, informed Beta version
CISAC[328]	Not peer-reviewed	Publicly accessible website	2022	Creative networking		Discovered after Alpha version development, informed Beta version
Resource Description Framework (RDF), World Wide	not peer-reviewed	Publicly accessible	2014	Data	World Wide Web Consortium (WC3)	Informed structured data usage

Name	Status	Access	Year	Field	Reporting standard, guideline or taxonomy	Aspects which informed STARDIT
Web Consortium (WC3)[94]		website			standards	
A New Way to Look at the Data: Similarities Between Groups of People Are Large and Important [315]	Peer-reviewed	Publicly Accessible Website	2019	Data analysis		Discovered after Alpha version development, informed Beta version
Initiative for Open Citations[95]	Not peer-reviewed	Publicly accessible website	2020	Data interoperability (citations)		Informed terminology to describe citations data sharing
ckan[309]	Not peer-reviewed	Publicly Accessible Website	2022	Data management system		Discovered after Alpha version development, informed Beta version
Schema.org[341]	Not peer-reviewed	Publicly accessible website	2022	Data schema		Discovered after Alpha version development, informed Beta version
HTA Core Model 3.0[332]	Not peer-reviewed	Publicly accessible website	2016	Data sharing		Discovered after Alpha version development, informed Beta version
Including all voices in international data-sharing governance[305]	Not peer-reviewed	Open Access	2018	Data sharing standards		Discovered after Alpha version development, informed Beta version
International Organization for Standardization (ISO)[96]	Not peer-reviewed	Publicly accessible website	2020	Data standardisation	International Organization for Standardization	Informed terminology to describe management of international standards

Name	Status	Access	Year	Field	Reporting standard, guideline or taxonomy	Aspects which informed STARDIT
					(multiple standards)	(including creation process for standards), including informing the design of STARDIT to align with ISO standards
Schema.org[97]	Not peer-reviewed	Publicly accessible website	2021	Data standardisation and structuring		Informed terminology for data structuring and to describe methods of collaboratively managing data standards
CDISC[311]	No peer-reviewed	Publicly Accessible Website	2020	Data standardization		Discovered after Alpha version development, informed Beta version
The Verge[306]	Not peer-reviewed	Publicly Accessible Website	2021	Defining 'on background' and accountability		Discovered after Alpha version development, informed Beta version
Using DNA Barcodes to Identify and Classify Living Things[380]	Not peer-reviewed	Publicly accessible website	2022	DNA Barcodes		Discovered after Alpha version development, informed Beta version
Husereau[98]	Peer-reviewed	Open access	2013	Economic evaluations of health interventions	Consolidated Health Economic Evaluation Reporting Standards (CHEERS) statement	Informing terminology for economic outcome measures
Schagner[99]	Peer-reviewed	Paywall access	2013	Ecosystem mapping		Informed terminology for

Name	Status	Access	Year	Field	Reporting standard, guideline or taxonomy	Aspects which informed STARDIT
				and management		describing methods of mapping people's 'values' in the context of 'ecosystem services', including concepts such as 'site-specific policy'
Guidance for stakeholder consultation to support national ecosystem services assessment: A case study from French marine assessment[303]	Peer-reviewed	Publicly Accessible Website	2022	Ecosystems services assessment		Discovered after Alpha version development, informed Beta version
Education Endowment Foundation[100]	Not peer-reviewed	Publicly accessible website	2020	Education		Informed terminology for standardised reporting of educational outcomes and evaluating educational interventions, including measuring concepts such as 'essential skills' and 'non-academic' outcomes, and using randomised controlled trials in an education context
SPECTRUM Database, Education Endowment Foundation[101]	Not peer-reviewed	Publicly accessible website	2020	Education		Informed terminology for standardised comparisons and review of 'non-academic' and 'essential skills' in

Name	Status	Access	Year	Field	Reporting standard, guideline or taxonomy	Aspects which informed STARDIT
						relation to outcomes
Star Portal, Australian Government, Office of The Chief Scientist[102]	Not peer-reviewed	Publicly accessible website	2020	Education		
The International Database of Education Systematic Reviews (IDESR)[103]	Not peer-reviewed	Publicly accessible website	2021	Education		
Tikly[104]	Peer-reviewed	Paywall access	2015	Education (evaluation)		Informed terminology to describe ways of consistently defining concepts such as 'learning', including facilitators and barriers or learning.
Atlas of Life[105]	Not peer-reviewed	Publicly accessible website	2020	Environment		
GlobalFungi Database, GlobalFungi	Not peer-reviewed	Publicly accessible website	2020	Environment (fungi)		
Impact report 2019, International Tree Foundation[106]	Not peer-reviewed	Publicly accessible website	2019	Environmental		Informed data categories for reporting impact from tree planting
NABERS[314]	Not peer-reviewed	Publicly Accessible	2022	Environmental impact of	NABERS	Discovered after Alpha version development, informed Beta version

Name	Status	Access	Year	Field	Reporting standard, guideline or taxonomy	Aspects which informed STARDIT
		Website		infrastructure		
Mackay[107]	Peer-reviewed	Paywall access	2019	Environmental Psychology		Informed terminology for describing and comparing behaviours as an outcome measure (for example 'pro-environmental behaviour')
DataONE[108]	Not peer-reviewed	Publicly accessible website	2020	Environmental science		Informed terminology to describe data standardisation
Citizen Science Evaluation Rubric, Port Phillip EcoCentre[109]	Not peer-reviewed	Publicly accessible website	2019	Evaluating citizen science	Citizen Science Evaluation Rubric	Informed terminology and methods of evaluating citizen science
Reed[110]	Peer-reviewed	Open access	2018	Evaluating involvement and engagement in interdisciplinary research	A common standard for the evaluation of public engagement with research	Informed general terminology, informed ways of evaluating involvement in research and other data collection
Maccarthy[111]	Peer-reviewed	Open access	2019	Evaluating involvement in basic and preclinical health research		Informed terminology and evaluation, including involvement assessment survey
Barber[112]	Peer-reviewed	Open access	2012	Evaluating involvement in health and social		Informed terminology to describe involvement in

Name	Status	Access	Year	Field	Reporting standard, guideline or taxonomy	Aspects which informed STARDIT
				research		research, including evaluation and impact assessment
Crocker[113]	Peer-reviewed	Open access	2018	Evaluating involvement in health research		Informed general terminology, including assessing impact of involvement in clinical trials and evidence synthesis
Edelman[114]	Peer-reviewed	Open access	2016	Evaluating involvement in health research		Informed general terminology, including evaluating involvement as part of research or initiative (not a distinct intervention)
Staley [115]	Peer-reviewed	Open access	2019	Evaluating involvement in health research		Informed general terminology, informed ways of describing learning as an outcome of involvement in research. Informed reporting methodology including language to describe reporting short, medium and longer term outcomes from involvement. Informed terminology to describe impacts from involvement.
United Nations Evaluation Group (UNEG)[116]	Not peer-reviewed	Publicly accessible	2013	Evaluation of functions and products of UN	United Nations Evaluation Group	Impact evaluation informed by ‘Impact Evaluation

Name	Status	Access	Year	Field	Reporting standard, guideline or taxonomy	Aspects which informed STARDIT
		website		entities	(UNEG)	Guidance Document'
A typology of reviews: an analysis of 14 review types and associated methodologies[367]	Peer-reviewed	Open access	2009	Evidence based practice	SALSA	Discovered after Alpha version development, informed Beta version
Guidelines and Standards for Evidence Synthesis in Environmental Management, The Collaboration for Environmental Evidence[117]	Not peer-reviewed	Publicly accessible website	2018	Evidence Synthesis (Environmental Management)	Guidelines and Standards for Evidence Synthesis in Environmental Management<br>VERSION 5.0	Informed terminology to describe evidence syntheses of data about environmental policy and practice as a public service
Guideline for Rapid Evidence Assessments in Management and Organizations, Center for Evidence-Based Management [118]	Not peer-reviewed	Publicly accessible website	2017	Evidence synthesis (organisational management)	Guideline for Rapid Evidence Assessments in Management and Organizations (Center for Evidence-Based Management)	Informed terminology for describing evidence assessment and synthesis
Supporting the Use of Research Evidence (SURE)[119]	Not peer-reviewed	Publicly accessible website	2011	Evidence-informed policy		Informed terminology to describe evidence-informed policy, informed terminology to describe stakeholders
External quality assessment Specification, Her Majesty's	Not peer-reviewed	Publicly accessible	2019	External quality assessment (Governm		Informed terminology to describe external quality

Name	Status	Access	Year	Field	Reporting standard, guideline or taxonomy	Aspects which informed STARDIT
Treasury (UK)[120]		website		ent services)		assessment for internal Government auditing
Royal College of Pathologists of Australasia Quality Assurance Programs, Royal College of Pathologists of Australasia[121]	Not peer-reviewed	Publicly accessible website	2020	External quality assessment (patholog y testing)		Informed terminology to describe external quality assessment for pathology assessment
How we're using AI to scale up global fact checking[335]	Not peer-reviewed	Publicly accessible website	2020	Fact checking		Discovered after Alpha version development, informed Beta version
IRIS+ [122]	Not peer-reviewed	Publicly accessible website	2020	Finance	IRIS+ Standards	Informed terminology and metrics for reporting impact for organisations and initiatives
A real options framework for reducing emissions from deforestation: Reconciling short-term incentives with long-term benefits from conservation and agricultural intensification[326]	Peer-reviewed	Open access	2021	Forest conservation	REDD+	Discovered after Alpha version development, informed Beta version
Forest Stewardship Council[336]	Not peer-reviewed	Publicly accessible website	2015	Forest conservation		Discovered after Alpha version development, informed Beta version

Name	Status	Access	Year	Field	Reporting standard, guideline or taxonomy	Aspects which informed STARDIT
Standardized Forest Stewardship Council Forestry Management audit reporting, Forest Stewardship Council[123]	Not peer-reviewed	Publicly accessible website	2020	Forest management (reporting and auditing)	Standardized Forest Stewardship Council Forestry Management audit reporting	Informed terminology to describe forest standardised reports of data from audits and management
Global Fungi Database[297]	Peer-reviewed	Publicly Accessible Website	2020	Fungi Genomics	NGS Fungal sequences	Discovered after Alpha version development, informed Beta version
The Personal Genome Project[337]	Not peer-reviewed	Publicly accessible website	2022	Genomics		Discovered after Alpha version development, informed Beta version
GeoBlacklight[338]	Not peer-reviewed	Publicly accessible website	2022	Geospatial data		Discovered after Alpha version development, informed Beta version
Geospatial Data Project[391]	Peer-reviewed	Open access	2022	Geospatial data	GBL	
Open Geospatial Consortium[348]	Not peer-reviewed	Publicly accessible website	2022	Geospatial information		Discovered after Alpha version development, informed Beta version
Open Geospatial Consortium Standards (OGC)[124]	Not peer-reviewed	Publicly accessible website	2020	Geospatial mapping	Open Geospatial Consortium Standards (OGC)	Informed terminology to describe standards in geospatial data
Standards and Interoperability Registry, Global Earth	Not peer-reviewed	Publicly accessible	2020	Geospatial mapping	Standards and Interoperability	

Name	Status	Access	Year	Field	Reporting standard, guideline or taxonomy	Aspects which informed STARDIT
Observation System of Systems (GEOSS)[125]		website			Registry (Global Earth Observation System of Systems)	
OpenWeb[301]	Not peer-reviewed	Publicly Accessible Website	2021	Global standards and norms of behaviour		Discovered after Alpha version development, informed Beta version
Department of Education, Skills and Employment[393]	Not peer-reviewed	Publicly Accessible Website	2022	Government data		
The Online System for Central Accounting and Reporting (OSCAR), Her Majesty's Treasury (UK)[126]	Not peer-reviewed	Publicly accessible website	2020	Government financial reporting (UK)		Informed terminology to describe inter-departmental Government financial data
Department of Education, Skills and Employment[355]	Not peer-reviewed	Publicly accessible website	2022	Government reporting		Discovered after Alpha version development, informed Beta version
Digital Service Standard, Digital Transformation Office (DTO) Australian Government[127]	Not peer-reviewed	Publicly accessible website	2016	Government services (online)	Digital Service Standard	Informed standardised ways to describe online services which are 'simpler, clearer and faster for all users'
Department for Digital, Culture, Media & Sport[340]	Not peer-reviewed	Publicly accessible website	2022	Government statistics		Discovered after Alpha version development, informed Beta version
John F. Kurtzke[128]	Peer-reviewed	Open access	1983	Health	Rating neurologic	

Name	Status	Access	Year	Field	Reporting standard, guideline or taxonomy	Aspects which informed STARDIT
					impairment in multiple sclerosis: An expanded disability status scale (EDSS)	
Rosen[129]	Peer-reviewed	Paywall access	1997	Health	The international index of erectile function (IIEF): a multidimensional scale for assessment of erectile dysfunction	
Draper[39]	Peer-reviewed	Paywall access	2010	Health (community Participation)	Evaluation framework for the analysis of the process of community participation in health initiatives	Informed language to describe indicators for the assessment of community participation in health initiatives
South[130]	Peer-reviewed	Open access	2016	Health (patient and public involvement in clinical trials)		Informed terminology to describe involving people in clinical trials
Chaplin[131]	Peer-reviewed	Open access	2020	Health (Pharmacogenetics)	STROPS guideline (STrengthening the Reporting Of	

Name	Status	Access	Year	Field	Reporting standard, guideline or taxonomy	Aspects which informed STARDIT
					Pharmacogenetic Studies)	
Wang[3]	Peer-reviewed	Open access	2015	Health (reporting guideline development)		Informed terminology to describe guideline development, informed methodology of STARDIT development
Methodological Expectations of Cochrane Intervention Reviews (MECIR), Cochrane Methods[132]	Not peer-reviewed	Publicly accessible website	2020	Health (review standard)	Methodological Expectations of Cochrane Intervention Reviews (MECIR)	
Cochrane Handbook for Systematic Reviews of Interventions, Cochrane[133]	Not peer-reviewed	Publicly accessible website	2020	Health (Systematic reviews)	Cochrane Handbook for Systematic Reviews of Interventions	Informed terminology to describe methodology for conducting systematic reviews, including critical appraisal
Assessing the quality of information to support people in making decisions about their health and healthcare, The Picker Institute[134]	Not peer-reviewed	Publicly accessible website	2006	Health and healthcare information		Informed ways of describing standardised ways of assessing readability and usability of information.
Health Literacy Universal Precautions Toolkit, Agency for Healthcare Research and	Not peer-reviewed	Publicly accessible website	2015	Health and healthcare information	Agency for Healthcare Research and Quality Health	Informed terminology to describe assessing whether health information is easy to

Name	Status	Access	Year	Field	Reporting standard, guideline or taxonomy	Aspects which informed STARDIT
Quality[135]					Literacy Universal Precautions Toolkit (Second Edition)	read and understand
Preparing written information for consumers that is clear, understandable and easy to Use, Australian Commission on Safety and Quality in Health Care[136]	Not peer-reviewed	Publicly accessible website	2019	Health and healthcare information	National Safety and Quality Health Service Standards (Australia), Standard 2: Partnering with Consumers	Informed terminology to describe clear health information
Toolkit for Making Written Material Clear and Effective, U.S. Department of Health and Human Services, Centers for Medicare & Medicaid Services [137]	Not peer-reviewed	Publicly accessible website	2020	Health and healthcare information	Toolkit for Making Written Material Clear and Effective	Informed categories for describing clear communication
Clear Communication Index, Centers for Disease Control and Prevention Office of the Associate Director for Communication[138]	Not peer-reviewed	Publicly accessible website	2016	Health and healthcare information and communication		Informed terminology to describe methods to assess public communication materials, including 'scoring' of certain criteria of sources
Statement on consumer and community involvement in health and medical research, National Health and Medical Research Council (Australia)[139]	Not peer-reviewed	Publicly accessible website	2016	Health and medical research		Influenced terminology for involving people in health and medical research

Name	Status	Access	Year	Field	Reporting standard, guideline or taxonomy	Aspects which informed STARDIT
Beyond Adoption: A New Framework for Theorizing and Evaluating Nonadoption, Abandonment, and Challenges to the Scale-Up, Spread, and Sustainability of Health and Care Technologies[368]	Peer-reviewed	Open access	2017	Health and social care		Discovered after Alpha version development, informed Beta version
Patient and public involvement in health and social care research: A handbook for researchers, National Institute for Health Research[140]	Not peer-reviewed	Open access	2014	Health and social care		Informed terminology to describe people involved in research and evaluation of involvement
Brett[28,29]	Peer-reviewed	Open access	2014	Health and social care research		Informed general terminology, methods of assessing impact and informed search strategy
Smits[141]	Peer-reviewed	Open access	2020	Health and social care research (planning involvement)	The "Involvement Matrix"	Informed write-up of co-design process to create a planning tool to support patient and public involvement in research.
Guidance on co-producing a research project	Not peer-reviewed	Publicly accessible website	2018	Health and social research	Guidance on co-producing a research project	Informed general terminology, how people can be involved in stages of

Name	Status	Access	Year	Field	Reporting standard, guideline or taxonomy	Aspects which informed STARDIT
(INVOLVE) [142]					(INVOLVE)	research
Public involvement in research: values and principles framework, INVOLVE[143]	Not peer-reviewed	Publicly accessible website	2015	Health and social Research		
Pandya-Wood[144]	Peer-reviewed	Open access	2017	Health and social research (ethical public involvement)		Informed terminology to describe the stages of ethical public involvement in research before and during ethics approval processes
International Classification of Functioning, Disability and Health, World Health Organisation[145]	Not peer-reviewed	Publicly accessible website	2001	Health and wellbeing	International Classification of Functioning, Disability and Health (ICF)	
Wada[146]	Peer reviewed	Open access	2020	Health and wellbeing		Informed terminology for involving people in explaining and disseminating research
Dissemination and Implementation Toolkit (PCORI) [147]	Not peer-reviewed	Publicly accessible website	2017	Health care		Informed terminology and assessment of involvement at the stages of dissemination and implementation
Dukhani[148]	Peer-reviewed	Open access	2018	Health care		Informed general terminology, search strategy, articulating outcome measures

Name	Status	Access	Year	Field	Reporting standard, guideline or taxonomy	Aspects which informed STARDIT
Nilsen [149]	Peer-reviewed	Open access	2006	Health care		Informed general terminology, informed search strategy
Tackling Representativeness: A Roadmap and Rubric [150]	Not peer-reviewed	Publicly accessible website	2018	Health care	Tackling Representativeness: A Roadmap and Rubric	Informed terminology around representation
The Value of Engagement (PCORI) [151]	Not peer-reviewed	Publicly accessible website	2018	Health care		Informed general terminology, concepts of ‘engagement’ and ‘involvement’ and partnerships and evidence informed involvement
Abma[152]	Peer-reviewed	Open access	2018	Health care and health research		Informed terminology to describe codesign in healthcare and health research context, including ‘agenda setting’ and prioritisation, power and ‘knowledge hierarchies’
Carman[153]	Peer-reviewed	Open access	2013	Health care, population health	A framework for understanding the elements and developing interventions and policies	Informed general terminology, including different descriptions of involvement and enabling conditions that support it

Name	Status	Access	Year	Field	Reporting standard, guideline or taxonomy	Aspects which informed STARDIT
Guide to producing and sourcing quality health information, Cochrane Consumers and Communication[154]	Not peer-reviewed	Open access	2019	Health information production	Guide to Producing and Sourcing Quality Health Information	Informed terminology to describe co-production of health information and outcome measures
John. E Ware[155]	Peer-reviewed	Open access	1992	Health outcomes	The MOS 36-Item Short-Form Health Survey (SF-36)	
Stakeholder Engagement Framework, Australian Government[156]	Not peer-reviewed	Publicly accessible website	2017	Health policy	Stakeholder Engagement Framework (Australian Government)	Informed terminology and involvement planning
Barriers to community involvement in health and medical research, University of Western Australia[157]	Not peer-reviewed	Publicly accessible website	2016	Health Research		Influenced terminology around barriers to involvement in research
Dillon[52]	Peer-reviewed	Open access	2017	Health research		Informed terminology defining stages of research, outcomes and indicators of involvement
EQUATOR reporting guidelines[158]	Not peer-reviewed	Publicly accessible website	2019	Health research	EQUATOR reporting guidelines	Informed general terminology, informed design of reporting 'explicit methodology', STARDIT designed to work with all

Name	Status	Access	Year	Field	Reporting standard, guideline or taxonomy	Aspects which informed STARDIT
						EQUATOR guidelines.
How to develop a reporting guideline, The EQUATOR Network[159]	Not peer-reviewed	Publicly accessible website	2018	Health research	How to develop a reporting guideline, EQUATOR	Informed general terminology, informed the design of the process for co-creating STARDIT
Kaisler[160]	Not peer-reviewed (pre-print)	Open access	2019	Health research	Patient and public involvement and engagement in research - a "how to" guide for researchers	Informed terminology to describe involving people in health research and assessing involvement
Moher[161]	Peer-reviewed	Open access	2010	Health research	EQUATOR method for developing reporting guidelines	Informed general terminology, informed the design of the process for co-creating STARDIT
National Standards for Public Involvement in Research (UK) [38]	Not peer-reviewed	Publicly accessible website	2018	Health research	National Standards for Public Involvement in Research (UK)	General terminology, indicators of involvement (including impact)
NIHR Public Involvement Impact Working Group [162]	Not peer-reviewed	Publicly accessible website	2019	Health research		Informed definitions of impact
Patient and Public Involvement and Engagement Plan, NIHR Central Commissioning	Not peer-reviewed	Open access	2019	Health Research		Informed terminology to describe people involved in health research

Name	Status	Access	Year	Field	Reporting standard, guideline or taxonomy	Aspects which informed STARDIT
Facility[163]						
Patient engagement (Canadian Institutes of Health Research) [164]	Not peer-reviewed	Publicly accessible website	2017	Health research		Informed general terminology around involving people in health research
Public Involvement (Health Research Authority) [165]	Not peer-reviewed	Publicly accessible website	2019	Health research		Informed general terminology, including distinctions between involvement and engagement
Public Involvement Impact Assessment Framework [166]	Not peer-reviewed	Publicly accessible website	2014	Health research	Public Involvement Impact Assessment Framework (PiiAF)	General terminology, mapping values and preferences associated with involvement, assessing impacts of involvement
Staniszewska[167]	Peer-reviewed	Open access	2017	Health research	GRIPP2 reporting checklists: tools to improve reporting 832 of patient and public involvement in research	Informed terminology, ways of reporting involvement and impact, mapping preferences for involvement
Strategy for Patient-Oriented Research (SPOR), Canadian Institutes of Health Research[168]	Not peer- reviewed	Publicly accessible website	2015	Health research		Informed general terminology, including describing outcomes of involvement, and involvement in governance
Tong[169]	Peer-reviewed	Open access	2019	Health research	Reporting guideline	Informed terminology for

Name	Status	Access	Year	Field	Reporting standard, guideline or taxonomy	Aspects which informed STARDIT
				(priority setting)	for priority setting of health research (REPRISE)	reporting of priority setting of health research
Workbook to guide the development of a Patient Engagement in Research (PEIR) Plan, University of British Columbia [170]	Not peer- reviewed	Publicly accessible website	2018	Health Research		Influenced terminology to describe partnership working
Wright[171]	Peer-reviewed	Open access	2010	Health research	Critical appraisal guidelines for assessing the quality and impact of user involvement in research.	Informed general terminology, including appraising involvement in research by assessing the quality and impact of involvement
Establishing and Sustaining meaningful Patient and Public Involvement and Engagement in Research in Sri Lanka, Institute for Research and Development (Sri Lanka)[172]	Not peer-reviewed	Publicly accessible website	2018	Health research (ethics)		Informed terminology to describe involving people in ethical review of research
Claw[173]	Peer-reviewed	Open access	2018	Health research (genomic research in Indigenous communities)	A framework for enhancing ethical genomic research with Indigenous communities	

Name	Status	Access	Year	Field	Reporting standard, guideline or taxonomy	Aspects which informed STARDIT
Viergever[174]	Peer-reviewed	Open access	2010	Health research (priority setting)		
Concannon[175]	Peer-reviewed	Paywall access	2012	Health research and policy		General terminology, taxonomy for stages of research adapted from this model
The Value+ Toolkit (European Patients' forum) [176]	Not peer-reviewed	Publicly accessible website	2013	Health research and services	The Value+ Toolkit (European Patients' forum)	Informed general terminology, informed definitions of involvement and indicators of involvement and barriers to involvement. STARDIT designed to align with Value+ Toolkit
ARDC[310]	Not peer-reviewed	Publicly Accessible Website	2020	Health research data	HeSANDA	Discovered after Alpha version development, informed Beta version
Cochrane Knowledge Translation Strategy[177]	Not peer-reviewed	Publicly accessible website	2017	Health research evidence, knowledge translation		General terminology, including 'audiences' and ways of assessing knowledge translation
Tugwell[178]	Peer-reviewed	Open access	2006	Health research evidence, knowledge translation	Cascade for equity-oriented knowledge translation (6ps)	General terminology, labels for multiple stakeholders, ways of assessing facilitators and barriers (including 'community' involvement),

Name	Status	Access	Year	Field	Reporting standard, guideline or taxonomy	Aspects which informed STARDIT
						ways of involving people in co-designing research, ways of assessing equity-oriented knowledge translation
Experience Based Co-Design Toolkit[356]	Not peer-reviewed	Publicly accessible website	2017	Health sector resources	EBCD Toolkit	Discovered after Alpha version development, informed Beta version
VCCC Toolkit, Victorian Comprehensive Cancer Centre, Melbourne, Australia[179]	Not peer-reviewed	Publicly accessible website	2019	Health services and research		Informed terminology specific to involving people in Australian healthcare
A Guide for Patient Group Partners, Scottish Medicines Consortium[180]	Not peer-reviewed	Open access	2017	Health technology assessment		Informed terminology to describe people involved in health technology processes, and data which informs the assessment process
European Network for Health Technology Assessment[181]	not peer-reviewed	Publicly accessible website		Health technology assessment (methodological framework)	HTA Core Model	
Fredriksson[182]	Peer-reviewed	Paywall access	2016	Healthcare		Informed general terminology
Ogrinc[183]	Peer-reviewed	Open access	2016	Healthcare	Revised Standards for Quality Improvement Reporting Excellence	Informed terminology to describe reporting of knowledge to improve

Name	Status	Access	Year	Field	Reporting standard, guideline or taxonomy	Aspects which informed STARDIT
					(SQUIRE 2.0)	healthcare
Partnering in healthcare; A framework for better care and outcomes [184]	Not peer-reviewed	Publicly accessible website	2019	Healthcare	Development of a Patient Engagement In Research (PEIR) Plan	Influenced terminology to describe involving people in improving healthcare services
The Innovation Imperative: The Future of Drug Development, The Economist Intelligence Unit[185]	Not peer-reviewed	Publicly accessible website	2018	Healthcare		Informed general terminology including involving patients in the design and management of both clinical trials and precision medicine
SPIRIT 2013 Checklist	Peer-reviewed	Open access	2013	Healthcare (clinical trial protocols)	Standard protocol items: recommendations for interventional trials (SPIRIT 2013 Checklist)	
Moullin[186]	Peer-reviewed	Open access	2015	Healthcare (implementation science and frameworks, knowledge translation)	Generic Implementation Framework (GIF)	Informed terminology to describe implementation framework types, including the variations in terminology to describe implementation frameworks.
Domecq[187]	Peer-reviewed	Open access	2014	Healthcare research		Informed general terminology, informed search strategy

Name	Status	Access	Year	Field	Reporting standard, guideline or taxonomy	Aspects which informed STARDIT
Patient-Centered Outcomes Research Institute definition of 'Stakeholders'[188]	Not peer-reviewed	Publicly accessible website	2018	Healthcare research		Informed general terminology within the word 'stakeholder'
Planning for Consumer and Community Participation in Heath and Medical Research, University of Western Australia[189]	Not peer-reviewed	Publicly accessible website	2014	Heath Research		Informed terminology to describe people involved in research
Impact in Participatory Health Research, International Collaboration for Participatory Health Research[190]	Not peer-reviewed	Publicly accessible website	2020	Heath research (participatory)		Informed terminology to describe impact from participatory health research
MedWatch, Food and Drug Administration (USA)[191]	Not peer-reviewed	Publicly accessible website		Human Medical Products (Safety Alerts)		
Association for the Accreditation of Human Research Protection Programs, Inc (AAHRPP)[192]	Not peer-reviewed	Publicly accessible website	2018	Human research ethics (accreditation)	Association for the Accreditation of Human Research Protection Programs<br>Standard I-9	Influenced language to describe standardised accreditation of human research ethics processes

Name	Status	Access	Year	Field	Reporting standard, guideline or taxonomy	Aspects which informed STARDIT
Global Humanitarian Operational Presence Who, What, Where (3W) Portal[385]	Not peer-reviewed	Publicly accessible website	2020	Humanitarian data		Discovered after Alpha version development, informed Beta version
Report of operations, Voluntary Assisted Dying Review Board, Safer Care Victoria[193]	Not peer-reviewed	Publicly accessible website	2019	Independent monitoring and reviewing (voluntary assisted dying)		Informed ways of explaining ethical data redaction and reporting oversight of voluntary assisted dying
CARE Principles for Indigenous Data Governance, Global Indigenous Data Alliance[194]	Not peer-reviewed	Publicly accessible website		Indigenous context to open data access	CARE Principles for Indigenous Data Governance	
Ethical Concerns about Psilocybin Intellectual Property[329]	Peer-reviewed	Open access	2021	Intellectual property		Discovered after Alpha version development, informed Beta version
Indigenous Data Sovereignty and Policy[375]	Not peer-reviewed	Open access	2021	Intellectual property		Discovered after Alpha version development, informed Beta version
Indigenous Data Sovereignty: Toward an agenda[370]	Peer-reviewed	Open access	2016	Intellectual property		Discovered after Alpha version development, informed Beta version
Traditional knowledge licenses[377]	Not peer-reviewed	Publicly accessible website	2022	Intellectual property		Discovered after Alpha version development, informed Beta version
The World Factbook, Central	Not peer-reviewed	Publicly	2020	Intelligence (USA		Informed methods for

Name	Status	Access	Year	Field	Reporting standard, guideline or taxonomy	Aspects which informed STARDIT
Intelligence Agency (USA)[195]		accessible website		Government)		reporting standardised data about planet Earth. Informed terminology to describe the 'intelligence cycle', including the data types 'basic', 'current' and 'estimative'.
Fazey [196]	Peer-reviewed	Paywall access	2014	Interdisciplinary and multi-stakeholder research		Informed terminology regarding knowledge exchange in interdisciplinary and multi-stakeholder research
Code of Conduct for Evaluation in the UN system (United Nations Evaluation Group)[197]	Not peer-reviewed	Publicly accessible website	2008	Inter-disciplinary evaluation	Code of Conduct for Evaluation in the UN system (United Nations Evaluation Group)	Informed general terminology, including evaluation
Detail of Impact Evaluation Guidance Document (United Nations Evaluation Group)[198]	Not peer-reviewed	Publicly accessible website	2013	Inter-disciplinary evaluation	Detail of Impact Evaluation Guidance Document (United Nations Evaluation Group)	Informed general terminology, including evaluation of impact, indicators of impact and concepts of 'validity' and 'transparency'
Good Practice Guidelines for Follow up to Evaluations (United Nations Evaluation Group)[199]	Not peer-reviewed	Publicly accessible website	2010	Inter-disciplinary evaluation	Good Practice Guidelines for Follow up to Evaluations (United	Informed general terminology, including evaluation

Name	Status	Access	Year	Field	Reporting standard, guideline or taxonomy	Aspects which informed STARDIT
					Nations Evaluation Group)	
Guidance on Evaluating Institutional Gender Mainstreaming (United Nations Evaluation Group)[200]	Not peer-reviewed	Publicly accessible website	2018	Inter-disciplinary evaluation	Guidance on Evaluating Institutional Gender Mainstreaming (United Nations Evaluation Group)	Informed general terminology, including evaluation
Mapping & Review of Evaluation Ethics (United Nations Evaluation Group)[201]	Not peer-reviewed	Publicly accessible website	2019	Inter-disciplinary evaluation		Informed general terminology, including evaluation
Norms and Standards for Evaluation (United Nations Evaluation Group)[202]	Not peer-reviewed	Publicly accessible website	2016	Inter-disciplinary evaluation	Norms and Standards for Evaluation (United Nations Evaluation Group)	Informed general terminology, including evaluation and indicators of impact
Quality Checklist for Evaluation Reports (United Nations Evaluation Group)[203]	Not peer-reviewed	Publicly accessible website	2010	Inter-disciplinary evaluation	Quality Checklist for Evaluation Reports (United Nations Evaluation Group)	Informed general terminology, including evaluation
Quality Checklist for Evaluation Terms of Reference and Inception Reports (United Nations Evaluation Group)[204]	Not peer-reviewed	Publicly accessible website	2010	Inter-disciplinary evaluation	Quality Checklist for Evaluation Terms of Reference and Inception Reports (United Nations	Informed general terminology, including evaluation

Name	Status	Access	Year	Field	Reporting standard, guideline or taxonomy	Aspects which informed STARDIT
					Evaluation Group)	
Professional Peer Review Of The UNESCO Evaluation Function (United Nations Evaluation Group)[205]	Professional peer review	Publicly accessible website	2020	Inter-disciplinary evaluation of evaluation methodology		Informed terminology to describe methods of evaluating evaluation methodologies
Wilsdon[206]	Published book	Paywall access	2015	Inter-disciplinary evaluation of metrics and indicators		Informed terminology to describe the scope and uses and limitations of research metrics and indicators.
Glossary of Key Terms in Evaluation and Results Based Management, Organisation for Economic Co-operation and Development (OECD)[207]	Not peer-reviewed	Publicly accessible website	2002	International development		Informed terminology around impact and evaluation, informed methods of parallel definitions in multiple languages
International Aid Transparency Initiative (IATI) Standard[208]	Not peer-reviewed	Publicly accessible website	2019	International development	International Aid Transparency Initiative (IATI) Standard	Informed terminology for data reporting for international aid and development, informed terminology to describe methods of reporting and sharing data
Broadening The Range Of	Not peer-reviewed	Publicly	2012	International		Informed terminology to

Name	Status	Access	Year	Field	Reporting standard, guideline or taxonomy	Aspects which informed STARDIT
Designs And Methods For Impact Evaluation, Department for International Development (UK)[209]		accessible website		development (evaluation)		describe impact and evaluation methodology and causal inference and terminology to describe theory of change
Impact Evaluation Glossary, International Initiative for Impact Evaluation (3ie)[210]	Not peer-reviewed	Publicly accessible website	2012	International development (evaluation)		Informed terminology to describe impact and evaluation methodology
Mansuri[211]	Peer-reviewed	Publicly accessible website	2004	International development (evaluation)		Informed terminology to describe concepts such as ‘community driven’ and ‘social capital’
Olken[212]	Peer-reviewed	Paywall access	2007	International development (evaluation)		Informed terminology to describe and report concepts such as ‘corruption’, ‘auditing’ and conflicting interests
The Social Impact of Social Funds in Jamaica: A Mixed-Methods Analysis of Participation, Targeting and Collective Action in Community Driven Development, The World Bank[213]	Not peer-reviewed	Publicly accessible website	2003	International development (evaluation)		Informed terminology for reporting participation and concepts such as ‘collective action’ and ‘community driven’
Woolcock[214]	Peer-reviewed	Paywall access	2008	International development		Informed terminology for reporting methods of reporting impact, including

Name	Status	Access	Year	Field	Reporting standard, guideline or taxonomy	Aspects which informed STARDIT
				(evaluation)		long-term impact trajectories
Gibson[214]	Peer-reviewed	Paywall access	2008	International development (participatory projects)		Informed terminology for reporting concepts such as 'empowerment', 'inequity' and 'social legitimacy'
Hentschel[215]	Peer-reviewed	Paywall access	1999	International development (primary healthcare)		Informed terminology to describe reporting methodology for evaluating international development in primary healthcare
Social Determinants of Health (World Health Organization) [216]	Published book (WHO)	Open access	2003	International public health		Informed 'Other information' in describing authors categories and 'barriers' (describing potential equity issues for who was involved)
Understanding Collective Impact in Australia: A new approach to interorganizational collaboration[327]	Peer-reviewed	Open access	2018	Interorganizational collaboration		Discovered after Alpha version development, informed Beta version
Cheer[217]	Peer-reviewed	Open access	2020	Involvement in research (environment, health and wellbeing)	Know the Risk, Own the Risk, Flip the Risk framework	Informed language to describe interdisciplinary involvement of people in research (specifically Torres Strait Islanders)

Name	Status	Access	Year	Field	Reporting standard, guideline or taxonomy	Aspects which informed STARDIT
Bellingcat[318]	Not peer-reviewed	Publicly Accessible Website	2022	Journalism		Discovered after Alpha version development, informed Beta version
Public Interest Journalism Initiative[218]	Not peer-reviewed	Open access	2020	Journalism		Informed terminology to describe public interest journalism principles, including defining 'public interest' and 'plurality'
Pinnock[219]	Peer-reviewed	Open access	2017	Knowledge translation (implementation science)	Standards for Reporting Implementation Studies (StaRI) Statement	
Current Good Manufacturing Practice in Manufacturing, Packaging, Labelling, or Holding Operations for Dietary Supplements, Center for Food Safety and Applied Nutrition[220]	Not peer-reviewed	Publicly accessible website	2018	Manufacturing (Good Manufacturing Practice)	Small Entity Compliance Guide: Current Good Manufacturing Practice in Manufacturing, Packaging, Labelling, or Holding Operations for Dietary Supplements	
Therapeutic Goods Administration, Department of	Not peer-reviewed	Publicly accessible	2018	Manufacturing (principles for	Good Manufacturing Practice (GMP),	

Name	Status	Access	Year	Field	Reporting standard, guideline or taxonomy	Aspects which informed STARDIT
Health, Australian Government[221]		website		medicinal products)	Australia	
Rison[27]	Peer-reviewed	Open access	2013	Medical Case Reports	CARE (CAse REport) guidelines and the standardization of case reports	Informed terminology for standardised creating standardised descriptions, including of medical case reports
International Committee of Medical Journal Editors (defining the role of authors and contributors) [222]	Not peer-reviewed	Publicly accessible website	2019	Medical journal publication		Informed terminology defining 'authorship', including authors of STARDIT reports
ISBT 128 Standard, International Council for Commonality in Blood Banking Automation[223]	Global standard for the identification, labelling, and information transfer of medical products of human origin	Open access	2018	Medical products of human origin	ISBT 128 Standard	Informed definitions for standard identification, labelling, and information transfer of medical products of human origin
Uniform requirements for manuscripts submitted to biomedical journals: Writing and editing for biomedical publication, International Committee Of Medical Journal Editors[224]	Peer-reviewed	Open access	2010	Medical publishing	Uniform requirements for manuscripts submitted to biomedical journals: Writing and editing for biomedical publication	Informed terminology to describe articles submitted to medical journals

Name	Status	Access	Year	Field	Reporting standard, guideline or taxonomy	Aspects which informed STARDIT
World Association of Medical Editors (WAME)[225]	Not peer-reviewed	Publicly accessible website	2020	Medical publishing		Informed general terminology including quality assurance in medicine
Guidelines for reporting patient and public involvement in research, British Medical Journal[226]	Not peer-reviewed	Publicly accessible website	2019	Medical research	Guidelines for reporting patient and public involvement in research, British Medical Journal	Informed minimum reporting requirements for STARDIT, informed 'cost' and 'time' reporting
The SPIRIT Statement[227]	Not peer-reviewed	Publicly accessible website	2020	Medical trial protocols	SPIRIT	Informed terminology to describe trial protocols
Therapeutic Goods Administration (TGA), Australian Government Department of Health[228]	Not peer-reviewed	Publicly accessible website	2019	Medicine and pharmaceutical manufacturing		Informed terminology to describe good manufacturing process clearance process
Dubois[229]	Peer-reviewed	Open access	2013	Medicine comparative effectiveness research	Framework for understanding the pace of evidence adoption	Informed terminology around involving people in assessing evidence adoption
Patient Engagement Quality Guidance [230]	Not peer-reviewed	Publicly accessible website	2018	Medicine development		Informed general terminology, including assessing involvement in the stages of medicine development

Name	Status	Access	Year	Field	Reporting standard, guideline or taxonomy	Aspects which informed STARDIT
The book of good practices in Patient Engagement [231]	Not peer-reviewed	Publicly accessible website	2018	Medicine development		Informed general terminology, including defining stakeholders and stages of medicine development
Spindler[232]	Peer-reviewed	Open access	2018	Medicine research and development	European Patients Academy on Therapeutic Innovation (EUPATI) Guidelines on Patient Involvement in Research and Development	Informed general terminology, including stages aligned to guidelines
Patient Focused Medicines Development (PFMD) 'Book of Good Practices' [233]	Not peer-reviewed	Publicly accessible website	2018	Medicines research		Informed general terminology, terminology around shared 'purpose' and data collection fields – in particular the 'Patient Engagement Quality Guidance Tool' ('Planning a project' and 'Assessing an ongoing or completed project')
Research Priorities for Children and Young People's Mental Health: Interventions and Services, McPin Foundation[234]	Not peer-reviewed	Publicly accessible website	2018	Mental health		Informed terminology to describe people involved and terminology for governance

Name	Status	Access	Year	Field	Reporting standard, guideline or taxonomy	Aspects which informed STARDIT
Ennis[235]	Peer-reviewed	Open access	2018	Mental health research		Informed general terminology, including language to describe longitudinal impact of involvement.
PEMA: a Pipeline for Environmental DNA Metabarcoding Analysis[379]	Not peer-reviewed	Open access	2021	Metabarcoding	PEMA	Discovered after Alpha version development, informed Beta version
Data Catalog Vocabulary (DCAT)[344]	Peer-reviewed	Open Access	2020	Metadaata	DCAT	
Data Use Ontology[394]	Peer-reviewed	Open access	2022	Metadata	DUO	
Dublin Core Metadata initiative[345]	Not peer-reviewed	Publicly accessible website	2022	Metadata		Discovered after Alpha version development, informed Beta version
Open Trials: towards a collaborative open database of all available information on all clinical trials[392]	Peer-reviewed	Open access	2016	Metadata		
OpenTrials: towards a collaborative open database of all available information on all clinical trials[376]	Peer-reviewed	Open access	2016	Metadata	OpenTrials	Discovered after Alpha version development, informed Beta version

Name	Status	Access	Year	Field	Reporting standard, guideline or taxonomy	Aspects which informed STARDIT
Wikidata[343]	Not peer-reviewed	Publicly accessible website	2022	Metadata	PeriodO	Discovered after Alpha version development, informed Beta version
DCMI Metadata Terms[346]	Not peer-reviewed	Publicly accessible website	2022	Metadata terminology		Discovered after Alpha version development, informed Beta version
France[236]	Peer-reviewed	Open access	2019	Meta-ethnography reporting	Meta-ethnography Reporting Guidance (eMERGe)	Informed terminology to describe the reporting the synthesis process of meta-ethnography
Military Expenditure Database, Stockholm International Peace Research Institute (SIPRI)[237]	Not peer-reviewed	Publicly accessible website	2021	Military Expenditure		
Arms Industry Database, Stockholm International Peace Research Institute (SIPRI)[238]	Not peer-reviewed	Publicly accessible website	2021	Military, weapons and arms		
Arms Transfers Database, Stockholm International Peace Research Institute (SIPRI)[239]	Not peer-reviewed	Publicly accessible website	2021	Military, weapons and arms		
DCMS: A data analytics and management system for molecular simulation[390]	Peer-reviewed	Open access	2014	Molecular science	DCMS	

Name	Status	Access	Year	Field	Reporting standard, guideline or taxonomy	Aspects which informed STARDIT
Multilateral Peace Operations Database, Stockholm International Peace Research Institute (SIPRI)[240]	Not peer-reviewed	Publicly accessible website	2021	Multilateral peace operations		
CISAC[382]	Not peer-reviewed	Publicly accessible website	2022	Music	CISAC	Discovered after Alpha version development, informed Beta version
SPUN[347]	Not peer-reviewed	Publicly accessible website	2022	Mycchorizal networks and climate change		Discovered after Alpha version development, informed Beta version
Drug, Chemical & Associated Technologies Association, Inc. (DCAT)[342]	Not peer-reviewed	Publicly accessible website	2022	Not-for-profit member organisation for pharmaceutical development		Discovered after Alpha version development, informed Beta version
CitizenScience.org[339]	Not peer-reviewed	Publicly accessible website	2022	Open access databases	PPSR_CORE Program Data Model Metadata Standard / data sharing protocol	Discovered after Alpha version development, informed Beta version
Center for Open Science[241]	Not peer-reviewed	Publicly accessible website	2020	Open Science		Informed terminology, including concepts such as ‘openness’, ‘integrity’ and ‘reproducibility’
Haddaway[242]	Peer-reviewed	Open access	2018	Open science		Informed terminology for

Name	Status	Access	Year	Field	Reporting standard, guideline or taxonomy	Aspects which informed STARDIT
						evidence synthesis across disciplines
Indicator Frameworks for Fostering Open Knowledge Practices in Science and Scholarship, European Union[243]	Not peer-reviewed	Publicly accessible website	2019	Open Science	Indicator Frameworks for Fostering Open Knowledge Practices in Science and Scholarship (European Union)	Informed terminology to describe indicators of open science and other related terminology for assessing open knowledge practices
Just One Giant Lab[365]	Not peer-reviewed	Publicly accessible website	2022	Open science		Discovered after Alpha version development, informed Beta version
Open Science Manifesto[244]	Not peer-reviewed	Publicly accessible website		Open Science	Open Science Manifesto	Informed terminology for open science and describing values such as 'inclusive'
Open science monitor[245]	Not peer-reviewed	Publicly accessible website	2020	Open science		Informed terminology to describe open scientific collaboration, including application program interfaces (APIs)
Zooniverse[246]	Not peer-reviewed	Publicly accessible website	2020	Open Science		Informed terminology to describe people involved in research, data sharing and other governance

Name	Status	Access	Year	Field	Reporting standard, guideline or taxonomy	Aspects which informed STARDIT
Todd[247]	Peer-reviewed	Open access	2019	Open Science, drug discovery	Six Laws of Open Source Drug Discovery	Informed terminology to describe open science, including governance and data ownership
Dico Presentation[304]	Not peer-reviewed	Publicly Accessible Website	2022	Participation rhetoric		Discovered after Alpha version development, informed Beta version
Patient Engagement Monitoring and Evaluation Framework with metrics, Paradigm[248]	Peer-reviewed	Open access	2020	Patient engagement in healthcare and medicine development		Informed terminology to describe monitoring and evaluation of patient engagement
Esmail[249]	Peer-reviewed	Open access	2015	Patient engagement research	Evaluating patient and stakeholder engagement in research	Informed general terminology, including evaluating the impact of involvement
Health Technology Assessment International[319]	Not peer-reviewed	Publicly Accessible Website	2022	Patient involvement in health research		Discovered after Alpha version development, informed Beta version
Dawn, P. Richards[164]	Peer-reviewed	Open access	2020	Patient-orientated research in chronic pain	CIHR Strategy for Patient-Oriented Research - Patient Engagement Framework	
OpenID Self-Certification,	Not peer-reviewed	Publicly accessible	2015	Personal identification		Informed terminology to describe self-certification and

Name	Status	Access	Year	Field	Reporting standard, guideline or taxonomy	Aspects which informed STARDIT
OpenID Foundation[250]		website		(identity verification)		identify verification
Parker[251]	Peer-reviewed	Open access	2019	Pharmaceuticals		Informed terminology and reporting of competing or conflicting interests.
Test, Learn, Adapt: Developing Public Policy with Randomised Controlled Trials[252]	Not peer-reviewed	Publicly accessible website	2012	Policy		Informed terminology and methodology for measuring the impact of policy
The Rise of Experimental Government, What Works UK[253]	Not peer-reviewed	Publicly accessible website	2018	Policy		Informed terminology to describe standardised reporting in government policy, including randomised controlled trials
Using research evidence: A Practice Guide, Nesta[254]	Not peer-reviewed	Publicly accessible website	2016	Policy		Informed terminology to describe reporting and using evidence in policy (in particular social policy), informed alternative methods of evaluation used for evidence-informed decision making
Gaudet[255]	Published book chapter	Open access	2015	Policy (science and evaluation)	Model of knowledge and ignorance mobilization dynamics in science	Informed definitions of ignorance and knowledge, including concepts of 'ignorance mobilisation'
Decoloniality and anti-	Peer-reviewed	Open access	2021	Practice of ecology		Discovered after Alpha

Name	Status	Access	Year	Field	Reporting standard, guideline or taxonomy	Aspects which informed STARDIT
oppressive practices for a more ethical ecology[323]						version development, informed Beta version
Wild[256]	Peer-reviewed	Open access	2005	Principles of good practice for patient-reported outcomes	Principles of Good Practice for the Translation and Cultural Adaptation Process for Patient-Reported Outcomes	
Product Safety Australia, Australian Competition and Consumer Commission[257]	Not peer-reviewed	Publicly accessible website	2020	Product safety	Mandatory and voluntary information standards	Informed language about for reporting voluntary safety standards.
Michie[258]	Peer-reviewed	Open access	2011	Public health (evidence-based behaviour change)	The COM-B system - a framework for understanding Behaviour	Informed terminology to describe differences between interventions and policy, including definitions of education, incentivisation, coercion and enablement.
Nunn[23]	Peer-reviewed	Open access	2019	Public health genomics		Informed general terminology, informed task-based reporting categories.
EAST: Four simple ways to apply behavioural insights, The Behavioural Insights Team[259]	Not peer-reviewed	Publicly accessible website	2014	Public policy (behaviour change)	EAST: Four simple ways to apply behavioural insights	Defining and measuring outcomes of initiatives and policy

Name	Status	Access	Year	Field	Reporting standard, guideline or taxonomy	Aspects which informed STARDIT
Kneebone[260]	Peer-reviewed	Paywall access	2016	Public policy (behaviour change)	The impact-likelihood matrix: a policy tool for behaviour prioritisation	Informed terminology for describing and measuring outcomes of interventions to change behaviours
The BehaviourWorks Method[261]	Not peer-reviewed	Publicly accessible website	2019	Public policy (behaviour change)		Informed terminology to describe involving people in identifying problems and outcome measures
Crossref[262]	Not peer-reviewed	Publicly accessible website	2020	Publication and data sharing		Informed terminology to describe references and standardising research outputs
Committee on Publication Ethics (COPE)[263]	Not peer-reviewed	Publicly accessible website	2015	Publication ethics		Informed terminology, including to describe guidelines and standards
Guidance for Editors: research, audit and service evaluations, Committee on Publication Ethics (COPE)[264]	Not peer-reviewed	Publicly accessible website	2014	Publication ethics	Guidance for Editors: research, audit and service evaluations, Committee on Publication Ethics (COPE)	Informed terminology to describe the variation of international ethical standards
Principles of Transparency and	Not peer-reviewed	Publicly	2014	Publication ethics	Principles of	Informed terminology,

Name	Status	Access	Year	Field	Reporting standard, guideline or taxonomy	Aspects which informed STARDIT
Best Practice in Scholarly Publishing, Committee on Publication Ethics (COPE)[265]		accessible website			Transparency and Best Practice in Scholarly Publishing, Committee on Publication Ethics (COPE)	including concepts of ethical transparency
O'Brien[266]	Peer-reviewed	Open access	2014	Qualitative research	Standards for Reporting Qualitative Research (SRQR)	Informed checklist creation and principles for reporting guidelines
Tong[267]	Peer-reviewed	Open access	2007	Qualitative research	Consolidated criteria for reporting qualitative research (COREQ)	Informed checklist creation and principles for reporting guidelines
Identification and Reporting of Patient and Public Partner Authorship on Knowledge Syntheses: Rapid Review[321]	Peer-reviewed	Open access	2021	Quantification of the effect of public and patient involvement in medical research		Discovered after Alpha version development, informed Beta version
Toward more mindful reporting of patient and public involvement in healthcare[312]	Peer-reviewed	Open Access	2021	Quantification of the effect of public and patient involvement in medical research	GRIPP2	Discovered after Alpha version development, informed Beta version
Schulz[268]	Peer-reviewed	Open access	2010	Randomised controlled	CONSORT for	General terminology,

Name	Status	Access	Year	Field	Reporting standard, guideline or taxonomy	Aspects which informed STARDIT
				trials	randomised controlled trials	including stages of trials, outcome reporting and other categories.
Reporting guideline for priority setting of health research (REPRISE)[307]	Peer-reviewed	Open access	2019	Reporting guidelines in health research	REPRISE	Discovered after Alpha version development, informed Beta version
DELWP Output Data Standards [269]	Not peer-reviewed	Publicly accessible website	2015	Reporting standards		Informed terminology and methodology for monitoring, evaluation and reporting of environmental initiatives
Ludwig Boltzmann Gesellschaft[322]	Peer-reviewed	Open Access	2022	Research		Discovered after Alpha version development, informed Beta version
Research Activity Identifier (RAiD)[270]	Not peer-reviewed	Publicly accessible website	2018	Research (multi-disciplinary)		STARDIT designed to be interoperable with RAiD
Engagement and Impact Assessment, Australian Research Council[271]	Not peer-reviewed	Publicly accessible website	2018	Research (multi-disciplinary)*	Engagement and Impact Assessment (Australian Research Council)	Informed general terminology, including assessing involvement and impact
Our World In Data[300]	Not peer-reviewed	Publicly Accessible Website	2011	Research and data		Discovered after Alpha version development, informed Beta version
RRI Tools[360]	Peer-reviewed	Publicly	2016	Research and	RRI Toolkit	Discovered after Alpha

Name	Status	Access	Year	Field	Reporting standard, guideline or taxonomy	Aspects which informed STARDIT
		accessible website		Innovation		version development, informed Beta version
The Health Equity Framework: A Science- and Justice-Based Model for Public Health Researchers and Practitioners[372]	Peer-reviewed	Open access	2020	Research and practitioner health and equity	Health Equity Framework (HEF)	Discovered after Alpha version development, informed Beta version
Partnering with Consumers Standards, Australian Commission on Safety and Quality in Health Care[272]	Not peer-reviewed	Publicly accessible website	2011	Safety and Quality in Health Care	Partnering with Consumers Standards, Australian Commission on Safety and Quality in Health Care	Informed general terminology and informed reporting of involvement.
Wilkinson[273]	Peer-reviewed	Open access	2016	Scholarly data (multi-disciplinary)	FAIR Guiding Principles for scientific data management and stewardship	Alignment with categories for reporting on principles for scientific data
Open Toolkit for Tracking Open Science Partnership Implementation and Impact[274]	Not peer-reviewed	Publicly accessible website	2019	Science (multi-disciplinary)		Informed terminology and data collection fields
Directory of Open access Journals[275]	Not peer-reviewed	Publicly accessible	2020	Scientific publishing		Informed terminology to described metadata and

Name	Status	Access	Year	Field	Reporting standard, guideline or taxonomy	Aspects which informed STARDIT
		website				independence
European Association of Science Editors[276]	Not peer-reviewed	Publicly accessible website	2019	Scientific publishing	Guidelines for Authors and Translators of Scientific Articles to be Published in English (European Association of Science Editors)	Informed terminology, in particular the concept of avoiding 'empty words and sentences' and correct use of scientific terms
Open access Scholarly Publishers Association[277]	Not peer-reviewed	Publicly accessible website	2020	Scientific publishing		Informed terminology to describe integrity and stakeholder collaboration
Promoting Integrity in Scientific Journal Publications, Council of Science Editors [278]	White paper	Publicly accessible website	2018	Scientific publishing		Informed terminology including concepts of confidentiality, conflict of interest, authorship and contribution models such as 'contributorship'
Scientific style and format, Council of Science Editors[279]	Published book	Paywall access	2014	Scientific publishing		
WikiJournal User Group Ethics Statement[280]	Not peer-reviewed	Publicly accessible website	2020	Scientific publishing		Informed general terminology and development of core ethical principles
Participation in Scientific Research (PPSR) Core metadata standards	Not peer-reviewed	Publicly accessible	2020	Scientific Research (public participation)	Participation in Scientific Research	Informed terminology to describe concepts such as

Name	Status	Access	Year	Field	Reporting standard, guideline or taxonomy	Aspects which informed STARDIT
repository, Citizen Science Association (USA)[281]		website			(PPSR) Core metadata standards repository	'public participation in scientific research'
Tricco[282]	Peer-reviewed	Open access	2018	Scoping reviews	PRISMA extension for scoping reviews (PRISMA-ScR): Checklist and explanation	Informed terminology, including standardised reporting of scoping reviews methodology
Assessing the Impacts of Social and Environmental Standards Systems[283]	Not peer-reviewed	Publicly accessible website	2014	Social and Environmental (data standards)	Assessing the Impacts of Social and Environmental Standards Systems	Informed terminology for assessing impacts of standardised data
International Social and Environmental Accreditation and Labelling Alliance[284]	Not peer-reviewed	Publicly accessible website	2020	Social and Environmental (data standards)		Informed terminology to describe international multi-disciplinary sustainability standards, including concepts of 'credibility'
Co-production in social care: What it is and how to do it, Social Care Institute for Excellence[285]	Not peer-reviewed	Publicly accessible website	2015	Social care		Informed terminology to describe co-production in the context of social care
The Australian Survey of Social Attitudes[378]	Not peer-reviewed	Publicly accessible	2022	Social science		Discovered after Alpha version development, informed Beta version

Name	Status	Access	Year	Field	Reporting standard, guideline or taxonomy	Aspects which informed STARDIT
		website				
Brazma[286]	Peer-reviewed	Open access	2001	Standard for recording and reporting microarray-based gene expression data	Minimum information about a microarray experiment (MIAME)	Informed standardised reporting of a form of genetic data (microarray-based gene expression data)
OpenSC[357]	Not peer-reviewed	Publicly accessible website	2022	Supply chain sustainability		Discovered after Alpha version development, informed Beta version
Better Cotton[330]	Not peer-reviewed	Publicly accessible website	2022	Sustainability		Discovered after Alpha version development, informed Beta version
EITI Standard[388]	Peer-reviewed	Open access	2022	Sustainability	EITI	
Information and Data Standard for Sustainability, Forest Stewardship Council and International Social and Environmental Accreditation and Labelling Alliance[287]	Not peer-reviewed	Publicly accessible website	2020	Sustainability (data standard)	Information and Data Standard for Sustainability	Informed terminology to describe standardised data 'sustainability'
LF Energy[389]	Not peer-reviewed	Publicly accessible website	2022	Sustainable energy		Discovered after Alpha version development, informed Beta version

Name	Status	Access	Year	Field	Reporting standard, guideline or taxonomy	Aspects which informed STARDIT
Aluminum Stewardship Initiative[395]	Not peer-reviewed	Publicly accessible website	2022	Sustainable mineral stewardship		Discovered after Alpha version development, informed Beta version
Tong[288]	Peer-reviewed	Open access	2012	Synthesis of qualitative research	Enhancing transparency in reporting the synthesis of qualitative research (ENTREQ)	Informed terminology for reporting the synthesis of qualitative research
Wong[289]	Peer-reviewed	Open access	2013	Systematic review method (Realist And Meta-narrative Evidence Syntheses)	RAMESES (Realist And MEta-narrative Evidence Syntheses: Evolving Standards)	Informed terminology for reporting realist and meta-narrative evidence syntheses
Wong[290]	Peer-reviewed	Open access	2016	Systematic review method (Realist And Meta-narrative Evidence Syntheses)	RAMESES II (Realist And MEta-narrative Evidence Syntheses: Evolving Standards)	Informed terminology for reporting realist and meta-narrative evidence syntheses
Moher[291]	Peer-reviewed	Open access	2009	Systematic Reviews	Preferred Reporting Items for Systematic Reviews and Meta-Analyses: The PRISMA Statement	General terminology, including stages of systematic reviews
Pollock[292]	Peer-reviewed	Paywall access	2019	Systematic reviews in health research	ACTIVE framework to describe stakeholder	Informed terminology around defining stakeholders and ways of defining involvement

Name	Status	Access	Year	Field	Reporting standard, guideline or taxonomy	Aspects which informed STARDIT
					involvement in systematic reviews	
Pollock[293]	Peer-reviewed	Open access	2018	Systematic reviews in health research		Informed general terminology, informed design of STARDIT creation process
PROGRESS-Plus, Cochrane[294]	Not peer-reviewed	Publicly accessible website	2017	Systematic reviews in health research	PROGRESS-Plus (Cochrane)	Informed 'Other information' in describing authors categories and 'barriers' (describing potential equity issues for who was involved)
Checklist of CITES Species, Convention on International Trade in Endangered Species of Wild Fauna and Flora[295]	Multilateral treaty	Open access	1975	Trade in Endangered Species of Wild Fauna and Flora	Checklist of CITES Species (Convention on International Trade in Endangered Species of Wild Fauna and Flora)	Informed design of structured data to describe species
20 Achieving evidence interoperability in the computer age: setting evidence on FHIR[308]	Peer-reviewed	Open access	2019	Universal Standards for numerical and categorical data	FHIR	Discovered after Alpha version development, informed Beta version
Developing a Framework for Public Involvement in Mathematical and Economic Modelling: Bringing New	Peer-reviewed	Open access	2021	Vaccination and immunization modelling	MEMVIE	Discovered after Alpha version development, informed Beta version

Name	Status	Access	Year	Field	Reporting standard, guideline or taxonomy	Aspects which informed STARDIT
Dynamism to Vaccination Policy Recommendations[325]						
Inglehart[296]	Peer-reviewed	Paywall access	2006	Values and beliefs		Informed terminology for describing methods of mapping people's 'values' and other associated beliefs and perspectives
New Measures of Well-Being[366]	Peer-reviewed	Open Access	2009	Well-being	SPANE	Discovered after Alpha version development, informed Beta version
Patient Focused Medicines Development	Not-peer reviewed	Open Access	2022	Patient engagement	IMI PARADIGM	Discovered after Alpha version development, informed Beta version

*This impact assessment has been piloted with the following kinds of research; Chemical Sciences, Medical and Health Sciences, History and Archaeology, and Philosophy and Religious Studies, Environmental Sciences, Agricultural and Veterinary Sciences, Engineering, Education, Studies in Creative Arts and Writing, Language Communication and Culture, and Indigenous research.[271]

Table 7: Reporting standards, guidelines or taxonomies discovered during scoping review

During the scoping review conducted by Nunn et al in 2020[11] (to be submitted for peer-review in 2022), 158 different reporting standards, guidelines and taxonomies were discovered. The review will be published in 2022 and the preliminary results are shared here, organised first by field, and then name.

Name	Status	Access	Year	Field	Reporting standard, guideline or taxonomy
The Hong Kong Principles for assessing researchers: Fostering research integrity[371]	Peer-reviewed	Open access	2020	Academic integrity	Hong Kong Principles (HKG)
Aircraft Accident and Incident Investigation , International Civil Aviation Organization[50]	Not peer-reviewed	Paywall access	2021	Aircraft incident investigation	International Standards and Recommended Practices for aircraft accident and incident Investigation
EBCD: Experience-based co-design toolkit[374]	Not peer-reviewed	Publicly accessible website	2022	Allied Health	EBCD
Paratiro.gr[363]	Not peer-reviewed	Publicly accessible website	2022	Anthropogenic impacts on wildlife	PARATIRO
Bank Track[298]	Not peer reviewed	Publicly Accessible Website	2003	Bank Finance Activities	Evaluates the social, environmental and economic consequences of major banks financial activities
Maccarthy[53]	Peer-reviewed	Open access	2019	Basic and preclinical health research	PPI Ready: Researcher Planning Canvas
Access to Biological Collection Data (ABCD),	Not peer-reviewed	Publicly	2020	Biodiversity (Data	Access to Biological Collection

This document contains additional information relevant to the article 'Standardised Data on Initiatives (STARDIT) Beta Version' https://doi.org/10.1186/s40900-022-00363-9

Name	Status	Access	Year	Field	Reporting standard, guideline or taxonomy
Taxonomic Databases Working group (TDWG)[54]		accessible website		standardisation)	Data (Taxonomic Databases Working group)
Biodiversity Information Standards, Taxonomic Databases Working group (TDWG)[55]	Not peer-reviewed	Publicly accessible website	2020	Biodiversity (Data standardisation)	Biodiversity Information Standards, Taxonomic Databases Working group (TDWG)
Darwin Core, Darwin Core Task Group, Taxonomic Databases Working group (TDWG)[56]	Not peer-reviewed	Publicly accessible website	2020	Biodiversity (Data standardisation)	Darwin Core, Darwin Core Task Group, Taxonomic Databases Working group (TDWG)
Ecological Metadata Language, National Center for Ecological Analysis and Synthesis[57]	Not peer-reviewed	Publicly accessible website	2020	Biodiversity (Data standardisation)	Ecological Metadata Language (National Center for Ecological Analysis and Synthesis)
World Geographical Scheme for Recording Plant Distributions, Taxonomic Databases Working group (TDWG)[58]	Not peer-reviewed	Publicly accessible website	2020	Biodiversity (Data standardisation)	World Geographical Scheme for Recording Plant Distributions, (Taxonomic Databases Working group)
Encyclopedia of Life[299]	Peer-reviewed	Publicly Accessible Website	2014	Biodiversity (multidisciplinary)	Taxonomic classification of various Eukaryotes, Prokaryotes and acellular organisms
CRediT taxonomy[61]	Not peer-reviewed	Publicly accessible website	2017	Biology	Contributor Roles (CRediT) taxonomy
Medical Subject Headings (MeSH), United States National Library of Medicine[62]	Controlled and hierarchically-	Open access	2020	Biomedical and health information	Medical Subject Headings (MeSH)

Name	Status	Access	Year	Field	Reporting standard, guideline or taxonomy
	organized vocabulary				
Deverka[67]	Peer-reviewed	Paywall access	2012	Cancer Genomics	Stakeholder participation in comparative effectiveness research: defining a framework for effective engagement
Embodied Carbon in Construction Calculator (EC3) tool	Not peer-reviewed	Publicly accessible website	2019	Carbon emissions in manufacturing	Embodied Carbon in Construction Calculator (EC3) tool
Climate Active, Australian Government[68]	Not peer-reviewed	Public disclosure summary	2019	Carbon Neutral certification	Climate Active Standard Carbon Neutral Program
NISO[316]	Peer-reviewed	Publicly Accessible Website	2022	Categorization of scholarly content	JATS4R
Support Guidelines (Evaluation Support Scotland)[70]	Not peer-reviewed	Publicly accessible website	2020	Charity evaluation (multidisciplinary)	Support Guidelines (Evaluation Support Scotland)
Australian Citizen Science Association[351]	Not peer-reviewed	Publicly accessible website	2022	Citizen science	ACSA’S PROJECT FINDER
Citizen Science COST Action[76]	Not peer-reviewed	Publicly accessible website	2019	Citizen science	Citizen-Science Ontology, Standards and Data

Name	Status	Access	Year	Field	Reporting standard, guideline or taxonomy
Medborgarforskning.se[362]	Not peer-reviewed	Publicly accessible website	2020	Citizen science	ARCS
Ten Principles of Citizen Science, European Citizen Science Association (ECSA)[83]	Not peer-reviewed	Publicly accessible website	2015	Citizen Science	Ten Principles of Citizen Science (European Citizen Science Association)
Pacific iCLIM[381]	Peer-reviewed	Publicly accessible website	2019	Climate information	iCLIM
Loudon[89]	Peer-reviewed	Open access	2015	Clinical trials	PRECIS-2 tool
McIlduff[92]	Peer-reviewed	Open access	2019	Community Based Participatory Research	Model of Engaging Communities Collaboratively
Resource Description Framework (RDF), World Wide Web Consortium (WC3)[94]	not peer-reviewed	Publicly accessible website	2014	Data	World Wide Web Consortium (WC3) standards
International Organization for Standardization (ISO)[96]	Not peer-reviewed	Publicly accessible website	2020	Data standardisation	International Organization for Standardization (multiple standards)
Husereau[98]	Peer-reviewed	Open access	2013	Economic evaluations of health interventions	Consolidated Health Economic Evaluation Reporting Standards (CHEERS) statement
NABERS[314]	Not peer-reviewed	Publicly Accessible Website	2022	Environmental impact of infrastructure	NABERS

Name	Status	Access	Year	Field	Reporting standard, guideline or taxonomy
Citizen Science Evaluation Rubric, Port Phillip EcoCentre[109]	Not peer-reviewed	Publicly accessible website	2019	Evaluating citizen science	Citizen Science Evaluation Rubric
Reed[110]	Peer-reviewed	Open access	2018	Evaluating involvement and engagement in interdisciplinary research	A common standard for the evaluation of public engagement with research
United Nations Evaluation Group (UNEG)[116]	Not peer-reviewed	Publicly accessible website	2013	Evaluation of functions and products of UN entities	United Nations Evaluation Group (UNEG)
A typology of reviews: an analysis of 14 review types and associated methodologies[367]	Peer-reviewed	Open access	2009	Evidence based practice	SALSA
Guidelines and Standards for Evidence Synthesis in Environmental Management, The Collaboration for Environmental Evidence[117]	Not peer-reviewed	Publicly accessible website	2018	Evidence Synthesis (Environmental Management)	Guidelines and Standards for Evidence Synthesis in Environmental Management<br>VERSION 5.0
Guideline for Rapid Evidence Assessments in Management and Organizations, Center for Evidence-Based Management [118]	Not peer-reviewed	Publicly accessible website	2017	Evidence synthesis (organisational management)	Guideline for Rapid Evidence Assessments in Management and Organizations (Center for Evidence-Based Management)
IRIS+ [122]	Not peer-reviewed	Publicly accessible website	2020	Finance	IRIS+ Standards

Name	Status	Access	Year	Field	Reporting standard, guideline or taxonomy
A real options framework for reducing emissions from deforestation: Reconciling short-term incentives with long-term benefits from conservation and agricultural intensification[326]	Peer-reviewed	Open access	2021	Forest conservation	REDD+
Standardized Forest Stewardship Council Forestry Management audit reporting, Forest Stewardship Council[123]	Not peer-reviewed	Publicly accessible website	2020	Forest management (reporting and auditing)	Standardized Forest Stewardship Council Forestry Management audit reporting
Global Fungi Database[297]	Peer-reviewed	Publicly Accessible Website	2020	Fungi Genomics	NGS Fungal sequences
Geospatial Data Project[391]	Peer-reviewed	Open access	2022	Geospatial data	GBL
Open Geospatial Consortium Standards (OGC)[124]	Not peer-reviewed	Publicly accessible website	2020	Geospatial mapping	Open Geospatial Consortium Standards (OGC)
Standards and Interoperability Registry, Global Earth Observation System of Systems (GEOSS)[125]	Not peer-reviewed	Publicly accessible website	2020	Geospatial mapping	Standards and Interoperability Registry (Global Earth Observation System of Systems)
Digital Service Standard, Digital Transformation Office (DTO) Australian Government[127]	Not peer-reviewed	Publicly accessible website	2016	Government services (online)	Digital Service Standard
John F. Kurtzke[128]	Peer-reviewed	Open access	1983	Health	Rating neurologic impairment in multiple sclerosis: An expanded disability status scale

Name	Status	Access	Year	Field	Reporting standard, guideline or taxonomy
					(EDSS)
Rosen[129]	Peer-reviewed	Paywall access	1997	Health	The international index of erectile function (IIEF): a multidimensional scale for assessment of erectile dysfunction
Draper[39]	Peer-reviewed	Paywall access	2010	Health (community Participation)	Evaluation framework for the analysis of the process of community participation in health initiatives
Chaplin[131]	Peer-reviewed	Open access	2020	Health (Pharmacogenetics)	STROPS guideline (STrengthening the Reporting Of Pharmacogenetic Studies)
Methodological Expectations of Cochrane Intervention Reviews (MECIR), Cochrane Methods[132]	Not peer-reviewed	Publicly accessible website	2020	Health (review standard)	Methodological Expectations of Cochrane Intervention Reviews (MECIR)
Cochrane Handbook for Systematic Reviews of Interventions, Cochrane[133]	Not peer-reviewed	Publicly accessible website	2020	Health (Systematic reviews)	Cochrane Handbook for Systematic Reviews of Interventions
Health Literacy Universal Precautions Toolkit, Agency for Healthcare Research and Quality[135]	Not peer-reviewed	Publicly accessible website	2015	Health and healthcare information	Agency for Healthcare Research and Quality Health Literacy Universal Precautions Toolkit (Second Edition)
Preparing written information for consumers	Not peer-reviewed	Publicly accessible	2019	Health and healthcare	National Safety and Quality Health Service Standards

Name	Status	Access	Year	Field	Reporting standard, guideline or taxonomy
that is clear, understandable and easy to Use, Australian Commission on Safety and Quality in Health Care[136]		website		information	(Australia), Standard 2: Partnering with Consumers
Toolkit for Making Written Material Clear and Effective, U.S. Department of Health and Human Services, Centers for Medicare & Medicaid Services [137]	Not peer-reviewed	Publicly accessible website	2020	Health and healthcare information	Toolkit for Making Written Material Clear and Effective
Smits[141]	Peer-reviewed	Open access	2020	Health and social care research (planning involvement)	The "Involvement Matrix"
Guidance on co-producing a research project (INVOLVE) [142]	Not peer-reviewed	Publicly accessible website	2018	Health and social research	Guidance on co-producing a research project (INVOLVE)
International Classification of Functioning, Disability and Health, World Health Organisation[145]	Not peer-reviewed	Publicly accessible website	2001	Health and wellbeing	International Classification of Functioning, Disability and Health (ICF)
Tackling Representativeness: A Roadmap and Rubric [150]	Not peer-reviewed	Publicly accessible website	2018	Health care	Tackling Representativeness: A Roadmap and Rubric
Carman[153]	Peer-reviewed	Open access	2013	Health care, population health	A framework for understanding the elements and developing interventions and policies
Guide to producing and sourcing quality health	Not peer-reviewed	Open access	2019	Health information	Guide to Producing and

Name	Status	Access	Year	Field	Reporting standard, guideline or taxonomy
information, Cochrane Consumers and Communication[154]				production	Sourcing Quality Health Information
John. E Ware[155]	Peer-reviewed	Open access	1992	Health outcomes	The MOS 36-Item Short-Form Health Survey (SF-36)
Stakeholder Engagement Framework, Australian Government[156]	Not peer-reviewed	Publicly accessible website	2017	Health policy	Stakeholder Engagement Framework (Australian Government)
EQUATOR reporting guidelines[158]	Not peer-reviewed	Publicly accessible website	2019	Health research	EQUATOR reporting guidelines
How to develop a reporting guideline, The EQUATOR Network[159]	Not peer-reviewed	Publicly accessible website	2018	Health research	How to develop a reporting guideline, EQUATOR
Kaisler[160]	Not peer-reviewed (pre-print)	Open access	2019	Health research	Patient and public involvement and engagement in research - a “how to” guide for researchers
Moher[161]	Peer-reviewed	Open access	2010	Health research	EQUATOR method for developing reporting guidelines
National Standards for Public Involvement in Research (UK) [38]	Not peer-reviewed	Publicly accessible website	2018	Health research	National Standards for Public Involvement in Research (UK)
Public Involvement Impact Assessment Framework [166]	Not peer-reviewed	Publicly accessible	2014	Health research	Public Involvement Impact Assessment Framework (PiiAF)

Name	Status	Access	Year	Field	Reporting standard, guideline or taxonomy
		website			
Staniszewska[167]	Peer-reviewed	Open access	2017	Health research	GRIPP2 reporting checklists: tools to improve reporting 832 of patient and public involvement in research
Tong[169]	Peer-reviewed	Open access	2019	Health research (priority setting)	Reporting guideline for priority setting of health research (REPRISE)
Wright[171]	Peer-reviewed	Open access	2010	Health research	Critical appraisal guidelines for assessing the quality and impact of user involvement in research.
Claw[173]	Peer-reviewed	Open access	2018	Health research (genomic research in Indigenous communities)	A framework for enhancing ethical genomic research with Indigenous communities
The Value+ Toolkit (European Patients' forum) [176]	Not peer-reviewed	Publicly accessible website	2013	Health research and services	The Value+ Toolkit (European Patients' forum)
ARDC[310]	Not peer-reviewed	Publicly Accessible Website	2020	Health research data	HeSANDA
Tugwell[178]	Peer-reviewed	Open access	2006	Health research evidence, knowledge translation	Cascade for equity-oriented knowledge translation (6ps)

Name	Status	Access	Year	Field	Reporting standard, guideline or taxonomy
Experience Based Co-Design Toolkit[356]	Not peer-reviewed	Publicly accessible website	2017	Health sector resources	EBCD Toolkit
European Network for Health Technology Assessment[181]	not peer-reviewed	Publicly accessible website		Health technology assessment (methodological framework)	HTA Core Model
Ogrinc[183]	Peer-reviewed	Open access	2016	Healthcare	Revised Standards for Quality Improvement Reporting Excellence (SQUIRE 2.0)
Partnering in healthcare; A framework for better care and outcomes [184]	Not peer-reviewed	Publicly accessible website	2019	Healthcare	Development of a Patient Engagement In Research (PEIR) Plan
SPIRIT 2013 Checklist	Peer-reviewed	Open access	2013	Healthcare (clinical trial protocols)	Standard protocol items: recommendations for interventional trials (SPIRIT 2013 Checklist)
Moullin[186]	Peer-reviewed	Open access	2015	Healthcare (implementation science and frameworks, knowledge translation)	Generic Implementation Framework (GIF)
Association for the Accreditation of Human Research Protection Programs, Inc (AAHRPP)[192]	Not peer-reviewed	Publicly accessible website	2018	Human research ethics (accreditation)	Association for the Accreditation of Human Research Protection Programs

Name	Status	Access	Year	Field	Reporting standard, guideline or taxonomy
					Standard I-9
CARE Principles for Indigenous Data Governance, Global Indigenous Data Alliance[194]	Not peer-reviewed	Publicly accessible website		Indigenous context to open data access	CARE Principles for Indigenous Data Governance
Code of Conduct for Evaluation in the UN system (United Nations Evaluation Group)[197]	Not peer-reviewed	Publicly accessible website	2008	Inter-disciplinary evaluation	Code of Conduct for Evaluation in the UN system (United Nations Evaluation Group)
Detail of Impact Evaluation Guidance Document (United Nations Evaluation Group)[198]	Not peer-reviewed	Publicly accessible website	2013	Inter-disciplinary evaluation	Detail of Impact Evaluation Guidance Document (United Nations Evaluation Group)
Good Practice Guidelines for Follow up to Evaluations (United Nations Evaluation Group)[199]	Not peer-reviewed	Publicly accessible website	2010	Inter-disciplinary evaluation	Good Practice Guidelines for Follow up to Evaluations (United Nations Evaluation Group)
Guidance on Evaluating Institutional Gender Mainstreaming (United Nations Evaluation Group)[200]	Not peer-reviewed	Publicly accessible website	2018	Inter-disciplinary evaluation	Guidance on Evaluating Institutional Gender Mainstreaming (United Nations Evaluation Group)
Norms and Standards for Evaluation (United Nations Evaluation Group)[202]	Not peer-reviewed	Publicly accessible website	2016	Inter-disciplinary evaluation	Norms and Standards for Evaluation (United Nations Evaluation Group)
Quality Checklist for Evaluation Reports (United Nations Evaluation Group)[203]	Not peer-reviewed	Publicly accessible website	2010	Inter-disciplinary evaluation	Quality Checklist for Evaluation Reports (United Nations Evaluation Group)
Quality Checklist for Evaluation Terms of Reference and Inception Reports (United	Not peer-reviewed	Publicly accessible	2010	Inter-disciplinary	Quality Checklist for Evaluation Terms of Reference and

Name	Status	Access	Year	Field	Reporting standard, guideline or taxonomy
Nations Evaluation Group)[204]		website		evaluation	Inception Reports (United Nations Evaluation Group)
International Aid Transparency Initiative (IATI) Standard[208]	Not peer-reviewed	Publicly accessible website	2019	International development	International Aid Transparency Initiative (IATI) Standard
Cheer[217]	Peer-reviewed	Open access	2020	Involvement in research (environment, health and wellbeing)	Know the Risk, Own the Risk, Flip the Risk framework
Pinnock[219]	Peer-reviewed	Open access	2017	Knowledge translation (implementation science)	Standards for Reporting Implementation Studies (StaRI) Statement
Current Good Manufacturing Practice in Manufacturing, Packaging, Labelling, or Holding Operations for Dietary Supplements, Center for Food Safety and Applied Nutrition[220]	Not peer-reviewed	Publicly accessible website	2018	Manufacturing (Good Manufacturing Practice)	Small Entity Compliance Guide: Current Good Manufacturing Practice in Manufacturing, Packaging, Labelling, or Holding Operations for Dietary Supplements
Therapeutic Goods Administration, Department of Health, Australian Government[221]	Not peer-reviewed	Publicly accessible website	2018	Manufacturing (principles for medicinal products)	Good Manufacturing Practice (GMP), Australia
Rison[27]	Peer-reviewed	Open access	2013	Medical Case Reports	CARE (CAse REport) guidelines and the standardization of case reports
ISBT 128 Standard, International Council for Commonality in Blood Banking Automation[223]	Global standard for the identification, labelling, and	Open access	2018	Medical products of human origin	ISBT 128 Standard

Name	Status	Access	Year	Field	Reporting standard, guideline or taxonomy
	information transfer of medical products of human origin				
Uniform requirements for manuscripts submitted to biomedical journals: Writing and editing for biomedical publication, International Committee Of Medical Journal Editors[224]	Peer-reviewed	Open access	2010	Medical publishing	Uniform requirements for manuscripts submitted to biomedical journals: Writing and editing for biomedical publication
Guidelines for reporting patient and public involvement in research, British Medical Journal[226]	Not peer-reviewed	Publicly accessible website	2019	Medical research	Guidelines for reporting patient and public involvement in research, British Medical Journal
The SPIRIT Statement[227]	Not peer-reviewed	Publicly accessible website	2020	Medical trial protocols	SPIRIT
Dubois[229]	Peer-reviewed	Open access	2013	Medicine comparative effectiveness research	Framework for understanding the pace of evidence adoption
Spindler[232]	Peer-reviewed	Open access	2018	Medicine research and development	European Patients Academy on Therapeutic Innovation (EUPATI) Guidelines on Patient Involvement in Research and Development
PEMA: a Pipeline for Environmental DNA Metabarcoding Analysis[379]	Not peer-reviewed	Open access	2021	Metabarcoding	PEMA

Name	Status	Access	Year	Field	Reporting standard, guideline or taxonomy
Data Catalog Vocabulary (DCAT)[344]	Peer-reviewed	Open Access	2020	Metadaata	DCAT
Data Use Ontology[394]	Peer-reviewed	Open access	2022	Metadata	DUO
OpenTrials: towards a collaborative open database of all available information on all clinical trials[376]	Peer-reviewed	Open access	2016	Metadata	OpenTrials
Wikidata[343]	Not peer-reviewed	Publicly accessible website	2022	Metadata	PeriodO
France[236]	Peer-reviewed	Open access	2019	Meta-ethnography reporting	Meta-ethnography Reporting Guidance (eMERGe)
DCMS: A data analytics and management system for molecular simulation[390]	Peer-reviewed	Open access	2014	Molecular science	DCMS
CISAC[382]	Not peer-reviewed	Publicly accessible website	2022	Music	CISAC
CitizenScience.org[339]	Not peer-reviewed	Publicly accessible website	2022	Open access databases	PPSR_CORE Program Data Model Metadata Standard / data sharing protocol
Indicator Frameworks for Fostering Open Knowledge Practices in Science and Scholarship, European Union[243]	Not peer-reviewed	Publicly accessible website	2019	Open Science	Indicator Frameworks for Fostering Open Knowledge Practices in Science and Scholarship (European Union)

Name	Status	Access	Year	Field	Reporting standard, guideline or taxonomy
Open Science Manifesto[244]	Not peer-reviewed	Publicly accessible website		Open Science	Open Science Manifesto
Todd[247]	Peer-reviewed	Open access	2019	Open Science, drug discovery	Six Laws of Open Source Drug Discovery
Patient Focused Medicines Development	Not-peer reviewed	Open Access	2022	Patient engagement	IMI PARADIGM
Esmail[249]	Peer-reviewed	Open access	2015	Patient engagement research	Evaluating patient and stakeholder engagement in research
Dawn, P. Richards[164]	Peer-reviewed	Open access	2020	Patient-orientated research in chronic pain	CIHR Strategy for Patient-Oriented Research - Patient Engagement Framework
Gaudet[255]	Published book chapter	Open access	2015	Policy (science and evaluation)	Model of knowledge and ignorance mobilization dynamics in science
Wild[256]	Peer-reviewed	Open access	2005	Principles of good practice for patient-reported outcomes	Principles of Good Practice for the Translation and Cultural Adaptation Process for Patient-Reported Outcomes
Product Safety Australia, Australian Competition and Consumer Commission[257]	Not peer-reviewed	Publicly accessible website	2020	Product safety	Mandatory and voluntary information standards
Michie[258]	Peer-reviewed	Open access	2011	Public health (evidence-based	The COM-B system - a framework for understanding

Name	Status	Access	Year	Field	Reporting standard, guideline or taxonomy
				behaviour change)	Behaviour
EAST: Four simple ways to apply behavioural insights, The Behavioural Insights Team[259]	Not peer-reviewed	Publicly accessible website	2014	Public policy (behaviour change)	EAST: Four simple ways to apply behavioural insights
Kneebone[260]	Peer-reviewed	Paywall access	2016	Public policy (behaviour change)	The impact-likelihood matrix: a policy tool for behaviour prioritisation
Guidance for Editors: research, audit and service evaluations, Committee on Publication Ethics (COPE)[264]	Not peer-reviewed	Publicly accessible website	2014	Publication ethics	Guidance for Editors: research, audit and service evaluations, Committee on Publication Ethics (COPE)
Principles of Transparency and Best Practice in Scholarly Publishing, Committee on Publication Ethics (COPE)[265]	Not peer-reviewed	Publicly accessible website	2014	Publication ethics	Principles of Transparency and Best Practice in Scholarly Publishing, Committee on Publication Ethics (COPE)
O'Brien[266]	Peer-reviewed	Open access	2014	Qualitative research	Standards for Reporting Qualitative Research (SRQR)
Tong[267]	Peer-reviewed	Open access	2007	Qualitative research	Consolidated criteria for reporting qualitative research (COREQ)
Toward more mindful reporting of patient and public involvement in healthcare[312]	Peer-reviewed	Open Access	2021	Quantification of the effect of public and patient involvement	GRIPP2

Name	Status	Access	Year	Field	Reporting standard, guideline or taxonomy
				in medical research	
Schulz[268]	Peer-reviewed	Open access	2010	Randomised controlled trials	CONSORT for randomised controlled trials
Reporting guideline for priority setting of health research (REPRISE)[307]	Peer-reviewed	Open access	2019	Reporting guidelines in health research	REPRISE
Engagement and Impact Assessment, Australian Research Council[271]	Not peer-reviewed	Publicly accessible website	2018	Research (multi-disciplinary)*	Engagement and Impact Assessment (Australian Research Council)
RRI Tools[360]	Peer-reviewed	Publicly accessible website	2016	Research and Innovation	RRI Toolkit
The Health Equity Framework: A Science- and Justice-Based Model for Public Health Researchers and Practitioners[372]	Peer-reviewed	Open access	2020	Research and practitioner health and equity	Health Equity Framework (HEF)
Partnering with Consumers Standards, Australian Commission on Safety and Quality in Health Care[272]	Not peer-reviewed	Publicly accessible website	2011	Safety and Quality in Health Care	Partnering with Consumers Standards, Australian Commission on Safety and Quality in Health Care
Wilkinson[273]	Peer-reviewed	Open access	2016	Scholarly data (multi-disciplinary)	FAIR Guiding Principles for scientific data management and stewardship
European Association of Science Editors[276]	Not peer-reviewed	Publicly accessible website	2019	Scientific publishing	Guidelines for Authors and Translators of Scientific Articles to be Published in English

Name	Status	Access	Year	Field	Reporting standard, guideline or taxonomy
					(European Association of Science Editors)
Participation in Scientific Research (PPSR) Core metadata standards repository, Citizen Science Association (USA)[281]	Not peer-reviewed	Publicly accessible website	2020	Scientific Research (public participation)	Participation in Scientific Research (PPSR) Core metadata standards repository
Tricco[282]	Peer-reviewed	Open access	2018	Scoping reviews	PRISMA extension for scoping reviews (PRISMA-ScR): Checklist and explanation
Assessing the Impacts of Social and Environmental Standards Systems[283]	Not peer-reviewed	Publicly accessible website	2014	Social and Environmental (data standards)	Assessing the Impacts of Social and Environmental Standards Systems
Brazma[286]	Peer-reviewed	Open access	2001	Standard for recording and reporting microarray-based gene expression data	Minimum information about a microarray experiment (MIAME)
EITI Standard[388]	Peer-reviewed	Open access	2022	Sustainability	EITI
Information and Data Standard for Sustainability, Forest Stewardship Council and International Social and Environmental Accreditation and Labelling Alliance[287]	Not peer-reviewed	Publicly accessible website	2020	Sustainability (data standard)	Information and Data Standard for Sustainability
Tong[288]	Peer-reviewed	Open access	2012	Synthesis of qualitative research	Enhancing transparency in reporting the synthesis of qualitative research (ENTREQ)
Wong[289]	Peer-reviewed	Open access	2013	Systematic review method (Realist And	RAMESES (Realist And MEta-narrative Evidence Syntheses:

Name	Status	Access	Year	Field	Reporting standard, guideline or taxonomy
				Meta-narrative Evidence Syntheses)	Evolving Standards)
Wong[290]	Peer-reviewed	Open access	2016	Systematic review method (Realist And Meta-narrative Evidence Syntheses)	RAMESES II (Realist And MEta-narrative Evidence Syntheses: Evolving Standards)
Moher[291]	Peer-reviewed	Open access	2009	Systematic Reviews	Preferred Reporting Items for Systematic Reviews and Meta-Analyses: The PRISMA Statement
Pollock[292]	Peer-reviewed	Paywall access	2019	Systematic reviews in health research	ACTIVE framework to describe stakeholder involvement in systematic reviews
PROGRESS-Plus, Cochrane[294]	Not peer-reviewed	Publicly accessible website	2017	Systematic reviews in health research	PROGRESS-Plus (Cochrane)
Checklist of CITES Species, Convention on International Trade in Endangered Species of Wild Fauna and Flora[295]	Multilateral treaty	Open access	1975	Trade in Endangered Species of Wild Fauna and Flora	Checklist of CITES Species (Convention on International Trade in Endangered Species of Wild Fauna and Flora)
20 Achieving evidence interoperability in the computer age: setting evidence on FHIR[308]	Peer-reviewed	Open access	2019	Universal Standards for numerical and categorical data	FHIR
Developing a Framework for Public Involvement in Mathematical and	Peer-reviewed	Open access	2021	Vaccination and immunization	MEMVIE

Name	Status	Access	Year	Field	Reporting standard, guideline or taxonomy
Economic Modelling: Bringing New Dynamism to Vaccination Policy Recommendations[325]				modelling	
New Measures of Well-Being[366]	Peer-reviewed	Open Access	2009	Well-being	SPANE

237 # Table 8: Detailed summary of STARDIT solutions to current limitations in standardised reporting

Limitations of current models and frameworks	Solutions to current limitations proposed by this system
There are numerous models and reporting frameworks, which change according to the research discipline – making it difficult for governments and international organisations to assess impacts from interventions and creating challenges and for global change research[196].<br><br>The principles of involvement in research and other interventions (and associated methodologies such as participatory action research and citizen science) are the same across all fields (health, environmental, basic, community based participatory, education), yet a significant number of models and frameworks are specific to health research. For example, existing models such as GRIPP2 are only appropriate for certain kinds of health and social care research[396].<br><br>Recent international consensus statements have clearly defined a role for the public in research which is 'data intensive', a kind of research which will continue to grow in importance[397]. While such statements call for both an assessment of impact and an evaluation of the process, numerous studies call for the creation of a standardised framework to report involvement in this kind of research[113,148,398].	This system creates a standardised way for reporting initiatives that is consistent across all disciplines of research.<br><br>This system does not attempt to replace any other model or system, but to 'sit above' them, complimenting and incorporating other systems when required.<br><br>Journals can adopt the online reporting system and research funders can require completion.
Most other models and frameworks require research authors to know about them and adopt that framework	Those reporting on initiatives are encouraged to adopt the reporting system when submitting papers for publication (by trialling the system with publishers and research funders). The system is intended to create new metrics for research impact, which over time, will create an incentive for researchers to report involvement and impact. While initial motivation for reporting may be intrinsic, as more partner organisations (such as research funders) adopt the system, there may be formal requirements to report, or a form of prestige associated with being able to report impacts.

Some models and frameworks are behind paywalls which impedes access; or are not legally in the public domain or published under open licenses, which prevents adaptation and improvement.	The model will be published under the Creative Commons Attribution-ShareAlike 4.0 International license (CC BY-SA 4.0) which specifically allows sharing, reuse, and adaptations, with the quality of any future iterations being the responsibility of not-for-profit host organisations.
The linguistic variation between terms such as ‘involvement’, ‘engagement’ and ‘participation’ makes objective comparisons of interventions difficult. Concepts do not always easily translate to other languages, despite many research projects being multi-lingual in scope and impact.	The system will use an ‘action-based’ approach to the spectrum of involvement [399] – requiring clearly described ‘tasks’, negating a need for fixed definitions of concepts such as ‘involvement’, ‘engagement’ and ‘participation’. Where possible, terms and concepts will be mapped to a taxonomy which will allow translation between languages. The participatory model of the project means that anyone will be able to get involved in co-designing and co-defining terms use in the system.
Time delays mean that evaluating the impact of research after the project is difficult to achieve and difficult to disseminate [110]. A number of reviews have called for standardised ways of reporting involvement in research over extended periods of time in order to improve evidence-informed ways of involving people, and to understand ‘works, for whom, why, and in what circumstances’ [23,28,29].	The reports are ‘living’ and can be updated over time, allowing impact from research projects to be captured after the project has completed. This aligns with the goals of both the UN and the Organisation for Economic Co-operation and Development (OECD) Development Assistance Committee which attempts to evaluate short, medium and long term impacts from initiatives[198].<br><br>The ‘purpose’ or the ‘why’ of the initiative and the purpose of involving people in certain tasks can also be reported in a consistent way.<br><br>The reports will be hosted by the WikiJournal of Science and quality-control (checking citations) will be carried out by the Editors.

	The reports will create an evidence-base for future living systematic reviews to analyse involvement and allow evidence informed involvement.
There is a potential power imbalance and bias in only having certain stakeholders write up research and assess the impacts (for example, only researchers, not research participants or research users).	The reporting system can be led by the authors of the research, and will encourage the inclusion of multiple stakeholders in order to define outcome measures, identify indicators and collect and interpret data [196]. It will also provide an opportunity for people involved in the project to be authors of the STARDIT report.
There is often an unhelpful or unnecessary divide between labels like 'researcher', 'participant', 'patient' and 'the public' which are likely to become increasingly blurred – for example, in future citizen science projects and public health genomics [400].	The system treats all with parity, encouraging self-identification to labels which people feel are most appropriate. Anyone can be listed as carrying out any task, regardless of their label.

Detailed reporting of design using STARDIT

The information below in Table 8 summarises best-practice from a number of international models of co-design and provides practical guidance on how STARDIT can be used at these stages. The column for 'People' is a guide to how many people might be involved at each stage, which will depend on the resources of each initiative.

Table 9: Detailed reporting of design using STARDIT

**Stage**	**Tasks**	People
Stage 1: Idea identified<br>An idea for an intervention, project or research is identified and articulated	1. Articulate the idea in appropriate accessible media, including plain language, simple visuals and other culturally appropriate methods of communication.<br>Report planned initiative	<10
Stage 2: Idea refined<br>The idea is refined with a small group of stakeholders	2. Send out proposed idea and draft involvement plan to all known stakeholders<br>3. Clarify tasks, expectations, shared values and accountabilities[119,166,230]<br>4. Discuss and record who is 'involving' who? [401,402]<br>5. Seek feedback on:<br>a. **The context** (is the understanding of context accurate? Have the possible and preferred future realities been accurately mapped? Who was involved?)[403]<br>b. **The idea** (is this a priority? Are others more important or urgent? Who is deciding this?)<br>c. **The involvement plan** (Can the stakeholders think of better ways to involve people?)<br>d. **The language and terms used** to describe the idea and the people involved (are people happy to be described as 'this label')[28,404]	<20
Stage 3: Stakeholder mapping<br>Existing stakeholders attempt to map who might currently be excluded from the process	6. Ask known stakeholders to agree a map of all those currently involved and all those who are known to be currently absent [404]<br>7. Explore reasons why people might be excluded and ways of including them [38]<br>Preference Mapping	<20

Stage	Tasks	People
Stage 4: Co-create communication plan<br>Develop a communication plan to invite people to co-create involvement	8. Develop a communication plan to tell people how they can give feedback on the planned idea. This should include clear information about:<br>a. What will be done with this feedback (including who will be involved in analysing it) [404,405]<br>b. How transparent the process will be[230]<br>9. What will be kept confidential to whom and how? Who decides this?	<20
Stage 5: Share idea and involvement plan<br>Share the idea (according to the communication plan) and ask for feedback on it (including the involvement plan)	10. Using the communication plan (Stage 4), disseminate the idea and the involvement plan, including a clear invitation for people to be involved in the next stages of the process[406].<br>Report updated plan<br>**Note**: the involvement plan could ask for feedback on:<br>• What are the potential and preferred outcomes? [261,403]<br>• Who should be involved in the initiative or research?<br>• How those people should be involved in specific tasks,<br>• Which methods and modes of communication should be used.	<100
Stage 6: Analyse feedback<br>Collect and analyse feedback, share results.	11. Collect feedback<br>12. Analyse feedback according to communications plan (Stage 4) [405]<br>13. Share results of the analysis	>100
Stage 7: Finalise idea and involvement plan<br>Co-create the plan (including the plan for involving people)	14. Informed by the results of the feedback (Stage 6), co-create the plan, including how people will be involved. This should include:<br>a. Agreeing budget required for involving people and integrate this into any funding applications [176]<br>b. Agreeing how the idea and the involvement will be evaluated[271]<br>Report final plan	>100
Stage 8: Evaluate involvement	Work in partnership with stakeholders to:<br>15. Appraise the process of involving people (what worked, what could be improved)	>100

This document contains additional information relevant to the article 'Standardised Data on Initiatives (STARDIT) Beta Version' https://doi.org/10.1186/s40900-022-00363-9

Stage	Tasks	People
Evaluate the process and the impact of both the initiative and involving people in the initiative	16. Evaluate the impact of involving people on the initiative<br>17. Evaluate the impact of the initiative	

References

1. Nunn, J. & Chang, S. Guidance for planning, reporting and evaluating initiatives: A multidisciplinary scoping review [pre-print]. https://en.wikiversity.org/wiki/WikiJournal_Preprints/Guidance_for_planning,_reporting_and_evaluating_initiatives:_A_multidisciplinary_scoping_review.

2. Nunn, J. *et al.* Standardised Data on Initiatives - STARDIT: Alpha Version. (2019) doi:10.31219/osf.io/5q47h.

3. Wang, X. *et al.* Methodology and reporting quality of reporting guidelines: systematic review. *BMC Medical Research Methodology* **15**, 74 (2015).

4. Science for All. Science For All - About. https://web.archive.org/web/20190708071126/https://scienceforall.world/about/ (2019).

5. Committee on Publication Ethics (COPE). *Guidelines on Good Publication Practice*. (1997).

6. Jack Nunn. *STARDIT Public Consultation Report – September To December 2019*. (2020).

7. Nunn, J. S. *Standardised Data on Initiatives (STARDIT) Public consultation report – September 2019 to May 2021*. (2021).

8. Science For All. STARDIT + GBIF - Science For All. https://web.archive.org/web/20210819071238/https://scienceforall.world/stardit/gbif/.

9. Jasanoff, S. States of knowledge : the co-production of science and social order. https://www.worldcat.org/title/states-of-knowledge-the-co-production-of-science-and-social-order/oclc/56545415 (2004).

10. Science for All (charity). *Science for All Values Statement*. (2019).

11. United Nations. *Universal Declaration of Human Rights*. (1948).

12. International Collaboration for Participatory Health Research (ICPHR). *Position Paper 1: What is Participatory Health Research? Version: May 2013*. *International Collaboration for Participatory Health Research* (2013).

13. Macaulay, A. C. Participatory research: What is the history? Has the purpose changed? *Family Practice* **351**, cmw117 (2016).

14. Internet Archive. Internet Archive: About IA. https://archive.org/about/ (2018).

15. Gagnon Thompson, S. C. & Barton, M. A. Ecocentric and anthropocentric attitudes toward the environment. *Journal of Environmental Psychology* **14**, 149–157 (1994).

16. Nordheim, L. V., Gundersen, M. W., Espehaug, B., Guttersrud, Ø. & Flottorp, S. Effects of School-Based Educational Interventions for Enhancing Adolescents Abilities in Critical Appraisal of Health Claims: A Systematic Review. *PLOS ONE* **11**, e0161485 (2016).

17. Nsangi, A. *et al.* Effects of the Informed Health Choices primary school intervention on the ability of children in Uganda to assess the reliability of claims about treatment effects: a cluster-randomised controlled trial. *The Lancet* **390**, 374–388 (2017).

18. Wikimedia Foundation. Phase I Clinical Trial - Wikidata Definition. https://www.wikidata.org/wiki/Q42824069 (2019).

19. Turki, H. *et al.* Wikidata: A large-scale collaborative ontological medical database. *Journal of Biomedical Informatics* vol. 99 103292 (2019).

20. National Library of Medicine. Medical Subject Headings.

21. Cochrane. Cochrane crowd. http://crowd.cochrane.org/index.html (2019).

22. Uher, R. Genomics and the classification of mental illness: Focus on broader categories. *Genome Medicine* (2013) doi:10.1186/gm501.

23. Nunn, J. S., Tiller, J., Fransquet, P. D. & Lacaze, P. Public Involvement in Global Genomics Research: A Scoping Review. *Frontiers in Public Health* **7**, 79 (2019).

24. Wikipedia. Google Knowledge Graph . https://en.wikipedia.org/w/index.php?title=Google_Knowledge_Graph&oldid=1002573625.

25. Guest, G., MacQueen, K. & Namey, E. *Applied Thematic Analysis*. (SAGE Publications, Inc., 2012). doi:10.4135/9781483384436.

26. Saldaña, J. *The Coding Manual for Qualitative Researchers (2nd Ed.)*. *SAGE Publications Inc.* (SAGE Publications, 2013). doi:10.1017/CBO9781107415324.004.

27. Rison, R. A., Kidd, M. R. & Koch, C. A. The CARE (CAse REport) guidelines and the standardization of case reports. *Journal of Medical Case Reports* **7**, 261 (2013).

28. Brett, J. *et al.* Mapping the impact of patient and public involvement on health and social care research: a systematic review. *Health Expect* **17**, 637–650 (2014).

29. Brett, J. *et al.* A Systematic Review of the Impact of Patient and Public Involvement on Service Users, Researchers and Communities. *Patient* **7**, 387–395 (2014).

30. Elliott, J. H. *et al.* Living systematic review: 1. Introduction-the why, what, when, and how. *J Clin Epidemiol* **91**, 23–30 (2017).

31. iNaturalist. What is the data quality assessment and how do observations qualify to become “Research Grade.” https://www.inaturalist.org/pages/help#quality (2020).

32. Mann, J. Health and human rights: if not now, when? *Am J Public Health* **96**, 1940–3 (1997).

33. Evans, D. *et al.* Public involvement in research: assessing impact through a realist evaluation. *Health Services and Delivery Research* **7**, 1–128 (2014).

34. Oliver, S. R. *et al.* A multidimensional conceptual framework for analysing public involvement in health services research. *Health Expectations* **11**, 72–84 (2008).

35. Sarrami-Foroushani, P., Travaglia, J., Debono, D. & Braithwaite, J. Key concepts in consumer and community engagement: a scoping meta-review. *BMC Health Serv Res* **14**, 250 (2014).

36. Stokes, F. *et al.* Public involvement in research: making sense of the diversity. *J Health Serv Res Policy* **20**, 45–51 (2014).

37. INVOLVE. *Guidance on co-producing a research project*. (2018).

38. INVOLVE. *National Standards for Public Involvement*. (2018).

39. Draper, A. K., Hewitt, G. & Rifkin, S. Chasing the dragon: Developing indicators for the assessment of community participation in health programmes. *Social Science and Medicine* **71**, 1102–1109 (2010).

40. Concannon, T. W. *et al.* Practical Guidance for Involving Stakeholders in Health Research. *Journal of General Internal Medicine* 1–6 (2018) doi:10.1007/s11606-018-4738-6.

41. Collins, H. M. & Evans, R. Studies of Expertise and Experience.

42. Hill, S. Involving the Consumer in Health Research. in *Researching Health: Qualitative, Quantitative and Mixed Methods* (eds. Saks, M. & Allsop, J.) (Sage, 2011).

43. Gradinger, F. *et al.* Values associated with public involvement in health and social care research: a narrative review. *Health Expect* **18**, 661–75 (2015).

44. United Nations For Indigenous Peoples. Indigenous Peoples at the UN. https://www.un.org/development/desa/indigenouspeoples/about-us.html (2018).

45. United Nations. *Data Strategy of the Secretary-General for Action by Everyone, Everywhere*. (2020).

46. Watts, A. Alan Watts. *Wikiquote* https://web.archive.org/web/20200913043749/https://en.wikiquote.org/wiki/Alan_Watts.

47. Manheim, D. & Garrabrant, S. Categorizing Variants of Goodhart's Law. (2018).

48. Strathern, M. 'Improving ratings': audit in the British University system. *European Review* **5**, 305–321 (1997).

49. Shortell, S. M. *et al.* An Early Assessment of Accountable Care Organizations' Efforts to Engage Patients and Their Families. *Medical Care Research and Review* **72**, 580–604 (2015).

50. International Civil Aviation Organization. Aircraft Accident and Incident Investigation. https://www.icao.int/safety/airnavigation/aig/pages/documents.aspx.

51. Victorian Agency for Health Information. About the Victorian Agency for Health Information. https://vahi.vic.gov.au/about-vahi (2020).

52. Dillon, E. C., Tuzzio, L., Madrid, S., Olden, H. & Greenlee, R. T. Measuring the Impact of Patient-Engaged Research: How a Methods Workshop Identified Critical Outcomes of Research Engagement. *Journal of Patient-Centered Research and Reviews* **4**, 237–246 (2017).

53. Maccarthy, J., Guerin, S., Wilson, A. G. & Dorris, E. R. Facilitating public and patient involvement in basic and preclinical health research. *PLOS ONE* **14**, e0216600 (2019).

54. Taxonomic Databases Working group (TDWG). Access to Biological Collection Data. (2020).

55. Taxonomic Databases Working group (TDWG). Biodiversity Information Standards. https://www.tdwg.org/.

56. Darwin Core Task Group, B. I. S. (TDWG). Darwin Core. https://www.tdwg.org/standards/dwc/ (2020).

57. National Center for Ecological Analysis and Synthesis. Ecological Metadata Language (EML). https://eml.ecoinformatics.org/ (2020).

58. Taxonomic Databases Working group (TDWG). World Geographical Scheme for Recording Plant Distributions. https://www.tdwg.org/standards/wgsrpd/.

59. Selinske, M. J. *et al.* Monitoring and evaluating the social and psychological dimensions that contribute to privately protected area program effectiveness. *Biological Conservation* **229**, 170–178 (2019).

60. Whitburn, J., Linklater, W. & Abrahamse, W. Meta-analysis of human connection to nature and proenvironmental behavior. *Conservation Biology* **34**, 180–193 (2020).

61. The Company of Biologists. Author contributions: CRediT taxonomy. *2019* http://dev.biologists.org/content/author-contributions.

62. United States National Library of Medicine. Medical Subject Headings (MeSH). https://www.nlm.nih.gov/mesh/meshhome.html (2020).

63. Shippee, N. D. *et al.* Patient and service user engagement in research: A systematic review and synthesized framework. *Health Expectations* **18**, 1151–1166 (2015).

64. Mark2Cure. About Mark2Cure. https://mark2cure.org/about/ (2020).

65. Communities For Impact. Virtual Communities For Impact. *2019* https://communitiesforimpact.org/.

66. B Lab. B Impact Assessment. https://bimpactassessment.net/how-it-works/assess-your-impact (2020).

67. Deverka, P. A. *et al.* Stakeholder participation in comparative effectiveness research: defining a framework for effective engagement. *J Comp Eff Res* **1**, 181–194 (2012).

68. Australian Government. Climate Active. https://www.climateactive.org.au/be-climate-active/certification (2021).

69. Charity Evaluation Working Group. Defining “evidence” and it’s role in decision making. https://www.chewgroup.org.uk/post/defining-evidence-and-it-s-role-in-decision-making (2020).

70. Evaluation Support Scotland. Evaluation Support Scotland. http://www.evaluationsupportscotland.org.uk/ (2020).

71. Fundraising Regulator. Code of Fundraising Practice. https://www.fundraisingregulator.org.uk/code.

72. Australian Charities and Not-for-profits Commission. The Annual Information Statement. https://www.acnc.gov.au/for-charities/annual-information-statement.

73. Charity Navigator. Charity Navigator. https://www.charitynavigator.org/index.cfm?bay=content.view&cpid=5593.

74. Australian Citizen Science Association. 10 Principles of Citizen Science. https://citizenscience.org.au/10-principles-of-citizen-science/.

75. Project Finder | Australian Citizen Science Project Finder. https://biocollect.ala.org.au/acsa#isCitizenScience%3Dtrue%26isWorldWide%3Dfalse%26max%3D20%26sort%3DdateCreatedSort.

76. Citizen Science COST Action. *On citizen-science ontology, standards and data*. (2019).

77. European Union. EU-Citizen.Science. https://eu-citizen.science/about/ (2020).

78. Göbel, C. Open Participatory Research - Four Questions to reflect on Openness in Citizen Science practice. (2019).

79. Göbel, C., Nold, C., Berditchevskaia, A. & Haklay, M. How Does Citizen Science “Do” Governance? Reflections from the DITOs Project. *Citizen Science: Theory and Practice* (2019) doi:10.5334/cstp.204.

80. SciStarter. SciStarter. https://scistarter.org/about (2020).

81. Soleri, D., Long, J. W., Ramirez-Andreotta, M. D., Eitemiller, R. & Pandya, R. Finding Pathways to More Equitable and Meaningful Public-Scientist Partnerships. *Citizen Science: Theory and Practice* (2016) doi:10.5334/cstp.46.

82. Strasser, B. J., Baudry, J., Mahr, D., Sanchez, G. & Tancoigne, E. “Citizen science”? Rethinking science and public participation. *Science and Technology Studies* (2019) doi:10.23987/sts.60425.

83. European Citizen Science Association (ECSA). Ten Principles of Citizen Science. https://ecsa.citizen-science.net/sites/default/files/ecsa_ten_principles_of_citizen_science.pdf (2015).

84. Citscibio. citscibio - About Us. https://citscibio.org/about (2020).

85. Borda, A., Gray, K. & Fu, Y. Research data management in health and biomedical citizen science: practices and prospects. *JAMIA Open* doi:10.1093/JAMIAOPEN/OOZ052.

86. QuestaGame. Core Values . https://questagame.com/core-values (2020).

87. Sacristán, J. A. *et al.* Patient involvement in clinical research: Why, when, and how. *Patient Preference and Adherence* vol. 10 631–640 (2016).

88. Bloom, D. *et al.* The Rules of Engagement. *Therapeutic Innovation & Regulatory Science* **52**, 206–213 (2018).

89. Loudon, K. *et al.* The PRECIS-2 tool: designing trials that are fit for purpose. *BMJ* **350**, h2147 (2015).

90. Australian Clinical Trials Alliance. Toolkit for Researchers and Research Organisations. https://involvementtoolkit.clinicaltrialsalliance.org.au/toolkit/ (2019).

91. Federal Communications Commission. FCC ID Search. https://fccid.io/.

92. Mcilduff, C. *et al.* Model of Engaging Communities Collaboratively. *International Journal of Critical Indigenous Studies* **13**, (2019).

93. Global Environment Facility. *Impact Evaluation - Case Study: Bwindi Impenetrable National Park and Mgahinga Gorilla National Park Conservation Project*. (2007).

94. The World Wide Web Consortium (W3C). RDF - Semantic Web Standards. https://www.w3.org/RDF/.

95. Initiative for Open Citations. Initiative for Open Citations (I4OC). https://i4oc.org/ (2020).

96. International Organization for Standardization (ISO). ISO - Standards. https://www.iso.org/standards.html (2020).

97. Schema. Schema.org. https://schema.org/ (2021).

98. Husereau, D. *et al.* Consolidated Health Economic Evaluation Reporting Standards (CHEERS) statement. *Bmj* **346**, 1–6 (2013).

99. Schägner, J. P., Brander, L., Maes, J. & Hartje, V. Mapping ecosystem services' values: Current practice and future prospects. *Ecosystem Services* vol. 4 33–46 (2013).

100. Education Endowment Foundation. Evaluation. https://educationendowmentfoundation.org.uk/projects-and-evaluation/evaluating-projects/ (2020).

101. Education Endowment Foundation. SPECTRUM Database . https://educationendowmentfoundation.org.uk/projects-and-evaluation/evaluating-projects/measuring-essential-skills/spectrum-database/.

102. The STARportal | Home. https://starportal.edu.au/.

103. IDESR. International Database of Education Systematic Reviews. https://idesr.org/.

104. Tikly, L. What works, for whom, and in what circumstances? Towards a critical realist understanding of learning in international and comparative education. *International Journal of Educational Development* **40**, 237–249 (2015).

105. About — Atlas of Life. https://atlasoflife.org.au/about-1.

106. International Tree Foundation. Impact report 2019. https://web.archive.org/web/20210102220323/https://internationaltreefoundation.org/wp-content/uploads/2020/07/Impact-Report-2019-compressed.pdf.

107. Mackay, C. M. L. & Schmitt, M. T. Do people who feel connected to nature do more to protect it? A meta-analysis. *Journal of Environmental Psychology* **65**, 101323 (2019).

108. DataONE. What is DataONE? https://www.dataone.org/what-dataone (2020).

109. Port Phillip EcoCentre. *Citizen Science Evaluation Rubric*. (2019).

110. Reed, M. S. *et al.* A common standard for the evaluation of public engagement with research. *Research for All* **2**, 143–162 (2018).

111. Maccarthy, J., Guerin, S., Wilson, A. G. & Dorris, E. R. Facilitating public and patient involvement in basic and preclinical health research. *PLOS ONE* **14**, e0216600 (2019).

112. Barber, R. *et al.* Can the impact of public involvement on research be evaluated? A mixed methods study. *Health Expectations* **15**, 229–241 (2012).

113. Crocker, J. C. *et al.* Impact of patient and public involvement on enrolment and retention in clinical trials: systematic review and meta-analysis. *BMJ* **363**, k4738 (2018).

114. Edelman, N. & Barron, D. Evaluation of public involvement in research: Time for a major re-think? *Journal of Health Services Research and Policy* **21**, 209–211 (2016).

115. Staley, K. & Barron, D. Learning as an outcome of involvement in research: what are the implications for practice, reporting and evaluation? *Research Involvement and Engagement* **5**, 14 (2019).

116. United Nations. United Nations Evaluation Group (UNEG). https://web.archive.org/web/20190912054509/http://www.uneval.org/document/guidance-documents (2013).

117. The Collaboration for Environmental Evidence. Guidelines and Standards for Evidence Synthesis in Environmental Management. https://www.environmentalevidence.org/information-for-authors (2018).

118. Barends, E., Rousseau, D. M. & Briner, R. B. *CEBMa center for Evidence-Based Management Rapid Evidence Assessments in Management and Organizations*. (2017).

119. WHO. Supporting the Use of Research Evidence (SURE). *WHO* (2013).

120. Her Majesty's Treasury (UK). *External Quality Assessment Specification*.

121. Royal College of Pathologists of Australasia. RCPAQAP Quality Assurance Programs : RCPAQAP. https://rcpaqap.com.au/ (2020).

122. IRIS+. IRIS+ Standards. *2020* https://iris.thegiin.org/standards/.

123. Forest Stewardship Council. Digital Audit Report . https://fsc.org/en/innovation/digital-audit-report#one-standard-format (2020).

124. Open Geospatial Consortium (OGC). Open Geospatial Consortium Standards. https://www.ogc.org/docs/is (2020).

125. Group on Earth Observations. Group on Earth Observations Standards and Interoperability Regsitry. https://www.earthobservations.org/gci_sr.shtml (2020).

126. Her Majesty's Treasury (UK). The Online System for Central Accounting and Reporting (OSCAR) User Guide. https://www.gov.uk/government/publications/the-online-system-for-central-accounting-and-reporting-oscar-user-guide (2020).

127. Digital Transformation Agency. About the Digital Service Standard. https://www.dta.gov.au/help-and-advice/about-digital-service-standard (2016).

128. Kurtzke, J. F. Rating neurologic impairment in multiple sclerosis: An expanded disability status scale (EDSS). *Neurology* **33**, 1444–1452 (1983).

129. Rosen, R. C. *et al.* The international index of erectile function (IIEF): A multidimensional scale for assessment of erectile dysfunction. *Urology* **49**, 822–830 (1997).

130. South, A. *et al.* Models and impact of patient and public involvement in studies carried out by the Medical Research Council Clinical Trials Unit at University College London: findings from ten case studies. *Trials* **17**, 376 (2016).

131. Chaplin, M. *et al.* STrengthening the reporting of pharmacogenetic studies: Development of the STROPS guideline. *PLoS Medicine* **17**, (2020).

132. Cochrane Methods. Methodological Expectations of Cochrane Intervention Reviews (MECIR). https://community.cochrane.org/sites/default/files/uploads/Version March 2020 Final Online version.pdf.

133. Cochrane Training. Cochrane Handbook for Systematic Reviews of Interventions . https://training.cochrane.org/handbook/current (2020).

134. Coulter, A. *et al. Assessing the quality of information to support people in making decisions about their health and healthcare Picker Institute Europe*. (2006).

135. Agency for Healthcare Research and Quality. Health Literacy Universal Precautions Toolkit (Second Edition). http:/www.ahrq.gov/sites/default/files/publications/files/healthlittoolkit2_4.pdf (2015).

136. Sheet, T. I. P. Standard 2 : Partnering with Consumers Tip Sheet 5 : Preparing written information for consumers that is clear , understandable and easy to use.

137. Centres for Medicare and Medicaid Services. Toolkit for Making Written Material Clear and Effective . https://www.cms.gov/Outreach-and-Education/Outreach/WrittenMaterialsToolkit/index?redirect=/WrittenMaterialsToolkit/ (2020).

138. Centers for Disease Control and Prevention. Clear Communication Index . https://www.cdc.gov/ccindex/ (2020).

139. National Health and Medical Research Council, NMHRC & National Health and Medical Research Counci. Statement on Consumer and Community Participation in Health and Medical Research. *Health and Medical Research National Health and Medical Research Council* https://www.nhmrc.gov.au/about-us/publications/statement-consumer-and-community-involvement-health-and-medical-research (2016).

140. National Institute for Health Research. *Patient and public involvement in health and social care research: A handbook for researchers*. (2014).

141. Smits, D. W., Van Meeteren, K., Klem, M., Alsem, M. & Ketelaar, M. Designing a tool to support patient and public involvement in research projects: The Involvement Matrix. *Research Involvement and Engagement* **6**, 30 (2020).

142. INVOLVE. *Guide on co-producing a research project*. (2018).

143. INVOLVE. Public involvement in research : values and principles framework. (2015).

144. Pandya-Wood, R., Barron, D. S. & Elliott, J. A framework for public involvement at the design stage of NHS health and social care research: time to develop ethically conscious standards. *Research Involvement and Engagement* **3**, 6 (2017).

145. *International Classification of Functioning, Disability and Health World Health Organization Geneva ICF ii WHO Library Cataloguing-in-Publication Data International classification of functioning, disability and health : ICF*. (2001).

146. Wada, M. *et al.* A protocol for co-creating research project lay summaries with stakeholders: guideline development for Canada's AGE-WELL network. *Research Involvement and Engagement* **6**, 1–8 (2020).

147. Policy Research, M. *PCORI Dissemination and Implementation Toolkit*. (2017).

148. Dukhanin, V., Topazian, R. & DeCamp, M. Metrics and Evaluation Tools for Patient Engagement in Healthcare Organization- and System-Level DecisionMaking: A Systematic Review. *International Journal of Health Policy and Management* (2018) doi:10.15171/ijhpm.2018.43.

149. Nilsen, E. S. E., Myrhaug, H. T. H., Johansen, M., Oliver, S. R. & Oxman, A. D. A. Methods of consumer involvement in developing healthcare policy and research, clinical practice guidelines and patient information material. in *Cochrane Database of Systematic Reviews* (ed. Nilsen, E. S.) CD004563 (John Wiley & Sons, Ltd, 2006). doi:10.1002/14651858.CD004563.pub2.

150. National Health Council. *Tackling Representativeness: A Roadmap and Rubric*. (2018).

151. PCORI. The Value of Engagement. https://www.pcori.org/about-us/our-programs/engagement/public-and-patient-engagement/value-engagement (2018).

152. Abma, T. A. Dialogue and deliberation: New approaches to including patients in setting health and healthcare research agendas. *Action Research* (2019) doi:10.1177/1476750318757850.

153. Carman, K. L. *et al.* Patient And Family Engagement: A Framework For Understanding The Elements And Developing Interventions And Policies. *Health Affairs* **32**, 223–231 (2013).

154. Cochrane Consumers and Communication. *Guide to Producing and Sourcing Quality Health Information*. (2019).

155. Ware, J. E. & Sherbourne, C. D. *The MOS 36-Item Short-Form Health Survey (SF-36) I. Conceptual Framework and Item Selection*. vol. 30 (1992).

156. Australian Government Department of Health. *Stakeholder engagement framework*. (2017).

157. Anne McKenzie. *Barriers to community involvement in health and medical research Researcher perspectives on consumer and community involvement in research: a qualitative study*. (2016).

158. EQUATOR Network. Reporting guidelines. https://www.equator-network.org/reporting-guidelines/.

159. The EQUATOR Network. Developing your reporting guideline. https://www.equator-network.org/toolkits/developing-a-reporting-guideline/developing-your-reporting-guideline/ (2018).

160. Kaisler, R. Patient and public involvement and engagement in research - a “how to” guide for researchers. (2019).

161. Moher, D., Schulz, K. F., Simera, I. & Altman, D. G. Guidance for Developers of Health Research Reporting Guidelines. *PLoS Medicine* **7**, e1000217 (2010).

162. Simon Denegri. Downloadable definition of the impact of public involvement in health research. https://web.archive.org/web/20190708073125/https://simondenegri.com/2019/07/03/downloadable-definition-of-the-impact-of-public-involvement-in-health-research-feat-patients-carers-and-the-public/ (2019).

163. NIHR Central Commissioning Facility. *Patient and Public Involvement and Engagement Plan*. (2019).

164. Canadian Institutes & of Health Research. Patient engagement (Canadian Institutes of Health Research). http://www.cihr-irsc.gc.ca/e/45851.html (2019).

165. Health Research Authority. Public Involvement (Health Research Authority). https://www.hra.nhs.uk/planning-and-improving-research/best-practice/public-involvement/ (2019).

166. Collins, M. PiiAF The Public Involvement Impact Assessment Framework Guidance. 1–8 http://piiaf.org.uk/documents/piiaf-guidance-jan14.pdf (2014).

167. Staniszewska, S. *et al.* GRIPP2 reporting checklists: tools to improve reporting of patient and public involvement in research. (2017) doi:10.1186/s40900-017-0062-2.

168. Canadian Institutes of Health Research. *Strategy for Patient-Oriented Research Patient Engagement Framework*. (2015).

169. Tong, A. *et al.* Reporting guideline for priority setting of health research (REPRISE). *BMC Medical Research Methodology* **19**, (2019).

170. Arthritis Research Canada & University of British Columbia. *Workbook to guide the development of a Patient Engagement In Research (PEIR) Plan*. (2018).

171. Wright, D., Foster, C., Amir, Z., Elliott, J. & Wilson, R. Critical appraisal guidelines for assessing the quality and impact of user involvement in research. *Health Expectations* **13**, 359–368 (2010).

172. Institute for Research and Development (Sri Lanka). Establishing & Sustaining meaningful PPIE in Sri Lankan Research | Institute for Research & Development. https://www.ird.lk/establishing-and-sustaining-meaningful-ppie-in-research-in-srilanka/ (2018).

173. Claw, K. G. *et al.* A framework for enhancing ethical genomic research with Indigenous communities. *Nature Communications* (2018) doi:10.1038/s41467-018-05188-3.

174. Viergever, R. F., Olifson, S., Ghaffar, A. & Terry, R. F. A checklist for health research priority setting: Nine common themes of good practice. *Health Research Policy and Systems* vol. 8 36 (2010).

175. Concannon, T. W. *et al.* A new taxonomy for stakeholder engagement in patient-centered outcomes research. *J Gen Intern Med* **27**, 985–91 (2012).

176. European Patients' forum. *The Value + Toolkit*. (2013).

177. *Cochrane Knowledge Translation Strategy*. (2017).

178. Tugwell, P., Robinson, V., Grimshaw, J. & Santesso, N. Systematic reviews and knowledge translation. *Bull World Health Organ* **84**, 643–51 (2006).

179. Victorian Comprehensive Cancer Centre. VCCC Toolkit. https://www.viccompcancerctr.org/about-vccc/consumer-engagement/toolkit/ (2019).

180. Scottish Medicines Consortium. *A Guide for Patient Group Partners*. (2015).

181. EUnetHTA. HTA Core Model. https://eunethta.eu/hta-core-model/.

182. Fredriksson, M. & Tritter, J. Q. Disentangling patient and public involvement in healthcare decisions: why the difference matters. *Sociology of Health and Illness* **39**, 95–111 (2017).

183. Ogrinc, G. *et al.* SQUIRE 2.0 (Standards for QUality Improvement Reporting Excellence): Revised publication guidelines from a detailed consensus process. *BMJ Quality and Safety* **25**, 986–992 (2016).

184. Safer Care Victoria, S. G. of V. *A framework for better care and outcomes Partnering in healthcare*. (2019).

185. The Economist Intelligence Unit. *The Innovation Imperative: The Future of Drug Development Part I: Research Methods and Findings*. (2018).

186. Moullin, J. C., Sabater-Hernández, D., Fernandez-Llimos, F. & Benrimoj, S. I. A systematic review of implementation frameworks of innovations in healthcare and resulting generic implementation framework. *Health Research Policy and Systems* **13**, 16 (2015).

187. Domecq, J. P. *et al.* Patient engagement in research: a systematic review. *BMC Health Serv Res* **14**, 89 (2014).

188. PCORI. PCORI's Stakeholders. *2014* https://www.pcori.org/about-us/our-programs/engagement/pcoris-stakeholders.

189. McKenzie, A. & R, H. Planning for consumer and community Participation in Health and Medical research: A practical guide for health and medical researchers. (2014).

190. Cook, T. *et al. Position Paper No. 3: Impact in Participatory Health Research*. (2020).

191. Food and Drug Administration (USA). Medical Product Safety Information. https://www.fda.gov/safety/medwatch-fda-safety-information-and-adverse-event-reporting-program/medical-product-safety-information.

192. Association for the Accreditation of Human Research Protection Programs. Standard I-9. (2018).

193. Safer Care Victoria. Voluntary Assisted Dying report of operations (2018-19). https://www.bettersafercare.vic.gov.au/reports-and-publications/voluntary-assisted-dying-report-of-operations-2018-19#goto-download (2019).

194. Global Indigenous Data Alliance. CARE Principles of Indigenous Data Governance. https://www.gida-global.org/care.

195. Central Intelligence Agency (USA). The World Factbook. https://www.cia.gov/library/publications/the-world-factbook/.

196. Fazey, I. *et al.* Evaluating knowledge exchange in interdisciplinary and multi-stakeholder research. *Global Environmental Change* **25**, 204–220 (2014).

197. United Nations Evaluation Group. Detail of UNEG Code of Conduct for Evaluation in the UN system. https://web.archive.org/web/20191102030441/http://www.uneval.org/document/detail/100 (2008).

198. United Nations Evaluation Group. Detail of Impact Evaluation Guidance Document. https://web.archive.org/web/20190909065035/http://www.uneval.org/document/detail/1433 (2013).

199. United Nations Evaluation Group. Detail of UNEG Good Practice Guidelines for Follow up to Evaluations. https://web.archive.org/web/20180721123543/http://www.uneval.org/document/detail/610 (2010).

200. United Nations Evaluation Group. Detail of Guidance on Evaluating Institutional Gender Mainstreaming. https://web.archive.org/web/20181214115712/http://www.uneval.org/document/detail/2133 (2018).

201. United Nations Evaluation Group. Detail of Mapping & Review of Evaluation Ethics. http://www.uneval.org/document/detail/2689 (2019).

202. United Nations Evaluation Group. Detail of Norms and Standards for Evaluation. https://web.archive.org/web/20181115190641/http://uneval.org/document/detail/1914 (2016).

203. United Nations Evaluation Group. Detail of UNEG Quality Checklist for Evaluation Reports. https://web.archive.org/web/20190718110339/http://uneval.org/document/detail/607 (2010).

204. United Nations Evaluation Group. Detail of UNEG Quality Checklist for Evaluation Terms of Reference and Inception Reports. https://web.archive.org/web/20180320223951/http://www.uneval.org/document/detail/608 (2010).

205. United Nations Evaluation Group. Professional Peer Review of the UNESCO Evaluation Function. http://www.unevaluation.org/document/detail/2778 (2020).

206. Wilsdon, James. & Wilsdon, J. *The metric tide : independent review of the role of metrics in research assessment and management*.

207. Organisation for Economic Co-operation and Development. *Glossary of Key Terms in Evaluation and Results Based Management*. (2002).

208. International Aid Transparency Initiative. About the IATI Standard. https://iatistandard.org/en/about/iati-standard/.

209. Department for International Development (UK). *Broadening The Range Of Designs And Methods For Impact Evaluation*. (2012).

210. International Initiative for Impact Evaluation (3ie). *Impact Evaluation Glossary*. (2012).

211. Mansuri, G. Community-Based and -Driven Development: A Critical Review. (2004).

212. Olken, B. A. Monitoring corruption: Evidence from a field experiment in Indonesia. *Journal of Political Economy* **115**, 200–249 (2007).

213. Ibanez, A. & Rao, V. *The Social Impact of Social Funds in Jamaica: A Mixed-Methods Analysis of Participation, Targeting, and Collective Action in Community-Driven Development*. (The World Bank, 2003). doi:10.1596/1813-9450-2970.

214. Gibson, C. & Woolcock, M. Empowerment, deliberative development, and local-level politics in Indonesia: Participatory projects as a source of countervailing power. *Studies in Comparative International Development* **43**, 151–180 (2008).

215. Hentschel, J. Contextuality and data collection methods: A framework and application to health service utilisation. *Journal of Development Studies* **35**, 64–94 (1999).

216. Wilkinson, R., Marmot, M. & Academic Search Complete. *Social Determinants of Health: the Solid Facts.* (World Health Organization, 2003).

217. Cheer, K. *et al.* The case for a Torres Strait Islander-driven, long-term research agenda for environment, health and wellbeing. *Australian and New Zealand Journal of Public Health* **44**, 177–179 (2020).

218. Public Interest Journalism Initiative. About the Public Interest Journalism Initiative. https://piji.com.au/about-us/ (2020).

219. Pinnock, H. *et al.* Standards for Reporting Implementation Studies (StaRI) Statement. *BMJ (Online)* **356**, (2017).

220. Food and Drug Administration (USA). Small Entity Compliance Guide: Current Good Manufacturing Practice in Manufacturing, Packaging, Labeling, or Holding Operations for Dietary Supplements. https://www.fda.gov/regulatory-information/search-fda-guidance-documents/small-entity-compliance-guide-current-good-manufacturing-practice-manufacturing-packaging-labeling.

221. Therapeutic Goods Administration. Manufacturing principles for medicinal products. https://www.tga.gov.au/publication/manufacturing-principles-medicinal-products.

222. International Committee of Medical Journal Editors. Defining the Role of Authors and Contributors.

223. International Council for Commonality in Blood Banking Automation. What is ISBT 128? https://web.archive.org/web/20200930085254/https://www.iccbba.org/home/isbt-128-basics/what-is-isbt-128.

224. International Committee Of Medical Journal Editors. Uniform requirements for manuscripts submitted to biomedical journals: Writing and editing for biomedical publication. *Journal of Pharmacology & Pharmacotherapeutics* **1**, 42 (2010).

225. World Association of Medical Editors. World Association of Medical Editors (WAME). http://wame.org/about (2020).

226. BMJ. *Reporting patient and public involvement in research*. (2019).

227. Group, T. S. The SPIRIT Statement. https://www.spirit-statement.org/spirit-statement/ (2020).

228. Therapeutic Goods Administration (TGA). GMP clearance guidance . https://www.tga.gov.au/publication/gmp-clearance-guidance.

229. Dubois, R. W., Lauer, M. & Perfetto, E. When is evidence sufficient for decision-making? A framework for understanding the pace of evidence adoption. **2**, 383–391 (2013).

230. Patient Focused Medicines Development. *Patient Engagement Quality Guidance Tool*. (2018).

231. Synapse. The book of good practices in Patient Engagement. in (2018).

232. Spindler, P. & Lima, B. S. Editorial: The European Patients Academy on Therapeutic Innovation (EUPATI) Guidelines on Patient Involvement in Research and Development. *Frontiers in Medicine* **5**, 310 (2018).

233. Patient Focused Medicine. *Patient Focused Medicines Development Book of Good Practices*. (2018).

234. McPin Foundation. Research Priorities for Children and Young People's Mental Health: Interventions and Services. (2018).

235. Ennis, L. & Wykes, T. Impact of patient involvement in mental health research: longitudinal study. *Br J Psychiatry* **203**, 381–6 (2013).

236. France, E. F. *et al.* Improving reporting of meta-ethnography: The eMERGe reporting guidance. *Journal of Advanced Nursing* **75**, 1126–1139 (2019).

237. Stockholm International Peace Research Institute. Military Expenditure Database. https://www.sipri.org/databases/milex.

238. Stockholm International Peace Research Institute. Arms Industry Database. https://www.sipri.org/databases/armsindustry.

239. Stockholm International Peace Research Institute. Arms Transfers Database. https://www.sipri.org/databases/armstransfers.

240. Stockholm International Peace Research Institute. Multilateral Peace Operations Database. https://www.sipri.org/databases/pko.

241. Center for Open Science. Center for Open Science Mission. https://www.cos.io/about/mission (2020).

242. Haddaway, N. R. Open Synthesis: On the need for evidence synthesis to embrace Open Science. *Environmental Evidence* (2018) doi:10.1186/s13750-018-0140-4.

243. European Union. *Indicator Frameworks for Fostering Open Knowledge Practices in Science and Scholarship*. (2019) doi:10.2777/445286.

244. OCSDNET. Open Science Manifesto. https://ocsdnet.org/manifesto/open-science-manifesto/.

245. European Commission. Open science monitor. https://ec.europa.eu/info/research-and-innovation/strategy/goals-research-and-innovation-policy/open-science/open-science-monitor_en (2020).

246. Zooniverse. Zooniverse. https://www.zooniverse.org/about (2020).

247. Todd, M. H. Six Laws of Open Source Drug Discovery. *ChemMedChem* **14**, 1804–1809 (2019).

248. *Patient Engagement Monitoring and Evaluation Framework with metrics*.

249. Esmail, L., Moore, E. & Rein, A. Evaluating patient and stakeholder engagement in research: Moving from theory to practice. *Journal of Comparative Effectiveness Research* vol. 4 133–145 (2015).

250. OpenID Foundation. *OpenID Self-Certification*. (2015).

251. Parker, L., Fabbri, A., Grundy, Q., Mintzes, B. & Bero, L. “asset exchange” - Interactions between patient groups and pharmaceutical industry: Australian qualitative study. *The BMJ* **367**, (2019).

252. UK Government. Test, Learn, Adapt: Developing Public Policy with Randomised Controlled Trials. (2012).

253. What Works UK. *The Rise of Experimental Government: Cross-Government Trial Advice Panel Update Report*. (2018).

254. Nesta. *Using research evidence: A Practice Guide*. (2016).

255. Gaudet, J. Unfolding the map: Making knowledge and ignorance mobilization dynamics visible in science evaluation and policymaking. in *Routledge International Handbook of Ignorance Studies* (2015). doi:10.4324/9781315867762.

256. Wild, D. *et al.* Principles of good practice for the translation and cultural adaptation process for patient-reported outcomes (PRO) measures: Report of the ISPOR Task Force for Translation and Cultural Adaptation. *Value in Health* **8**, 94–104 (2005).

257. Product Safety Australia. About mandatory standards. https://www.productsafety.gov.au/product-safety-laws/safety-standards-bans/mandatory-standards/about-mandatory-standards#safety-criteria (2020).

258. Michie, S., van Stralen, M. M. & West, R. The behaviour change wheel: A new method for characterising and designing behaviour change interventions. *Implementation Science* **6**, (2011).

259. The Behavioural Insights Team *et al. EAST Four simple ways to apply behavioural insights*. (2014).

260. Kneebone, S., Smith, L. & Fielding, K. The Impact-Likelihood Matrix: A policy tool for behaviour prioritisation. *Environmental Science and Policy* **70**, 9–20 (2017).

261. BehaviourWorks Australia. The BehaviourWorks Method. https://www.behaviourworksaustralia.org/the-method/ (2019).

262. Crossref. Crossref . https://www.crossref.org/ (2020).

263. Committee on Publication Ethics. Committee on Publication Ethics. https://publicationethics.org/about/our-organisation (2020).

264. Committee on Publication Ethics. *Guidance for Editors: research, audit and service evaluations*. (2014).

265. Committee on Publication Ethics. *Principles of Transparency and Best Practice in Scholarly Publishing*. (2014) doi:10.24318/cope.2019.1.12.

266. O'Brien, B. C., Harris, I. B., Beckman, T. J., Reed, D. A. & Cook, D. A. Standards for Reporting Qualitative Research. *Academic Medicine* **89**, 1245–1251 (2014).

267. Tong, A., Sainsbury, P. & Craig, J. Consolidated criteria for reporting qualitative research (COREQ): a 32-item checklist for interviews and focus groups. *International Journal for Quality in Health Care* **19**, 349–357 (2007).

268. Schulz, K. F., Altman, D. G. & Moher, D. coNSorT 2010 Statement: updated guidelines for reporting parallel group randomised trials. *BMJ* **340**, 332 (2010).

269. Department of Environment, Land, Water and Planning, Victorian Government, A. *DELWP Output data standards*.

270. RAID. Research Activity Identifier. https://www.raid.org.au/ (2018).

271. Australian Research Council. *Engagement and Impact Assessment Pilot Report*. (2018).

272. Australian Commission on Safety and Quality in Health Care. *Partnering with Consumers Standards*. (2011).

273. Wilkinson, M. D. *et al.* The FAIR Guiding Principles for scientific data management and stewardship. *Scientific Data* **3**, 160018 (2016).

274. Richard Gold, E. *et al.* Gates Open Research An open toolkit for tracking open science partnership implementation and impact [version 1; peer review: awaiting peer review]. *Diseases of the Developing World* (2019) doi:10.12688/gatesopenres.12958.1.

275. Directory of Open Access Journals. Directory of Open Access Journals. https://doaj.org/about (2020).

276. European Association of Science Editors. EASE Guidelines for Authors and Translators of Scientific Articles to be Published in English. (2018) doi:10.20316/ESE.2018.44.e1.

277. Open Access Scholarly Publishers Association. Open Access Scholarly Publishers Association Mission . https://oaspa.org/about/mission-and-purpose/ (2020).

278. Council of Science Editors. *Promoting Integrity in Scientific Journal Publications - Council of Science Editors*. (2009).

279. Council of Science Editors. *Scientific style and format : the CSE manual for authors, editors, and publishers*.

280. WikiJournal User Group. WikiJournal User Group Ethics statement. https://en.wikiversity.org/wiki/WikiJournal_User_Group/Ethics_statement (2020).

281. Citizen Science Association (USA). About the Public Participation in Scientific Research (PPSR) Core metadata standards repository. https://github.com/CitSciAssoc/DMWG-PPSR-Core/wiki/About-the-PPSR-Core (2020).

282. Tricco, A. C. *et al.* PRISMA extension for scoping reviews (PRISMA-ScR): Checklist and explanation. *Annals of Internal Medicine* vol. 169 467–473 (2018).

283. International Social and Environmental Accreditation and Labelling Alliance. *Assessing the Impacts of Social and Environmental Standards Systems ISEAL Code of Good Practice*. (2014).

284. International Social and Environmental Accreditation and Labelling Alliance. ISEAL is the global membership organisation for credible sustainability standards . https://www.isealalliance.org/ (2020).

285. Social Care Institute for Excellence. *Co-production in social care: What it is and how to do it*. (2015).

286. Brazma, A. *et al.* Minimum information about a microarray experiment (MIAME) - Toward standards for microarray data. *Nature Genetics* vol. 29 365–371 (2001).

287. Forest Stewardship Council. Data Standard Report with International Social and Environmental Accreditation and Labelling Alliance. https://fsc.org/en/innovation/data-standard-report (2020).

288. Tong, A., Flemming, K., McInnes, E., Oliver, S. & Craig, J. Enhancing transparency in reporting the synthesis of qualitative research: ENTREQ. *BMC Medical Research Methodology* **12**, 181 (2012).

289. Wong, G., Greenhalgh, T., Westhorp, G., Buckingham, J. & Pawson, R. RAMESES publication standards: Realist syntheses. *BMC Medicine* **11**, 1–14 (2013).

290. Wong, G. *et al.* RAMESES II reporting standards for realist evaluations. *BMC Medicine* **14**, 1–18 (2016).

291. Moher, D., Liberati, A., Tetzlaff, J. & Altman, D. G. Preferred Reporting Items for Systematic Reviews and Meta-Analyses: The PRISMA Statement. *PLoS Medicine* **6**, e1000097 (2009).

292. Pollock, A. *et al.* Development of the ACTIVE framework to describe stakeholder involvement in systematic reviews. *Journal of Health Services Research & Policy* 135581961984164 (2019) doi:10.1177/1355819619841647.

293. Pollock, A. *et al.* Stakeholder involvement in systematic reviews: a scoping review. *Syst Rev* **7**, (2018).

294. Cochrane. PROGRESS-Plus. https://methods.cochrane.org/equity/projects/evidence-equity/progress-plus.

295. CITES. Checklist of CITES species. https://checklist.cites.org/#/en (2020).

296. Inglehart, R. Mapping global values. *Comparative Sociology* **5**, 115–136 (2006).

297. Větrovský, T. *et al.* GlobalFungi, a global database of fungal occurrences from high-throughput-sequencing metabarcoding studies. *Scientific Data* **7**, 228 (2020).

298. BankTrack. https://www.banktrack.org/.

299. Parr, C. S. *et al.* The Encyclopedia of Life v2: Providing Global Access to Knowledge About Life on Earth. *Biodiversity Data Journal* **2**, e1079 (2014).

300. Our World in Data. https://ourworldindata.org/.

301. OpenWeb Moderation Standards | OpenWeb. https://www.openweb.com/moderation-standards/.

302. Global Anti-Corruption Consortium - Projects - Transparency.org. https://www.transparency.org/en/projects/global-anti-corruption-consortium.

303. Scemama, P. *et al.* Guidance for stakeholder consultation to support national ecosystem services assessment: A case study from French marine assessment. *Ecosystem Services* **54**, 101408 (2022).

304. Presentation DICO | DicoPart. https://www.dicopart.fr/fr/dico/presentation-dico.

305. Kaye, J. *et al.* Including all voices in international data-sharing governance. *Human Genomics* **12**, 13 (2018).

306. Updating The Verge's background policy - The Verge. https://www.theverge.com/press-room/22772113/the-verge-on-background-policy-update.

307. Tong, A. *et al.* Reporting guideline for priority setting of health research (REPRISE). *BMC Medical Research Methodology* **19**, 243 (2019).

308. Alper, B. *et al.* 20 Achieving evidence interoperability in the computer age: setting evidence on FHIR. in *Oral Presentations* A15.1-A15 (BMJ Publishing Group Ltd, 2019). doi:10.1136/bmjebm-2019-EBMLive.28.

309. CKAN - The open source data management system. https://ckan.org/.

310. Health Studies National Data Asset program - ARDC. https://ardc.edu.au/collaborations/strategic-activities/national-data-assets/health-studies-national-data-asset-program/.

311. CDISC | Clear Data. Clear Impact. https://www.cdisc.org/.

312. Scholz, B. & Bevan, A. Toward more mindful reporting of patient and public involvement in healthcare. *Research Involvement and Engagement* **7**, 61 (2021).

313. National Greenhouse and Energy Reporting NGER. http://www.cleanenergyregulator.gov.au/NGER.

314. About | NABERS. https://www.nabers.gov.au/about.

315. Hanel, P. H. P., Maio, G. R. & Manstead, A. S. R. A new way to look at the data: Similarities between groups of people are large and important. *Journal of Personality and Social Psychology* **116**, 541–562 (2019).

316. JATS4R | NISO website. http://www.niso.org/standards-committees/jats4r.

317. Participedia. https://participedia.net/getting-started#.

318. bellingcat - the home of online investigations. https://www.bellingcat.com/.

319. Values and Standards for Patient Involvement in HTA | Health Technology Assessment International (HTAi). https://htai.org/interest-groups/pcig/values-and-standards/.

320. Gani, S. *et al.* Clear, transparent, and timely communication for fair authorship decisions: a practical guide. *Geoscience Communication* **4**, 507–516 (2021).

321. Ellis, U., Kitchin, V. & Vis-Dunbar, M. Identification and Reporting of Patient and Public Partner Authorship on Knowledge Syntheses: Rapid Review. *Journal of Participatory Medicine* **13**, e27141 (2021).

322. Who we are – LBG OIS Center. https://ois.lbg.ac.at/about-us/team-profile/.

323. Trisos, C. H., Auerbach, J. & Katti, M. Decoloniality and anti-oppressive practices for a more ethical ecology. *Nature Ecology & Evolution* **5**, 1205–1212 (2021).

324. Schäfer, T., Kieslinger, B., Mayer, K. & Schürz, S. CoActD7.1: Impact Assessment Plan. (2020) doi:10.5281/ZENODO.4443470.

325. Staniszewska, S. *et al.* Developing a Framework for Public Involvement in Mathematical and Economic Modelling: Bringing New Dynamism to Vaccination Policy Recommendations. *The Patient - Patient-Centered Outcomes Research* **14**, 435–445 (2021).

326. Golub, A., Herrera, D., Leslie, G., Pietracci, B. & Lubowski, R. A real options framework for reducing emissions from deforestation: Reconciling short-term incentives with long-term benefits from conservation and agricultural intensification. *Ecosystem Services* **49**, 101275 (2021).

327. Salignac, F., Wilcox, T., Marjolin, A. & Adams, S. Understanding Collective Impact in Australia: A new approach to interorganizational collaboration. *Australian Journal of Management* **43**, 91–110 (2018).

328. Homepage | CISAC. https://www.cisac.org/.

329. Gerber, K. *et al.* Ethical Concerns about Psilocybin Intellectual Property. *ACS Pharmacology & Translational Science* **4**, 573–577 (2021).

330. The world doesn't just need cotton, it needs Better Cotton. https://bettercotton.org/.

331. Citizen Science | CS Track European project. https://cstrack.eu/.

332. HTA Core Model 3.0 - EUnetHTA. https://www.eunethta.eu/hta-core-model-3-0/.

333. Charity Register innovation to showcase charities' work | ACNC. https://www.acnc.gov.au/media/news/charity-register-innovation-showcase-charities-work.

334. Ceccaroni, L. *et al.* Opportunities and Risks for Citizen Science in the Age of Artificial Intelligence. *Citizen Science: Theory and Practice* **4**, (2019).

335. How we're using AI to scale up global fact checking - Full Fact. https://fullfact.org/blog/2020/jul/afc-global/.

336. FSC® launches new global brand: Forests For All Forever | Forest Stewardship Council. https://fsc.org/en/newsfeed/fscr-launches-new-global-brand-forests-for-all-forever.

337. Personal Genome Projects: Global Network. https://www.personalgenomes.org/.

338. GeoBlacklight. https://geoblacklight.org/.

339. PPSR_CORE Metadata Standard - Citizen Science AssociationNews |. https://citizenscience.org/2015/10/09/ppsr_core-metadata-standard/.

340. Department for Digital, Culture, Media & Sport - GOV.UK. https://www.gov.uk/government/organisations/department-for-digital-culture-media-sport.

341. Schema.org - Schema.org. https://schema.org/.

342. Home - DCAT. https://dcat.org/.

343. Wikidata:Property proposal/PeriodO period ID - Wikidata. https://www.wikidata.org/wiki/Wikidata:Property_proposal/PeriodO_period_ID.

344. Data Catalog Vocabulary (DCAT) - Version 2. https://www.w3.org/TR/vocab-dcat-2/.

345. DCMI: Home. https://www.dublincore.org/.

346. DCMI: DCMI Metadata Terms. https://www.dublincore.org/specifications/dublin-core/dcmi-terms/.

347. SPUN. https://spun.earth/.

348. The Home of Location Technology Innovation and Collaboration | OGC. https://www.ogc.org/.

349. PLANETARY HEALTH - Planetary Health Alliance. https://www.planetaryhealthalliance.org/planetary-health.

350. SciStarter - SciStarter. https://scistarter.org/.

351. ACSA's Project Finder – Australian Citizen Science Association. https://citizenscience.org.au/ala-project-finder/.

352. An Ecosystem of Citizen Observatories for Environmental Monitoring - weobserve. https://www.weobserve.eu/.

353. Data and Metadata Working Group - Citizen Science Association. https://citizenscience.org/get-involved/working-groups/data-and-metadata-working-group/.

354. PPSR Core | PPSR Core. https://core.citizenscience.org/.

355. Department of Education, Skills and Employment. https://www.dese.gov.au/.

356. Experience Based Co-Design Toolkit | Australian Healthcare & Hospitals Association. https://ahha.asn.au/experience-based-co-design-toolkit.

357. OpenSC - Home. https://opensc.org/.

358. Features: Online Community Engagement Tool | CitizenLab. https://www.citizenlab.co/platform-online-engagement-toolbox.

359. Citizen Science Observatory in Spain. https://ciencia-ciudadana.es/.

360. Home Page - RRI Tools. https://rri-tools.eu/.

361. Citizen Science Projects - Österreich forscht. https://www.citizen-science.at/en/.

362. Medborgarforskning.se. https://medborgarforskning.se/.

363. Αρχική | Paratiro.gr - Εθνικό Δίκτυο Παρατηρητών Άγριας Ζωής. https://www.paratiro.gr/.

364. Home - Citizen Science. http://www.citizenscience.cz/.

365. Just One Giant Lab - Learning and Solving Together - JOGL. https://jogl.io/.

366. Diener, E. *et al.* New Measures of Well-Being. in 247–266 (2009). doi:10.1007/978-90-481-2354-4_12.

367. Grant, M. J. & Booth, A. A typology of reviews: an analysis of 14 review types and associated methodologies. *Health Information & Libraries Journal* **26**, 91–108 (2009).

368. Greenhalgh, T. *et al.* Beyond Adoption: A New Framework for Theorizing and Evaluating Nonadoption, Abandonment, and Challenges to the Scale-Up, Spread, and Sustainability of Health and Care Technologies. *Journal of Medical Internet Research* **19**, e367 (2017).

369. Core Values, Ethics, Spectrum – The 3 Pillars of Public Participation - International Association for Public Participation. https://www.iap2.org/page/pillars.

370. Taylor, J., Kukutai, T. & Australian National University. Centre for Aboriginal Economic Policy Research. Indigenous data sovereignty : toward an agenda. 318.

371. Moher, D. *et al.* The Hong Kong Principles for assessing researchers: Fostering research integrity. *PLOS Biology* **18**, e3000737 (2020).

372. Peterson, A., Charles, V., Yeung, D. & Coyle, K. The Health Equity Framework: A Science- and Justice-Based Model for Public Health Researchers and Practitioners. *Health Promotion Practice* **22**, 741–746 (2021).

373. Spiers, H. *et al.* Everyone counts? Design considerations in online citizen science. *Journal of Science Communication* **18**, A04 (2019).

374. EBCD: Experience-based co-design toolkit - Point of Care Foundation. https://www.pointofcarefoundation.org.uk/resource/experience-based-co-design-ebcd-toolkit/.

375. Walter, M., Kukutai, T., Carroll, S. R. & Rodriguez-Lonebear, D. *Indigenous Data Sovereignty and Policy*. (Routledge, 2020). doi:10.4324/9780429273957.

376. Prainsack, B. The 'citizen science' of genetics. in *Genetics as Social Practice* (eds. Prainsack, B., Werner-Felmayer, G. & Schicktanz, S.) vol. 1 147–164 (Farnham: Ashgate, 2014).

377. Traditional Knowledge Licenses – Local Contexts. https://localcontexts.org/licenses/traditional-knowledge-licenses/.

378. The Australian Survey of Social Attitudes | ACSPRI. https://www.acspri.org.au/aussa.

379. LifeWatch ERIC Metadata Catalogue. https://metadatacatalogue.lifewatch.eu/srv/eng/catalog.search#/metadata/55dfbb63-4304-4fa8-a7b6-17988e0b33df.

380. DNA Learning Center Barcoding 101. https://dnabarcoding101.org/.

381. Pacific iCLIM. https://www.griffith.edu.au/research/research-excellence/griffith-climate-change-response-program/pacific-iclim.

382. About | CISAC. https://www.cisac.org/about.

383. OSF | A Standard Taxonomy for Peer Review. https://osf.io/68rnz/.

384. Heigl, F. *et al.* Co-Creating and Implementing Quality Criteria for Citizen Science. *Citizen Science: Theory and Practice* **5**, (2020).

385. Global Humanitarian Operational Presence. https://3w.unocha.org/.

386. The Frozen Ark. https://web.archive.org/web/20220218140847/https://www.frozenark.org/.

387. Australian Frozen Zoo. https://www.australianfrozenzoo.org.au/.

388. EITI Standard 2019 | EITI. https://eiti.org/collections/eiti-standard.

389. LF Energy - LF Energy. https://www.lfenergy.org/.

390. Kumar, A. *et al.* DCMS: A data analytics and management system for molecular simulation. *Journal of Big Data 2014 2:1* **2**, 1–22 (2014).

391. GitHub - BTAA-Geospatial-Data-Project/GBL-Schema-Update. https://github.com/BTAA-Geospatial-Data-Project/GBL-Schema-Update.

392. Goldacre, B. & Gray, J. OpenTrials: towards a collaborative open database of all available information on all clinical trials. *Trials* **17**, 164 (2016).

393. Department of Education, Skills and Employment. https://www.dese.gov.au/.

394. GitHub - EBISPOT/DUO: Ontology for consent codes and data use requirements. https://github.com/EBISPOT/DUO.

395. ASI Home - Aluminium Stewardship Initiative. https://aluminium-stewardship.org/.

396. BMC. Research Involvement and Engagement - Reporting standards. *Research Involvement and Engagement* https://researchinvolvement.biomedcentral.com/submission-guidelines/preparing-your-manuscript/research (2019).

397. Aitken, M. *et al.* Consensus Statement on Public Involvement and Engagement with Data-Intensive Health Research. *Under review* **4**, (2018).

398. Regan de Bere, S. & Nunn, S. Towards a pedagogy for patient and public involvement in medical education. *Med Educ* **50**, 79–92 (2016).

399. International Association for Public Participation. Participation Spectrum. https://www.iap2.org.au/Tenant/C0000004/00000001/files/IAP2_Public_Participation_Spectrum.pdf (2014).

400. Nunn, J. S., Scott, C. L., Stubbs, J. W., Cherry, S. F. & Bismark, M. M. Involving the Public in Rare Cancer Care and Research. *Textbook of Uncommon Cancer* (2017) doi:10.1002/9781119196235.ch3.

401. Stickley, Theo. & Basset, Thurstine. *Learning about Mental Health Practice*. *Learning about Mental Health Practice* (Wiley, 2008). doi:10.1002/9780470699300.

402. Alison Faulkner. *National Involvement Standards Involvement for influence*. (2015).

403. Hancock, T. & Bezold, C. Possible Futures, Preferebale Futures. *Healthcare Forum Journal* 23–29 (1994).

404. Bergold, J. & Stefan, T. Participatory Research Methods : A Methodological Approach in Motion. *Forum: Qualitative Social Research* **13**, Art. 1 (2012).

405. Gretta Pecl, Chris Gillies, Carla Sbrocchi & Philip Roetman. *Building Australia Through Citizen Science*. (2015).

406. Brett, J. *et al.* The PIRICOM Study : A systematic review of the conceptualisation, measurement, impact and outcomes of patients and public involvement in health and social care research. 1–292 (2010).

This is the end of the document.

For more up to date information visit: ScienceForAll.World/STARDIT
