# Supplementary material for: Standardised data on initiatives—STARDIT: Beta version
Source: Res Involv Engagem. 2022 Jul 19;8:31. doi: 10.1186/s40900-022-00363-9 (PMC9294764; doi:10.1186/s40900-022-00363-9)
Supplement: Supplementary file 2 — Additional file 2. This document contains a STARDIT Beta version report about the co-creation process of the STARDIT Beta version. [file 40900_2022_363_MOESM2_ESM.pdf]

Standardised Data on Initiatives (STARDIT) report: A report about the article 'Standardised Data on Initiatives – STARDIT: Beta Version'

This document contains additional information relevant to the article 'Standardised Data on Initiatives (STARDIT) Beta Version'. This document contains a STARDIT report about this article, and uses the Beta version of the STARDIT report as described in the associated paper: https://doi.org/10.1186/s40900-022-00363-9

The 'living' version of this STARDIT report can be found here: https://wikispore.wmflabs.org/wiki/STARDIT_Beta_Version_co-creation_process

**Identifying information**	
*Initiative name	Standardised Data on Initiatives – STARDIT: Beta Version
*Geographic location(s)	International
Date range (start and end dates of initiative)	Start: September 20th 2019<br>End: August 27th 2021
Purpose of the initiative (aims, objectives, goals) *	The objective of STARDIT is to address current limitations and inconsistencies in sharing data about initiatives. The STARDIT system features standardised data reporting about initiatives, including who has been involved, what tasks they did, and any impacts observed. STARDIT is designed to support a culture of partnership across disciplines and is, wherever possible, aligned and interoperable with existing reporting models and frameworks such as those used in health, environment, manufacturing , publishing, government policy, education, arts and international development (see Table 1). In addition, the STARDIT Preference Mapping (STARDIT-PM) tool provides a standardised way to report information about different stakeholders' preferences, including preferences for power-sharing and methods of involving people during an initiative. The working Beta Version of STARDIT uses Wikidata (collaboratively edited structured data) to enable definitions to be co-created by contributors anywhere in the world, and therefore works across human languages, with interoperability with other platforms planned for future versions.
*Organisations or other initiatives involved (list all if multi-centre)	Individuals working for or with the following organisations are co-authors on this article:<br>Science for All (Education Charity registered in Australia<br>School of Life Sciences, La Trobe University, Victoria, Australia

	La Trobe University, Victoria, Australia<br>Patient Advocate, Co-Editor-in-Chief, 'Research Involvement and Engagement'<br>Health Research Authority (England)<br>UCL Institute of Education<br>Adjunct Assistant Professor, Amrita Institute of Medical Sciences & Research Centre, Kochi, Kerala, India<br>Cochrane<br>Department of Linguistics, Faculty of Medicine, Health and Human Sciences, Macquarie University<br>Poche Centre Indigenous Health, The University of Sydney<br>University of Liverpool<br>University of Melbourne<br>Taylor and Francis<br>Consumers Health Forum of Australia<br>European Organisation for Rare Diseases<br>Applied Ecology and Environmental Change Research Group, Institute for Sustainable Industries and Liveable Cities, Victoria University, Melbourne, Australia.<br>Australian Citizen Science Association<br>University of Sydney<br>The Poche Centre for Indigenous Health, Faculty of Medicine and Health, The University of Sydney, NSW 2006, Australia<br>School of Population Health, Faculty of Medicine and Health, University of New South Wales, Sydney 2052, Australia;<br>Faculty of Medicine, Health and Human Sciences, Macquarie University, Sydney 2109, Australia<br>Co-Labs Melbourne<br>Norwegian University of Science and Technology (NTNU)<br>University of Bath, United Kingdom<br>MammalWeb project<br>Johns Hopkins University<br>Institute of Zoology, Zoological Society of London
*Funding information (link to publicly accessible URL if possible) - *this may include the project funder or,*	Science for All has provided pro bono support for Jack Nunn to lead the co-design process and provided funding ($500) for a developer to build the working STARDIT Beta version online reporting tool.

<table>
<tr><td>funding agreements, grants or donations</td><td colspan="2">The STARDIT project received in kind support from the EPPI-Centre, which provided a free meeting room and refreshments.

Dave McCall worked with Jack Nunn and led the design of the STARDIT logos, artwork and brand guide and provided his time and skills pro-bono.</td></tr>
<tr><td>*Ethics approval information (if applicable)</td><td colspan="2">Not applicable</td></tr>
<tr><td rowspan="2">Relevant publicly accessible URLs/URIs (for example, website, clinical trial registration details if applicable)*</td><td>Link: https://scienceforall.world/stardit/beta/</td><td>Description: Section of Science for All website explaining STARDIT</td></tr>
<tr><td>Link: https://doi.org/10.31219/osf.io/w5xj6</td><td>Description: Pre-print version of STARDIT Beta before submission to journal ‘Research Involvement and Engagement’, including Supplementary Resources</td></tr>
<tr><td>Other relevant information (free text)</td><td colspan="2">A multi-disciplinary international team of over 100 citizens, experts and data-users has been involved in co-creating STARDIT to help everyone in the world share, find and understand information about collective human actions, which are referred to as ‘initiatives’.

STARDIT is an open access data-sharing system to standardise the way that information about initiatives is reported, including information about which tasks were done by different people. Reports can be updated at all stages, from planning to evaluation, and can report impacts in many languages, using Wikidata. STARDIT is free to use, and data can be submitted by anyone. Report authors can be verified to improve trust and transparency, and data checked for quality.

STARDIT can help create high-quality standardised information on initiatives trying to solve complex multidisciplinary global problems. Among its main benefits, STARDIT offers those carrying out research and interventions access to standardised information which enables well-founded comparisons of the effectiveness of different methods. This article outlines progress to date; information about submitting reports; planned next steps and how anyone can become involved.</td></tr>
<tr><td>RAiD (API) - identifier for research activities (if applicable)</td><td colspan="2">Not applicable</td></tr>
<tr><td>Keywords or metatags – including relevant search</td><td colspan="2">Data, open, standardised, participatory, democracy, evidence, systematic, genomics, health, indigenous</td></tr>
</table>

headings (e.g. Medical Subject Headings - MeSH)	
**State of initiative**	
* What is the current state of the initiative or research project? Select from:<br>1. **Before** the initiative– this report is prospective or describes planned activity<br>2. **Ongoing** – the initiative is still taking place<br>3. **After** the initiative has occurred	After
**Methods and paradigms**	
*Methods of the initiative (what is planned to be done, or is being reported as done). Include information about any populations or eco-systems being studied, any 'interventions', comparators and outcome measures (qualitative or quantitative).<br><br>If appropriate, include a link to a publicly accessible document (such as a research protocol or project plan).	Participatory action research:<br><br>STARDIT development is guided by participatory action research (PAR) paradigms, which guides initiatives by aiming to involve all stakeholders in every aspect of the development and evaluation of an initiative[1,2]. Participatory research is a form of collective, self-reflective enquiry undertaken by people in order to understand their situation from different perspectives[3]. Development has also been influenced by existing work in health research, including the multidisciplinary area of public health, which incorporates social, environmental and economic research. In a health context, participatory research attempts to reduce health inequalities by supporting people to be involved in addressing health issues that are important to them, data collection, reflection and ultimately in action to improve their own health[4]. At the core of participatory research is 'critical reflexivity'. The process asks people involved to reflect on the causes of problems, possible solutions, take any actions required which might improve the current situation, and evaluate the actions[2].<br><br>Rights-based paradigm:<br><br>The United Nations (UN) Universal Declaration Human Rights states everyone should be able to 'receive and impart information and ideas'[5]. The UN also states that democracy, development and respect for all human rights and fundamental freedoms are interdependent and mutually reinforcing'[6]. To uphold human rights and 'environmental rights'[7], and for 'the maintenance of peace', people require 'media freedom' in order to 'seek, receive and impart information'[6], free of unaccountable censorship. STARDIT

has been created in order to help anyone uphold these universal rights, by providing a way to share open access information in a structured way with a transparent process for quality checking.

Cultural neutrality:

Values, assumptions, ways of thinking and knowing are not shared universally. The participatory process used for developing STARDIT required and will continue to require that it attempts to map cultural variations, in an attempt to avoid unconsciously reinforcing particular (often 'dominant')[8] values. Transparent acknowledgement of differing values and perspectives is critically important, in particular when mapping if different stakeholders' values are complimentary or opposing. A participatory process requires mapping all of these perspectives and, where possible, involving people in labelling different perspectives and values. For example, STARDIT has already been used to map the varying perspectives of multiple stakeholders when planning a multi-generational cohort study[9].

Many problems facing humans are shared by non-human life forms and ecosystems, including rapid climate change, air pollution and sea-level rise. If initiatives are to operate in inclusive, culturally-neutral ways, reconsideration of the language used to describe relationships between humans, non-human life and the environment is essential.[10] Environmental and social sciences are challenging and redefining colonial-era concepts of what can be 'owned' as property or who 'owns' [10,11]. As a result, ecosystems such as rivers and non-human animals, are being assigned 'personhood'[12–14]. For example, a public consultation by a 'dominant' group might ask, 'who owns the rights to the water in a river system?'[8] This question imposes the dominant group's values on people who may not share the same concept of 'ownership'. In this way, Western European legal and economic traditions are frequently incompatible with those of some Indigenous peoples'.[10,15,16]

The participatory process used for developing STARDIT has attempted to be transparent about how different stakeholders have been involved in shaping it in order to improve how the system can be used to map values and provide more culturally neutral guidance for planning and evaluating involvement in initiatives. However, it is acknowledged that it will be a challenging process to 'de-colonialise' and 'de-anthropocise' language and action[17,18], as this may be perceived as a challenge to some people's cultural attitudes which may not align with the United Nation's universally enshrined principles of democracy, human rights and environmental rights. In addition, ongoing co-

	design will be required to ensure STARDIT is as accessible and inclusive as possible.
Include any information about theoretical or conceptual models or relevant ‘values’ of people involved with this initiative, including any rationale for why certain methods were chosen	What are the values of STARDIT?<br><br>As STARDIT is a new initiative, we have adopted the values of Science for All[9], the organisation which is hosting the participatory action research process.<br><br>Summary of values<br><br>1. We are not-for-profit – our motivation is not profit but working for all life on earth<br>2. We value the freedom to ask any question, using the scientific method wherever possible<br>3. We support the principles of democracy, the ‘rule of law’ and evidence-informed policy wherever we can<br>4. We are transparent and accountable<br>5. We include as many people as we can, as best we can in every aspect of our work<br>6. We know that science means knowledge, and knowledge takes many forms<br><br>STARDIT values:<br><br>**1. We are not-for-profit – working for all life on earth**<br>● We value outcomes that everyone is involved in creating – including ones which could be measured in biodiversity, happiness indexes and often – are currently immeasurable.<br>● We recognise the need to be inclusive, and therefore, to pay people for their time, skills and expertise. When we work in economic structures, we do so only to support our values, not to make profit at the expense of others.<br>● We do not seek to impose our values (or other values such as economic outcome measures) on any life on earth.<br>● We value human rights, but also note that ‘rights’ don’t start and end with humans – we seek to explore what ‘working for all life on earth’ means in any way possible.<br>**2. We value the freedom to ask any question, using the scientific method wherever possible**<br>● Asking a question can be the most simple, most profound or the most complex thing we can do as a species. Questions like ‘why?’ and ‘why not?’ are powerful.

- The freedom to ask the questions is essential, and we hope to support people everywhere to ask any question they feel is important.
- Some questions can be answered using the ‘scientific method’ – which essentially seeks to build our knowledge by asking questions, making observations about the available reality and using this to make predictions. If the predictions can be tested – or are repeatable – others can verify results. We value this method of asking questions, ‘peer review’, sharing open data and results – new knowledge, for free, for everyone.
- We recognise the limits of the scientific method and that some things can never be ‘known’ or ‘peer reviewed’.

3. **We support the principles of democracy, the ‘rule of law’ and evidence-informed policy wherever we can**
    - We support the United Nation’s statement on democracy, including that ‘democracy is a universal value’ and that the principles of ‘democracy, development and respect for all human rights and fundamental freedoms are interdependent and mutually reinforcing’[1].
    - We also accept that there is no single model of democracy and that democracy does not belong to any country or region. To uphold human rights and for ‘the maintenance of peace’, we agree that people require ‘media freedom’ in order to ‘seek, receive and impart information’[9]
    - We support decisions and policies which are informed by evidence, preferably created from open data, shared freely and peer-reviewed by those who are collectively defined as experts. We do not support decisions or policy which appears to be informed by real or perceived conflicting or competing interests. We do not align with any political ‘parties’ or ideologies, and will only work with elected representatives to help uphold the values articulated in this document.
    - We recognise ‘law’ and ‘lore’ as forms of knowledge which must evolve and interact with reality and language. We support universal access to this kind of knowledge. We believe the purpose and enforcement of these laws must be collectively decided in the interests of all life on earth. We support the principle of the rule of law, which implies that every person is

[1] http://www.un.org/en/ga/search/view_doc.asp?symbol=A/RES/64/155

subject to the law, including people who are lawmakers, law enforcement officials, elected representatives and judges. However, we recognise that that are often multiple, sometimes competing or conflicting systems of ‘law’ and ‘lore’, and concepts of ‘property’. We will always be guided transparently and collectively when navigating any conflicts or competing interests.

4. **We are transparent and accountable**
   - We support transparent decision making, reporting and evaluation.
   - We aspire to be as transparent as possible. We will always publicly disclose any funding or ‘in kind’ donations. Partnership with organisations will always align with these values.
   - We recognise that real or perceived conflicting or competing interests can damage trust, and we will do everything we can to avoid any doubt in regards to the motives of our actions being anything other than aligned with our values.
   - We accept that some things may have to be confidential (for example, storing confidential personal information to align with privacy laws) and we will always work transparently, inviting everyone to be involved in helping us get the balance right between privacy and transparency as the world evolves with new technologies.
   - We expect and encourage others to hold us to account, to make sure we do what we say we do, and act as we say we do. Transparency is the best tool to enable this.
   - We will always try to understand the effect or impact of our actions – and work openly with anyone who can help us improve how we are measuring this. This will help ensure we are doing things the best way we can.
5. **We include everyone we can as best we can in every aspect of our work**
   - ‘Organisations’ are people working together towards a shared goal or purpose – and everyone is welcome to get involved.
   - We will be transparent about who is involved, how we are supporting people to stay involved, how we are working to involve new people and will always welcome ideas about how we can improve this.
   - Ensuring we are inclusive and do not knowingly ‘exclude’ anyone is central to our way of working
6. **We know that science means knowledge, and knowledge takes many forms**

- We recognise that knowledge takes many forms – this includes people who are subject area experts, people with personal experience, people with traditional, indigenous or local knowledge, artists – and those with big dreams and big ideas
- Linguistic labels like ‘knowledge’, ‘skills’, ‘expert’, ‘artist’, ‘patient’ and ‘citizen’ can be both helpful and unhelpful. Wherever possible we will include people in helping us define what we mean when we use these words.
- Some things are knowable, some things are not – we work to explore the limits of what knowledge means – with everyone, for all life on earth.

Additional values and paradigms:

In addition to Science for All’s values, the following values are specific to the STARDIT initiative:

- STARDIT is **system and language agnostic**, it should always be designed to work across and with as many systems as possible, in as many countries and languages as possible

- STARDIT **designs and code should always be open access** and relevant licenses should always be those which allow others to build on and improve the project, while maintain central control over quality

- STARDIT **development will be guided by the participatory action research (PAR) paradigm**[10]. PAR is an umbrella term which describes a number of related approaches, including [11(p1)], community-based participatory research, participatory action research (including critical participatory action research), participatory health research, community-partnered participatory research, cooperative inquiry. It may also include other forms of action research embracing a participatory philosophy which may include ‘co-design’ of research and other kinds of research which might include forms of ‘public involvement’ (or sometimes ‘engagement’).

- STARDIT will be guided by the **United Nations rights-based paradigm,** including human rights, environmental rights and other emerging rights

Immutable values

While these values will evolve, we will keep an immutable record of our values. They will always be shared via a publicly accessible URL and regularly archived on the ‘Internet Archive’ for future reference[12].

	Our values will be reviewed annually – with a process for involving the public, with final approval going to a vote in the Steering Committee. This review process may change over time but will always require and invite specialist, expert and public scrutiny, according to the transparent governance principles described in the manual.<br><br>STARDIT must be implemented in a way which encourages those involved to acknowledge cultural values and assumptions in a transparent way. For example, some people can be labelled as having human-centred (anthropocentric) values, which values natural resources in relation to benefits they can provide for humans. In contrast, some people who think the value of nature should be measured using non-human outcomes can be labelled ecocentric[13]. A participatory process requires mapping all of these perspectives and, where possible, labelling them.
**Report Authorship**	
*Name	Jack Nunn
*Publicly accessible profiles, institutional pages	https://scholars.latrobe.edu.au/j2nunn
*Open Researcher and Contributor ID (orcid.org)	https://orcid.org/0000-0003-0316-3254
Tasks in report completion	Wrote STARDIT report
Other information	
*Key contact at initiative for confirming report content (include institutional email address)	Jack.Nunn@ScienceForAll.World
Date of report submission *(automatically generated)*	2021.08.27
*Who was involved or how would you label those involved (select from group labels or submit new group label name in free-text) *You can name individuals or use 'labels' to describe groups of people such as 'professional researchers', 'service users' or 'research participants'. Additional 'labels' or 'meta-*	1. Lead author<br><br>2. Authors<br><br>3. Formally acknowledged project supporters<br><br>4. People involved in public events or discussions (including online discussions on STARDIT forums) and public consultations which informed STARDIT

tags' to describe people may be added if appropriate.	
How many people were in each grouping label?	1. 1<br>2. 24<br>3. 18<br>4. 50+ (estimate)
*Tasks of this person or group (list as many as possible) – *including any information about why certain people were included or excluded in certain tasks (such as data analysis)*	1. Came up with concept of STARDIT, initiated project and co-design process, named STARDIT, co-created logo, lead co-creation process, wrote article and co-ordinated feedback from co-atuhors and public consultation<br>2. Commented on STARDIT manuscripts (Alpha and/or Beta versions), attended public events, commented in discussions, attended meeting with Jack Nunn about STARDIT<br>3. Informed STARDIT creation, but did not comment on manuscript versions. Attended public events, commented in discussions, attended meeting with Jack Nunn about STARDIT<br>4. Attended public events, commented in discussions, attended meeting with Jack Nunn about STARDIT
Method of doing task? How did these people complete these tasks? (what methods were used) – *for example 'group discussion' or 'reviewing documents'*	1-4: Formal and informal online meetings, group and individual informal and formal face to face meetings, online facilitated text-based asynchronous discussions, online voting and decision making tools, commenting on documents, completing online surveys, email discussions, informal discussions around a campfire at 'Campfires and Science' events.
Communication modes? What modes of communication were used – *for example, 'group video calls', 'telephone interviews' or 'postal survey'*	1-4: group video calls, face to face meetings, online text based discussions, online surveys
How were people recruited, contacted or informed about these tasks?	1-4: social media, emails, contacting known people in networks ('snowballing' method of asking people if they know anyone who might be interested), public events, webinars, 'Campfires and Science' events
**Involvement appraisal**	
Methods of appraising and analysing involvement (assessing rigour, deciding	Self-assessment report, planned survey of people involved in co-design.

outcome measures, data collection and analysis)	
Enablers of involvement (what do you expect will help these people get involved – or what helped them get involved)	Working with partner organisations where people are paid for their time, attending meetings and webinars already organised (rather than setting up STARDIT specific events), informal discussions with different communities of shared interest
Barriers of involvement (what do you expect will inhibit these people from getting involved – or what inhibited them from getting involved). Are there any known equity issues which may contribute?	People were not paid for their time while being involved (except for one developer), discussions and documentation were in English language only
How did the initiative change as a result of involving people (did the design or evaluation change?)	Multiple changes were made as a result of involving people, including changing the name and the logo, multiple changes to the article text (Alpha and Beta) and the STARDIT reporting tool.
*Were there any outcomes, impacts or outputs from people being involved? *When describing these, attempt to label which groupings were affected and how. These can include impacts on people, organisations, processes or other kinds of impacts.*	The lead author (Jack Nunn) and the second author (Thomas Shafee) agreed that involving people in multiple ways at multiple stages improved the quality of the STARDIT project, made the reporting tool more relevant to multiple disciplines and improved the language used to describe the project.
What worked well, what could have been improved? Was anything learned from the process of involving these people?	Online discussions and voting tools were an efficient way to make collective decisions.<br>More face to face events were planned for other countries, but this was not possible owing to COVID.<br>Commenting on a shared online document was an efficient way to get feedback from multiple authors simultaneously. An online form was a efficient way of collecting other information and longform feedback, compared to via email.
Which stage of the initiative were these people involved? *(please provide information about any distinct stages of*	1-4: All stages

this initiative, noting some may overlap)	
What was the estimated financial cost for involving people. How much time did it take. Were there any costs that cannot be measured financially?	Estimated financial cost of involving people (not including pro-bono time of Jack Nunn): $2000 AUD (venues, catering, online tools and licenses)<br><br>Estimated financial cost of involving people including pro-bono time of Jack Nunn: $50,000 AUD (500 pro-bono hours over 3 years, if paid at Science for All's rate)<br><br>The time of the co-authors, project supporters and other people involved was not paid and is difficult to estimate. If the average time of each person involved (>74) is put at 4.7 hours, then it is 348 hours of unpaid work.
Were there other resources needed aside from time and money (and if so what were they)?	Online tools (such as Loomio, Google Workspace, Zoom) were all used and had costs associated with them that were paid for by Science for All, or required expertise to facilitate, that was provided by Science for All staff.<br><br>Online facilitation was provided by Jack Nunn.
What was the estimated financial cost for the initiative. How much time did it take. Were there any costs that cannot be measured financially (eg. volunteer time)?	Cost: $66,800 AUD<br><br>Time: 848 hours of volunteer time over 3 years
**Mapping financial or other 'interests'**	
*Describe any financial relationship or other interests anyone involved with this project might have	Jack Nunn is the Director of Science for All (a voluntary unpaid position), a PhD candidate at the University of La Trobe (where he received a scholarship from 2016-2020) and STARDIT reports and the STARDIT Beta version article will feature as elements in Jack's PhD thesis (in-progress). Jack is a member of the Cochrane Council (a voluntary unpaid position), the Strategy Liaison for the Wiki Journals (a voluntary unpaid position), a member of the Australian Federal Departments of Health's Medical Services Advisory Committee Evaluation Sub-committee (where he is paid for attending meetings and associated work). Jack is on the Editorial Board for the Wiki Journal of Science, the Wiki Journal of Humanities and 'Research Involvement and Engagement'. A discount of the 'Article Processing Fee' is offered to 'Research Involvement and Engagement' Editorial Board members, and this was utilised to be able to afford publishing in this journal, as there was no project budget for publication.

	Thomas Shafee is the Editor-in-Chief of the Wiki Journals (a voluntary unpaid position). In order to be inclusive (and not exploitative), Science for All paid Thomas Shafee to build a working Beta of the STARDIT form (after a public tender and transparent selection process).<br>Science for All occasionally pays Jack Nunn to work on other projects (overseen by the Science for All Steering Committee and reported transparently), as described in the 'Ways of Working' document, but has not paid Jack to work on STARDIT.<br>No other authors have declared any financial or other interests.
*Describe any conflicting or competing interests (including any relevant information about authors of this report), or any other 'interests', including personal interest or (for example, how you may be personally or professionally affected by the outcome of the initiative).	All authors may benefit financially from being involved in this project, as their familiarity with the project may mean individuals and organisations wish to remunerate them for their time and expertise in relation to applying and using the STARDIT tools.<br>Some authors may receive funding to their employing organisations for being a co-author.<br>All authors may benefit reputationally from being involved in this project, although this is a prospective outcome and the opposite may happen (!)
**Data (including code, hardware designs or other relevant information)**	
*Who is the data from this initiative shared with?	Publicly accessible
*How is it stored and hosted?	Research Involvement and Engagement journal servers, Wikimedia servers, Internet Archive
Who is involved in analysing the data? (align with terms used to describe who is involved)	Jack Nunn analysed all feedback from the public consultation
What methods will be used to analyse the data (including a link to any relevant code and information about validity)	Thematic analysis and other methods of analysis
*How is information about this data disseminated?	Social media, newsletters, podcasts, webinars, videos
Who 'owns' the data or claims any kind of 'intellectual property' or	Science for All<br>Science for All is a charity registered in Australia<br>(ABN: 37636063351 ACN: 636063351).

rights (include relevant licensing information)	
Who controls access to the data, how are decisions about data access made? Is data anonymised or de-identified? What methods are used for re-identification? What is the risk of unauthorised re-identification?	Science for All controls access to public consultation data, relevant emails, mailing lists of relevant people and online discussion data.
Who manages (or ’curates’) the data?	STARDIT data is currently curated by the Wiki Journal of Science Editorial Board.
Who is accountable for ensuring the quality and integrity of the data? (this may be an individual or organisation)	The Wiki Journal of Science Editorial Board is currently accountable for ensuring the quality and integrity of the STARDIT data.
*How is/will the data be ‘Findable, Accessible, Interoperable, Reusable’ according to the FAIR criteria? (provide a link to a publicly accessible URL if appropriate)	Findable: Publicly accessible reports and websites<br>Accessible, Interoperable and reusable: Structured data used to describe the project
If any data relates to Indigenous peoples, describe how data governance aligns with the CARE Principles for Indigenous Data Governance.	Indigenous people involved with this project had control over what was disclosed about their identity and their involvement.
**Impacts and outcomes**	
*What new knowledge has been generated? (if appropriate, include effect size, relevant statistics and level or evidence)	STARDIT is a tool which has applications across multiple disciplines.
Describe how the learning or knowledge generated from this initiative has or will be used	Learning from the STARDIT Beta will be applied to the development of Version One.
*Has anything changed or happened as a result of this	A new data standard which is interoperable with others has been created. A working Beta version has been built. STARDIT reports

initiative that isn't captured in previous answers?	have been submitted and exist for a number of peer-reviewed articles. STARDIT is being adopted by other organisations to use.
How has or how will this be measured or evaluated?	Informal feedback from people involved in co-design, or people using STARDIT
Who is involved in measuring or evaluating this?	Science for All staff
Who was or is involved in deciding on the outcomes used to evaluate any impacts or outcomes? How were they involved?	No specific outcome measures have been decided other than 'number of reports completed'.

References

1. Cook T, Abma T, Gibbs L, et al. *Position Paper No. 3: Impact in Participatory Health Research*.; 2020. http://www.icphr.org/uploads/2/0/3/9/20399575/icphr_position_paper_3_impact_-_march_2020__1_.pdf. Accessed May 24, 2020.

2. International Collaboration for Participatory Health Research (ICPHR). *Position Paper 1: What Is Participatory Health Research? Version: May 2013*.; 2013. http://www.icphr.org/uploads/2/0/3/9/20399575/ichpr_position_paper_1_defintion_-_version_may_2013.pdf. Accessed June 13, 2017.

3. Kemmis S, Nixon R, McTaggart R. *The Action Research Planner: Doing Critical Participatory Action Research.*; 2014. doi:10.1007/978-981-4560-67-2

4. Baum F, Macdougall C, Smith D. Participatory action research. *J Epidemiol Community Heal*. 2006;60(60):854-857. doi:10.1136/jech.2004.028662

5. United Nations. *Universal Declaration of Human Rights*.; 1948. http://www.ohchr.org/EN/UDHR/Documents/UDHR_Translations/eng.pdf. Accessed February 5, 2018.

6. Report of the Secretary-General, Nations U, Assembly UNG. *Strengthening the Role of the United Nations in Enhancing the Effectiveness of the Principle of Periodic and Genuine Elections and the Promotion of Democratization*.; 2013. https://digitallibrary.un.org/record/827187. Accessed April 17, 2019.

7. UN Environment Programme. Why does environmental rights and governance matter? https://www.unenvironment.org/explore-topics/environmental-governance/why-does-environmental-governance-matter. Published 2021. Accessed February 5, 2021.

8. United Nations For Indigenous Peoples. Indigenous Peoples at the UN. https://www.un.org/development/desa/indigenouspeoples/about-us.html. Published 2018. Accessed April 16, 2019.

9. Nunn JS, Sulovski M, Tiller J, Holloway B, Ayton D, Lacaze P. Involving elderly research participants in the co-design of a future multi-generational cohort study. *Res Involv Engagem*. 2021;7(1):23. doi:10.1186/s40900-021-00271-4

10. Bromley DW. The commons, common property, and environmental policy. *Environ Resour Econ*. 1992;2(1):1-17. doi:10.1007/BF00324686

11. Butler JRA, Tawake A, Skewes T, Tawake L, McGrath V. Integrating traditional ecological knowledge and fisheries management in the torres strait, Australia:The catalytic role of turtles and dugong as cultural keystone species. *Ecol Soc*. 2012;17(4). doi:10.5751/ES-05165-170434

12. Rachel Feltman. Orangutan granted rights of personhood in Argentina. *The Washington Post*. https://www.washingtonpost.com/news/speaking-of-science/wp/2014/12/22/orangutan-granted-rights-of-personhood-in-argentina/?noredirect=on&utm_term=.53d1313c54bc. Published 2014. Accessed April 17, 2019.

13. Hutchison A. The Whanganui River as a Legal Person. *Altern Law J*. 2014;39(3):179-182. doi:10.1177/1037969X1403900309

14. O'Donnell E. *Legal Rights for Rivers: Competition, Collaboration and Water Governance*.; 2018. https://www.routledge.com/Legal-Rights-for-Rivers-Competition-Collaboration-and-Water-Governance/ODonnell/p/book/9780367584160. Accessed April 26, 2021.

15. Genome British Columbia. *Genomics Positively Affects Life, Every Day*.; 2019. https://www.genomebc.ca/wp-content/uploads/2019/06/GBC-4228-2018-AR-FINAL_WEB.pdf?utm_source=Master+List&utm_campaign=8c36d44d96-ANNUAL_RPT_CAMPAIGN_2019_07_10&utm_medium=email&utm_term=0_007b42effb-8c36d44d96-96302171&mc_cid=8c36d44d96&mc_eid=40205f8c68. Accessed July 18, 2019.

16. Indigenous Corporate Training. Who owns Traditional Ecological Knowledge? https://www.ictinc.ca/blog/owns-tek. Published 2013. Accessed July 18, 2019.

17. Rubis JM. The orang utan is not an indigenous name: knowing and naming the maias as a decolonizing epistemology. *Cult Stud*. 2020;34(5):811-830. doi:10.1080/09502386.2020.1780281

18. Rubis JM, Theriault N. Concealing protocols: conservation, Indigenous survivance, and the dilemmas of visibility. *Soc Cult Geogr*. 2020;21(7):962-984. doi:10.1080/14649365.2019.1574882
