# Supplementary material for: Standardised data on initiatives—STARDIT: Beta version
Source: Res Involv Engagem. 2022 Jul 19;8:31. doi: 10.1186/s40900-022-00363-9 (PMC9294764; doi:10.1186/s40900-022-00363-9)
Supplement: Supplementary file 3 — Additional file 3. This document contains a GRIPP report about the co-creation process of the STARDIT Beta version. [file 40900_2022_363_MOESM3_ESM.pdf]

# GRIPP2 report for ‘Standardised Data on Initiatives – STARDIT: Beta Version’

This report has been completed using the ‘GRIPP2 reporting checklists: tools to improve reporting of patient and public involvement in research’ available at https://doi.org/10.1136/bmj.j3453.

## GRIPP2 short form

| Section and topic | Category description | Data |
|---|---|---|
| 1: Aim | Report the aim of PPI in the study | STARDIT development is guided by participatory action research (PAR) paradigms, which guides initiatives by aiming to involve all stakeholders in every aspect of the development and evaluation of an initiative[1,2]. Participatory research is a form of collective, self-reflective enquiry undertaken by people in order to understand their situation from different perspectives[3]. Development has also been influenced by existing work in health research, including the multidisciplinary area of public health, which incorporates social, environmental and economic research. In a health context, participatory research attempts to reduce health inequalities by supporting people to be involved in addressing health issues that are important to them, data collection, reflection and ultimately in action to improve their own health[4]. At the core of participatory research is ‘critical reflexivity’. The process asks people involved to reflect on the causes of problems, possible solutions, take any actions required which might improve the current situation, and evaluate the actions[2]. |
| 2: Methods | Provide a clear description of the methods used for PPI in the study | Formal and informal online meetings, group and individual informal and formal face to face meetings, online facilitated text-based asynchronous discussions, online voting and decision making tools, commenting on documents, completing online surveys, email discussions, informal discussions around a campfire at ‘Campfires and Science’ events. |
| 3: Study results | Outcomes—Report the results of PPI in the study, including both positive and negative outcomes | Multiple changes were made as a result of involving people, including changing the name and the logo, multiple changes to the article text (Alpha and Beta) and the STARDIT reporting tool. |
| 4: Discussion and conclusions | Outcomes—Comment on the extent to which PPI influenced | Multiple changes were made as a result of involving people and are summarised in detail in the public consultation report. These included: |

| Section and topic | Category description | Data |
|---|---|---|
| | the study overall. Describe positive and negative effects | • Plain English summary and introduction rewritten in order to provide a clearer introduction<br>• Added categories to STARDIT reporting framework about data sharing and purpose of research in preference mapping to capture more nuanced views about preferences in power sharing<br>• Added categories to the STARDIT report in order to capture data about the purpose of research<br>• Built a working version of STARDIT reports and form for submitting them<br>• Multiple other changes, summarised in the public consultation report[5] |
| 5: Reflections/critical perspective | Comment critically on the study, reflecting on the things that went well and those that did not, so others can learn from this experience | Enablers: Working with partner organisations where people are paid for their time, attending meetings and webinars already organised (rather than setting up STARDIT specific events), informal discussions with different communities of shared interest<br><br>Barriers: People were not paid for their time while being involved (except for one developer), discussions and documentation were in English language only |

# References


1. Cook T, Abma T, Gibbs L, et al. *Position Paper No. 3: Impact in Participatory Health Research*.; 2020. http://www.icphr.org/uploads/2/0/3/9/20399575/icphr_position_paper_3_impact_-_march_2020__1_.pdf. Accessed May 24, 2020.

2. International Collaboration for Participatory Health Research (ICPHR). *Position Paper 1: What Is Participatory Health Research? Version: May 2013*.; 2013. http://www.icphr.org/uploads/2/0/3/9/20399575/ichpr_position_paper_1_defintion_-_version_may_2013.pdf. Accessed June 13, 2017.

3. Kemmis S, Nixon R, McTaggart R. *The Action Research Planner: Doing Critical Participatory Action Research.*; 2014. doi:10.1007/978-981-4560-67-2

4. Baum F, Macdougall C, Smith D. Participatory action research. *J Epidemiol Community Heal*. 2006;60(60):854-857. doi:10.1136/jech.2004.028662

5. Nunn JS. *Standardised Data on Initiatives (STARDIT) Public Consultation Report – September 2019 to May 2021*.; 2021. https://doi.org/10.26181/611dfcf12c6a9.
