# Supplementary material for: Standardised data on initiatives—STARDIT: Beta version
Source: Res Involv Engagem. 2022 Jul 19;8:31. doi: 10.1186/s40900-022-00363-9 (PMC9294764; doi:10.1186/s40900-022-00363-9)
Supplement: Supplementary file 4 — Additional file 4. This document describes how the public were invited to be involved in giving feedback on ‘Standardised Data on Initiatives (STARDIT)' between 2019 and 2021. [file 40900_2022_363_MOESM4_ESM.pdf]

STARDIT

Standardised Data on Initiatives (STARDIT)

Sharing the 'who', 'how' and 'what'

Public consultation report

September 2019 to May 2021

SCIENCE FOR ALL

WIKIMEDIA
MOVEMENT AFFILIATES

About this document

This document describes how the public were invited to be involved in giving feedback on the 'Standardised Data on Initiatives (STARDIT): Alpha Version'[1] between September 24th 2019 to the end of 2019. The feedback from this process was summarised into learning points and actions which were used to inform the co-creation of the Beta version of STARDIT[2]. This report then describes the public consultation process for 'Standardised Data on Initiatives (STARDIT): Beta Version' from February 2021 to May 2021.

This report is licensed under a Creative Commons Attribution-ShareAlike 4.0 International Licence. This report has been written by Jack Nunn, Director of Science for All and PhD researcher at La Trobe University. This project is being run in partnership with the Wikipedia Journals (Wikimedia Foundation). More information can be found at **ScienceForAll.World/STARDIT**

Contents

About this document 2

Public consultation 3

- Consultation period: September 2019 to December 2019 3
- Consultation period February 2021 to May 2021 5

Learning points from the public consultations 6

- STARDIT as a project 6
- Proposed collaborative way of working 6
- Authenticity and trust 7
- Personal safety risks 7
- Life or death information 7
- Sharing power 7
- Knowledge translation 7
- Diversity and inclusion 8
- Technical considerations 8
- Readability and plain English 8
- Systematic Searching 8
- Indigenous knowledge 8

Actions and results 10

Supplementary resources 11

- Facilitation plan for public event – 1st October: London 11
- Anonymised Feedback on Beta Version 16
- Change log from Alpha Version 39

References 48

Public consultation

Consultation period: September 2019 to December 2019

The ‘Standardised Data on Initiatives (STARDIT): Alpha Version’(1) was published in September 2019. Opportunities to be involved in co-creating this version were advertised online using social media and shared via email to potential authors. Comments from co-authors were then incorporated into a series of versions, with all co-authors reviewing the final pre-print version.

The pre-print was shared online and promoted using emails, newsletter and social media. Feedback from the public gathered by:

- Emails
- Phone and video calls
- Online discussion forums
- Online forms
- Public events in London and Melbourne
- Face-to-face conversations

More information about the public event in London is shared in the next section. Relevant learning points from a **Wikimedia Youth Salon** is also incorporated into this report.

In addition, Jack Nunn (Director of Science for All) worked with a number of people to complete STARDIT reports, in order to test how appropriate and useful the data entry was. This involved a series of phone and video calls, followed by exchanging versions of STARDIT reports in order to create finalised versions.

Data from all these sources has been collated and organised into themes using qualitative thematic analysis. Event attendees were invited to ensure this report captured comments from the event. Further information about this data (including how it was collated and analysed) will be shared in the planned peer-reviewed paper ‘Standardised Data on Initiatives (STARDIT): Beta Version’.

London Event Summary

On 1st October 2019, Science for All facilitated the first public meeting about ‘Standardised Data on Initiatives (STARDIT)’. The event was facilitated by Jack Nunn (Director, Science for All) and hosted by the University College London Institute of Education, London.

Registration was free and open to anyone. People could join both in person and online. The facilitated discussion lasted three hours, with breaks. A detailed facilitation plan can be found in the supplementary materials.

Learning from the discussion has been incorporated into feedback from other sources and has not been attributed to individuals.

List of attendees

In person:

- **Jack Nunn** – Director, Science for All, Strategy Liaison and Editor for the WikiJournals, member of the Cochrane Advocacy Advisory Group, PhD candidate at La Trobe University, Melbourne (Australia)
- **Sandy Oliver -** Director of the Social Science Research Unit and Deputy Director of the EPPI-Centre, Professor of Public Policy at University College London, Editor of the journal 'Research for All'
- **Carolyn Thompson** - PhD Researcher, Institute of Zoology and University College London, Postgraduate Teaching Assistant and Lecturer, University College London.
- **Mick Mullane -** Innovation Lead, National Institute for Health Research Digital Office
- **Jim Elliot -** Public Involvement Lead, Health Research Authority (England)
- **Richard Stephens** - Patient Advocate, Co-Editor-in-Chief, 'Research Involvement and Engagement', National Cancer Research Institute 'consumer' representative

Online:

- **Chloe Mayeur –** Sciensano (Belgium)
- **Wannes Van Hoof –** Sciensano (Belgium)
- **James Ansell –** Consumers Health Forum (Australia)

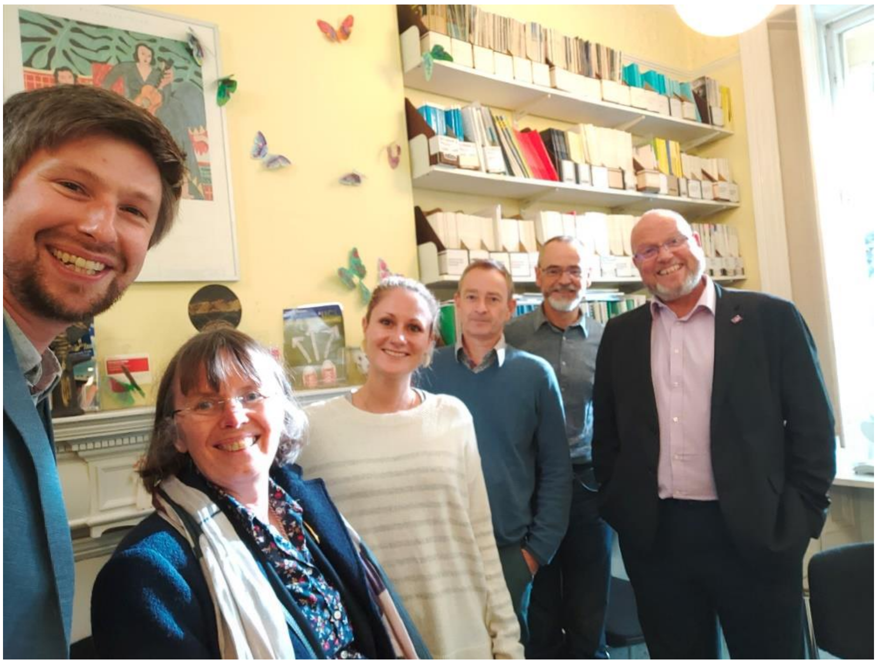

The first 'STARDIT' selfie at the London event
Left to right: Jack Nunn, Sandy Oliver, Carolyn Thompson, Mick Mullane, Jim Elliot, Richard Stephens

Consultation period February 2021 to May 2021

After the feedback from the Alpha version was collated, work began on the Beta version. Between January 2020 and February 2021 multiple meetings took place (with some face to face involvement cancelled owing to the COVID-19 pandemic).

Online video presentations and discussions

Online activities where feedback on STARDIT was invited and given include (but are not limited to) the following invited presentations and discussions:

Title	Invited by	Given by	Date
Standardised Data on Initiatives (STARDIT)[3]	WikiCite 2020 Virtual conference	Jack Nunn and Thomas Shafee (**recording, resource and transcript**)	27th October 2020
Standardised Data on Initiatives (STARDIT)[4]	Poche Centre for Indigenous Health, 9th Annual Research Showcase Program	Jack Nunn	18th November 2020
Involving people in DNA research	Poche Centre for Indigenous Health, Research Advisory Board	Jack Nunn	8th September 2020
Involving People In DNA Research	Ludwig Boltzmann Gesellschaft	Jack Nunn (recording, resource, presentation)	24th September 2020
Genomics Research and Involving People[5]	La Trobe University	Jack Nunn	13th October 2020
Involving everyone in research: Creating the evidence[6]	Australian Citizen Science Association	Jack Nunn	1st April 2021
Involving People in Rare Disease Research	Rare Voices Australia	Jack Nunn	5th August 2021

Text-based feedback and discussion

Method

A number of methods for gathering feedback and hosting online text-based discussions were used for this period of the consultation. This included using an online text-based discussion platform (hosted pro-bono by Science for All) to discuss the STARDIT Beta version, online forms for collecting feedback, online shared documents for simultaneous editing and commenting and using online pre-print servers to share stable versions[7]. A version of the Science for All STARDIT Beta webpage was archived to preserve how feedback was invited during this period[8]. Information about the consultation process was also shared by STARDIT authors via email, social media (including Twitter, Facebook and LinkedIn), and the Science for All website. Specific areas where feedback was requested included:

- Helping improve areas which are unclear or might not make sense
- Checking the STARDIT data fields are appropriate (anything missing or unclear)
- Improving the 'Example applications of STARDIT' table for your own discipline(s)
- Suggesting any relevant references that may be missing

Results

A total of 27 people provided feedback on the Beta version via the online form and collaborative document. Over 7000 words of feedback and comments were provided via the online form with 144 separate points, comments or corrections. While there were multiple small changes and comments on the collaborative document, there were 51 comments with logged changes which were 577 words in total.

All of the feedback, comments, corrections and responses by the lead authors can be found in the supplementary resources, in the section 'Anonymised Feedback on Beta Version' and 'Change log from Alpha Version'.

The final version was sent to all co-authors for checking before submitting for publication.

Learning points from the public consultations

STARDIT as a project

- The principle of standardised reporting described by STARDIT is **useful across disciplines, this is 'unique'**
- STARDIT reports will be useful for a number of disciplines, including health research, environmental research, public policy, educational interventions and community arts projects.
- Many people don't know who to trust and one participant noted that 'most of our decisions are based on trust'. **STARDIT was identified as a way of sharing data that will facilitate people to critically appraise many kinds of data**.
- STARDIT is especially **helpful for people to self-assess research** and appraise it, including supporting **informed decision making about whether to participate.**
- STARDIT was identified as a way of reporting how people were involved in defining 'shared purpose', including defining outcome measures (for example, answering the question 'what does success look like and how will we measure it')
- STARDIT could provide **an independent way for researchers and policy makers to show how people have been involved in co-producing it**
- STARDIT was identified as **a helpful tool for international development**, including planning, reporting and evaluating initiatives[9]
- STARDIT was identified as **a helpful tool for people planning, reporting and evaluating initiatives**, including mapping preferences for involvement, reporting involvement and impacts from involving people.

Proposed collaborative way of working

- While the project is 'ambitious', the proposed collaborative way of working balances openness with efficiency
- In order to make STARDIT happen, it was suggested to 'start small' and 'think like a start-up'

- A number of partner organisations were suggested throughout the public consultation including Academic Health Science Networks (UK), The National Cancer Research Institute (UK), Independent Cancer Patients' Voice (UK), Clinical Trials Units (UK), Patient Focused Medicines Development (global), National Institute for Health Research (England), Good Things Foundation (UK and Australia), Google ('Scholar' team), National Institute of Health (USA), Patient-Centered Outcomes Research Institute (USA) and the James Lind Alliance

Authenticity and trust

"This is so global and so big – it comes back to trust, how do I trust the people who report data using STARDIT"

STARDIT will be assessed by an editorial board and eventually, open peer review. It will use indicators from public domain sources. However, the root problem of authenticity and truthful reporting remains for all peer-reviewed data. While STARDIT provides data to facilitate critical appraisal, ongoing work will be required to ensure the authenticity of data. Partnering with the Wikidata project will ensure data is linked and machine-readable. Assigning Digital Object Identifiers to STARDIT reports will ensure that versions are immutable, but that the reports themselves can be updated should errors or inaccuracies be discovered.

Personal safety risks

Risks were identified with STARDIT for people who may share information or data which might have legal or safety implications. For example, data provided by members of the public about illegal activities (such as poaching or illegal logging) might incriminate individuals or put those sharing the data at personal risk. Ways of ensuring data is shared in ways which balance transparency with personal safety need to be carefully considered. China was identified by one researcher as an example of a country where special attention and cultural sensitivity would be required.

Life or death information

STARDIT was identified as a tool which could help people critically appraise information which might be life-saving or potentially lethal if incorrect. As well as medical information, this also includes information on Wikipedia pages about things such as edible fungi and plants[10].

STARDIT should have a transparent process for redacting information which might contribute to the destruction, poaching or killing of rare or threatened species, for example, not sharing detailed location information of rare species.

Sharing power

There is 'knowledge as power and powerful knowledge', STARDIT is a way of sharing both kinds of knowledge. Some 'power brokers' might not welcome knowledge sharing, transparency and scrutiny in certain areas and may actively resist attempts to share data and power. 'Power brokers' who might be resistant were identified as people working in government and for-profit organisations.

Knowledge translation

Understanding and measuring comprehension and knowledge translation are ongoing challenges in many disciplines. While STARDIT can report data on this, ongoing work will be required to ensure reporting is aligned with international best-practice. Partnering with organisations such as Cochrane

and Campbell will help ensure the reporting tool is useful. STARDIT can report transformative learning as an impact, but this will require careful tailoring to each language and culture.

Diversity and inclusion

Ensuring the process for both involving people in the development of and for using STARDIT are inclusive will need continuous reassessment, potentially requiring a group of experts and advisors. In addition, as STARDIT is developed for languages other than English, groups of people specialising in linguistic and cultural diversity will need to be involved in ensuring STARDIT is appropriate, culturally safe and inclusive. In addition, learning and development opportunities will need to be co-created with multiple stakeholders in order to ensure people are given inclusive opportunities to learn how to get involved with the STARDIT project. This was raised as a particular consideration of Indigenous peoples during one presentation to the Poche Centre for Indigenous Health.

Technical considerations

Machine learning and ‘artificial intelligence’ could be employed to create reports. Wikidata is built for machine learning and provides an open, public domain and free way of sharing data that anyone can access, anywhere. After providing a way to host reports, multiple ways to submit them should be co-created.

Readability and plain English

The ‘Standardised Data on Initiatives (STARDIT): Alpha Version’ needs to be improved for readability and plain English. In addition, the purpose and scope needs to be explained more clearly. Tailoring communication to specific disciplines should also be considered. Future versions that might be translated into other languages will require co-creation with language communities to ensure they are comprehensible to as wide an audience as possible.

Systematic Searching

Future versions of STARDIT after the Beta version will require a systematic review in order to ensure that all appropriate data sources have been consulted. As this will require a significant investment of time from those involved in the STARDIT project, it was agreed that at this stage of the co-creation process, a ‘mini-review’ (published in the peer-reviewed WikiJournal of Science) was an appropriate intermediate step to ensure the current search strategy is appropriate.

Indigenous knowledge

A report by Science for All written for the Wikimedia Foundation identified that there might be systematic, technical and cultural barriers to incorporating the knowledge of indigenous peoples into Wikipedia and other peer-reviewed repositories[10]. After additional meetings with staff from the Wurundjeri Woi Wurrung Cultural Heritage Aboriginal Corporation, it was agreed that it could be helpful to explore using STARDIT to co-create a way for indigenous peoples to share traditional and oral knowledge. STARDIT could be used to transparently report who created any content containing the knowledge, what tasks they had, how this knowledge was shared and any relevant concepts of ‘owning’ or ‘property’. Members of Indigenous communities could work in partnership with the Wikimedia Foundation to create ‘verified’ users who formally represent relevant communities are have permission to share and verify knowledge (including stories, beliefs, medicine).

The report concluded that a detailed piece of research needs to be commissioned (potentially by the Wikimedia Foundation) to explore concepts of ‘intellectual property’ and ‘owning knowledge’, and

how this respectfully interacts with the free knowledge and open access movements. Certain cultures have restricted, taboo or ‘secret’ knowledge[10]. This can include culturally significant sites which may be at greater risk of vandalism if they are shared in the public domain. STARDIT needs to be co-developed with the Wikimedia Foundation and with indigenous peoples to ensure that a balance is struck between sharing, storing and preserving unique intangible culture, while also remaining sensitive to respective cultural practices and attitudes regarding ‘ownership’.

Actions and results

1. Create a peer-reviewed scoping review to supplement the STARDIT beta paper, in preparation for a future systematic review.

   Result: Completed and ready for submission

2. Science for All to pay developers for creation of STARDIT report hosting. Create a project brief and invite developers to apply to create a beta version of STARDIT hosting.

   Result: Completed, Beta version built by paid developers (paid by Science for All, approved by the Steering Committee)

3. Host more face to face and online events in other capital cities, including Canberra and Berlin.

   Result: Abandoned. Planned face to face events in London, Berlin and other cities in 2020 were converted to online meetings and presentations

4. Rewrite parts of the STARDIT paper to be clearer and in plain English.

   Result: Completed , Beta version ready for submission in open access journal

Supplementary resources

Facilitation plan for public event – 1st October: London

Purpose of event

Create an opportunity for people from different disciplines to talk about standardised ways of reporting initiatives, including research, education and international development.

Aims

- Summarise what different disciplines are reporting about initiatives and how
- Suggest a common framework for reporting (STARDIT)
- Host a discussion about common challenges and generate ideas

Session	Summary	Instructions	Outcomes	Timing
Introductions	A chance to learn who is in the room, and what they hope to get out of today - and what the process for the afternoon is	Ask everyone in room and online to say what their area of expertise or knowledge is and why they've come today - **Online: Facilitator will summarise comments from people**	Everyone knows who is in the room and online.	15
Jack Nunn, Director of Science for All	A short presentation from Jack Nunn, Director of Science for All - about the learning from his recent projects, including his PhD about involving people in genomics research. A short summary of 'Science for All' and what led to STARDIT			10
Carolyn Thompson, Primatologist	A primatologist investigating small ape decline in China, Vietnam and Myanmar. She's working with local people, using participatory action research to investigate the patterns and drivers of critically			10

cc BY SA

	endangered gibbon decline. She will discuss how STARDIT could be useful for recording impacts from this kind of work.			
Open discussion (including input via Zoom)		Ask people online to type thoughts or comments to be read out (also check Twitter #stardit).<br>Ask people in the room to share initial thoughts, **summarise comments from people online.**	People online and in the room have contributed to discussion	5
Break - mingle - tell people online to log back on in either ten minutes to hear speakers or log back on at 2:10 to join the 'Idea Vortex' - **note Australians might be going to bed and say goodnight**				10
Reporting the what, who and how?	Short introduction to STARDIT with 3 different speakers leading discussions on how it could be used and improved.			
	Sandy Oliver - a personal and professional perspective (including thoughts on journals) - **lessons from successful reporting tools?**			7
	Richard Stevens - a personal perspective as a cancer patient and a professional perspective in relation to genomics research and on journals)<br>**What would make people want to use this?**			7
	Jim Elliot - a personal perspective and a professional perspective in relation to the work of the Health Research Authority. **What support would people need to use STARDIT?**			7
Idea vortex	Using the 'Idea Vortex' model - a series of questions designed to find issues and create solutions	**Welcome back people online!**		50

CC BY SA

Open discussion and break				10
Learning so far	A chance for anyone to speak about what has been learned so far, any reflections. Also a chance to map who's not involved who should be moving forward - and who will invite them!	**Invite comments from people online**		15
Agreeing tasks, actions and discussion areas	What actions have been agreed, what tasks and areas for further discussion	Explain how Loomio will be used going forward and how actions and decisions will be made	A list of actions, tasks and decisions to be posted on Loomio.	15
Open discussion - tea - cake - 'networking'				20
Formally close event 4pm. Adjourn to nearby pub.				

Additional Discussion Points

These additional discussion points were used to supplement the discussion:

- Can anyone write STARDIT reports? People unaffiliated with projects? Can this be one data-line that contributes to a 'living report' - in other words, could people report on behalf of organisations (like people can write Wikipedia pages about organisations without their approval)
    - Solution could be that reports make it clear when people from the organisation have been involved - verified (tick like on twitter?)
- How should peer review work?
    - In the short term, it will have to be an editorial board (volunteers associated with the WikiJournals) - we will use the existing processes of the WikiJournals for the Alpha version and Beta version
    - In the longer term - post beta version - it should be an open peer review process. For discussion
    - Peer review needs to ask the question is there evidence/data to back claims in STARDIT report - does it require some kind of standard critical appraisal tool?
- In the longer term STARDIT could 'score' projects
    - STARDIT scored- a peer reviewed score for an initiative which scores it on criteria including 'power sharing/involvement', data sharing, dissemination and translation
    - Scoring could be based on 'is there a data source for this item' so that it is not subjective (Binary yes or no on indicators of involvement)
    - Scoring continually reviewed but must be future-proofed so historical scores still have validity and use
    - This function would likely require funding/grant etc to support infrastructure - while peer reviewing would be voluntary the process of editorial control and back end would need not-for-profit investment.

STAR	Dissemination	Involvement	Translation
4.9	5	2	3

- Can things like 'documentaries' be included - who made it, who did what, who funded it? Would this be a category of 'educational intervention' - allowing documentaries to actually measure impact
- Risk of confusion with reporting guideline: STARD http://www.equator-network.org/reporting-guidelines/stard/
    - Not considered an issue by attendees
- Create API for other journals etc to use with their site? Create badge
- Partners get accredited to improve participation and recruitment
- Citing Aboriginal stories - create STARDIT report for story?

Anonymised Feedback on Beta Version

Respondee ID	Feedback with an action for the paper	General feedback about STARDIT	Change made	Lead author's response if no change made	Second Author Response	Status
2		mention of patients/public being able to complete a STARDIT report themselves, but would research teams or those responsible for patient/public activities need to deliver training to equip them with the skills to complete the form or do you envisage the reports being a joint effort?	added to discussion: 'Similarly, based on feedback from Indigenous community leaders, patient representatives and others, it is essential to ensure access to learning and development opportunities is available to support people to both access and create STARDIT reports.'			Comment incorporated into Beta
3		132 & 133	See log from tracked changes			Comment incorporated into Beta
4	Need examples earlier on exactly WHAT you are collecting -<br><br>The challenge for any non-research person reading the abstract and the lengthy preambles in the paper is always, "Yes, but WHAT data?" There are no examples given until page 10 of the main paper, where there are some very useful and comprehensive lists. Even then as a lay person I think of it as "information" not "data". All the more reason why you need examples much earlier on of exactly WHAT you are collecting - you mention responding to an epidemic, which is a great outcome, but STARDIT is about process, and that's what isn't clear.	mention responding to an epidemic, which is a great outcome, but STARDIT is about process, and that's what isn't clear.	Very helpful point, thank you. I have added this to background section 'For example, when designing a response to an epidemic, standardised data can improve retrieval of relevant information which can be used to inform which affected individuals or organisations could be involved in the design of the response and which outcomes are most important. This can include deciding which stakeholders should be involved in which tasks, such as prioritising outcomes.'<br><br>I also added this para to differentiate between raw data and metadata, as both of those are included in what we refer to as 'data' - 'Hereafter, data generated by an initiative (including raw data), information about the data (meta-data) and information about the initiative will all be referred to as 'data' unless otherwise specified.'			Comment incorporated into Beta

Respondee ID	Feedback with an action for the paper	General feedback about STARDIT	Change made	Lead author's response if no change made	Second Author Response	Status
9	line 405, Table 4: first, regarding the Section “Involvement in initiative”, Data category “Involvement appraisal”, data field “How did the initiative change as a result of involving people (did the design or evaluation change?)”. Suggest not only ask for effects of involvement on the initiative where involvement took place per se, but also on other areas.	If these categories are supposed to be described under the data category “Involvement outcomes, impacts, learnings or outputs” I would be a bit more specific, because right now, the data field description “Where there any outcomes, impacts, learnings or outputs from people begin involved?” reads to me as if this category focuses solely on effects on the people – which I think does deserve its own category – but not on what the people (and their organizations) may do based on their involvement. I do think this aspects differs from the section “Impacts and outcomes” because it may be directly correlated to the involvement of the project. I am thinking, for example, of a research project that involved local practicioners. One of them told us that he started applying the participatory methods he experienced in the research project as a participant in his work environment at a social service organization. I would not classify this as an outcome from the project per se, but from the involvement directly. It is, however, “more” than just an outcome on the people involved, but a “spill-over” from their involvement to affect others outside the involvement process.	Thank you for this, I think all the things you identified in your comment can currently be covered by this point, however I agree in future versions perhaps this could be articulated further. I have added to the help text 'These can include impacts on people, organisations, processes or other kinds of impacts' which I hope addresses your point? I will also flag with Thomas whether this section should just be added to the other impacts categories, although I note this is more of a design interface issue rather than a structured data issue.		I think it should be possible to create structured data to indicate these sorts of outcomers something like this (e.g. using "applies to part (P518)" to indicate the person and "caused by (P828)"+"involvement (Q1671829)" to indicate that it waws the act of involvement that lead to said outcome). Indeed indicating if any outcomes apply to a specific person/group may be useful in other circumstances (e.g. applies to study participants, some governmental department, NGO, entire industry sector, etc)<br><br>Having said that, I don't know whether it's going to be common enough that we'd want a whole separate section for it in terms of a data entry form. I'd be keen to have it simply as an option in the normal outputs section.	Comment incorporated into Beta

Respondee ID	Feedback with an action for the paper	General feedback about STARDIT	Change made	Lead author's response if no change made	Second Author Response	Status
9	line 405, Table 4: section "Impacts and outcomes". I am wondering whether a section about who decides on the outcomes used to evaluate the impact of an initiative may be helpful.	I think it would be quite interesting to know if the outcome measures were decided on collaboratively as well or who was in charge/what was the process of agreeing on them. I found that often those initiating the project (in our case, researchers) have quite different goals and outcomes in mind than participants – sometimes these goals can even contradict themselves. If only initiators of the project decide on outcome measures, these measures may not reflect the full reality of what the project really "should" lead to, but only a limited (maybe distorted) view that reflects the interests of only one party rather than all. Therefore	Thank you - after careful thought I agree and decided this did need extra categories. While strictly this whole section could be put into methods, I think it's important to make it distinct and clear. As a result I added 'Who was or is involved in deciding on the outcomes used to evaluate any impacts or outcomes? How were they involved?' - I note this could be two data categories but for now kept them as one row in the table just more to keep table length down than anything.			Comment incorporated into Beta
10	Note- I answered 'no' on the first two questions even though the technical answer to the question(s) is (were) 'yes' but both the Abstract and Plain-English need a but of tweaking/clarification.			Please share any further changes you think should be made		comment noted but not incorporated into this version
10	Line 38: suggest 'aims' instead of 'exists' potentially a better fit		Thank you - I went with 'was created' as it's more active and concrete			Comment incorporated into Beta
10	Line 44-45: noting open-access is repeating info given in line 39+40 so is redundant. Unsure why it is being noted here that 'authors can be verified', suggest delete or elaborate on why this is important.		changed to 'STARDIT is free to use and data can be accessed or submitted by anyone. The authors of the data can be verified (to improve trust)'			Comment incorporated into Beta
10	Line 44-45: Data being "assessed for quality" is not plain English, needs refinement and clarification.		changed to 'checked'			Comment incorporated into Beta
10	Line 46: suggest 'counter complex global problems' rather than 'improve'		changed to 'solve' as counter is not plain english			Comment incorporated into Beta
10	Line 47: delete ''being"		changed			Comment incorporated into Beta

Respondee ID	Feedback with an action for the paper	General feedback about STARDIT	Change made	Lead author's response if no change made	Second Author Response	Status
10	Line 97+98: adding a third example in the 'pandemics and air pollution' in line 97 (i.e. pandemics, air pollution and X')		added 'biodiversity destruction' although climate change could be another example to use, sadly too many to choose from			Comment incorporated into Beta
10	Line 102: putting 'research' into quote marks like the terms in line Line 103. Just for consistency and to emphasises all the terms are equally valid.		changed thank you, good spot			Comment incorporated into Beta
10	Line 49: "interventions in which affected population groups are integrally involved" is a bit convoluted. Needs tweaking/shortening.		Changed to 'STARDIT was developed on the understanding that such problems require evidence-informed collaborative methods, multidisciplinary research and interventions in which people who are affected are involved in every stage.'			Comment incorporated into Beta
10	Line 69-73: not clear as written how exactly Stardit as a mechanism leads to the benefits explained. Elaborating that it does these things through making retrieval and comparison of data easier/more efficient/faster to lead to better and quicker decisions.		Changed to 'For example, when designing a response to an epidemic, standardised data can improve retrieval of relevant information which can be used to inform which affected individuals or organisations could be involved in the design of the response and which outcomes are most important.'			Comment incorporated into Beta
10	Line 34-73: clarifying the distinction in these two sections (and in the Beta as a whole) between Stardit as a tool/concept and the Stardit repository of information.		I have worked to address this throughout but it's a valid point (and a nuanced one that's challenging to explain in plain english)			Comment incorporated into Beta
10	Line 118-120: the situation being highlighted here is bad but should tease out why exactly to lay groundwork for how Stardit will solve it.		added 'In addition to providing new standardised data categories for describing who was involved in which tasks, STARDIT can also incorporate existing data standards (see the supplementary resources 'Using Standardised Data on Initiatives (STARDIT): Beta Version Manual'), creating a unifying system for data hosting, linking and analysis. '			Comment incorporated into Beta
10	Line 115-120: This paragraph is a bit confusing and the three statements/information bits don't seem strongly linked with each other. Starts by talking about the importance of involving broad groups in initiatives generally but then ends talking about a problem of too many reporting tools in a specific type of data.					Comment incorporated into Beta
10	Line 127: unsure if 'compete' is the right word. Do we mean 'conflict'?		added 'compete or conflict' as these are distinct and important			Comment incorporated into Beta
10	Line 202: should 'Participatory Action Research' be capitalised?		changed to 'STARDIT development is guided by participatory action research (PAR) paradigms' as it's not a proper noun and the singula use implied there's only one paradigm			Comment incorporated into Beta

Respondee ID	Feedback with an action for the paper	General feedback about STARDIT	Change made	Lead author's response if no change made	Second Author Response	Status
10	Line 208: Critical reflexifivity should maybe be in quote marks and/or be capitalised given it's the name of a, uhhh, theory(?) (methodology?)		whatever it is it's certainly a jargon term (but one that can't be avoided, as that's what it's called!) so I've changed to 'At the core of participatory research is 'critical reflexivity''			Comment incorporated into Beta
10	Line 215-216: unsure what 'media freedom' means in this context and how it is relevant to Stardit. Presume that it is referring to a free media not controlled by the State but that doesn't really seem to clearly link to Stardit processes or aims.		changed to 'To uphold human rights and 'environmental rights'64, and for 'the maintenance of peace', people require 'media freedom' in order to 'seek, receive and impart information'63, free of unaccountable censorship' - I would say media freedom and censorship are likely to be at the very core of some of the reasons STARDIT might be challenged by some people with power to challenge such things			Comment incorporated into Beta
10	Line 216-217: How will/does Stardit held to 'uphold these universal rights'?		Thank you yes that was missing - added 'STARDIT has been created in order to help anyone uphold these universal rights, by providing a way to share open access information in a structured way with a transparent process for quality checking'			Comment incorporated into Beta
10	Line 281: there is an errant * against 'Genomic research' that does seem to correspond to any post-table notes		removed			Comment incorporated into Beta
10	Line 292: unclear where people are meant to start in this Figure i.e. which is step 1. adding numbers like in line 303. the line 'Report planned initiative' have the addendum 'into Stardit' into it. Similailrly 'preference mapping' should maybe be standardised/expanded to be 'Stakeholder preference mapping' as currently	it isn't clear what the term means within the figure.	Added 'Figure 2 describes how STARDIT can be used to map how people might be involved in designing, doing, reporting and evaluating initiatives, starting with 'idea sharing', to clarify the cycle, but I guess the point is you can start at any stage of the cycle<br><br>RE Pref mapping, reworded to this for clarity 'The STARDIT Preference Mapping (STARDIT-PM) tool provides a standardised way to report the preference of multiple stakeholders.' I think adding the word 'stakeholder' is redundant, as who else would be having preferences mapped? Will check with Thomas on this one		It may be worth including the redundant 'stakeholder' just to emphasise that it is for stakeholders generally (or indeed a subset of stakeholders) as readers may have implicit assumptions that preference mapping might just be for a specific group (e.g. investors) or something like that.	Comment incorporated into Beta
10	Line 303+305: Ditto prior comment about saying 'Stakeholder preference mapping' consistently for clarity					Comment incorporated into Beta
10	Line 317: citation needed for the study being referred to		added			Comment incorporated into Beta
10	Line 326: Table X needs to be updated with relevant number		Thank you good spot			Comment incorporated into Beta

Respondee ID	Feedback with an action for the paper	General feedback about STARDIT	Change made	Lead author's response if no change made	Second Author Response	Status
10	Line 346: In the fourth row of the table, the second column should maybe read “To establish the purpose, motivations and values of the research from different viewpoints” or similar		agreed - changed to 'To establish the purpose of the research, and the motivations and values of the initiative from multiple perspectives'			Comment incorporated into Beta
10	Line 406: Maybe title should read ‘Discussion and Next Steps’?		changed to 'Discussion and future versions' to reflect the content			Comment incorporated into Beta
10	Page 75- errant highlighted text Page 83- errant track change spell check to be approved		eagle-eyed! changed			Comment incorporated into Beta
10	Page 160- in the bottom row of the table, righthand column, on the second last line it should read ‘there may be formal’ not ‘the may be formal’		Incredible spot, you really did read it all! Thank you, corrected			Comment incorporated into Beta
10	Page 105, line 164- another ‘Table X’ needs number inserted		Changed!			Comment incorporated into Beta
12	Line 281: Table 1: Example applications of STARDIT. involve different parties is the evaluation of marketing authorisations, and also appraisal. Health Technology assessment is mentionned, but not medicines or medical devices regulation (authorisation) and/or appraisal/pricing.		added 'regulation and authorisation processes (for example medicines and medical devices)' to 'Production, consumerism and business ' sub category 'Other products (medical devices, electronics)'. I also added 'code and algorithm checking (for example, autonomous vehicles)' as I think this fits here			Comment incorporated into Beta
12	Line 403 - Table 4: Summary of STARDIT Beta Version data fields In data, there are different methods used to anonymise data so that individual cannot be re-identified.	It would be important to mention 1/ the risk of re-identification, 2/ the method used to de-identify data	Super important points, thank you. Reworded to 'Who controls access to the data, how are decisions about data access made? Is data anonymised or de-identified? What methods are used for re-identification? What is the risk of unauthorised re-identification? '			Comment incorporated into Beta
12	Line 403 - Table 4: Summary of STARDIT Beta Version data fields There is a varying risk of re-identification (from 0.5 to 0.05 or less, depending on which anonymization method is used and of the context).	It would be important to mention 1/ the risk of re-identification, 2/ the method used to de-identify data				Comment incorporated into Beta
12		line 346: financial relationships and other interests are mentioned; however other interests are not too detailed. I think they're key, sometimes more important to know that financial interests. You might be involved in the early phase of an exciting project, and continune until the end. When final results are disappointing, you might not be totally objective when intepreting them (participatory type conflict of interest. Other types are carrier interests, intellectual interests, conflicts between persons etc.) . Instead of simply "other interests", create a list of all types we can think of, otherwise people will not realise they should declare some.	Changed to 'Financial or other interests (including personal or professional interests)' and also added 'Describe any conflicting or competing interests (including any relevant information about authors of this report), or any other ‘interests’, including personal interest or (for example, how you may be personally or professionally affected by the outcome of the initiative)'. I'm keen to keep 'interests' open ended as an exhaustive list is impossible, but certainly in future versions we can work on standardising more types of interests			Comment incorporated into Beta
18		ABSTRACT Given how important framing at the onset is for ensuring the initiative is inviting for people (feeling like they can relate to it) and setting expectations, as I find abstracts above 250-300 words and with subsections quite rare across the 3 disciplines I work in, and some of the information seems pretty detailed for an abstract. It’s also good to end an abstract with a conclusion-focused line about contributions, impacts, and/or future directions, based on what results show to date. I thought it equally worthwhile for me to consolidate a list of initiatives, people, and resources I thought you would be interested in, as well as help you to make contact with some of those folks too. I hope you find the below comments helpful for the paper more broadly as well.				Comment incorporated into Beta

Responder ID	Feedback with an action for the paper	General feedback about STARDIT	Change made	Lead author's response if no change made	Second Author Response	Status
	LINE 63: Make this far more generic and inviting to anyone engaged in activities that have potential to inform science. Talk about the range of activities later, with broad descriptions of each, so that those unfamiliar with any or all terms can look them up and acknowledge.		reworded abstract to 'Current reporting methods lack information about the ways in which different people are involved in initiatives, making it difficult to collate and appraise data about the most effective ways to involve different people. For example, forms of participatory action research where anyone can be involved in any aspect of research (including 'citizen science') are increasingly recognised as crucial paradigms for solving global problems, as they can help ensure that initiatives are aligned with the priorities of those affected, thus redefining what it means to be a 'researcher'.'<br><br>also reworded and re-order background section so it doesn't focus on health/citizen science too early but uses them as illustrated examples.			Comment incorporated into Beta
18	**cite the following refs with the last point, as they both give an overview of terms commonly used for citsci:<br>Eitzel, M. V., Cappadonna, J. L., Santos-Lang, C., Duerr, R. E., Virapongse, A., West, S. E., Kyba, C. C. M., Bowser, A., Cooper, C. B., Sforzi, A., Metcalfe, A. N., Harris, E. S., Thiel, M., Haklay, M., Ponciano, L., Roche, J., Ceccaroni, L., Shilling, F. M., Dörler, D., Heigl, F., Kiessling, T., Davis, B. Y., & Jiang, Q. (2017). Citizen Science Terminology Matters: Exploring Key Terms. Citizen Science: Theory and Practice., 2(1). https://doi.org/10.5334/cstp.113<br>Kullenberg, C., & Kasperowski, D. (2016). What is citizen science? – A scientometric meta-analysis. PLoS One, 11(1), e0147152. https://doi.org/10.1371/journal.pone.0147152l.**		added			Comment incorporated into Beta

This report was created on 06 August 2021 by Jack Nunn for Science for All. Contact **Jack.Nunn@ScienceforAll.World** CC BY SA

Respondee ID	Feedback with an action for the paper	General feedback about STARDIT	Change made	Lead author's response if no change made	Second Author Response	Status
18	LINE 66: I am not sure why researcher is in brackets, it might be more fruitful to talk about different forms of knowledge that everyone has, which could make valuable contributions to scientific research people's knowledge.	In my experience, involved in activities that inform science may not feel comfortable with being labelled as a researcher or a scientist, but are happy to share their knowledge to help us I know more collectively. Professional researchers likewise may feel threatened by language that may be perceived as diminishing their work too, and framing around diverse forms of knowledge (e.g. experiential and cosmopolitan [vetter]) Vetter, J. (2011). Introduction: Lay Participation in the History of Scientific Observation. Science in Context, 24(2), 127-141. https://doi.org/10.1017/S0269889711000032	Changed abstract to read 'blurring the lines between concepts such as 'researcher', 'public', 'patient' and 'citizen''			Comment incorporated into Beta
18	LINE 71: [food for thought] The epidemic example seems very specific while also vague. Perhaps also adding a biodiversity or social science example could help people invasion the broad scope of this effort and how standardisation of all projects could allow for innovative cross pollination?	If wanting a medical example, projects looking at spread of mosquito-borne diseases can include medical, biodiversity (e.g. mozzies spread animal diseases like avian flu too), habitat assessment, and social impacts (e.g. who has access to different forms of preventative tools and meds).	Thank you - at the moment I thought to keep it one worked example throughout and changed it from air pollution to a pandemic, as most people naturally have a better understanding of that now - happy to use more examples throughout but for now have kept as it is as I worry it's too confusing to have too many worked examples too soon?			Comment incorporated into Beta
18	LINE 78: "co-created in a collaborative way" is redundant as "co" created is cooperative, by definition, but what you actually mean by "co-design" is the key part,	LINE 78: "co-created in a collaborative way" is redundant as "co" created is cooperative, by definition, but what you actually mean by "co-design" is the key part, as people is the term in VERY broadly and often ambiguously.	reworded: STARDIT has been co-created, involving collaboration with people from around the world in multiple ways. Informed by a number of literature reviews and guidelines, methods of involving people have included public events, online discussions and a consultation process.			Comment incorporated into Beta
18	TABLE 1: I find it interesting that there is "environmental research" but there is no mention of other activities that aren't research based necessarily (e.g. natural resource management, conservation) . It might be worth acknowledgement explicitly that are diverse ways of gaining knowledge beyond reductionist scientific methods?	This implies all projects on environmental must be research, but at a community level, this is often not the case. I also find it odd that this is under the initiative "science for all" and yet science isn't explicitly discussed much, or a key component of Table 1. I also feel it would be useful to This table seems pretty health dominant, which surprised me a bit.	I agreed with these two comments. I have created the category in table 1 called 'Management and monitoring' where I include both environmental and natural resource management, and alsp public and private essential services (e.g. water/power blur that line between natural resource and essential critical infrastructure) - but how both of these are 'managed' and monitored (or not managed and monitored) is important data to have. I also included data management and monitoring too - but note there's already a section about data and code etc.			Comment incorporated into Beta
18	have an area explicitly talking about technology, particularly in the digital age of it transforming how we gain knowledge beyond traditional conventions of science and the scientific method (e.g. e-science involves data mining, which is very different)?					Comment incorporated into Beta
18	FIGURE 2: This figure is interesting to me, and I would really like to know more about what you mean by each of the variables included, as many of those could be interpreted in a variety of ways that may not have been intent. These aspects also apply to Figure 3.	**As a designer myself, I feel like it's missing some key aspects around understanding the practices and cultures of people, their actions in a particular context, and their use of technologies, with interactive design as new practices develop. Without considering existing conditions, constraints, variables, etc in a local context, projects often fall down. The paper exemplifies exploring peoples practices and actions to understand feasibility and needs for future projects: Oliver, J. L., Brereton, M., Watson, D. M., & Roe, P. (2019). Listening to Save Wildlife: Lessons Learnt from Use of Acoustic Technology by a Species Recovery Team Proceedings of the 2019 on Designing Interactive Systems Conference - DIS '19 https://doi.org/10.1145/3322276.3322360**	Thank you for this comment - reference added. I feel that the design stage is covered in more detail in figure 3, figure 2 being the standard 'plan, do, evaluate' cycle, figure 3 being the 'design' cycle which incorporates those elements you've mentioned. If you can think of ways of making this more clear please let me know			Comment incorporated into Beta
18	ADDITIONAL ETHICAL CONSIDERATIONS Ethical considerations: I wonder, how we make sure that people running the initiatives become aware of it being added?	I have seen in project finders that projects are added by people not necessarily closely involved, and this leads to inaccurate information being in there, but if project leaders have no awareness they may not know it needs correcting. Alternatively, what if a project doesn't want to be listed? I know this to be true for a few community-driven projects that don't want to recruit broader interest, but would rather keep the project very locally focused without being contacted but those outside of the community.	Thank you for this comment, I've given this a great deal of thought too and hadn't included explicit information in this version about that scenario. The challenge is to stop the more powerful from censoring the voices of the less powerful, but also to prevent baseless slander etc (the challenge of all societies really!). I have now corrected this in the main text and further elaborated in the supplementary In future			Comment incorporated into Beta

Respondee ID	Feedback with an action for the paper	General feedback about STARDIT	Change made	Lead author's response if no change made	Second Author Response	Status
			versions it will be necessary to further develop a transparent process if a report has been created about an initiative with no involvement from anyone associated with the project, or only one subset of stakeholders. In such cases, the Editorial team might give a standard period of time for any other stakeholders to be involved in checking and editing any information (similar to the concept of 'right of reply') before the report is published, or given the status 'human reviewed'. However, the process for deciding which stakeholders to contact and how to ensure equity (alongside capacity considerations of the Editorial team) is an area for active discussion and development in future versions. For example, ensuring Indigenous peoples who are stakeholders in a mining initiative have been involved in checking a STARDIT report about a mining initiative created by a company with a declared financial interest in the mining initiative might present challenges that it is not possible for the Editorial team to overcome. Such challenges could be labelled and incorporated as future data categories in the STARDIT reports, for example labels such as 'report not checked by all stakeholders' could be updated if more stakeholders have subsequently been involved in checking any report.'			
18	FOLKSONOMY VS ONOLOGY I also wonder if there has been any consideration to creating mechanisms for a folksonomy approach to tagging projects? I have suggested with the US and Euopean groups for citsci both.		from my understanding of the term 'folksonomy' (which I only learned from your comment!) both Wikidata and therefore STARDIT are a form of folksonomy. However, as I consider it a jargon term, I'll add the reference only and have incorporated the main points in into a minor re write of this section pasted below. Perhaps it is something that can be explored in more detail in future versions of the manual, as it's important to highlight this aspect of STARDIT. 'The current Beta Version of STARDIT maps terms and concepts using the Wikidata initiative (part of the Wikimedia Foundation)36, which includes definitions (taxonomy), a way of describing relationships between concepts (ontology)37, and a system to translate definitions and ontology between many languages. Examples of existing taxonomies include the National Library of Medicine's Medical Subject Headings (MeSH), which are used extensively in multiple kinds of literature reviews 38.<br>How to involve people in combining or merging overlapping taxonomies for different subsets of data has been identified as an important question in the process of taxonomy development 5960. By using Wikidata, STARDIT can be used by anyone store both public domain data and metadata (data about data), and link to hosted structured linked data. While it is a novel element set, where possible it will also incorporate element sets from established data standards and map them where possible (see Table X in appendix for examples of data standards which could be incorporated). This includes standard elements and value sets and controlled vocabularies 61. The terms used in this paper are working terms, which will be			Comment incorporated into Beta

Respondee ID	Feedback with an action for the paper	General feedback about STARDIT	Change made	Lead author's response if no change made	Second Author Response	Status
			progressively standardised over the lifetime of the project.<br>Structured Wikidata can help define terms and concepts clearly and unambiguously, in a transparent and open way.			
18		CITSCI GROUPS I WORK WITH DOING SIMILAR THINGS & ASSOCIATED RESOURCES WORK CONSIDERING CITING I have already directly shared this info with Jack and put him in contact with associated leaders, but just so you have it all in one place, citsci initiatives worth including: SciStarter [global citsci project finder]: https://scistarter.org/ Australian project finder: https://citizenscience.org.au/ala-project-finder/ [I helped facilitate SciStarter & ALA developing an API] The EU Citizen Science Cost Action CA15212 [https://cs-eu.net/] and it's working group on Interoperability Working Group [https://cs-eu.net/wgs/wg5]; The working group's outputs are reports [https://cs-eu.net/wgs/wg5/resources] and for context, I contributed to the workshop in Geneva and subsequent report "On the citizen-science ontology, standards & data [https://cs-eu.net/news/workshop-report-wg-5-geneva-declaration-citizen-science-data-and-metadata-standards]. The initiative recently rapped up and an associated book was published [https://cs-eu.net/news/book-science-citizen-science]. Several chapters may be of interest but around data and metadata efforts for citsci, see the chapter led by Rob Lemmens. I am currently working with him, Xeni, and Ina to progress some testing of the standards based on existing projects (included Jack in an email including them and Luigi as the COST Action working group). There is also a citizen science community of practice on data interoperability that is ending this month through the EU initiative, WeObserve. There is a lot of overlap in people with the COST Action group. See the resources created on their page: https://www.weobserve.eu/weobserve-cop3-interoperability-and-standards-for-citizen-observatories/ From the US there is the CSA Data and Metadata working group [be sure and see overhead tabs too: https://www.citizenscience.org/get-involved/working-groups/data-and-metadata-working-group/], and a subset of that group, including myself has been exploring development of the PPSR Core standards. I also CCed Greg Newman as chair of that group and Brandon Budnicki who is largely responsible for pulling all of our years discussions to date into creating the publicly accessible website very recently with ability to contribute via GitHub. PPSR Core: A Data Standard for Public Participation in Scientific Research (Citizen Science) [https://core.citizenscience.org/]	Thank you - any new projects will be added to table 5			Comment incorporated into Beta
19	Two other potential examples for health and social care include: process for identifying patterns of sub-optimal service, patterns for evaluating service improvement initiatives		Thank you, added			Comment incorporated into Beta
20		overall really impressed with where this is at and the progress that has been made since I last read this work.	Thank you - all your comments addressed and responded to			
21		Added comments to the Google Drive current version	Thank you - all your comments addressed and responded to			
24		Feedback on Google doc manuscript and in email.	Thank you - all your comments addressed and responded to			

Responder ID	Feedback with an action for the paper	General feedback about STARDIT	Change made	Lead author's response if no change made	Second Author Response	Status
23		Feedback is attached as a separate document (see 'STARDIT edits_feedback ABorda 24 03 21.docx'				
23	Building on Figures 2-3. Possibly a Flow chart visual to support readers/practitioners in the development of data for a STARDIT Report using the microcategories? For example, a chart breaking down steps and possible decision points with refs to sections in Table 4, for example.			I think this is a fantastic idea. Sadly it is out of my personal capacity at this stage to create that although I think this would be good for future versions. I have flagged this with Thomas.	Agree it could be good. Alternative (or related) implementations could include:<br>- In the technical manual a summary of questions you should ask yourself for each section<br>- A stardit report for this current startit beta community feedback process<br>- An annotated video of the above stardit form being filled out	comment noted for future versions
23	(2) Due to the size of the STARDIT document – possibly divide into 3 separate standalone documents:<br>About STARDIT, MANUAL, RESOURCES.		Thank you - yes I think the idea would be (once published) the main body of the document is the STARDIT paper and then the supplementary resource is the manual, which will be as a PDF with references and additional information			Comment incorporated into Beta
23	Table 5 resources to separate into thematic/domain sections based on field categorisations? Standardise these themes possibly aligning with Tables 2 and 4 in Manual.		Yes, this is a good idea. While there are categories at the moment, perhaps a further level of categorization would be helpful? The amount of work to do this means it might need to be something for a future version but I will investigate this.			comment noted for future versions
23	(4) Consider expanding PAR section with co-design and related methods of engagement? See some suggested resources below.					
23	· Line 38 - STARDIT (Standardised Data on Initiatives) 'R' needs to be bold?Also see: Lines 69, 105		Corrected thank you			Comment incorporated into Beta
23	· Line 124 - The word stakeholders includes the public.. = use single quotes? ='stakeholders'		Thank you, done.			Comment incorporated into Beta
23	· Line 142 - used in health, environment, manufacturing , publishing, government policy, education, arts and international development - link to Table 1. ?		Thank you, done.			Comment incorporated into Beta
23	**· Line 288 - Across all disciplines, 'plan', 'do' and 'evaluate' are recognised as distinct stages of initiatives. Consider use of 'PDSA' – Plan, Do, study, Act ? a standardised iterative, four-stage problem-solving model. Check NHS publication: https://improvement.nhs.uk/documents/2142/plan-do-study-act.pdf**		**Thank you, very helpful illustration - I have added this reference to a systematic review calling for standardised reporting of PDSAs: https://qualitysafety.bmj.com/content/23/4/290.short (your link was expired)**			Comment incorporated into Beta
23	· Line 326 - Table X summarises questions - Table X?		Corrected thank you			Comment incorporated into Beta
23	ADDITIONAL RESOURCES ON FRAMEWORKS, etc		Thank you- novel references will be added into text or Table 5			Comment incorporated into Beta
23	'Proposed policy (manifestoes)' = should be: manifestos		Changed to 'Proposed policy (including draft policy and manifestoes)'			Comment incorporated into Beta
23	Suggestion to organize using a knowledge organization system? Consider Dewey Decimal 10 main classes to organize themes as outlined in table.			Good suggestion, noted for future versions as no capacity to implement at this stage		comment noted for

Respondee ID	Feedback with an action for the paper	General feedback about STARDIT	Change made	Lead author's response if no change made	Second Author Response	Status
						future versions
23	'cultural heritage ' : Suggestion to add cultural heritage to Arts section or on its own? - Large category<br>• Tangible cultural heritage: movable cultural heritage (paintings, sculptures, coins, manuscripts) immovable cultural heritage (monuments, archaeological sites, and so on) underwater cultural heritage (shipwrecks, underwater ruins and cities)<br>• Intangible cultural heritage: oral traditions, performing arts, rituals.<br>Consider revised header: 'Information, media and cultural heritage' – to : Information, media and local traditional knowledge. See comment above		Thank you - rejigged categories and created tangible cultural heritage, using UNESCO terminology			Comment incorporated into Beta
23	Referring to Table 3 Questions for mapping preferences for involvement p. 17:<br><br>To establish which group the person identifies as being part of – for example 'researcher' or 'participant'<br>Is there a table of definitions? There are several definitions in the introduction but these may be better highlighted in a table. Also note this Line 122 which suggests a blurring across some definitional boundaries?		Thank you - reworded 'To establish which grouping(s) the person identifies as being part of – for example 'researcher' or 'participant' (noting any groupings should be co-defined)'			Comment incorporated into Beta
23	Referring to Table 4: Summary of STARDIT Beta Version data fields<br>p. 21 Microcategory Section on 'Methods' - perhaps breakdown further e.g. quantitative, qualitative? Examples under each ?<br>Link to methods of approach, such as PAR, co-design, referring to relevant sections…		Change category to 'Methods and paradigms' - recognising that PAR and associated terms might be considered guiding paradigms not strictly methods themselves. Also added quant and qual as an e.g.			Comment incorporated into Beta
14	Why only 'involvement' (line 247)? In our systematic review/qualitative evidence synthesis (https://aagts.brasilia.fiocruz.br/wp-content/uploads/2020/10/Relatorio_POPART_final.pdf) on models and methods of social participation in R&D, policy, HTA, monitoring decision-making, we outlined engagement as the more comprehensive terminology, following, especially: Woolley, J.P., McGowan, M.L., Teare, H.J.A. et al. Citizen science or scientific citizenship? Disentangling the uses of public engagement rhetoric in national research initiatives. BMC Med Ethics 17, 33 (2016). https://doi.org/10.1186/s12910-016-0117-1		Thank you for this comment, the confusion around terms such as 'involvement' and 'engagement' is central to this paper and while it acknowledges there are different terms for the same thing, this paper articulates what this paper means by involvement in the section 'While meanings of these terms are often imprecise and can be used interchangeably, 'involvement' here is distinct from 'engagement', which is where which information and knowledge about initiatives is shared, for example, with study participants who remain passive recipients of interventions' - however I have added the ref 'Disentangling the uses of public engagement rhetoric in national research initiatives'			Comment incorporated into Beta
14	I understand the use of 'co-creation' here (line 253) and it is obviously adequate for the communal development approach deployed for STARDIT, but why not outline Sheila Jasanoff (2004) 'co-production' conceptualisation? It brings the systemic idea that there is the co-production of a culture that sustains and promotes the scientific knowledge productions and technological innovation while feedbacking the culture for STARDIT.		Ref added to supplementary information as we can't say co creation was informed by this but I've said 'Future co-creation processes should be decided collaboratively'.			Comment incorporated into Beta
14	line 263: I am happy to support this development and future development phases as well as operationalisation stages of STARDIT - especially regarding the proposed conceptualisation/terminology development abovementioned, if there is interest.			Thank you - support gladly recieved!		Comment incorporated into Beta
14	Table 1 line 281 - Research reporting: Why is there not 'data analysis' as a descriptor? This is a key 'research' activity to which I have been most often been asked about when I talk for experts and general public audiences to demystify misconceptions about citizen science around Brazil (especially high-ranking officers at the MoH and decision-makers at research foundations in Sao Paulo) [entire research process]: 'data validation' is also a		Thank you, added			Comment incorporated into Beta

Respondee ID	Feedback with an action for the paper	General feedback about STARDIT	Change made	Lead author's response if no change made	Second Author Response	Status
	concern for professionaly-trained researchers and decision-makers around Brazil. - as per my previous comment.					
14	Coding and algorythms reporting: Information about Data Privacy, Laws, Regulations, Directives and data security (not only pertaining to data ownership) should be mentioned here as several countries either follow GDPR, HIPPAA, GINA and Brazil, for instance adapted GDPR to its own Lei Geral de Proteção de Dados that came into effect Sept/2020.		Thank you - added to 'Management and monitoring' section			Comment incorporated into Beta
14	Other services: Jasanoff (2004) co-production conceptualisation here would be very beneficial as it outlines in which ways STARDIT can contribute to both identification and geolocalisation of impact indicators that should inform in real-time (ongoing manner) about indicators that are context-specific and may be used to develop glocally-relevent, local, regional, national indicators on various issues that remain as an unmet need somewhere, which requires data-intensive publications - it's the concept of change the culture behind the research-to-practice gap.		added ', process for identifying impact indicators (including geolocation data)'			Comment incorporated into Beta
14	involvement' (line 307): Again, do reffer back to our qualitative evidence synthesis as you may find how we have updated Rowe & Frewer's (2004) typology from Arnstein's ladder of social participation to outline that engagement is a more comprehensive terminology when it comes to citizen science and active research methods: https://aagts.brasilia.fiocruz.br/wp-content/uploads/2020/10/Relatorio_POPART_final.pdf line		Thank you -as per previous response this is covered in detail and we're using consistent terminology in this article. Have added ref - please check it's correct			Comment incorporated into Beta
14	337: OTHER OPTION: people who are not affected directly or indirectly but who hold citizenship to a certain region, country, municipality, neighbourhood and has interest in participating in decision/policy-making and/or conttributing with R&D that might be of relevance to improoving the livelihood of fellow citizens from the same region, country, municipality, neighbourhood.			thank you - I think this is covered by 'everyone' but I note this distinction. These categories are fairly fixed in this now as they were used accross the preference mapping for the alpha version but noted for future versions		comment noted for future versions
14	line 169 - REFERENCES: I have looked through your references and I see that you are familiar with Muki Haklay's amazing work on 'extreme citizen science', but I found that you may not be aware of Barbara Prainsack's work on citizen science (she had proposed in this chapter from 2013 that I have revised something very similar to what you propose here with STARDIT, but not as developed as you have done - Prainsack, B. Understanding Participation: The 'citizen science' of genetics. In: Prainsack, B., Werner-Felmayer, G., Schicktanz, S. (eds). Genetics as Social Practice. Farnham: Ashgate. Available at: https://www.researchgate.net/publication/236850804_Prainsack_B_Understanding_Participation_The_'citizen_science'_of_genetics_In_Prainsack_B_Werner-Felmayer_G_Schicktanz_S_eds_Genetics_as_Social_Practice_Farnham_Ashgate) and other publications - you may want to ask her to review STARDIT (I can mediate connection, if need be).		Thank you for offer of connection with Barbara - only just seen this - yes please for future versions! Reference added to table 5			
26	1) The definition of "initiative" is a bit novel – are there ways of highlighting what is meant by it in the context of STARDIT? One suggestion would be to have it explained in a text box, where additional level of detail and examples could be given for those that need it without disrupting the flow of the text.			I feel this is hopefully this is covered in the section 'Defining 'initiative' and 'involvement' - but a list of terms used in the paper could be a useful additional table. For consideration		comment noted but not incorporated into this version
26	1) I think STARDIT has a lot of potentially very significant contributions that could really make a difference, but I find it a bit hard to pick them out from the text. Would it be possible to make a box just highlighting the (potential) benefits/value added of STARDIT? This would be in addition to the table on the applications of STARDIT in different disciplines, and basically just be X number of bullet points explaining what STARDIT can do and why it is important.			Noted - for consideration		comment noted but not incorporated into this version

Respondee ID	Feedback with an action for the paper	General feedback about STARDIT	Change made	Lead author's response if no change made	Second Author Response	Status
26	3) It might be helpful to go into more detail, if possible, of the benefits of getting standardized data about initiatives? I.e. what does it help us to know the values of people involved in a policy initiative.		added 'Transparent acknowledgement of differing values and perspectives is critically important, in particular when mapping if different stakeholders' values are complementary or opposing'			Comment incorporated into Beta
26	4) The abstract seemed to me to be very strongly focused on methodology (citizen science and participation action research), but as I understood it STARDIT goes well beyond this. The phrasing in para two in the abstract (line 64 onwards) seems to me to suggest a delimitation of STARDIT to standardised data about who and how people are involved in initiatives, and I feel that underscores the potential contributions of STARDIT.		thank you - reworded for clarity			
26	5) A lot of initiatives will focus on evaluating another initiative, and it would be great to cross-reference all initiatives related to a specific topic or theme, especially to see how outcomes are assessed by different initiatives. I might have missed this in the paper, or the added material, but I think a way to examine the results of all evaluations of outcomes for project X would be a great benefit, including for policy and future option parts of regional and global assessments, such as those produced by IPBES and the IPCC.		added sentence 'In addition it allows comparison of both evaluation methods and any impacts or outcomes in relation to standardised terminology.'			Comment incorporated into Beta
26	Accurate and reliable data is of critical importance for all planned, collective human initiatives (see box 1). Whether we are implementing government policies, undertaking research, or developing new industry, better decisions improve outcomes – and the quality of our decisions is inextricably linked to the quality of the information we have at our disposal.<br><br>The amount and quality of data we need to make good decisions is increasing<br>exponentially as the problems we are trying to solve become increasingly complex, global and and cross-sectorial. This is reflected in the range of on-going efforts to mobilise, standardise and share data in a number of fields. However, what is currently lacking is a standardized way to share information about initiatives themselves. What was the objective, who were involved, who did which tasks, what methods were used, what was the governance structure, which results were achieved? Answers to all of these questions contain important data that could, and should, provide valuable insight and inform design and implementation of future initiatives. STARDIT' (Standardised Data on Initiatives) aims to address this gap by standardising and sharing data about human collective actions.<br><br>STARDIT is a free open access data sharing system that works across fields, disciplines and languages. Data about initiatives can be submitted by anyone, and updated throughout the lifetime of an initiative, from planning to evaluation and reporting any impacts. The authors of the data can be verified, and the data assessed for quality. STARDIT is being developed on the understanding that the complex global problems we are facing today require evidence-informed collaborative methods, multidisciplinary research and interventions in which affected population groups are integrally involved in every stage.<br><br>Among its main benefits (see box 2 for further information), STARDIT offers those carrying out research and interventions access to standardised information which enables well-founded comparisons of the effectiveness of different methods. Uniquely, STARDIT also enables sharing of information about stakeholder involvement in initiatives that works in many languages, using the Wikidata system.<br><br>This article outlines how STARDIT works and how contributors from multiple disciplines and organisations globally might continue to be involved in the development of the current Beta Version.		Thank you for this fantastic plain english summary. I have incorporated some words. Noting the word limit I will save this plain english summary to try and incorporate more if possible.			comment noted for future versions

Respondee ID	Feedback with an action for the paper	General feedback about STARDIT	Change made	Lead author's response if no change made	Second Author Response	Status
26	44-47: Lines 38-39 gives a very wide definition of what is understood by ‘initiatives’, but lines 44-47 can be read as only linking to one set of problems (i.e. complex global problems). Would it be possible to open this up slightly to stress from the very beginning the wide possible application and relevance of STARDIT? For instance:<br><br>The authors of the data can be<br>45 verified, and the data assessed for quality, offering a potentially important source of high-quality<br>46 standardised information on initiatives trying to improve complex global problems (responses to which<br>47 transcend the capacity of any single discipline).		Thank you, changed plain english summary: 'There is currently no standardised way to share information across disciplines about initiatives, including fields such as health, environment, basic science, manufacturing, media and international development. All problems, including complex global problems such as air pollution and pandemics require reliable data sharing between disciplines in order to respond effectively.			Comment incorporated into Beta
26	59-63: Is distinctions the right word here? Maybe rewrite to something like “sectorial and disciplinary barriers can limit”?		changed to 'As we face increasingly complex problems, such as global air and water pollution, disciplinary and sectorial distinctions can limit our ability to respond effectively'			Comment incorporated into Beta
26	64-65: I would perhaps not lead with this focus on citizen science and participatory action research, but if we do it would probably be best to rewrite this part: “crucial methods to use to solve”		rewritten as 'Current reporting methods lack information about the ways in which different people are involved in initiatives, making it difficult to collate and appraise data about the most effective ways to involve different people. For example, forms of participatory action research where anyone can be involved in any aspect of research (including ‘citizen science’) are increasingly recognised as crucial paradigms for solving global problems, as they can help ensure that initiatives are aligned with the priorities of those affected, thus blurring the lines between concepts such as ‘researcher’, ‘public’, ‘patient’ and ‘citizen’. '			Comment incorporated into Beta
26	66-68: Does it also miss other things that STARDIT provides? Highlighting that here might help make the value added of STARDIT even clearer.		rewritten as 'Standardised data can inform effective ways to share power during the design, implementation and evaluation stage of initiatives. For example, when designing a response to an epidemic, standardised data can improve retrieval of relevant information which can be used to inform which affected individuals or organisations could be involved in the design of the response and which outcomes are most important.'			Comment incorporated into Beta
26	69-71: “Standardised data can inform effective ways to share power during the design…” sounds a bit strange to me – I understand what is meant, but it probably isn’t quite correct (e.g. might need to specify which standardised data) and could probably be rewritten to make it more precise.		reworded to 'Standardised data can inform effective ways to plan, implement and evaluate all stages of initiatives. '			Comment incorporated into Beta
26	75-76: Is it possible to be more precise? Or, alternatively, sound more precise than “many kinds of data”?		reworded to 'STARDIT will enable multiple categories of data to be reported in a standardised way across disciplines, facilitating appraisal of initiatives and synthesising evidence for the most effective for people to be involved in initiatives. '			Comment incorporated into Beta
26	86-88: Would it be better of simply having an annex with a list of everyone that participated, and their affiliation, rather than highlighting a few organizations? It comes across as a bit strange to me (possibly because I do not understand these organizations are highlighted – but that will probably be the case for most readers).		good point. I added them for credibility but highlighting only a few is potentially sending the wrong message. removed.			Comment incorporated into Beta

Responder ID	Feedback with an action for the paper	General feedback about STARDIT	Change made	Lead author's response if no change made	Second Author Response	Status
26	95-98: This is so well established that it might be better to not mention any specific organization, but just to have a several of the most relevant references for it. Then you could also remove the “For example”.		agreed. also reworded to 'Many problems facing life on earth transcend the capacity of any single discipline to address' so it's not so human specific in relation to the problems			Comment incorporated into Beta
26	100-102: The sentence starting with “A scientific…” seems to be a put on its head somehow, especially with the part saying “in many contexts”. Maybe write: A scientific evidence-informed approach is often the most appropriate model for analysing the effectiveness of interventions.		Excellent pick up, done.			Comment incorporated into Beta
26	100-104: This para is a bit unclear to me – both the way it is phrased and its message. As I read it the message seems to be: 1. People need access to valid and reliable information. 2. Analysing the effectiveness of interventions typically requires an evidence-based approach. 3. This evidence-based approach goes under many names, including evaluation, international development, education or initiative. 4. We use the term initiative to refer to all of the above.		thank you - I have reworded as suggested.			Comment incorporated into Beta
26	109: The united Nations secretary-general stated that ‘…..		thank you, corrected			Comment incorporated into Beta
26	115: Is this wording a bit strong? Because the ‘initiative’-category is so large it probably contains a lot of examples of initiatives where inclusion it is not strictly “essential”?		changed to 'It is often essential' noting probably better wording is 'always best practice' - but that's wordy			Comment incorporated into Beta
26	117: What does “international consensus statement” mean? Could it be spelled out or explained in the sentence?		now just called it a 'statement'			Comment incorporated into Beta
26	123-124: Would it be possible to clarify what is meant by “STARDIT can report any different ‘interests’ and ways of sharing power among different stakeholders”?		**I thought about this a lot and added this sentence with a ref: An interest can include a kind of commitment, goal, obligation, duty or sense of connection which relates to a particular social role, practice, profession, experience or medical diagnosis 31. I also tried to create a wikidata entry as I don't feel there is one currently: https://www.wikidata.org/wiki/Lexeme:L483913**			Comment incorporated into Beta
26	129-131: Substitute meanwhile with another word?		changed to 'Other examples include'			Comment incorporated into Beta
26	132: Convoluted sentence? Change to: Sharing data in a consistent manner may help ensure that benefits of initiatives are shared more equitably?		changed as suggested			Comment incorporated into Beta
26	141: Move “is”, so that the sentence says: … a culture of partnership across disciplines and is, whenever possible, aligned and….”		changed as suggested			Comment incorporated into Beta
26	142: Specify that these are examples?		changed to 'such as those used in health, environment, manufacturing , publishing, government policy, education, arts and international development '			Comment incorporated into Beta
26	143: Be specific about how it works across human languages or is this evident to all/most?		sentence reordered to 'The working Beta Version of STARDIT uses Wikidata to enable definitions to be co-created by contributors anywhere in the world, and therefore works across human			Comment incorporated into Beta

Responder ID	Feedback with an action for the paper	General feedback about STARDIT	Change made	Lead author's response if no change made	Second Author Response	Status
			languages, with interoperability with other platforms planned for future versions'			
26	147: Sounds slightly underwhelming – could the benefits be highlighted better?		whole section rewritten			Comment incorporated into Beta
26	222-223: What exactly would this entail? Sounds very ambitious.		added example ' For example, STARDIT has already been used to map the varying perspectives of multiple stakeholders when planning a multi-generational cohort study 73'			Comment incorporated into Beta
26	234-235: How are they mapped and reported?		reworded to 'The participatory process used for developing STARDIT has attempted to be transparent about how different stakeholders have been involved in shaping it in order to improve how the system can be used to map values and provide more culturally neutral guidance for planning and evaluating involvement in initiatives. '			Comment incorporated into Beta
26	415: Change to: is expected to take at least 5 years, and will likely involve?		changed to 'amassing sufficient reports to create a useful database is estimated to take at least 5 years, and will likely require machine learning'			Comment incorporated into Beta
26	438-439: are is paramount		changed			Comment incorporated into Beta
26	440: in the future		changed			Comment incorporated into Beta
27	1. The current text uses terms such as “intellectual property”, “ownership”, “authorship”, and “license” in a way that is ambiguous, misleading, and internally contradictory. Unfortunately, if this critical issue is not clarified early on (i.e. now), it will create a ticking time-bomb that will go off at a later point during this project. I have been involved in open science initiatives for many years, co-founded a citizen science project, edited a guide on best practices for researchers, and received official certification from the Creative Commons organisation on copyright and licensing. In addition to my specific comments further below, please let me know if and how I can assist in fixing this problem to enable the wider sharing and implementation of STARDIT that it clearly deserves.		Any further support in correcting this would be much appreciated			
27	2. I respectfully take issue with the claim that STARDIT is useful for “any type of initiative, across any discipline” (line 278). Reading the text and tables (such as Tables 1 and 5), it seems that STARDIT is heavily informed by public health, citizen science, education, environmental, and/or international development initiatives. While commendable, they do not encompass “any discipline”. For example, would STARDIT be useful for an astrophysicist, chemical engineer, archaeologist, or historian? If so, how? If not, then the wording of “any initiative” or “any discipline” should be changed and constrained.			I would say that yes, STARDIT could be very useful for all those other diciplines and initiatives, although naturally the development so far has been by people from health and enviroment, we want to 'leave the door open' to all diciplines. While we have provided mulitple examlpes in the table of how it could be applied, I acknowledge this table is not exhaustive. I have however attempted to include data management (part of astrophysics, chemical engineering) and also cultural management in there too. I've yet to think of a collective human action that couldn't be explained with a STARDIT report, so I		comment noted but not incorporated into this version

Respondee ID	Feedback with an action for the paper	General feedback about STARDIT	Change made	Lead author's response if no change made	Second Author Response	Status
				respectfully acknowlege this point and will keep the text as is.		
27	3. Broadly speaking, I would like to see more details on how STARDIT will continue to evolve, adapt, and improve after version 1.0.		more information has been added to the supplementary section, noting that providing any specifics past version 1 would be inconsistent with the co-creation process - but the values which guide it would point towards many more versions we would hope.			Comment incorporated into Beta
27	4. Exactly who is the intended audience of this paper? As described in the “Beta Version interface” section, substantial technical skills and knowledge (such as familiarity with the usage of APIs, data science, RDF structure, etc.) are needed to make full use of STARDIT. Therefore, I don’t think literally anyone running an initiative can just jump in. Are there plans to make STARDIT more accessible and useable? If so, to whom and how?			"In short, yes I agree some of the language is very technical two parts acknowledge we need to be more inclusive (all, pending funding!) - ' and may require creating additional tools to create more inclusive ways of involving people in developing taxonomies.40' and ', ongoing co-design will be required to ensure STARDIT is as accessible and inclusive as possible. ' - which I hope are sufficient<br><br>It's hard to answer who is the audience. In summary probably anyone with a professional role in planning or managing an initiative, in particular those with a focus on involving people/citizen science "		comment noted for future versions
27	5. There is terminology not accessible to a broad, non-technical audience. For example, Wikidata is not defined on its first use on line 144.		definition of wikidata included in objectives			comment noted for future versions
27	It is not clear what the “Preferred Reporting Items for Systematic Reviews and Meta-Analyses” is which should at least come with a citation.		citation added and comma added to clarify it's a definition of what is 'Future versions should be informed by a regular, systematic search, review and appraisal processes, using the Preferred Reporting Items for Systematic Reviews and Meta-Analyses (PRISMA) data set, used for reporting in systematic reviews and meta-analyses.'			comment noted for future versions
27	Importantly, the Internet Archive is mentioned several times without explaining what it really is and why it is used.		definition added and ref			comment noted for future versions
27	Also, ORCID is not clearly defined and will be confusing for a non-technical reader. These things should be clarified.			ORCID explained in full in table 4 and not used in text anywhere else in main body of paper		comment noted but not incorporated into this version
27	The document emphasised a desire to make STARDIT “always be open access”. I wholeheartedly support the general sentiment, but the terminology and concepts employed throughout the text are unintentionally but highly misleading and sometimes contradictory:<br>The term “public domain” is used many times throughout the text. “Public domain” has a specific legal definition meaning something that is without copyright. According to national laws and international agreements such as the Berne Convention with 179 signatories, copyright is automatically applied to any intellectual work at the moment of creation with no way to opt-out. Copyright gives the copyright holder monopoly privileges and powers to dictate how the copyrighted work is to be used. This automatically applies to STARDIT and any other information/material mentioned in the current document. Colloquially, “public domain” might not be used with such specific meaning, but in the context of this STARDIT Beta version manuscript –		This is incredibly helpful feedback. I've replaced all uses of 'public domain' with 'publicly accessible' and note that these terms have been used incorrectly in a colloquial way (as I'm not qualified in this area) and this terminology absolutely needs to be unambiguous.			Comment incorporated into Beta

Respondee ID	Feedback with an action for the paper	General feedback about STARDIT	Change made	Lead author's response if no change made	Second Author Response	Status
	which explicitly mentions "open access" and licensing – it is imperative to use unambiguous language. There are too many uses of "public domain" throughout the current text, but I strongly suggest replacing those instances with "publicly accessible", "in public view", or "in the public" as appropriate (unless, of course, when the intention is to refer to the legally-defined term "public domain"). If additions have been made after the version at https://osf.io/w5xj6/, I suggest running a search-and-replace operation to find and remove ambiguous uses of "public domain".					
27	Line 91 claims that the STARDIT beta version is released under "a Creative Commons license." There are six separate Creative Commons licenses with vastly different implications on how the licensed work can be shared and used. Please be clear about which one STARDIT uses. Is it the Creative Commons Attribution license, the Creative Commons Attribution-ShareAlike license, or another one? This point applies to other mentions of these licenses throughout the rest of the text. On that note, a license is granted by the copyright holder to others who wish to make use of a work. If something is in the public domain, then by definition no license can be applied to it.<br>In addition, please avoid the ambiguous term "intellectual property" which has no specific definition. Generally speaking, "intellectual property" includes legal concepts such as copyright, patents, trademarks, trade secrets, among others. Text such as this manuscript or software code are mostly covered by copyright while physical designs like that for a machine are commonly dealt with through patents. Logos, such as the one proposed for STARDIT, are often handled through trademark registrations. Please be specific.<br>To be clear, open access usually does not mean a lack of copyright and being in the public domain. Open access is very much enabled by using open licenses (such as among the six Creative Commons licenses) to expressly grant freedoms to share and reuse information while requiring attribution. On a more pedantic note, it is possible to use the CC0 Public Domain Dedication to explicitly relinquish all copyright associated with a work and it would still technically be considered open access. However, this also means that no attribution is necessary when the work is being shared and reused, so that might not be desirable in many cases.<br>I recognise that lines 230-233 states the desire to avoid imposing a set of values on what constitutes "ownership". However, STARDIT explicitly adopts the Creative Commons licenses which operate within a copyright regime that has (unfortunately) been imposed on almost the whole world. Therefore, I stress again that terminology around copyright and licensing be made clear and unambiguous.		**Thank you for this detailed and valuable feedback. I think the entire STARDIT project should take further advice on this, but for now the most sensible decision I feel is going with a Creative Commons license and reviewing any next steps for version one - as this step gives us some control over usage of logo and other work associated with this project, without it being in danger of becoming a commercial commodity that could be 'bought out' as it were. My instinct is to go with this one (Attribution-NonCommercial-NoDerivs CC BY-NC-ND - https://creativecommons.org/licenses/by-nc-nd/3.0/au/) currently as I think not allowing anyone to change it would hopefully prevent the project 'forking' - centralising the control of what STARDIT is inside the steering committee (and any other subsequent governance processes decided upon) rather than having a free-for-all, which could work against the very thing we're trying to achieve (standardisation!). Multiple derivations of STARDIT would be a nightmare!**			Comment incorporated into Beta
27	Line 91 – Replace "a Creative Commons license" with "the Creative Commons x license" where x is the specific license that has been applied to STARDIT.		I have added information including this sentence 'STARDIT and all associated work and logos are currently licensed under Creative Commons license (Attribution-NonCommercial-NoDerivs CC BY-NC-ND), with the quality of any future iterations being the responsibility of not-for-profit host organisations and future licensing decisions to be made transparent, with anyone invited to be involved.' - note I did not add specific information to abstract for word count reasons			Comment incorporated into Beta
27	Table 1 sub-area "Coding and algorithms" – Replace "concepts of intellectual property and copyright" with "license information".		changed to this, noting that copyright etc is not a universally accepted construct or way of working '(including concepts of intellectual property, copyright and license information, relevant blockchains and non-fungible tokens), evaluating knowledge translation, reporting impacts and outcomes			Comment incorporated into Beta

This report was created on 06 August 2021 by Jack Nunn for Science for All. Contact **Jack.Nunn@ScienceforAll.World**

Respondee ID	Feedback with an action for the paper	General feedback about STARDIT	Change made	Lead author's response if no change made	Second Author Response	Status
27	Table 1 area “Information, media and cultural heritage” – Many initiatives also produce physical artefacts such as medical devices to treat malaria or a do-it-yourself solar cooker. These designs are also valuable information to be published. I suggest a sub-area titled “Hardware designs” with this “Relevant data categories” text: Reporting: Who created the designs, who reviewed them, what formats are the designs shared as and in what medium, information on license(s), outcomes and impact of the hardware.		agree this is a distinct category, added			Comment incorporated into Beta
27	Line 360 – Replace “public domain reports” with “publicly-viewable reports		done			Comment incorporated into Beta
27	• Line 366 – Replace “public domain URLs” with “public URLs”.		changed to 'publicly accessible URLs '			Comment incorporated into Beta
27	• Line 387 – Replace “will also be archived in the public domain” with “will also be archived in a publicly-accessible location online”.		done			Comment incorporated into Beta
27	• Line 393 – Replace “public domain sources” with “public sources”.		done			Comment incorporated into Beta
27	• Table 4 section “Initiative context” data category “Identifying information” – Replace “public domain URL with” with “URL”.		done			Comment incorporated into Beta
27	• Table 4 section “Initiative context” data category “Methods” – Replace “include a link to a public domain document” with “include a link to a published document”.		done			Comment incorporated into Beta
27	• Table 4 section “Report authorship” data category “Identifying information for each author” – Replace “public domain profiles” with “public profiles”.		done			Comment incorporated into Beta
27	• Table 4 section “Data” - Rename this section to “Data, software code, and hardware designs”.		changed to 'Data (including code, hardware designs or other relevant information)'			Comment incorporated into Beta
27	• Table 4 section “Data” data category “Ownership and control” – Replace “Who ‘owns’ the data or claims any kind of ‘intellectual property’ or rights (include relevant licensing information)” with “detailed licensing information”.		I've kept it plain english but added brackets 'Who ‘owns’ the data or claims any kind of ‘intellectual property’ or rights (include relevant licensing information)'			Comment incorporated into Beta

Respondee ID	Feedback with an action for the paper	General feedback about STARDIT	Change made	Lead author's response if no change made	Second Author Response	Status
27	• Table 4 section “Data” data category “Ownership and control” – Replace “public domain URL” with “public URL”.		done			Comment incorporated into Beta
27	• Table 4 section “Information completed by Editoris” (misspelling of “Editors”?) - Replace “public domain URL” with “public URL”.		done and corrected, thank you			Comment incorporated into Beta
27	• Line 485 – Replace “shared in the public domain” with “publicly shared”.		done			Comment incorporated into Beta
27	• Page 28 section “Who is involved in STARDIT?” - Replace “all decisions made transparently and in the public domain” with “all decisions made transparently and in the public”.		changed to 'In plain English, anyone can get involved and have a say in how it should be designed and run, with all decisions made transparently and stored in a publicly accessible way.'			Comment incorporated into Beta
27	• Page 31 “Additional values and paradigms” – This section mentions “STARDIT design and code should always be open access and relevant licenses should always be Creative Commons”. One of the six Creative Commons licenses must be specified here. The Creative Commons Attribution 4.0 or Creative Commons Attribution-ShareAlike 4.0 licenses are the most often used in open science. In addition, there is a separate set of licenses used for software code such as the GNU General Public License (GPL) 3.0 and that should be specified as well. It is legally highly problematic to apply any of the Creative Commons licenses to code and it should be avoided.		Changed to '• STARDIT designs and code should always be open access and relevant licenses should always be those which allow others to build on and improve the project, while maintain central control over quality (such as the Creative Commons Attribution-NonCommercial-NoDerivs CC BY-NC-ND license and the GNU General Public License (GPL) 3.0 for code)'			Comment incorporated into Beta
27	• Page 31 “Immutable values” – Replace “They will always be shared in the public domain” with “They will always be shared publicly”.		changed to 'While these values will evolve, we will keep an immutable record of our values. They will always be shared via a publicly accessible URL and regularly archived on the ‘Internet Archive’ for future reference 120.'			Comment incorporated into Beta

Respondee ID	Feedback with an action for the paper	General feedback about STARDIT	Change made	Lead author's response if no change made	Second Author Response	Status
27	• Page 44 line 17-20 (under "Data ownership and hosting" section) – Here it states a STARDIT report cannot contain any information that is not already in the public domain. If the current authors are referring to the legal term "public domain", then this will be almost impossible. For example, the vast majority of open access scientific peer-reviewed publications are under copyright and released under one of the Creative Commons licenses (usually the Creative Commons Attribution license). By definition they are not in the public domain and the information they contain cannot be included in a STARDIT report. In addition, "to avoid 'intellectual property' issues" is highly ambiguous and misleading. If the current authors are not referring to "public domain" in the legal sense of the term, the I suggest replacing the first sentence of this section with "To reduce data sharing barriers and encourage reuse, a STARDIT report cannot contain any proprietary information that is not open access".		Thank you - does this work? 'To reduce data sharing barriers and encourage reuse, a STARDIT report cannot currently contain any proprietary information that is not open access or publicly accessible, except for information volunteered by the report authors (such as institutional email addresses), much like a 'corresponding author' on a peer reviewed paper. '			Comment incorporated into Beta
27	• Page 47 lines 110-115 – The current paragraph implies that Science for All will hold the trademarks for the STARDIT logos and the copyright over the STARDIT specification, this manuscript, and other outputs from the PAR process. Is this the case (e.g. did Science for All register the trademark for the logo)? If so, please be specific. Also, if the intent is for Science for All to hold the copyrights stated above, then that implies the authors do not. Is this also intentional? The copyright holder(s) and author(s) are not always the same people. I am happy to suggest edits to this important paragraph but cannot do so without more specific knowledge of what the intent is.		Thank you - working backwards, what I'm trying to achieve is a stepping stone stage - the end goal being STARDIT set up formally as its own thing - but for now it needs a host organisation, which is Science for All (as I'm Director, so it makes things simple). In the future, if it's another charitable/not for profit organisation which takes this over, great (e.g. 'Standardised Data International - STARDIT'). What I'm trying to prevent is a situation where someone uses the STARDIT name or logo and we have no legal way of preventing it (e.g used for commercial purposes or an extreme political group). So I think for now, for all practical purposes, Science for All will be the copyright holder until a better solution is found. I'm really no expert on this so legal advice would be very welcome<br><br>Changed to 'For the purposes of concepts of intellectual property (including trademarks), and to protect STARDIT from being used by people in ways which are outside of the values defined in this document, any intellectual property (including logos or code associated with STARDIT) are currently owned by the charity Science for All, which is currently hosting the participatory action research process to create and manage STARDIT. Any decisions relating to the above (including transfer of ownership of any intellectual property) are to be made by the STARDIT Steering Committee, which hosted by Science for All, but independent of Science for All. In the future ownership may be transferred to an appropriate organisation established specifically for the purpose of owning such intellectual property, for example, establishing a charity called 'Standardised Data International – STARDIT).'			Comment incorporated into Beta

Respondee ID	Feedback with an action for the paper	General feedback about STARDIT	Change made	Lead author's response if no change made	Second Author Response	Status
27	• Table 5 – The “Access” column in this table makes a distinction between “public domain” and “open access”. This is confusing because material in the public domain counts as open access.		corrected as either Publicly accessible website or Open access			Comment incorporated into Beta
27	• Citizen science is briefly mentioned in the abstract but not defined or elaborated on in the text. I suggest just removing the mention of it in the abstract. Otherwise it needs to be defined in the text with relevant citations such as, but not limited to:<br>Auerbach, J., Barthelmess, E. L., Cavalier, D., Cooper, C. B., Fenyk, H., Haklay, M., Hulbert, J. M., Kyba, C. C. M., Larson, L. R., Lewandowski, E., & Shanley, L. (2019). The problem with delineating narrow criteria for citizen science. Proceedings of the National Academy of Sciences, 116(31), 15336–15337. https://doi.org/10.1073/pnas.1909278116<br>Silvertown, J. (2009). A new dawn for citizen science. Trends in Ecology & Evolution, 24(9), 467–471. https://doi.org/10.1016/j.tree.2009.03.017		the term 'citizen science' is used in inverted commas in the abstract, and there's no space for explanation - I think the term is familiar enough to be used in this context in the abstract, although a more full explanation is now included in the background section with added refs			Comment incorporated into Beta
27	• Line 415-416 (page 25 in Discussion section) – Why and how might machine learning be applied to STARDIT data? Without elaborating more this feels like a throwaway sentence.		added extra sentence and ref 'STARDIT seeks to be an easy-to-use way for people from multiple disciplines to share data about initiatives. However, amassing sufficient reports to create a useful database is estimated to take at least 5 years, and will likely require machine learning. For example, adversarial machine learning may be used in parallel with humans (for verifying data) to generate STARDIT reports from existing publicly accessible data at a scale and speed impossible for humans alone to achieve. '			Comment incorporated into Beta
27	• Lines 97-100 on page 46 section “How does STARDIT work within ‘law’ and ‘lore?” (missing ‘ after lore?) - In addition to indigenous peoples, there are entire sovereign states that are not recognised by the United Nations. So, if “STARDIT conduct will be guided by the United Nations on all matters of law”, how will citizens of non-UN-recognised states be heard, represented, and included?		Thank you - added this 'STARDIT will always defer to United Nations declarations in favour of any sovereign laws, and does not recognise itself as a legal entity which is acting in any one sovereign state. Citizens of non-UN-recognised states will be recognised as individual legal persons and included equally alongside any other person, regardless of status (including citizens, residents, asylum seekers and refugees). STARDIT is an initiative to support individuals to self-organise ways of sharing information, and it is the responsibility of each individual (including individuals working on behalf of organisations) to act both within the values of STARDIT, and any laws to which they may be subject. '			Comment incorporated into Beta

Change log from Alpha Version

Section	Change	Lead Author comment (Jack)	Lead author's response if no change made (Jack)	Second Author's response (Thomas)
Plain English summary	Improved first para in line with feedback			
	Made it clearer in the phases section that initial audiences might need targeting	Although I like this concept, in practice it won't be "anyone" that is submitting or bothering to standardise data. The people you are ultimately trying to target are researchers, NGOs, governments etc., so maybe worth mentioning who the specific target audiences are (including the encouragement of citizen scientists). With that in mind, it is worth considering that those people (like me!) will not be experts in standardising jargon so it may be good to be as specific as possible (using examples) throughout. Also, make it clear how STARDIT will run alongside government and academic outputs.	I think this is a wider point about STARDIT that is touched on later in the development phases and also a point about messaging and audience targeting, which I think is something to address for version One	
	Added use case for NGOs in table 1 examples of use and added this ref: https://www.brookings.edu/wp-content/uploads/2016/06/11_development_aid_kharas.pdf	Agreed, it says all those carrying out research and interventions, which covers what I say next. It may be worth highlighting somewhere in the paper that another really important factor is that it provides information to various development aid agencies (governments, NGOs etc.) about what initiatives has and are being done, which can save precious time and money for regions that can ill afford to have limited aid budgets blown on projects already done elsewhere.		
		The three main issues with aid architecture include: poor information sharing, coordination and planning; no info on results effectiveness and aid allocation rules that lead to a limited ability to scale up, learning and innovation. STARDITs design can help with all these issues! See Page 17 of this highlights this issue:https://www.brookings.edu/wp-content/uploads/2016/06/11_development_aid_kharas.pdf<br><br>I am not saying that this should be mentioned here, but it might be worth adding as a benefit somewhere else?<br><br>I know from my experience in the Pacific, there was so much money being wasted everywhere - with aid money being splashed everywhere on projects that often duplicated each other. No one had any real idea who was doing what in the Pacific? Often we would hear about a project that was closely matched to our own, but could never access documents, info etc. Different countries involved. With STARDIT there could be greater information sharing, leading to greater coordination and planning and improvements from access to info on outcomes e.g., use lessons learnt and increase the likelihood of greater success in aid projects.		

Section	Change	Lead Author comment (Jack)	Lead author's response if no change made (Jack)	Second Author's response (Thomas)
	have added 'biodiversity loss' at later point, as habitat loss is a jargon term (some people might think it means places for humans to live) but this point is about how health and enviroment are interlinked	...global air pollution and habitat loss,		
	Attempted to work in more examples	Also, examples throughout would be really helpful.		
		If a project has access to STARDIT would that have any benefits? E.g. lowered costs, improved results? Can we put some numbers/values in to add strength to the initiative?	I think this is a great idea, but beyond my capacity and expertise for the Beta version and certainly a good thing to do for version one	
	Done	Could this be framed positively as "By reporting data in a standardised way, essential information generated can provide opportunities for collaboration and comparison"		
	revised sentence strcuture but kept 'citizen science' in inverted commas as it requires defining, and using it outside commas suggests it has a fixed meaning the reader should understand, which often it doesn't.	Why is citizen science in inverted commas? Is it to suggest it is still a developing science, not yet established? Not saying it is, the inverted commas infer that.		
	clarified wording	Maybe clarify what it means to "involve people": involve people in research? Involve people in action-planning? Also, ultimately standardising data saves time/money and results in more accessible evidence-based action planning (whether that is by governments or NGOs etc.).		
		See my previous comments above. Really like the opening paragraphs, but now it seems STARDIT is aimed at 'governments, industry, research organisations and people around the world'. Needs to be clarified earlier who STARDIT is for, and how it will work alongside/in place of existing publication platforms.	the audience is 'everyone', have attempted to make use cases for different audiences clearer in the table of examlpe use cases	
		Who will do this? Will it be peer-reviewed? How is quality in the database assured? Will it have a Wikipedia model of self-review?	This is covered in detail in additional resources	
Background	added reference	This is also similar to realist evaluation, good to make this link here or later		
	excellent. Included	I know the UN and data is mentioned above but seeing that STARDIT is about multiple sectors & disciplines and that Climate Change is the biggest threat humanity faces, it might also be worth mentioning somewhere an environmental body (i.e., not just health and economics) that also outlines the importance that information and knowledge sharing has to overcoming this challenge. This could be included further down where you mention the benefits of knowledge sharing. For example, the		

Section	Change	Lead Author comment (Jack)	Lead author's response if no change made (Jack)	Second Author's response (Thomas)
		Paris Agreement highlighted the critical role of Information and Knowledge Management to Climate Change Adaptation in Article 7 that Parties "strengthen cooperation and enhance action" to share information, experience and lessons; strengthen institutional arrangements; strengthen scientific knowledge; assist developing countries to identify effective adaptation actions and improve effectiveness of adaptation actions (UNFCCC 2015). From: UNFCCC (2015) Paris Agreement. FCCC/CP/2015/10/Add.1.1 32. See: http://unfccc.int/files/essential_background/convention/application/pdf/english_paris_agreement.pdf		
	added references to GBIF and references about the limitations of such platforms	how about adding something about previous successful initiatives for standardizing data. you could also include current trends like GBIF which have a similar rationale and also open data/ open source initiatives.		
	Thank you, noted. Vulnerable has a very specific meaning in certain contexts (for example, vulneralbe adults in the mental health context) but whether this term should be applied to whole populations is a good question. I would prefer soemthing like 'at greater risk of exploitation'. Minority is problematic, as in what frame of reference is someone in a minority, and who is doing that framing? Also, very rich people are in a minority, but are not 'vulnerable'. Lots of lethal euphemisms here!	There has been criticism of this term recently eg. https://www.tandfonline.com/doi/abs/10.1080/09581596.2019.1656800?journalCode=ccph20 Perhaps "minority"?		
	added comments about data access and citations.	1. Could it be appropriate to also add in a sentence about data ownership? For example, which stakeholders/projects are more likely to share data? Comment that we need to encourage stakeholders (that own the data) and educate on the importance of sharing data to enable others to take control over their lives/decisions etc.<br><br>2. Agree this would be important to define/describe before the STARDIT part and to touch on opportunities for community data ownership, sovereignty		
Objective	covered later in paper but added 'with interoperability with other platforms planned for future versions.'	How could STARDIT continue if Wikidata suddenly disappeared? Suggest describing how STARDIT is held on a platform that enables accessible data sharing and that Wikidata is currently used		
Current usage		1. There is a growing call for scientific journals to make reviewers and editors anonymous comments and reviews part of the official scientific record. A few journals are taking it up, but resistance is there and uptake is slow, STARDIT could help to build a stronger case for it. See: Polka et al. (2018). Publish peer reviews. Nature: https://www.nature.com/articles/d41586-018-06032-w.<br><br>I am all for it to help improve transparency and help with better public perceptions of science. Although it is worth factoring in that asking reviewers to consent to being identified to the author has no important effect on the quality of the review, the recommendation regarding publication, or the time taken to review, but it significantly increased the likelihood of reviewers declining to review.<br><br>See: Van Rooyen, S., Godlee, F., Evans, S., Black, N. and Smith, R., 1999. Effect of open peer review on quality of reviews and on reviewers' recommendations: a randomised trial. Bmj, 318(7175), pp.23-27. link: https://www.bmj.com/content/bmj/318/7175/23.full.pdf?casa_token=y5JgdYfuEIcAAAAA:RsmsQAgdYlUglSnUQ-JIQHfIEVQnJA2yEwHmLIKkK5F-KSyBKrHCavVYKOd9YIgUx6c-WeZltsmo1A	I think this is an important issue (open peer review) but one that is outside the scope of this paper. In other words, open peer review is already happending (and in fact is used by the Wiki Journals and the journal where this will be submitted) and in most cases where	Partial solutions also involve embargoing comments and/or reviewer identities

Section	Change	Lead Author comment (Jack)	Lead author's response if no change made (Jack)	Second Author's response (Thomas)
		2. I agree with Roan's comments. I'm all for publishing the reviewer comments, as this will give much better transparency. However, if you don't make them anonymous you are going to find it very difficult to find people to review articles, and people will start to be less critical, especially when the community is small. It's already incredibly difficult to find experts with free time to review.	I've been part of open peer review process you can chose to be anonysmous or not.<br><br>Regardless of the benefits or cons - the point is that STARDIT can be used to report information about peer review process - it's not in itself an open peer review process. How the editorial process works for future STARDIT reports is also an open discussion.<br><br>Comments flagged with Thomas for his input as he has superior expertise in this area	
	added more examples with references	This is great! Could we mention an example to explain the benefit of STARDIT? E.g. What new information was shared though STARDIT that wasn't published by the journal (assuming this is where it was published)? How could this information be useful to future initiatives?		
Potential applications		This application could be mentioned earlier as a way of describing the benefit of STARDIT, e.g. 'one of the advantages of standardizing data reporting is that comparisons between methods and impacts across multiple initiatives can easily be made.'	mentioned in abstract - repitition if mentioned in introduction?	
	Added this para with references 'In addition, STARDIT could be used to share information which makes research more reproducible43,44, improving accessibility to the information required to critically appraise research and evidence and thus improving trust in processes such as the scientific method45,46, and facilitate an appraisal of different knowledge systems, including Indigenous knowledge systems47.	STARDIT might also help with the scourge of irreproducible scientific studies. Tens of billions of dollars are wasted each year on irreproducible research, where insufficient information on methodology and data prevents many studies from being reproduced. With 14% of scientists reporting that they have witnessed scientific fraud (Fanelli 2009), there is a push for more stringent demands of proof from investigators/ scientists (Clark 2017) e.g., video taping of experiments and full raw data. STARDIT could facilitate this. Examples of the issue, see: Clark, T.D., 2017. Science, lies and video-taped experiments. Nature News, 542(7640), p.139. Link: https://www.nature.com/news/science-lies-and-video-taped-experiments-1.21432 AND Fanelli, D., 2009. How many scientists fabricate and falsify research? A		

Section	Change	Lead Author comment (Jack)	Lead author's response if no change made (Jack)	Second Author's response (Thomas)
		systematic review and meta-analysis of survey data. PloS one, 4(5), p.e5738. link: https://journals.plos.org/plosone/article?id=10.1371/journal.pone.0005738		
		there is also an opportunity to improve our overall understanding of social and environmental problems by embracing the strengths and weaknesses of the different knowledge systems (UNESCO 2017) UNESCO, 2017: Local knowledge, global goals. Local and Indigenous Knowledge Systems Programme Rep., UNESCO, 48 pp., http://unesdoc.unesco.org/images/0025/002595/259599E.pdf		
		...also help to improve public trust in scientific method and outcomes. e.g., https://www.nature.com/articles/nbt1199supp2_14 and https://journals.sagepub.com/doi/pdf/10.1177/0963662519869097		
	Added in this sentence: Such data sharing could also improve the translation of trusted, quality research and data, by empowering people to both access and appraise relevant data. For example, improved access to more standardised information (in multiple languages) about data and outcomes, could help to facilitate more informed collaborations between researchers and those monitoring and protecting critically endangered species48–50.	For a multidisciplinary environmental benefit that STARDIT could also help with: As with health and other disciplines, there is a big issue with a lack of knowledge translation between scientists and conservation managers (see Linklater 2003for an example). With the ongoing biodiversity crisis (Ripple et al. 2017), having improved access to more standardised shared project information, data and outcomes, could help to facilitate a better understanding of the quality of what work has been done and lead to more informed collaborations between managers and researchers, perhaps to more applied projects on conservation management focussed research for improved decision making when managing critically endangered species. An example of how a lack of standardised info and data can lead to poor outcomes, rhino conservation managers had to rely on several non-standardised home range study sources for black rhino, incorrectly interpreting the impact of the non-standardised studies, and initiated actions that had significant detrimental impacts for a key black rhino population (See Plotz et al. 2016).<br>References:<br>Linklater, W.L., 2003. Science and management in a conservation crisis: a case study with rhinoceros. Conservation Biology, 17(4), pp.968-975.Doi: https://doi.org/10.1046/j.1523-1739.2003.01449.x<br><br>Plotz, R.D., Grecian, W.J., Kerley, G.I. and Linklater, W.L., 2016. Standardising home range studies for improved management of the critically endangered black rhinoceros. PLoS One, 11(3), p.e0150571. Doi: https://doi.org/10.1371/journal.pone.0150571<br><br>William J. Ripple, Christopher Wolf, Thomas M. Newsome, Mauro Galetti, Mohammed Alamgir, Eileen Crist, Mahmoud I. Mahmoud, William F. Laurance, 15,364 scientist signatories from 184 countries, World Scientists' Warning to Humanity: A Second Notice, BioScience, Volume 67, Issue 12, December 2017, Pages 1026–1028, https://doi.org/10.1093/biosci/bix125		
	made it clearer that sometimes meanings are vague and that we are therefore defining what they mean in this article: While meanings of these terms are often imprecise and can be used interchangeably, 'involvement' here is distinct from 'engagement', which is where which information and knowledge about initiatives is shared, for example, with study participants who remain passive recipients of interventions.54	Not sure this is the best example, engagement is surely more than just being a study participant with no input or contribution. Agree involvement is a more active term than engagement		

Section	Change	Lead Author comment (Jack)	Lead author's response if no change made (Jack)	Second Author's response (Thomas)
	Added 'see Table X in appendix for examples of data standards which could be incorporated' as there's a big list in what is currently table 5	Such as? As someone who comes from a very niche background, it would be useful to know what other established data standards can/are used.		
	added 'Development has also been influenced by existing work in health research, including the multidisciplinary area of public health, which incorporates social, environmental and economic research. '	and say that this can be applied across disciplines? Public health is already multidisciplinary, incorporating economics, environment, social etc.		
	added ref	Another example of community ownership and TEK principles in WA: https://researchonline.jcu.edu.au/24751/1/ES-2012-5165.pdf		
	added ref	also relevant is O'Donnell, E.L. and Talbot-Jones, J., 2018. Creating legal rights for rivers. Ecology and Society, 23(1). http://www.ecologyandsociety.org/vol23/iss1/art7/; https://www.routledge.com/Legal-Rights-for-Rivers-Competition-Collaboration-and-Water-Governance/ODonnell/p/book/9780367584160		
	added 'In addition, ongoing co-design will be required to ensure STARDIT is as accessible and inclusive as possible. '	I think this is such an important paragraph! Also, the article touches on this, but are we not excluding different types of people/cultures already by asking them to fill in these reports? I am just thinking of people I work with in Indonesia and China, many of whom, would not respond well to a wordy document/guidelines and would need strong benefits to encourage them to use STARDIT (which goes back to one of my original points).	This is a very important point, and one raised by an aboriginal community member too, it comes down to accebility and if there's training in place. I've mentioned this in limitations but also, frankly, it's a limiation of ALL kinds of publishing and any online tool. If anything, being able to complete it in other langauges makes it more accecible but ongoing co-creation is needed to keep improving it (including training)	
	added '	Is this the first mention of Science for All? Maybe some more information on who they are, their values and objectives?		
Version One implementation	changed to 'Once STARDIT Beta (version 0.2) has been **submitted** for publication, work will begin on the next version, (version 1.0). '	Perhaps confidence in the current version should enable version 1.0 work to commence regardless of publication status. The journal may not want to publish an interim version		

Section	Change	Lead Author comment (Jack)	Lead author's response if no change made (Jack)	Second Author's response (Thomas)
	I have added 'more detail in appendicies'	would be useful to describe the make-up of the working group, in categories e.g. researcher, citizen scientists or as a figure?	I'm keen to keep it brief in the main body and also open ended (not too prescriptive) but I have added 'more detail in appendicies'	
		How did Wikipedia become so universally adopted? Any lessons or examples there we can use to improve adoption and reporting into STARDIT?		
		How did Wikipedia become so universally adopted? Any lessons or examples there we can use to improve adoption and reporting into STARDIT?	I think this is outside of the scope of the paper but the short answer from me would be transparent governance and editirial processes, which we've emulated with STARDIT	
Scope and applications		Agreed. As an fyi, there is a regional information and knowledge management system called iCLIM. It is not across any discipline but many of the principles align with STARDIT's objectives. The Pacific iCLIM Project aims to enable better climate change resilience and adaptation planning in the Pacific by improving the discoverability, storage, access, and utilisation of climate change data and information. For more info see here: https://www.griffith.edu.au/research/research-excellence/griffith-climate-change-response-program/pacific-iclim and a situation analysis here: https://www.redicomar.com/wp-content/uploads/2019/12/Pacific-Situation-Analysis_Pacific-iCLIM-Project-2019.pdf		
Table 1: Example applications of STARDIT		The Table is great. In the first two pages of it, there is a lot of repetition of categories that overlap across all the different Areas & sub-areas (e.g., people affected/ involved; process for deciding and measuring outcomes; experts involved; in most cases 'funding'). Just a suggestion: could all the similar categories that occur within and across all the areas/ sub-areas be collated in a separate table to say - 'these categories will be recorded as standard across all areas, and then have this table show any additional categories that are uniquely/ specifically recorded for each of these different application areas. It might make it easier for the reader to understand what is being recorded for their area of interest and make more meaningful comparisons. As it is now, I found it a bit hard to absorb all the various categories relevant to specific areas with all the repetition. Also, an added benefit once all the similar/standard categories across areas are evaluated and collated, allows easier comparisons to evaluate whether any of the categories currently listed should actually be recorded for that area or if a category currently not considered in area should actually be recorded and vice versa. The added complication is that this is an evolving tool and categories that are currently listed within an area might	I think this is a great idea but I just didn't have the time to do this right now. Perhaps this could be something we work on for future version when trying to communicate it to dofferent audiences?	

Section	Change	Lead Author comment (Jack)	Lead author's response if no change made (Jack)	Second Author's response (Thomas)
		shift, and others likely to be added and removed over time - we could add an appropriate caveat to indicate that.		
		important to engage with Indigenous people around this content, hope this has occurred through Poche Centre engagement but if not yet, suggest engaging directly with Boe	Thank you - yes, STARDIT presented at multiple Poche Centre meetings, and feedback invited and incorporated	
	Thank you - incorporated and added refs	Data custodianship' might cover this but might be worth mentioning whether the initiative followed any local, national and international legislation and policies that might be in place to govern the way in which TK is collected, documented, and stored. When projects involve TK, there is a need to be aware of any legal frameworks that may apply, including those designed to protect cultural and intellectual property (IP). This can be in the form of national<br>policies and Acts and /or local cultural restrictions. This will vary across the globe.<br>In short, have initiatives documenting Traditional and Indigenous Knowledge considered the following key issues:<br>(1) legal and national contexts; (2) prior informed consent; (3) cultural restrictions;<br>and (4) IP rights. For example, the use of prior informed consent is part of a best practice approach. In the Pacific, we provided information to participants about the project, including project purpose, who was involved, methods of collection and dissemination and consent was asked, including the level of sensitivity of the information e.g., low =publicly available, medium = known only to community or knowledge holder and can only be shared with requested permission from the knowledge holders; High sensitivity (spiritual info/ customary laws) = to remain with knowledge holder and within database.<br>For specific example see:<br>Malsale, P., Sanau, N., Tofaeono, T.I., Kavisi, Z., Willy, A., Mitiepo, R., Lui, S., Chambers, L.E. and Plotz, R.D., 2018. Protocols and partnerships for engaging Pacific Island communities in the collection and use of traditional climate knowledge. Bulletin of the American Meteorological Society, 99(12), pp.2471-2489. DOI: DOI: https://doi.org/10.1175/BAMS-D-17-0163.1		
		I think a measure of gender representation/ inclusiveness in initiatives involving IK needs specific consideration here? Indigenous Knowledge, and use, of environmental information can vary and are often segregated according to gender. In many IK projects and outputs the female voice is absent. It is therefore important to consider gender inclusiveness when collecting IK information, particularly as the impacts of environmental variability can impact genders differently in many remote and vulnerable regions. E.g., Balakrishnan, R., 1998: The Pacific. Rural Women and Food Security: Current Situation and Perspectives, Food and Agriculture Organization of the United Nations, 50–60, https://www.fao.org/3/W8376E/w8376e05.htm. AND Lane, R., and R. J. G. McNaught, 2009: Building gendered approaches to adaptation in the Pacific. Gend. Dev., 17, 67–80, https://doi.org/10.1080/13552070802696920.		
		There is an example of a database for Traditional Knowledge forecast indicators in the Pacific Islands that was designed with such added levels of security to culturally sensitive information, through		

Section	Change	Lead Author comment (Jack)	Lead author's response if no change made (Jack)	Second Author's response (Thomas)
		limiting access according to restrictions imposed by the TK expert/ community who initially provided the information.<br>For info see: Chambers, L.E., Plotz, R.D., Dossis, T., Hiriasia, D.H., Malsale, P., Martin, D.J., Mitiepo, R., Tahera, K. and Tofaeono, T.I., 2017. A database for traditional knowledge of weather and climate in the Pacific. Meteorological Applications, 24(3), pp.491-502. doi: https://doi.org/10.1002/met.1648<br><br>The TK Database needed to enable restrictions based on things such as:<br>user's membership of a clan or tribe;<br>user's status/role within the tribe;<br>user's gender, and<br>the context in which the resource will be reused or reproduced etc.		
	added	ethics important to clarify here, with additional ethics requirements for research involving Indigenous people		
	Thank you - incorporated and added refs	I have said some of this before in another comment here. e.g., free, prior and informed consent. Also worth considering sui generis systems based upon customary law (ref: https://mckinneylaw.iu.edu/iiclr/pdf/vol17p67.pdf). For example, this is straight from from UNESCO 2017 Indigenous Knowledge document: "Many communities are calling for the protection of their knowledge from inappropriate use, emphasising the need for free, prior and informed consent and benefit sharing. Existing intellectual property regimes are ill-adapted to indigenous knowledge. More appropriate methods are being developed, such as sui generis systems based upon customary law. There is also knowledge that the community may want to keep for themselves (e.g. locations of sacred groves and preferred harvesting areas). It is important to understand the different types of knowledge, and both the individual's and community's right to control access. "<br><br>UNESCO, 2017: Local knowledge, global goals. Local and Indigenous Knowledge Systems Programme Rep., UNESCO, 48 pp., http://unesdoc.unesco.org/images/0025/002595/259599E.pdf✎ EditSign		
	done - good suggestion	Add a referenced definition? e.g. UK MRC guidance?		
	added	Indigenous knowledge might need a caveat here, due to cultural sensitivity and ownership issues limiting full immediate transparency.		
Mapping preferences for involvement	added to objectives	I think it would be good to touch on/briefly introduce this tool earlier on e.g in the aims		
	added	Reference missing.		
	Done - and changed strucuture of discussion	Highlight more STARDIT strengths in these early paragraphs e.g. the strength of co-design in its development, interdisciplinary nature etc.		
	changed to 'estimated' - there's no preceedant really, but GRIPP2 is quite old now and one review says it's not very well used	How or on what basis is 5-years deemed the likely time-frame? Any precedence or examples to compare -e.g., wikijournal?		

References

1. Nunn J, Shafee T, Chang S, et al. Standardised Data on Initiatives - STARDIT: Alpha Version. 2019. doi:10.31219/osf.io/5q47h
2. Nunn JS, Shafee T. Standardised Data on Initiatives – STARDIT: Beta Version. doi:10.31219/osf.io/w5xj6
3. Nunn JS. "Standardised Data on Initiatives" WikiCite/2020 Virtual conference - Meta. https://meta.wikimedia.org/wiki/WikiCite/2020_Virtual_conference. Accessed May 6, 2021.
4. Nunn JS. Standardised Data on Initiatives (STARDIT), 9th Annual Research Showcase Program. In: ; 2020. https://pochehealth.edu.au/2020/12/20/9th-annual-research-showcase-program/. Accessed May 6, 2021.
5. Nunn J. Genomics Research and Involving People - PhD presentation by Jack Nunn 13.10.20. October 2020. doi:10.26181/5F8664AE962A9
6. Australian Citizen Science Association. EMCR Seminar Update. https://citizenscience.org.au/2021/03/24/emcr-seminar-update/. Published 2021. Accessed May 6, 2021.
7. Nunn JS, Shafee T. Standardised Data on Initiatives – STARDIT: Beta Version. doi:10.31219/OSF.IO/W5XJ6
8. Science for All. Beta - Science For All. https://web.archive.org/web/20210805234623/https://scienceforall.world/stardit/beta/. Accessed August 6, 2021.
9. Oliver S, Gough D, Copestake J, Thomas J. Approaches to evidence synthesis in international development: a research agenda. *J Dev Eff*. 2018;10(3):305-326. doi:10.1080/19439342.2018.1478875
10. Nunn J. *WikiJournal Youth Salon Evaluation Report - Science for All*.; 2019. https://web.archive.org/web/20191219020842/https://meta.wikimedia.org/wiki/File:WikiJournal_Youth_Salon_Evaluation_Report_-_Science_for_All.pdf.
